# Mitochondrial NADH-redox inflexibility constrains genomic and epigenetic stability in pluripotent stem cells

**DOI:** 10.1038/s44318-026-00784-2

**Published:** 2026-05-27

**Authors:** Tanveer Ahmed, Hui Zhang, Ghulam M Mushraf, Yongzhang Pan, Zeyu Zhang, Zhenzhu Sun, Mengran Yin, Xueting Xu, Xinyu Wu, Yin Gao, Yan Li, Peizhi Li, Liang Ding, Qiang Zhang, Runxia Lin, Khair Ullah, Yinghua Huang, Bushra Mirza, Yifeng Huang, Abdul Sammad, Xiangqian Kong, Dajiang Qin, Miguel A Esteban, Lulu Wang, Baoming Qin

**Affiliations:** 1 Guangzhou Institutes of Biomedicine and Health, Chinese Academy of Sciences; Guangdong Provincial Key Laboratory of Stem Cell and Regenerative Medicine, Guangzhou, 510530 China; 2 Cadson College of Pharmacy, University of the Punjab, Lahore, Pakistan; 3 Bioland Laboratory (Guangzhou Regenerative Medicine and Health Guangdong Laboratory), 510005 Guangzhou, China; 4 Medical Science Program, Faculty of Medicine, Chulalongkorn University, Bangkok, 10330 Thailand; 5 Key Laboratory of Biological Targeting Diagnosis, Therapy and Rehabilitation of Guangdong Higher Education Institutes, The Fifth Affiliated Hospital, Guangzhou Medical University, Guangzhou, 510700 China; 6 Department of Biochemistry, Quaid-i-Azam University, Islamabad, 45320 Pakistan; 7 The Fifth Affiliated Hospital of Guangzhou Medical University-BGI Center for Integrative Biology, The Fifth Affiliated Hospital of Guangzhou Medical University, Guangzhou, 510700 China; 8 3DC STAR Lab, BGI CELL, Shenzhen, 518083 China; 9 Prince Fahad bin Sultan Research Chair for Biomedical Research, University of Tabuk, Tabuk, 47713 Saudi Arabia

**Keywords:** Metabolism, Stem Cells & Regenerative Medicine

## Abstract

The electron transport chain (ETC) is essential for NAD^+^ regeneration and proliferation. While many cell types tolerate ETC inhibition when pyruvate or aspartate is supplied, pluripotent stem cells (PSCs) enter a reversible paused state even at abundant pyruvate levels. Here, we show that ETC inhibition triggers severe NADH reductive stress in mouse embryonic stem cells (mESCs), driven mainly by threonine dehydrogenase (TDH). TDH-derived NADH establishes a metabolic environment that disfavors cells with compromised mitochondrial function, maintains inhibition of pyruvate dehydrogenase (PDH), and is associated with increased genomic and epigenetic stability at the cellular population level. ETC inhibition similarly induces pausing in early mouse embryos and in human pluripotent stem cells (hPSCs). In hPSCs, combined inhibition of the one-carbon metabolism enzymes serine hydroxymethyltransferase (SHMT1/2) and methylenetetrahydrofolate dehydrogenase 2 (MTHFD2) effectively reduced reductive stress and rescued the paused phenotype. Together, these findings support a model in which limited mitochondrial redox adaptability represents a conserved metabolic feature of pluripotent stem cells and in which NADH reductive stress is associated with genomic and epigenetic stability.

## Synopsis

Inhibition of the electron transport chain (ETC) triggers NADH reductive stress and a reversible paused state in pluripotent stem cells (PSCs). This study delineates threonine dehydrogenase (TDH) and one-carbon metabolism as distinct metabolic sources for ETC dysfunction-induced stress and associated genomic and epigenetic instability in mouse versus human pluripotency.

- TDH contributes to NADH reductive stress and facilitates ETC inhibition-induced pausing in mouse ESCs.
- TDH-driven reductive stress constitutes a metabolic constraint that passively selects against cells with compromised mitochondrial function.
- This intrinsic reductive stress suppresses pyruvate dehydrogenase activation and associates with a reduction in genomic and epigenetic abnormalities.
- ETC inhibition pauses early mouse embryos and human PSCs, which is rescued by combined inhibition of one-carbon metabolism enzymes SHMT1/2 and MTHFD2.

## Abstract (web summary)

Threonine dehydrogenase and one-carbon metabolism constitute distinct metabolic sources for ETC dysfunction-induced metabolic stress in mouse versus human pluripotency.

## Graphical abstract

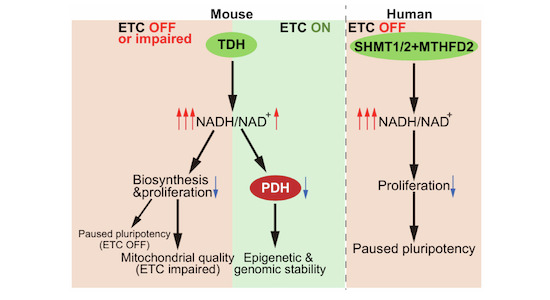

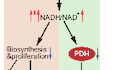

## Introduction

The electron transport chain (ETC) transfers electrons from NADH and FADH_2_ to oxygen and generates ATP through ATP synthase (complex 5). Concurrently, the ETC facilitates the recycling of NAD^+^ and FAD in both mitochondrial and cytosolic compartments, which is essential for sustaining redox metabolism, including the tricarboxylic acid (TCA) cycle and one-carbon (1C) metabolism pathways that are integral for cell proliferation (Hosios and Vander Heiden, 2018). Dysfunctional ETC leads to an accumulation of NADH and FADH_2_, a state known as reductive stress, which inhibits many NAD^+^- and FAD-dependent metabolic enzymes and pathways essential for cell proliferation. Interestingly, it has long been known that supplementing with extra pyruvate can rescue the proliferation defects in cells with ETC blockade, but the detailed mechanism remained elusive until recently. Two studies clarified that extra pyruvate recycles NAD^+^ during ETC inhibition to enable alternative aspartate production, which is essential for protein and nucleic acid biosynthesis (Birsoy et al, 2015; Sullivan et al, 2015). Additionally, adding aspartate alone rescues the proliferation defects caused by ETC inhibition without alleviating the reductive stress. Thus, proliferating cells can exhibit high flexibility in rewiring redox metabolism to adapt to ETC impairment or inhibition.

Unlike somatic cells and other types of stem cells, pluripotent stem cells (PSCs) are distinguished by their self-renewal capacity and ability to maintain pluripotency and genomic and epigenetic integrity. Metabolism is crucial in supporting rapid self-renewal and epigenetic regulation of PSCs (Jackson and Finley, 2024). For example, mouse embryonic stem cells (mESCs) rely on threonine, while human ESCs rely on methionine to support self-renewal and pluripotency-related histone methylation (Shiraki et al, 2014; Shyh-Chang et al, 2013; Wang et al, 2009). Similarly, ETC controls the self-renewal of PSCs, with its impact on pluripotency appearing to differ between primed and naive PSCs (Carbognin et al, 2016; Khoa et al, 2020; Tsogtbaatar et al, 2020). Notably, both mouse and human PSCs are typically cultured in media supplemented with pyruvate, yet their self-renewal is still arrested when the ETC is blocked. This raises important questions about whether the stem cell redox metabolism network is unique and, if so, why this is the case.

In this study, we demonstrate that the paused pluripotent state induced by ETC inhibition in mESCs is not caused solely by aspartate limitation or insufficient pyruvate but is instead contributed to by sustained NADH reductive stress. We identify the mitochondrial threonine dehydrogenase (TDH) pathway as a major source of this stress, as it continues to generate NADH even when the ETC is inhibited. Our proof-of-principle models suggest that this property creates a metabolic environment that passively filters out cells with compromised mitochondrial function. Importantly, TDH also maintains intrinsic reductive stress under normal conditions, thereby imposing a metabolic constraint that limits PDH activation and is associated with reduced genomic and epigenetic stability. We further show that ETC inhibition induces a developmental pause in mouse blastocysts and 2-cell (2 C) embryos. Finally, ETC inhibition elicits a paused pluripotent state in human PSCs through reductive stress generated by the one-carbon metabolism pathway. Together, our findings reveal a distinctive redox metabolic feature of PSCs and uncover a previously unappreciated consequence of reductive stress in shaping PSC population dynamics.

## Results

### TDH maintains reductive stress and mediates ETC inhibition-induced pausing in mESCs

**Figure EV1:**
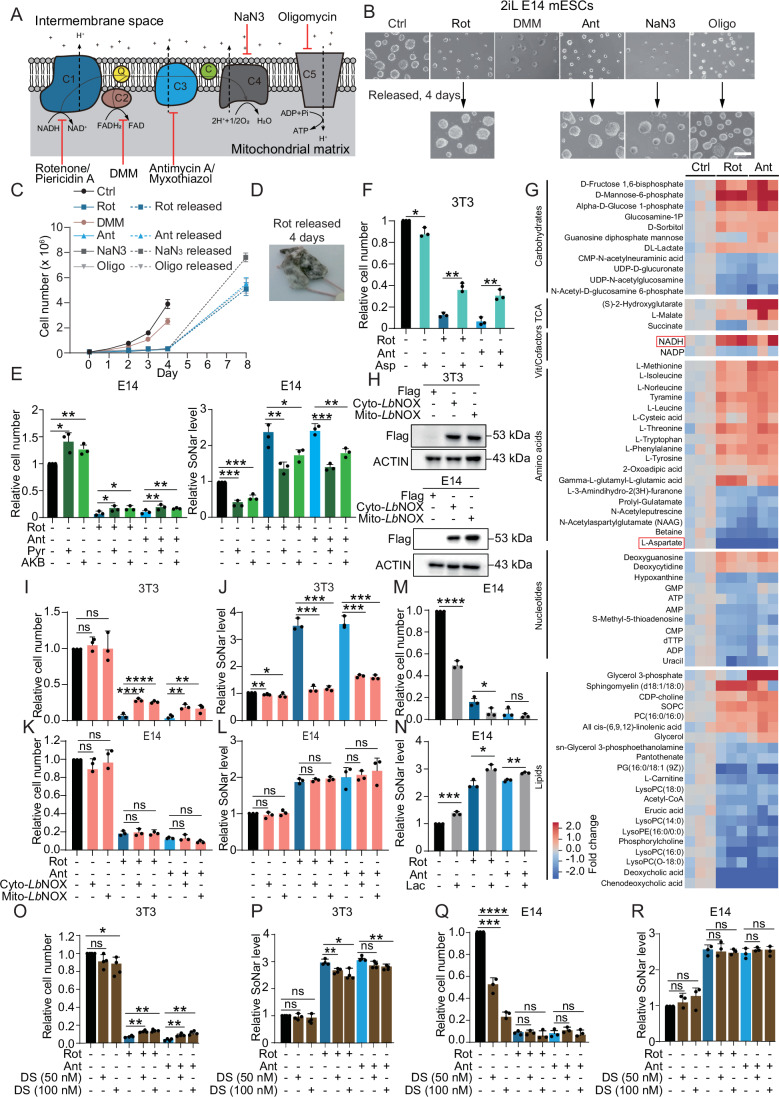
TDH sustains reductive stress and contributes to ETC C1 inhibition-induced pausing in mESCs, related to Fig. 1. (**A**) Schematic depiction of ETC complex (C1-5) and their functions with respective complex specific inhibitors. (**B**,** C**) Cell images (**B**) and cell number (**C**) of E14 mESCs treated with rotenone (Rot 150 nM), dimethyl malonate (DMM 15 µM), antimycin A (Ant 10 nM), sodium azide (NaN3 500 µM), and oligomycin A (Oligo 10 nM) for 4 days in 2iL and released for 4 days (mean ± SD, *n* = 3 each with biological replicates), scale bar, 200 µm. (**D**) Image of chimeric mice from Rot-induced paused mESCs for 4 days and released for 4 days. (**E**) Relative cell number and relative SoNar levels of E14 mESCs treated with Rot or Ant with and without pyruvate or AKB in pyruvate-free medium for 4 days (mean ± SD, *n* = 3 each with biological replicates). (**F**) Relative cell number of 3T3 treated with Rot (200 nM) or Ant (1 µM) with or without dimethyl-aspartate (Asp 10 mM) for 4 days in MEF medium (mean ± SD, *n* = 3 each with biological replicates). (**G**) Heat maps showing relative differential metabolites from E14 mESCs treated with Rot or Ant for 3 days. (**H**) Western blot analysis from 3T3 and E14 mESCs transduced with Flag, or cyto-*Lb*NOX or mito-*Lb*NOX. ACTIN was used as a loading control. (**I, J**) Relative cell number (**I**) and relative SoNar levels (**J**) from 3T3 cells transduced with Flag and cyto-*Lb*NOX or mito-*Lb*NOX treated with or without Rot or Ant for 4 days (mean ± SD, *n* = 3 each with biological replicates). (**K**,** L**) Relative cell number (**K**) and relative SoNar levels (**L**) from E14 cells transduced with Flag or cyto-*Lb*NOX or mito-*Lb*NOX treated with or without Rot or Ant for 4 days (mean ± SD, *n* = 3 each with biological replicates). (**M**,** N**) Relative cell number (**M**) and relative SoNar levels (**N**) from E14 mESCs treated with Rot or Ant and with and without lactate (10 mM) for 4 days (mean ± SD, *n* = 3 each with biological replicates). (**O**–**R**) Relative cell number (**O**) and relative SoNar levels of 3T3 (**P**), relative cell number (**Q**), and relative SoNar levels of E14 (**R**), treated with the indicated treatments for 4 days (mean ± SD, *n* = 3 each with biological replicates). Data information, in Fig. EV1E–H, K–R, columns represent mean, error bars are standard deviation, points are individual biological replicates; lines indicate comparisons between different groups; stars indicate statistical significance. *p < 0.05, **p < 0.01, ***p < 0.001, ****p < 0.0001; ns, not significant by unpaired two-tailed Student’s *t*-test. Individual *p* values are provided in Table EV1.

**Figure 1:**
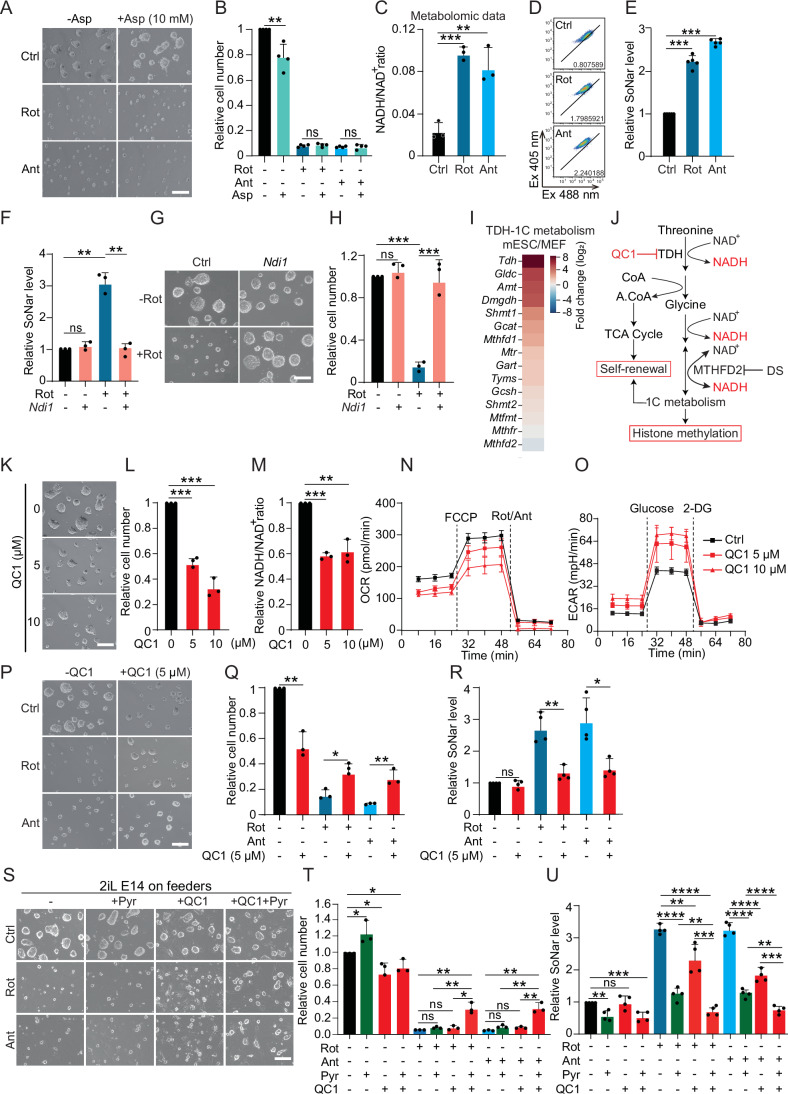
TDH sustains reductive stress and contributes to ETC C1 inhibition-induced pausing in mESCs. (**A**,** B**) Cell images (**A**) and relative cell number (**B**) of 2iL E14 mESCs treated with rotenone (Rot 150 nM) or antimycin A (Ant 10 nM) for 4 days with or without dimethyl-aspartate (Asp 10 mM) (*n* = 4 each with biological replicates), scale bar, 200 µm. (**C**) Intracellular NADH/NAD^+^ ratio of metabolomics from E14 mESCs treated with Rot or Ant for 3 days (mean ± SD, *n* = 3 each with biological replicates). (**D**,** E**) Cell cytometry plots of SoNar levels (**D**) and its quantification (**E**) from E14 mESCs treated with Rot or Ant for 4 days (mean ± SD, *n* = 4 each with biological replicates). (**F**) Relative SoNar levels from E14 mESCs transduced with Flag or *Ndi1* with or without Rot for 4 days (mean ± SD, *n* = 3 each with biological replicates). (**G**,** H**) Cell images (**G**) and relative cell number (**H**) of E14 mESCs transduced with Flag or *Ndi1* with or without Rot for 4 days (mean ± SD, *n* = 3 each with biological replicates), scale bar, 200 µm. (**I**) Heatmap showing the log_2_ fold change of expression levels of selected enzymes related to *Tdh*, glycine cleavage, and 1 C metabolic pathway in 2iL mESCs compared to MEFs. (**J**) Schematic depiction of TDH, glycine cleavage, and 1C metabolic pathways that produce NADH. (**K**,** L**) Cell images (**K**) and relative cell number (**L**) of E14 mESCs treated with the indicated doses of QC1 for 4 days (mean ± SD, *n* = 3 each with biological replicates), scale bar, 200 µm. (**M**) Relative total NADH/NAD^+^ ratio of E14 mESCs treated with the indicated doses of QC1 in 2iL for 4 days (mean ± SD, *n* = 3 each with biological replicates). (**N**) Oxygen consumption rate (OCR) analysis of E14 mESCs treated with the indicated doses of QC1 in 2iL for 4 days (Mean ± SD, *n* = *1* biological replicate with four technical replicates are shown). (**O**) Extracellular acidification rate (ECAR) analysis of E14 mESCs treated with the indicated doses of QC1 in 2iL for 4 days (Mean ± SD, *n* = *1* biological replicate with 5 technical replicates are shown). (**P**–**R**) Cell images (**P**), relative cell number (**Q**), and relative SoNar levels (**R**) of E14 mESCs treated with the rotenone (Rot 150 nM), and Antimycin A (Ant 10 nM), examined rescue by QC1 for 4 days (mean ± SD, *n* = 3 each with biological replicates), scale bar, 200 µm. (**S**–**U**) Cell images (**S**), relative cell number (**T**), and relative SoNar levels (**U**) of E14 mESCs treated with the indicated treatments and with or without pyruvate (Pyr 2 mM) for 4 days (mean ± SD, *n* = 3 each with biological replicates), scale bar, 200 µm. Data information, in Fig. 1B, C, E, F, H, L, M, Q, R, T, U, columns represent mean, error bars are standard deviation, points are individual biological replicates; lines indicate comparisons between different groups; stars indicate statistical significance. *p < 0.05, **p < 0.01, ***p < 0.001, ****p < 0.0001; ns not significant by unpaired two-tailed Student’s *t*-test. Individual *p* values are provided in Table EV1. See also Figs. EV1 and EV2. Source data are available online for this figure.

We initially confirmed that blocking ETC complexes 1 (with rotenone, Rot), 3 (with antimycin, Ant), 4 (with sodium azide, NaN_3_), and 5 (the ATP synthase, with oligomycin, Oligo)—but not complex 2 (with dimethyl malonate, DMM)—arrested the proliferation of ground-state E14 mESCs, as previously shown (Ying et al, 2008) (Fig. EV1A,B). Upon inhibitor withdrawal, the cells restored normal self-renewal levels and could contribute to chimeric mice following blastocyst injection and surrogate mother transplantation (Fig. EV1C,D). These findings validate that electron transport, primarily through complex1 (C1), is essential for mESC self-renewal, and its inhibition induces a paused pluripotent state. Considering pyruvate is normally included in the N2B27 2iL medium at 1.5 mM, we tested the role of pyruvate during ETC inhibition. Adding 2 mM of either pyruvate or the electron acceptor α-ketobutyrate (AKB) produced only a modest rescue of the pausing phenotype and reduced cytosolic reductive stress to some extent, but neither approach restored cells to normal levels (Fig. EV1E). These results indicate that while electron acceptors are functional in this context, their capacity to relieve the phenotype is very limited. Moreover, adding aspartate, the side-product of TCA and a vital component for nucleotide biosynthesis and cell proliferation in many other cells, including NIH3T3 (3T3), also failed to rescue the pausing phenotype in E14 mESCs. (Figs. 1A,B and EV1F). To identify the reason for these failures, we analyzed the metabolomic profiles of E14 cells paused by ETC inhibition. As expected, Rot and Ant, the ETC C1 and C3 inhibitors, respectively, induce highly similar changes in the metabolomic profile, with only a few exceptions, likely due to the SDH inhibition caused by Ant (e.g., succinate, glycerol 3-phosphate, and (S)-2-hydroxyglutarate) (Fig. EV1G). Notably, aspartate dropped sharply, NADH increased, and NAD^+^ remained unchanged, leading to reductive stress (an imbalanced NADH/NAD^+^ ratio) (Figs. 1C and EV1G). Due to redox shuttling between mitochondria and the cytosol, reductive stress was also induced in the cytosol, as measured by the fluorescent sensor SoNar (Zhao et al, 2015) (Fig. 1D,E). To test whether reductive stress mediates the pausing phenotype, we expressed both mitochondrial and cytosolic forms of *Lb*NOX (mito-*Lb*NOX and cyto-*Lb*NOX) in 3T3 and E14 cells and confirmed their expression (Titov et al, 2016) (Fig. EV1H). In 3T3 cells, both forms partially rescued ETC inhibitor-induced proliferation defects (~2–4-fold) and effectively normalized SoNar signals (Fig. EV1I,J). In contrast, neither form rescued proliferation nor corrected SoNar signals in E14 cells (Fig. EV1K,L). Notably, the failure of *Lb*NOX to elicit a rescue effect in mESCs may be due to limited enzyme activity, impaired compartmental access, or an inability to reach the relevant NADH pool, rather than reflecting the absence of reductive stress itself. By comparison, NDI1—the yeast complex I homolog that is insensitive to rotenone—fully restored redox balance and self-renewal in rotenone-treated mESCs (Fig. 1F–H). Furthermore, adding lactate induces reductive stress and inhibits self-renewal in E14 cells treated with or without ETC inhibitors (Fig. EV1M,N). Together, these findings indicate that excessive NADH/NAD^+^ disequilibrium is a key determinant of the paused phenotype.

**Figure EV2:**
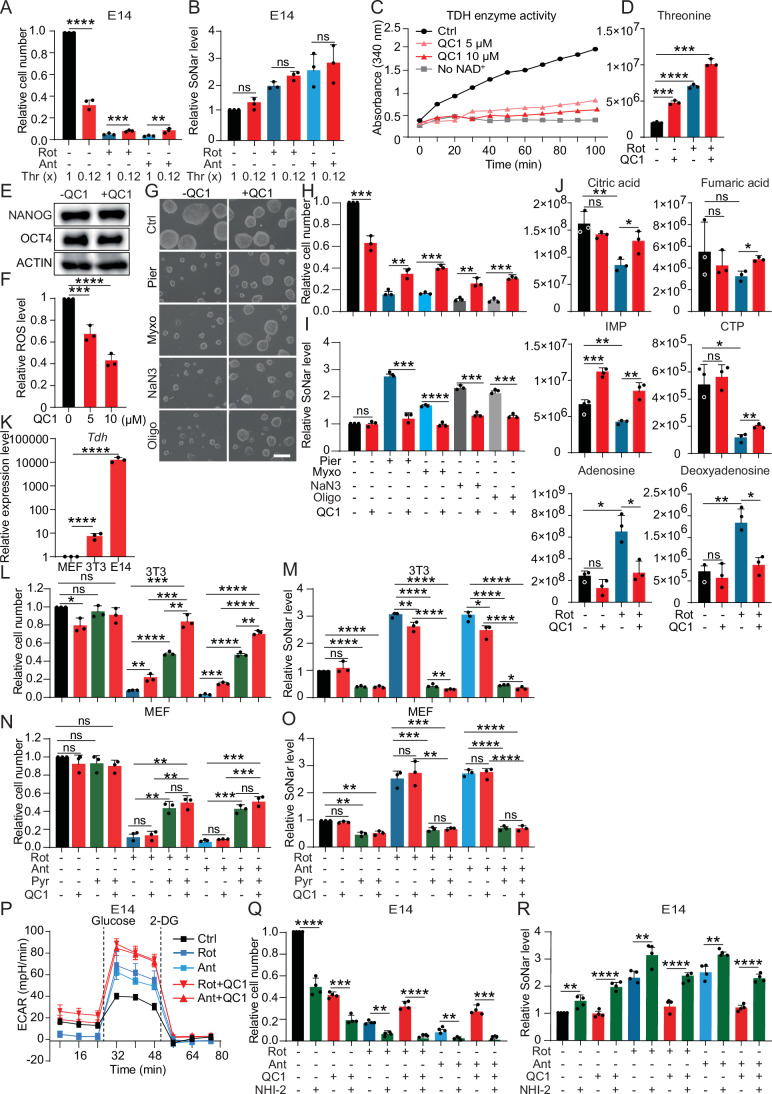
TDH sustains reductive stress and contributes to ETC C1 inhibition-induced pausing in mESCs, related to Fig. 1. (**A**,** B**) Relative cell number (**A**) and relative SoNar levels (**B**) of E14 mESCs with Rot and Ant with indicated doses of threonine for 4 days in threonine-free 2iL media (mean ± SD, *n* = 3 each with biological replicates). (**C**) TDH enzyme activity assay for E14 mESCs treated with different doses of QC1. Enzyme activity was determined by absorbance at 340 nm over time to monitor the conversion of NAD^+^ to NADH, and no NAD^+^ acts as a negative control. (**D**) Intracellular threonine levels of metabolomics from E14 mESCs treated with Rot either alone or in combination with QC1 5 µM for 3 days (mean ± SD, *n* = 3 each with biological replicates). (**E**) Western blotting for OCT4 and NANOG in E14 mESCs treated with QC1 5 µM for 4 days. ACTIN was used as the loading control. (**F**) Relative reactive oxygen species (ROS) of E14 mESCs with the indicated doses of QC1 for 4 days (mean ± SD, *n* = 3 each with biological replicates). (**G**–**I**) Cell images (**G**), relative cell number (**H**), and relative SoNar levels (**I**) from E14 mESCs treated with Piericidin (Pier 2 µM) for C1^,^ Myxothiazol (Myxo 5 nM) for C3, sodium azide (NaN3 500 µM) for C4, and oligomycin A (Oligo 10 nM) for C5 and examined rescue by QC1 for 4 days (mean ± SD, *n* = 3 each with biological replicates), scale bar, 200 µm. (**J**) Intracellular metabolites citrate, fumarate, IMP, CTP, adenosine, and deoxyadenosine of metabolomics from E14 mESCs treated with Rot either alone or in combination with QC1 for 3 days (mean ± SD, *n* = 3 each with biological replicates). (**K**) Relative expression level of *Tdh* in somatic cells (MEFs and 3T3) and E14 mESCs. (**L**–**O**) Relative cell number (**L**), relative SoNar levels of 3T3 (**M**), relative cell number (**N**), and relative SoNar levels of MEF (**O**), treated with the indicated treatments for 4 days (mean ± SD, *n* = 3 each with biological replicates). (**P**) ECAR analysis of E14 mESCs treated with the indicated treatments for 4 days (Mean ± SD *n* = 1 biological replicate with four technical replicates are shown). (**Q**,** R**) Relative cell number (**Q**) and relative SoNar levels (**R**) of E14 mESCs treated with the indicated treatments for 4 days (mean ± SD, *n* = 3 each with biological replicates). Data information, in Fig. EV2A, D, F, H–O, Q, R, columns represent mean, error bars are standard deviation, points are individual biological replicates; lines indicate comparisons between different groups; stars indicate statistical significance. *p < 0.05, **p < 0.01, ***p < 0.001, ****p < 0.0001; ns, not significant by unpaired two-tailed Student’s *t*-test. Individual *p* values are provided in Table EV1.

We reasoned that some NAD^+^-dependent dehydrogenase(s) in mESCs remain active during ETC inhibition and continue to generate NADH despite reductive stress. We first examined MTHFD2, a 1C pathway dehydrogenase previously shown to sustain NADH production during complex I inhibition (Yang et al, 2020a). Inhibition of MTHFD2 with the specific inhibitor DS18561882 (Kawai et al, 2019) alleviated ETCi-induced defects in proliferation and redox balance in 3T3 cells but not in E14 cells (Fig. EV1O–R), suggesting that additional or stronger NADH-producing pathways operate in mESCs. Consistently, comparison of published RNA-seq datasets (Huang et al, 2020) revealed that 2iL E14 cells express markedly higher levels of genes involved in the threonine dehydrogenase (TDH) pathway, the glycine cleavage system, and the broader 1C metabolism (Fig. 1I,J). Beyond generating acetyl-CoA and glycine, TDH simultaneously produces substantial amounts of NADH (Shyh-Chang et al, 2013; Wang et al, 2009), yet its reductive stress contribution has remained unexplored. To test this, we first lowered threonine concentrations during ETC inhibition. Threonine reduction moderately rescued the paused phenotype, although cytosolic redox showed only a non-significant upward trend (Fig. EV2A,B), consistent with partial but incomplete suppression of TDH flux. To directly inhibit TDH, we employed the specific small-molecule inhibitor QC1 (Alexander et al, 2011) and established an effective dose range of 5–10 μM in 2iL E14 cells (Fig. EV2C). Metabolomic analysis confirmed threonine accumulation upon QC1 treatment in both basal and ETC-inhibited conditions (Fig. EV2D), indicating effective TDH inhibition. QC1 reduced self-renewal capacity without altering colony morphology or pluripotency gene expression (Figs. 1K,L and EV2E), accompanied by a decrease in total NADH/NAD⁺ ratio, reduced OCR and ROS, and increased glycolysis—features consistent with a compensatory shift toward glycolytic ATP production when mitochondrial NADH oxidation is limited (Figs. 1M–O and EV2F).

Importantly, in ETC-paused E14 cells cultured with pyruvate, QC1 treatment restored both self-renewal and redox balance and reversed alterations in TCA-related metabolites (Figs. 1P–R and EV2G–J). In the absence of pyruvate, however, 2iL mESCs co-treated with ETC and TDH inhibitors survived only on feeder layers; under these conditions, TDH inhibition enabled pyruvate to reverse reductive stress and significantly rescue self-renewal (Fig. 1S–U). Consistent with endogenous *Tdh* expression levels, QC1 also rescued ETCi-induced defects in 3T3 cells—though to a lesser extent than pyruvate—but had no effect in MEFs, which lack *Tdh* expression (Fig. EV2K–O). By inducing glycolytic activation (Fig. EV2P–R), TDH inhibition helped maintain redox balance and proliferation. This finding reveals that an integrated redox network coordinates metabolic adaptation to ETC blockade.

Taken together, these findings indicate that TDH inhibition relieves the excessive NADH burden imposed by ETC blockade, while pyruvate provides the necessary cytosolic NAD^+^ recycling to support proliferation. This raises an important question: is such reductive stress merely a metabolic by-product of supporting biosynthesis and self-renewal, or does it serve additional functions in pluripotent cells?

### TDH-driven reductive stress during ETC inhibition favors cells with intact mitochondrial function in mESCs

**Figure 2:**
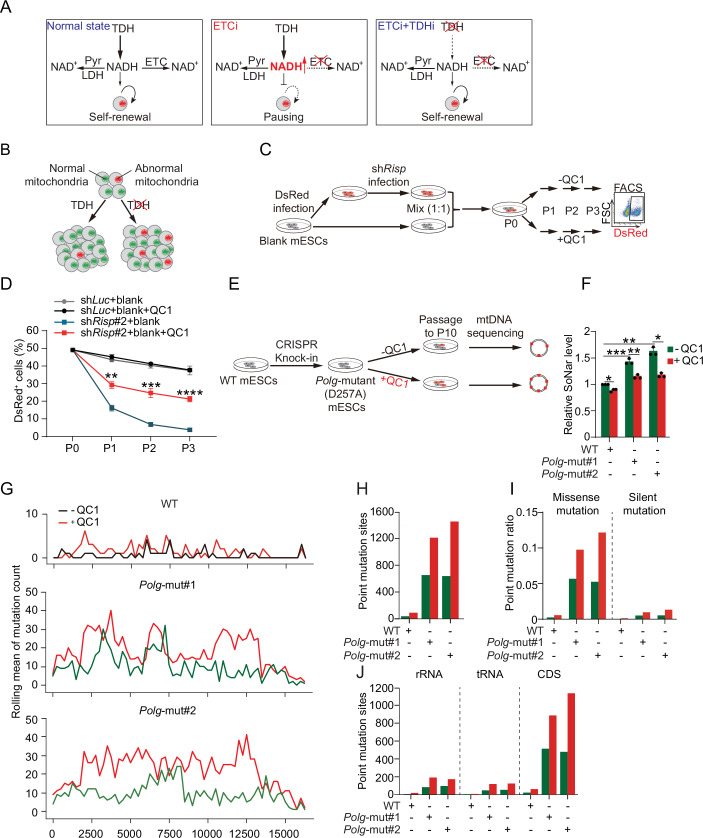
TDH-driven reductive stress during ETC inhibition creates selection pressure against cells with mitochondrial impairment in mESCs. (**A**) Schematic summary of Fig. 1 on how TDH inhibition partially rescues the ETCi-induced paused proliferation in naive E14 mESCs. (**B**) Schematic depiction of ETC-impaired cells in normal or TDH-blocked conditions, highlighting that ETC-impaired cells are enriched more in TDH-blocked conditions. (**C**) Schematic depiction of cell competition between wild-type and ETC-impaired E14 mESCs. The percentage of DsRed^+^ cells was measured with flow cytometry for each passage from passages 1 to 3. (**D**) Curves showing the percentage of DsRed^+^ E14 mESCs transduced with sh*Luc* or sh*Risp* in co-culture with sh*Luc* blank cells from passages 1–3 with or without QC1 (5 µM) (*n* = 4 each with biological replicates, points represent mean, error bars are standard deviation; stars indicate statistical significance of sh*Risp* with and without QC1 from P1-3. (**p < 0.01, ***p < 0.001, ****p < 0.0001, by unpaired two-tailed Student’s t-test. Individual *p* values are provided in Table EV1). (**E**) Schematic depiction of the construction of *Polg* mutant knock-in cell lines and mtDNA samples preparation for next-generation sequencing. (**F**) Relative SoNar levels in WT and the two *Polg* mutant cell lines with or without QC1 (5 µM) from passage 7 (mean ± SD, *n* = 3 each with biological replicates, columns represent mean, error bars are standard deviation, points are individual biological replicates; lines indicate comparisons between different groups; stars indicate statistical significance. (*p < 0.05, **p < 0.01, ***p < 0.001, by unpaired two-tailed Student’s *t*-test. Individual *p* values are provided in Table EV1). (**G**) Lines showing the rolling mean (250 bp) of mutation number in WT and the two *Polg* mutant cell lines (middle and lower panels) from passage 10 with or without QC1. (**H**) Number of total mutated positions in WT and the two *Polg* mutant cell lines from passage 10 with or without QC1. (**I**) Numbers of positions for missense and silent mutations of WT and the two *Polg* mutant cell lines from passage 10 with or without QC1. (**J**) Number of mutated positions for rRNA, tRNA, and CDS in the mtDNA of WT and the two *Polg* mutant cell lines from passage 10 with or without QC1. Figure EV3. Source data are available online for this figure.

**Figure EV3:**
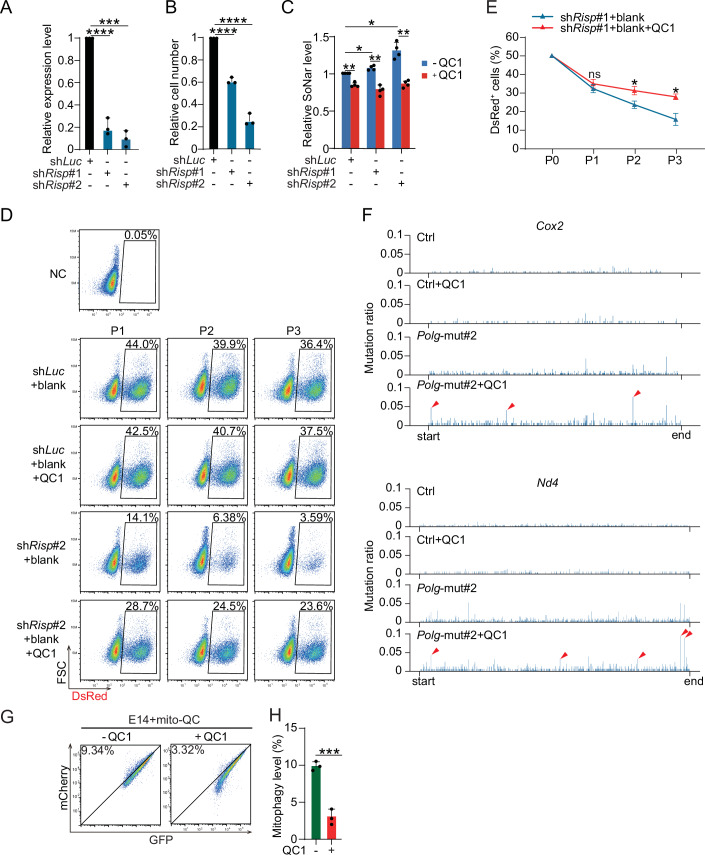
TDH-driven reductive stress during ETC inhibition creates selection pressure against cells with mitochondrial impairment in mESCs, related to Fig. 2. (**A**) RT-qPCR analysis showing the knockdown efficiency of sh*Risp* relative to sh*Luc* in E14 mESCs (mean ± SD, *n* = 3 each with biological replicates). (**B**) Relative cell number of E14 mESCs transduced with sh*Luc*, or sh*Risp* from passage 1 (mean ± SD, *n* = 3 each with biological replicates). (**C**) Relative SoNar levels of E14 mESCs transduced with sh*Luc*, or sh*Risp* with or without QC1 (5 µM) from passage 1 (mean ± SD, *n* = 4 each with biological replicates). (**D**) Represented flow cytometry analysis of DsRed^+^ E14 mESCs in the indicated conditions. (**E**) Curves showing the percentage of DsRed^+^ E14 mESCs transduced with sh*Luc* or sh*Risp* in co-culture with sh*Luc* blank E14 mESCs from passages 1 to 3 with or without QC1 (5 µM) (*n* = 4 each with biological replicates, points represent mean, error bars are standard deviation; stars indicate statistical significance of sh*Risp* with and without QC1 from P1 to 3. (**p < 0.01, ***p < 0.001, ****p < 0.0001, by unpaired two-tailed Student’s *t*-test. Individual *p* values are provided in Table EV1). (**F**) The mutation ratio of two representative mitochondrial genes from the indicated conditions at passage 10. (**G**,** H**) Cell cytometry plots of mito-QC reporter (**G**), and its quantification (**H**) from E14 mESCs treated with or without QC1 (5 µM) from passage 10 (mean ± SD, *n* = 3 each with biological replicates). Columns represent mean, error bars are standard deviation, points are individual biological replicates; lines indicate comparisons between different groups; stars indicate statistical significance. Data information, in Fig. EV3A–C, H, columns represent mean, error bars are standard deviation, points are individual biological replicates; lines indicate comparisons between different groups; stars indicate statistical significance. *p < 0.05, **p < 0.01, ***p < 0.001, ****p < 0.0001; by unpaired two-tailed Student’s *t*-test. Individual *p* values are provided in Table EV1.

Based on the above results that ETC blockade-induced pausing can be partially rescued by TDH inhibition (Fig. 2A), we proposed that in any mESCs with mitochondrial defects, TDH-driven reductive stress reduces the proliferative capacity of cells with mitochondrial impairment and thereby creates a population-level selection pressure against such cells (Fig. 2B). To investigate this possibility, we artificially introduced mitochondrial abnormality (achieved by knocking down *Risp* and labeling with DsRed) in mESCs and mixed them with an equal number of normal cells. Then, we monitored the percentage of the DsRed^+^ (i.e., ETC-impaired) cells using flow cytometry across several passages with or without TDH inhibition (Fig. 2C). When cultured alone, ETC-deficient cells exhibited reduced self-renewal and increased reductive stress, which was efficiently eliminated by TDH inhibition with 5 μM QC1 (Fig. EV3A–C). In the mixed condition, the proportion of ETC-deficient cells decreased rapidly over three passages in untreated conditions. However, this decline was significantly slowed by inhibiting TDH (Figs. 2D and EV3D,E), consistent with the idea that TDH-driven reductive stress accelerates the competitive loss of cells with mitochondrial defects.

Given the critical role of mitochondrial DNA (mtDNA) in mitochondrial function and its relatively faster mutation rate than nuclear DNA, coupled with fewer repair mechanisms (Kong et al, 2022), we further explored whether the TDH pathway could mitigate mtDNA mutations in mESCs. Because the natural mutation rate for mitochondrial DNA is relatively low, to prove the idea in principle, we introduced a *Polg*-D257A mutation (Trifunovic et al, 2004) to increase the basal mutation rate. We cultured wild-type (WT) and two D257A mutant cell lines with or without TDH inhibition using QC1 for over ten passages and sequenced their mtDNA mutations (Fig. 2E). SoNar measurement confirmed the induction of reductive stress in the mutant cells, significantly ameliorated by QC1 treatment (Fig. 2F). After ten passages, mtDNA sequencing with an average depth of ~500x coverage of the entire mitochondrial genome showed that while QC1 treatment increased mutation in WT cells only slightly, it did so much more strikingly in the two mutant cell lines (Fig. 2G,H). We identified up to 10% missense mutations and less than 1% nonsense mutations in the *Polg* mutant cells treated with QC1 (Fig. 2I). Moreover, there was a notable enrichment of mutations in the protein-coding regions, which comprise the major part of the mitochondrial genome (Fig. 2J). The detailed mutation profiles in two representative genes, *Cyclooxygenase 2* (*Cox2*) and *NADH dehydrogenase 4* (*Nd4*), are shown (Fig. EV3F). Mitochondrial quality in mESCs has been reported to be regulated in part by mitochondrial autophagy (mitophagy) (Liu et al, 2016). To assess whether mitophagy is affected in QC1-treated mESCs, we used the established fluorescent reporter mito-QC (McWilliams et al, 2016). Strikingly, TDH inhibition markedly reduced mitophagy levels in mESCs (Fig. EV3G,H). These results suggest that TDH-dependent metabolism contributes to basal mitophagy activity and may influence the retention or elimination of mitochondria with impaired function.

In conclusion, our findings indicate that the reductive stress-insensitive NADH-producing activity of TDH imposes a metabolic constraint that selectively disadvantages cells with compromised mitochondrial function. Through this passive selection process, TDH-driven reductive stress limits the persistence of cells with mitochondrial defects and thereby biases the population toward metabolically competent mESCs.

### TDH-driven intrinsic reductive stress suppresses PDH activation and is associated with genomic and epigenetic stability in mESCs

**Figure 3:**
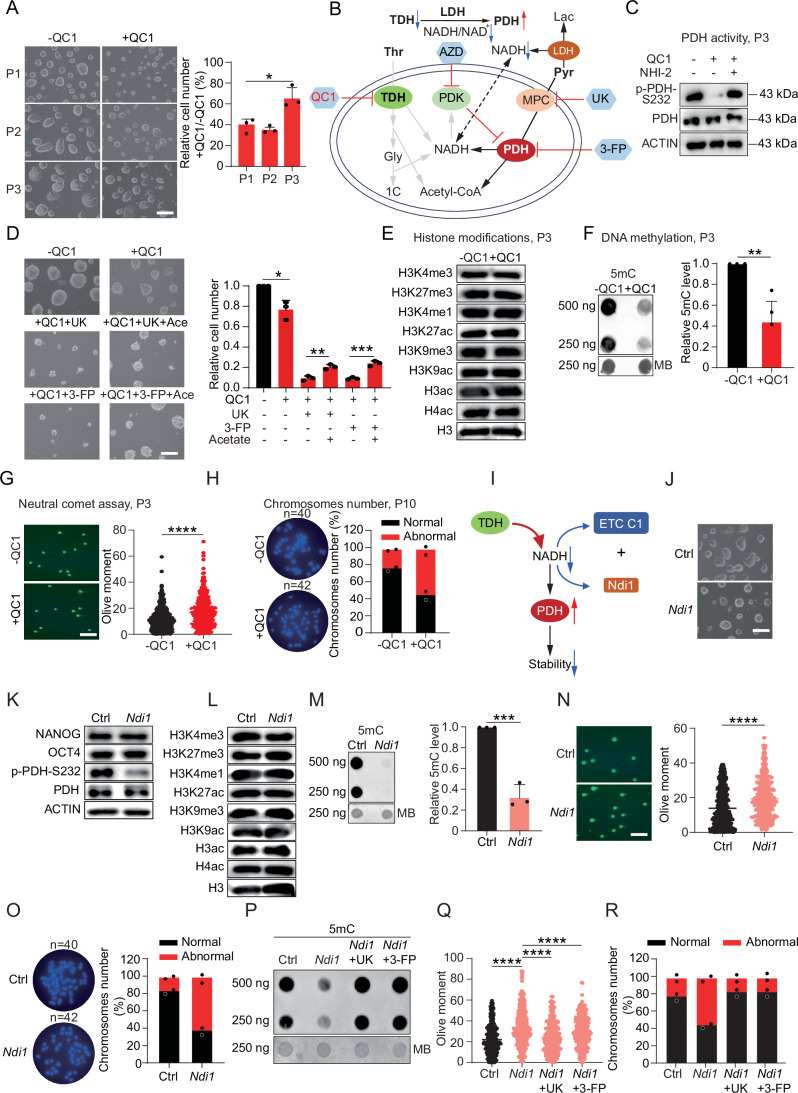
TDH-driven intrinsic reductive stress suppresses PDH activity and correlates with reduced genomic and epigenetic abnormalities in mESCs. (**A**) Cell images and relative cell number +QC1/-QC1 (%) of 2iL E14 mESCs treated with QC1 (10 µM) for 1–3 passages (mean ± SD, *n* = 3 each with biological replicates), scale bar, 200 µm. (**B**) Schematic depiction of the TDH-to-PDH adaptation for cell survival. (**C**) Western blotting for PDH and p-PDH-S232 of E14 mESCs treated with and without QC1 and QC1 + NHI-2 for 3 passages. ACTIN was used as the loading control. (**D**) Cell images and relative cell number of E14 mESCs treated with QC1 for 3 passages and with or without UK5099 (15 µM) or 3-FP (700 nM) for 2 passages and acetate (10 mM) for 1 passage (mean ± SD, *n* = 3 each with biological replicates), scale bar, 200 µm. (**E**) Western blotting for histone marks with the indicated antibodies of E14 mESCs treated with QC1 for three passages. Histone 3 (H3) was used as the loading control. (**F**) Images and relative quantification of the DNA dot blot of E14 mESCs treated with QC1 for three passages. Methylene blue (MB) was used as the loading control (mean ± SD, *n* = 3 each with biological replicates). (**G**) Representative images and quantification (olive moment) of neutral comet assay of E14 mESCs treated with QC1 for three passages (mean ± SD, *n* = 3 each with biological replicates), scale bar, 200 µm. (**H**) Representative images and quantification (percentage of 41–43 chromosomes) of chromosomes of E14 mESCs treated with QC1 for 10 passages (mean ± SD, *n* = 2 each with biological replicates), percentages of euploid (black, 40 chromosomes) and hyperploid (red, 41–43 chromosomes) mESCs are represented as bar graphs, scale bar, 40 µm. (**I**) Schematic depiction of *Ndi1* recycling the NAD^+^ to activate the PDH and impair the genomic stability. (**J**) Cell images of E14 mESCs transduced with Flag or *Ndi1* from passage 3, scale bar, 200 µm. (**K**) Western blotting for OCT4, NANOG, PDH and p-PDH-S232 of E14 mESCs transduced with Flag or *Ndi1* for three passages. ACTIN was used as the loading control. (**L**) Western blotting for histone marks with indicated antibodies of E14 mESCs transduced with Flag or *Ndi1* for three passages. H3 was used as the loading control. (**M**) Images and relative quantification of DNA dot blot of E14 mESCs transduced with Flag or *Ndi1* for 3 passages. Methylene blue was used as the loading control (mean ± SD, *n* = 3 each with biological replicates). (**N**) Representative images and quantification (olive moment) of neutral comet assay of E14 mESCs transduced with Flag or *Ndi1* for 3 passages (mean ± SD, *n* = 3 each with biological replicates), scale bar, 200 µm. (**O**) Representative images and quantification (percentage of 41–43 chromosomes) of chromosomes in E14 mESCs transduced with Flag or *Ndi1* for 10 passages (mean ± SD, *n* = 2 each with biological replicates), scale bar, 40 µm. (**P**) Images of DNA dot blot of E14 mESCs transduced with Flag or *Ndi1* with or without UK5099 (15 µM) or 3-FP (700 nM) for three passages. Methylene blue was used as the loading control. (**Q**) Neutral comet assay quantification of E14 mESCs transduced with Flag or *Ndi1* with or without UK5099 or 3-FP for 3 passages (mean ± SD, *n* = 3 each with biological replicates), scale bar, 200 µm. (**R**) Chromosome quantification of E14 mESCs transduced with Flag or *Ndi1* with or without UK5099 or 3-FP for ten passages (mean ± SD, *n* = 2 each with biological replicates). Data information, in Fig. 3A, D, F, M, columns represent mean, points are individual biological replicates; and in **G**,** N**, **Q**, violin plots represent mean, points are individual cell DNA; error bars are standard deviation, lines indicate comparisons between different groups; stars indicate statistical significance. *p < 0.05, **p < 0.01, ***p < 0.001, ****p < 0.0001; ns not significant by unpaired two-tailed Student’s *t*-test. Individual *p* values are provided in Table EV1. See also Fig. EV4. Source data are available online for this figure.

**Figure EV4:**
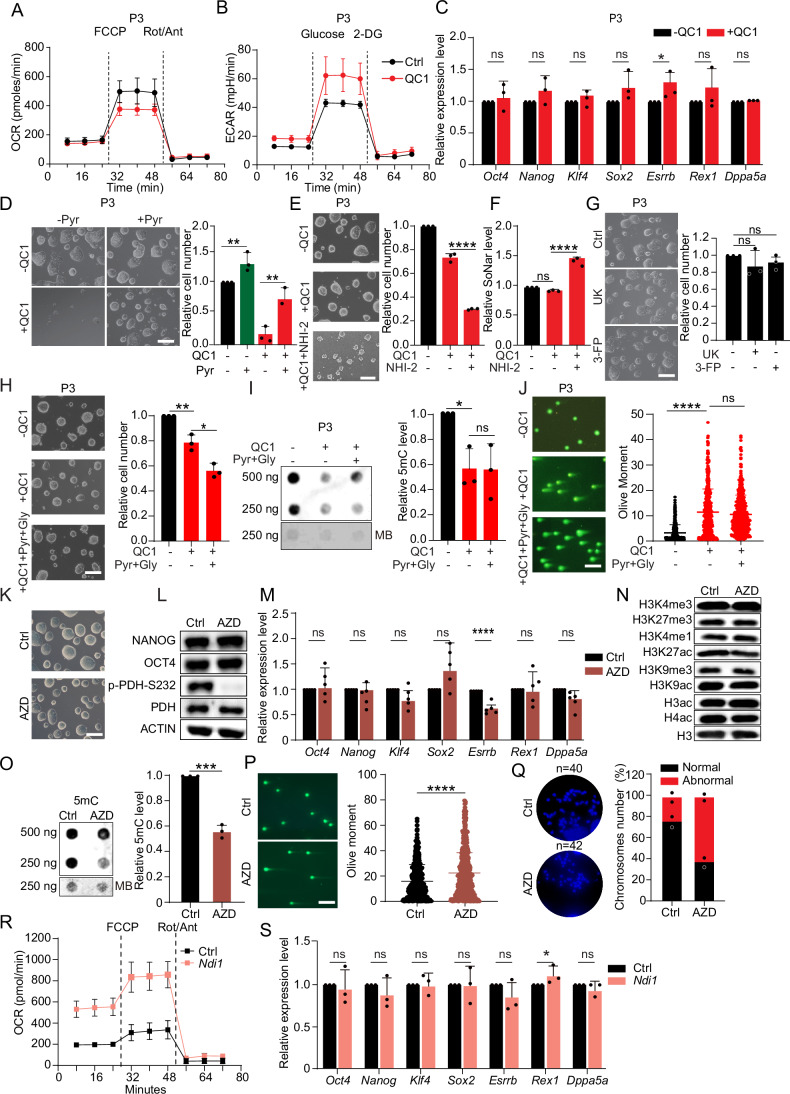
TDH-driven intrinsic reductive stress suppresses PDH activity and correlates with reduced genomic and epigenetic abnormalities in mESCs, related to Fig. 3. (**A**) OCR analysis of E14 mESCs treated with and without QC1 (10 µM) in 2iL for three passages (Mean ± SD, *n* = 1, 5 technical replicates are shown). (**B**) ECAR analysis of E14 mESCs treated with and without QC1 (10 µM) in 2iL for three passages (Mean ± SD *n* = 1, 4 technical replicates are shown). (**C**) Relative expression levels of pluripotent markers in E14 mESCs treated with QC1 (10 µM) for three passages (mean ± SD, *n* = 3 each with biological replicates). (**D**) Cell images and relative cell number of E14 mESCs treated with or without QC1 in combination with or without pyruvate (2 mM) for 3 passages (mean ± SD, *n* = 3 each with biological replicates). (**E**,** F**) Cell images and relative cell number (**E**) and relative SoNar levels (**F**) of E14 mESCs treated with QC1, either alone or in combination with NHI-2 (10 µM), for three passages (mean ± SD, *n* = 3 each with biological replicates). (**G**) Cell images and relative cell number of E14 mESCs treated with and without UK5099 (15 µM) or 3-FP (700 nM) for three passages (mean ± SD, *n* = 3 each with biological replicates). (**H**) Cell images and relative cell number of E14 mESCs treated with and without QC1 in the absence or presence of a combination of pyruvate (10 mM) and glycine (6 mM), for ten passages (mean ± SD, *n* = 3 each with biological replicates). (**I**) Images and relative quantification of DNA dot blot of E14 mESCs treated with and without QC1, in the absence or presence of a combination of pyruvate and glycine for ten passages. Methylene blue was used as the loading control (mean ± SD, *n* = 3 each with biological replicates). (**J**) Representative images and quantification (olive moment) of neutral comet assay of E14 mESCs treated with and without QC1, in the absence or presence of a combination of pyruvate and glycine for ten passages (mean ± SD, *n* = 3 each with biological replicates), scale bar, 200 µm. (**K**) Cell images of E14 mESCs treated with AZD7545 (3 µM) for three passages, scale bar, 200 µm. (**L**) Western blotting for OCT4, NANOG, PDH, and p-PDH-S232 in E14 mESCs treated with AZD7545 for three passages. ACTIN was used as the loading control. (**M**) Relative expression levels of pluripotent markers in E14 mESCs treated with AZD7545 for three passages (mean ± SD, *n* = 5 each with biological replicates). (**N**) Western blotting for histone marks with indicated antibodies in E14 mESCs treated with AZD7545 for three passages. H3 was used as the loading control. (**O**) Images and relative quantification of the DNA dot blot of E14 mESCs treated with AZD7545 for three passages. Methylene blue was used as the loading control (mean ± SD, *n* = 3 each with biological replicates). (**P**) Representative images and quantification (olive moment) of neutral comet assay of E14 mESCs treated with AZD7545 for three passages (mean ± SD, *n* = 3 each with biological replicates), scale bar, 200 µm. (**Q**) Representative images and quantification (percentage of 41–43 chromosomes) of chromosomes of E14 mESCs treated with AZD7545 for ten passages (*n* = 2 each with biological replicates), scale bar, 40 µm. (**R**) OCR analysis of E14 mESCs transduced with Flag or *Ndi1* for three passages (Mean ± SD, *n* = 1, 5 technical replicates are shown). (**S**) Relative expression levels of pluripotent markers of E14 mESCs transduced with Flag or *Ndi1* for 3 passages (mean ± SD, *n* = 3 each with biological replicates). Data information, in Fig. EV4C**–**I, M, O, S; columns represent mean, points are individual biological replicates; and in (**J** and **P**), violin plots represent mean, points are individual cell DNA; error bars are standard deviation, lines indicate comparisons between different groups; stars indicate statistical significance. *p < 0.05, **p < 0.01, ***p < 0.001, ****p < 0.0001; ns, not significant by unpaired two-tailed Student’s *t*-test. Individual *p* values are provided in Table EV1.

The experiments in Fig. 2 used a lower dose of QC1 (5 μM) to permit moderate self-renewal in mESCs. A higher dose (10 μM) of QC1 markedly impaired self-renewal, likely due to insufficient acetyl-CoA production (Fig. 1K,L; Wang et al, 2009). Interestingly, when high-dose QC1 treatment was extended for more than two passages, E14 cells recovered >60% of their normal self-renewal capacity while maintaining reduced OCR and elevated glycolysis, without changes in pluripotency gene expression (Figs. 3A and EV4A–C). These observations suggest that an alternative source of acetyl-CoA becomes activated during prolonged TDH inhibition.

Because pyruvate metabolism through PDH is a major route for acetyl-CoA synthesis in mammalian cells (Guertin and Wellen, 2023), we first tested whether pyruvate becomes essential for long-term QC1-treated cells. As expected, these cells now depended on pyruvate not only for proliferation but also, to some extent, for survival (Fig. EV4D). Previous studies have shown that PDH activity is suppressed by NADH reductive stress, and our results demonstrate that TDH inhibition lowers the NADH/NAD^+^ ratio through lactate dehydrogenase (LDH) (Yang et al, 2020a; Titov et al, 2016) (Fig. EV2R). We therefore hypothesized that upon TDH inhibition, enhanced LDH activity decreases NADH/NAD^+^ levels and reactivates PDH to supply acetyl-CoA, thereby supporting self-renewal (Fig. 3B). To test this model, we inhibited LDH in long-term QC1-treated E14 cells and confirmed that LDH was required to sustain redox balance and self-renewal (Fig. EV4E,F).

We next examined PDH activity by assessing phosphorylation of serine 232 (S232), a site specifically inhibited by pyruvate dehydrogenase kinases (PDKs). As expected, phosphorylation at S232 was markedly reduced in QC1-treated cells, indicating PDH activation, and returned to control levels when LDH was inhibited, supporting a TDH-to-PDH metabolic adaptation (Fig. 3C). Consistent with this, blocking mitochondrial pyruvate import with UK5099 or inhibiting PDH with 3-fluoropyruvate (3-FP) significantly reduced survival and self-renewal in long-term QC1-treated mESCs (hereafter referred to as PDH-dependent mESCs), but not in QC1-untreated controls (Figs. 3D and EV4G). Importantly, acetate supplementation significantly rescued these phenotypes, confirming that the TDH-to-PDH adaptation is critical for maintaining acetyl-CoA levels and supporting survival and self-renewal under sustained TDH inhibition.

Next, we sought to understand the consequences of this TDH-to-PDH adaptation. Although PDH activation has been reported to impair pluripotency maintenance or re-establishment (Folmes et al, 2011; Rodrigues et al, 2015), the PDH-dependent mESCs retained normal morphology and pluripotency gene expression (Figs. 3A and EV4C). Beyond supplying acetyl-CoA, TDH also contributes to histone methylation and supports pluripotency through *S*-adenosylmethionine (SAM) production via the one-carbon (1C) pathway (Shyh-Chang et al, 2013). We therefore assessed whether these cells maintained an intact epigenetic landscape. Western blot analysis of multiple histone modifications (H3K4me3, H3K27me3, H3K4me1, H3K27ac, H3K9ac, and global H3/H4 acetylation, as well as H3K9me3) revealed no detectable differences (Fig. 3E). In contrast, global DNA methylation was markedly reduced in the PDH-dependent mESCs (Fig. 3F). DNA hypomethylation in PSCs is often associated with genomic instability (Choi et al, 2017; Di Stefano et al, 2018), and accordingly, these cells showed a significant increase in DNA double-strand breaks (Fig. 3G) and ~55% displayed abnormal chromosome numbers after extended culture (Fig. 3H). Importantly, supplementation with additional pyruvate or glycine failed to rescue these epigenetic and genomic abnormalities in the PDH-dependent mESCs (Fig. EV4H–J), indicating that the defects do not arise from insufficient PDH substrates or impaired SAM/1 C metabolism, but rather from the altered metabolic state caused by sustained PDH activation. Moreover, activating PDH alone with the PDK inhibitor AZD7545 in normal 2iL E14 cells induced similar DNA methylation loss and genomic instability without affecting pluripotency gene expression or histone modifications (Fig. EV4K–Q), demonstrating that PDH activation phenocopies the abnormalities observed under TDH inhibition.

Reductive stress could arise from insufficient NAD^+^ recycling through complex I. To test this possibility, we enhanced mitochondrial NAD^+^ regeneration and electron transfer by overexpressing *Ndi1* (Fig. 3I). As expected, *Ndi1* overexpression markedly increased OCR, consistent with enhanced NAD^+^ recycling (Fig. EV4R). Similar to the PDH-dependent mESCs, *Ndi1*-expressing E14 cells displayed PDH activation, maintained normal morphology and pluripotency gene expression, and exhibited no detectable alterations in histone modifications (Figs. 3J–L and EV4S). However, *Ndi1* overexpression led to pronounced DNA hypomethylation, increased double-strand breaks, and chromosome instability (Fig. 3M–O). Importantly, blocking either mitochondrial pyruvate import or PDH activity fully reversed these abnormalities (Fig. 3P–R), demonstrating that relief of reductive stress is sufficient to activate PDH and induce genomic and epigenetic instability in mESCs.

In summary, TDH-induced intrinsic reductive stress suppresses PDH activation and is associated with reduced accumulation of genomic and epigenetic abnormalities.

### ETC inhibition induces pausing in early embryonic development and human PSCs

**Figure 4:**
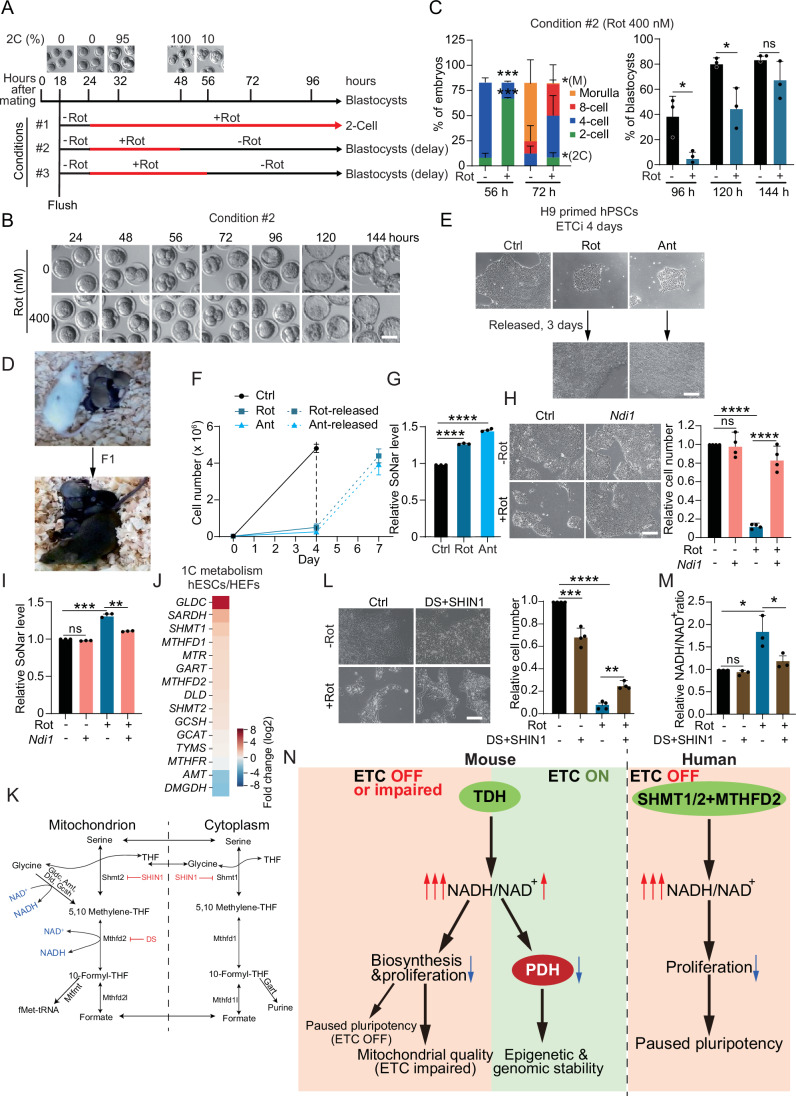
ETC inhibition induces pausing in early embryonic development and human PSCs. (**A**) Schematic depiction of different time windows of Rot treatment starting at the 1-cell embryo stage. (**B**,** C**) Images (**B**) and percentage (**C**) of developing embryos from control and condition #2 in (**A**) at the indicated time points (mean ± SD, *n* = 3 each with biological replicates), scale bar, 200 µm. (**D**) Images of live pups (F0, left image) born from Rot-treated cells for 24 h (24–48 h treatment) post-hCG 48 h and released for 1 h and F1 live mice born from F0 (right image). (**E**,** F**) Cell images (**E**) and cell number (**F**) of H9 primed hESCs treated with Rot (50 nM) or Ant (4 nM) for 4 days and released for 3 days in mTeSR (mean ± SD, *n* = 3 each with biological replicates), scale bar, 200 µm. (**G**) Relative SoNar levels of H9 primed hESCs treated with Rot or Ant for 4 days (mean ± SD, *n* = 3 each with biological replicates). (**H**,** I**) Cell images and relative cell number (**H**) and relative SoNar levels (**I**) of H9 primed hESCs transduced with Flag or *Ndi1* with or without Rot for 4 days (mean ± SD, *n* = 4 each with biological replicates), scale bar, 200 µm. (**J**) Heatmap showing the log_2_ fold change of expression levels of selected enzymes related to glycine cleavage, and 1C metabolic pathway in hESCs compared to HEFs. (**K**) Schematic depiction of glycine cleavage and 1C metabolic pathways that produce NADH. (**L**) Cell images and relative cell number of H9 primed hESCs treated with indicated treatment (mean ± SD, *n* = 4 each with biological replicates). (**M**) Relative intracellular NADH/NAD^+^ ratio of H9 primed hESCs treated with indicated treatment (mean ± SD, *n* = 3 each with biological replicates). (**N**) Schematic representation of TDH-driven NADH-reductive stress, mediating paused pluripotency and regulating mitochondrial quality, epigenetic modifications, and genomic stability across different ETC states. Data information, in Fig. 4C, G, H, I, L, M, columns represent mean, error bars are standard deviation, points are individual biological replicates; lines indicate comparisons between different groups; stars indicate statistical significance. *p < 0.05, **p < 0.01, ***p < 0.001, ****p < 0.0001; ns not significant by unpaired two-tailed Student’s *t*-test. Individual *p* values are provided in Table EV1. See also Fig. EV5. Source data are available online for this figure.

**Figure EV5:**
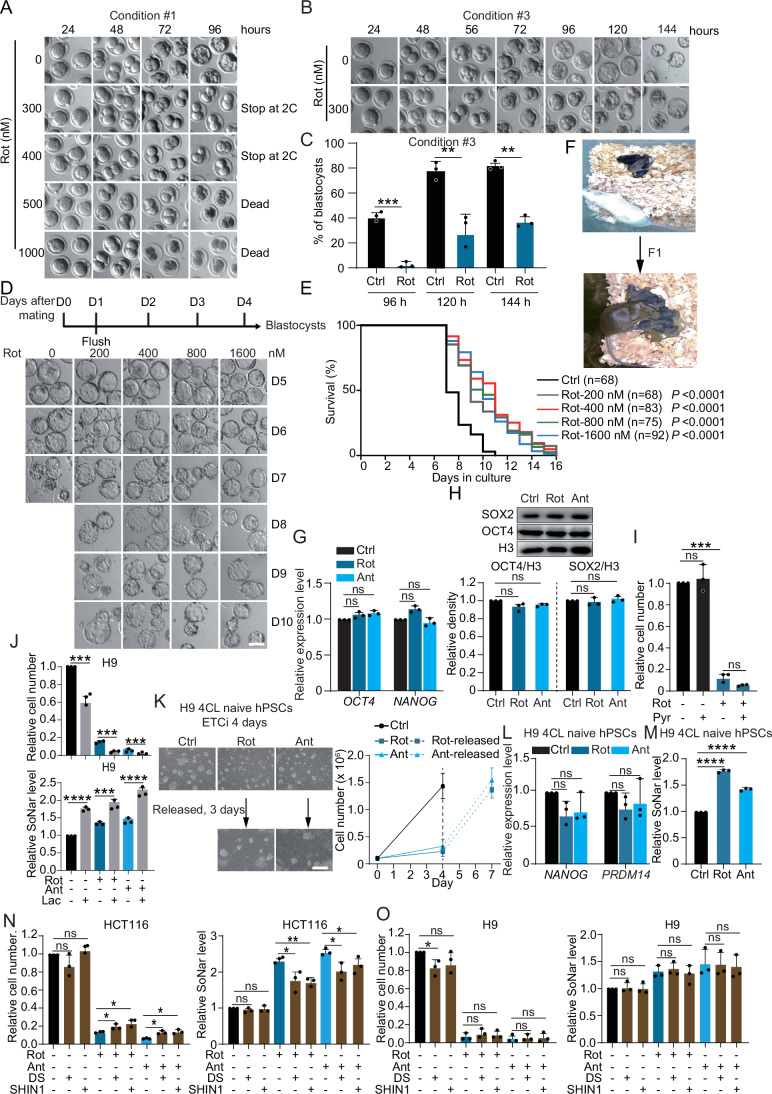
ETC inhibition induces pausing in early embryonic development and human PSCs, related to Fig. 4. (**A**) Images of developing embryos from control and condition 1 treated with different doses of Rot starting at 24 h post-mating, scale bar, 200 µm. (**B**,** C**) Images (**B**) and percentage (**C**) of developing embryos from control and condition #3 at the indicated time points (mean ± SD, *n* = 3 each with biological replicates), scale bar, 200 µm. (**D**) Collection and culture of fertilized eggs for blastocyst generation ex vivo. Blastocysts were treated with indicated doses of Rot and cultured until the blastocysts collapsed, scale bar, 200 µm. (**E**) Kaplan–Meier survival curves of blastocysts treated with rotenone (Rot). *n* represents the total number of blastocysts per condition, pooled from three independent experiments showing consistent survival trends. Statistical significance was determined by the log-rank (Mantel–Cox) test, comparing each Rot dose to control (*p* < 0.0001). (**F**) Images of live pups (F0, upper image) born from blastocysts treated with Rot 400 nM for 3 days and F1 live mice generated from F0 (lower image). (**G**) Relative expression levels of *OCT4* and *NANOG* in H9 primed hESCs treated with Rot or Ant for 4 days (mean ± SD, *n* = 3 each with biological replicates). (**H**) Western blotting for OCT4 and SOX2 and relative density of H9 primed hESCs treated with Rot or Ant for 4 days. H3 was used as the loading control (mean ± SD, *n* = 3 each with biological replicates). (**I**) Relative cell number of H9 primed hESCs treated with or without Rot and pyruvate (2 mM) in mTeSR (mean ± SD, *n* = 3 each with biological replicates). (**J**) Relative cell number and relative SoNar levels from H9 primed hESCs treated with Rot or Ant and with and without lactate (10 mM) for 4 days (mean ± SD, *n* = 3 each with biological replicates). (**K**) Cell images and cell number of H9 4CL naive hESCs treated with Rot (150 nM) or Ant (250 nM) for 4 days and released for 3 days in 4CL (mean ± SD, *n* = 3 each with biological replicates), scale bar, 200 µm. (**L**) Relative expression levels of pluripotent markers of H9 4CL naive hESCs treated with Rot or Ant for 4 days in 4CL (mean ± SD, *n* = 3 each with biological replicates). (**M**) Relative SoNar levels of H9 4CL naive hESCs treated with Rot or Ant for 4 days in 4CL medium (mean ± SD, *n* = 3 each with biological replicates), scale bar, 200 µm. (**N**,** O**) Relative cell number and relative SoNar levels (HCT116) (**N**), relative cell number and relative SoNar levels (H9) (**O**), with the indicated treatments for 4 days (mean ± SD, *n* = 3). Data information, in Fig. EV5C, G, H–J, L–O, columns represent mean, points are individual biological replicates; error bars are standard deviation, lines indicate comparisons between different groups; stars indicate statistical significance. *p < 0.05, **p < 0.01, ***p < 0.001, ****p < 0.0001; ns not significant by unpaired two-tailed Student’s *t*-test. Individual *p* values are provided in Table EV1.

In mouse 2C embryos, oxidative phosphorylation (OXPHOS) is minimally active, with a higher NADH/NAD^+^ ratio than in later stages, indicating limited NAD^+^ recycling activity (Sharpley et al, 2021; Zhao et al, 2021). However, during the transition from early to late 2C stage, a decrease in cytoplasmic redox has been observed (Sun et al, 2023), suggesting the initiation of NAD^+^ recycling. To determine the necessity of ETC for 2C embryo development, zygotes were isolated 18 h post-mating and treated with Rot 6 h later, before the first cell division, for varying durations (Fig. 4A). Continuous treatment within a 300–400 nM concentration range effectively halted embryonic development at the 2C stage (Fig. EV5A). Next, we pinpointed the maximum duration of Rot treatment that does not affect the rate of blastocyst development and identified an optimal 24-h window of 24–48 h (condition #2, Fig. 4A–C), while extending the treatment to 32 h (24–56 h, condition #3) decreased the developmental rate (Fig. EV5B–C). 2C embryos from condition #2 were successfully implanted into surrogate mice and could produce viable and fertile offspring (Fig. 4D), validating the paused state.

Next, we investigated whether a similar developmental pause occurs in blastocysts due to ETC blockade. Zygotes were cultured ex vivo from post-mating until blastocyst formation over 3 days. These blastocysts remained viable for an additional three days before deteriorating. Treatment with Rot at varying doses during this period showed that, while untreated blastocysts began to deteriorate by day 7, those treated with Rot experienced a 2- to 5-day extension in viability (Fig. EV5D,E). Following a 3-day treatment with Rot starting from day 4 and a subsequent 1-h recovery period, blastocysts were injected into surrogate mice, resulting in the birth of viable and fertile mice (Fig. EV5F), confirming that ETC C1 blockade induces a reversible paused state in mouse blastocysts. Sufficient pyruvate was included in the culture media for both the 2C and blastocyst stages. These results underscore the critical role of the ETC in the developmental transitions of both the 2C stage and blastocysts, where its inhibition leads to a developmental pause.

In human primed state PSCs, inhibition of the ATP synthase by oligomycin halted self-renewal and triggered differentiation (Khoa et al, 2020). Revisiting the role of ETC in H9 primed hESCs using Rot and Ant, we found that a 4-day treatment significantly inhibited self-renewal but did not affect pluripotency gene expression (Figs. 4E,F and EV5G). The cells resumed self-renewal upon inhibitor withdrawal and could maintain normal morphology and pluripotency gene expression (Fig. EV5H), confirming a paused pluripotent state in ETC inhibition. Because the H9 primed hESCs culture medium, mTeSR, contains ~0.4 mM pyruvate, we added an extra 2 mM pyruvate but couldn’t see any rescue effect on self-renewal for the Rot-induced paused state (Fig. EV5I). The redox levels, as measured by SoNar, increased significantly, though to a lesser degree than in mESCs (Fig. 4G). Like in mESCs, adding lactate inhibits the cell proliferation and increases the SoNar level with and without ETC inhibitors in H9 primed hESCs (Fig. EV5J), supporting a conserved role of reductive stress in mediating pausing in hESCs. Moreover, overexpression of *Ndi1* in Rot-treated H9 primed hESCs rescued both self-renewal and redox levels (Fig. 4H,I). In the H9 human 4CL naive ESCs (Mazid et al, 2022), paused pluripotency could also be induced by Rot and Ant, accompanied by reductive stress (Fig. EV5K–M). These findings suggest that ETC inhibition induces a paused pluripotent state in both human primed and naive PSCs that is accompanied by reductive stress, paralleling observations in mESCs.

Because TDH is a non-functional pseudogene in humans (Pruitt et al, 2007), we anticipated that hPSCs rely on a different redox mechanism to sustain NADH production under ETC inhibition. Based on our mESC findings, we reasoned that certain dehydrogenases enriched in hPSCs might serve a similar role. To explore whether the 1C pathway is similarly involved in hPSCs, we compared the expression of 1C enzymes between hESCs and human embryonic fibroblasts (HEFs) using published RNA-seq data (Guan et al, 2022). Most 1C enzymes were upregulated in hESCs, with particularly strong enrichment of the glycine cleavage system (GCS) (Fig. 4J). Because specific GCS inhibitors are not available, we used inhibitors of SHMT1/2 (SHIN1) and MTHFD2 (DS18561882) (Fig. 4K). Individually, each inhibitor partially rescued proliferation and SoNar signals in HCT116 cells during ETC inhibition but showed no effect in H9 hESCs (Fig. EV5N,O). Given the strong GCS enrichment in primed H9 hESCs, we hypothesized that dual inhibition might be required. Indeed, combined treatment with DS and SHIN1 significantly rescued self-renewal in rotenone-treated H9 primed H9 hESCs and markedly reduced the total NADH/NAD^+^ ratio (Fig. 4L,M).

In summary, our study supports a model in which NADH reductive stress—likely contributed to by one-carbon (1C) metabolism in hPSCs—is associated with the paused phenotype during ETC inhibition.

## Discussion

In summary, our study supports a model in which NADH reductive stress contributes to ETC inhibition-induced pausing in both mouse and human PSCs, with TDH serving as a major source in mESCs and one-carbon (1C) metabolism likely contributing in hPSCs. In mESCs, TDH represents a major contributor to reductive stress and, through this metabolic constraint, may influence the relative fitness of cells with compromised mitochondrial or genomic integrity (Fig. 4N). We further show that ETC inhibition induces developmental pausing in both blastocysts and two-cell embryos even in the presence of pyruvate, supporting consistency between in vitro models and in vivo early embryogenesis. These findings are consistent with the possibility that cells harboring mtDNA mutations may be selectively disadvantaged during early embryogenesis (Wang et al, 2024) and may facilitate more controlled perturbations of embryogenesis ex vivo. Several questions emerge from this work. For example, how does suppression of PDH activation contribute to stability maintenance in mESCs, and is PDH similarly targeted by mitochondrial reductive stress in human PSCs? Moreover, might PDH activation generally compromise genomic or epigenetic stability across pluripotent states? Addressing these issues will deepen our understanding of how redox metabolism interfaces with developmental potential. Technically, flux-based isotope tracing in future work would further strengthen conclusions regarding pathway specificity and directionality. Finally, based on our findings, and recent advances in mitochondrial health (Lima et al, 2021) and reductive stress biology (Pan et al, 2024; Yang et al, 2022), whether similar redox-driven pausing mechanisms operate in other stem cell types or differentiated cells remains an open question for future investigation. Such work may provide a conceptual framework for research in stem cell biology, regeneration, and aging.

## Methods

**Table:** Reagents and tools table

Reagent/Resource	Reference or source	Identifier or Catalog Number
**Experimental models**		
mESCs: E14tg2a (E14)	Dr. Igor M. Samokhvalov	GIBH
HEK293T	ATCC	CRL-3216
NIH3T3	ATCC	CRL-1656
HCT116	Dr. Li Zhiyuan	GIBH
Human Primed H9 ESCs	WiCell Research Institutes	(USA)
MEFs (feeder cells)	This paper	N/A
CD-1®(ICR)	Charles River Laboratory	2021-210
C57BL/6NCrl	Charles River Laboratory	493
**Recombinant DNA**		
Ndi1	Addgene	72876
Cyto-*Lb*Nox	Addgene	75285
Mito-*Lb*NOX	Addgene	74448
pLKO.1-TRCcloning vector	Addgene	10878
**Antibodies**		
Anti-β-ACTIN	Sigma-Aldrich	A2066
Anti-OCT4	Santa Cruz Biotechnology	Sc-8628
Anti-NANOG	Bethyl Laboratories	A300-397A
Anti-SOX2	Bethyl Laboratories	MAB2018
anti-Flag	Sigma-Aldrich	F1804
Anti-H3K4me1	Abcam	ab8895
Anti-H3K4me3	Abcam	ab8580
Anti-H3K9me3	Abcam	ab8898
Anti-H3K9ac	Abcam	ab10812
Anti-H3K27ac	Abcam	ab4729
Anti-H3K27me3	Millipore	07-449
Anti-H3ac	Millipore	06-599
Anti-H4ac	Millipore	06-866
Anti-H3	Abcam	ab1791
Anti-PDH	Cell Signaling	3205S
Anti-p-PDH-E1α-Ser232	Millipore	AP1063
Anti-5mC	Cell Signaling	D3S2Z
Anti-HRP goat anti-rabbit	Beyotime	A0208
Anti-HRP goat anti-mouse	Beyotime	A0216
**Oligonucleotides and other sequence-based reagents**		
**Target sequences for shRNAs**		
*Risp*#1	5′CCGGCGAGATCAAGTTGTCTGATATCTCGAGATATCAGACAACTTGATCTCG TTTTTG3′	
*Risp*#2	5′CCGGGCAATGTCTTTGATAGCTAATCTCGAGATTAGCTATCAAAGACATTGC TTTTTG3′	
**Target sequences for sgRNA**		
Mouse- *Polg* sgRNA-ki-1	5′CCTGATATGGGCTCGGTCAA3′	
Mouse- *Polg* sgRNA-ki-2	5′CCTTTGACCGAGCCCATATC3′	
*Polg* target sequence	5′CCTTTGACCGAGCCCATATCAGG3′	
**Primers sequences for** ***Polg*** **wildtype and mutant donor**		
*Polg* WT FW	5′TGAGTGGCGTGAGGAGCAGC3′	
*Polg* WT RV	5′TTCAAACACCATGCTAGTGTG3′	
*Polg* Mut-Donor FW	5′GGTGGGGCACAATGTTTCCTTCGCAAGGGCACACATTAGGGAACAGTATCTGATTCAGG3′	
*Polg* Mut-Donor RV	5′CCTGAATCAGATACTGTTCCCTAATGTGTGCCCTTGCGAAGGAAACATTGTGCCCCACC3′	
**Primers for amplifying the whole mtDNA of mouse E14 ESCs**		
Region 1872–6222 FW	5′GGAATGCCTAAAGGAAAGATCCAAAAAGATAA3′	
Region 1872–6222 RV	5′CATCTAATCCTACTGTGAAT3′	
Region 6203-10627 FW	5′ATTCACAGTAGGATTAGATG3′	
Region 6203-10627 RV	5′AGGGTATAAAATAGGAAA3′	
Region 10622-1871 FW	5′TACCCTAATCGGTTCTATTCCACTGCTAATTG3′	
Region 10622-1871 RV	5′GGTGTTGGGTTAACAGAGAAGTTATAGGTGGA3′	
**Primers for RT-qPCR**		
*Actb* FW	5′TGCTAGGAGCCAGAGCAGTA3′	
*Actb* RV	5′AGTGTGACGTTGACATCCGT3′	
*Risp* FW	5′GAGCCACCTGTTCTGGATGTG3′	
*Risp* RV	5′GCACGACGATAGTCAGAGAAGTC3′	
*Oct4* FW	5′TAGGTGAGCCGTCTTTCCAC3′	
*Oct4* RV	5′GCTTAGCCAGGTTCGAGGAT3′	
*Nanog* FW	5′CTCAAGTCCTGAGGCTGACA3′	
*Nanog* RV	5′TGAAACCTGTCCTTGAGTGC3′	
*Sox2* FW	5′AGGGCTGGGAGAAAGAAGAG3′	
*Sox2* RV	5′CCGCGATTGTTGTGATTAGT3′	
*Klf4* FW	5′ACCGAAAGCACACAGGTCA3′	
*Klf4* RV	5′CATGTGTCGCTTCATGTGC3′	
*Esrrb* FW	5′GCACCTGGGCTCTAGTTGC3′	
*Esrrb* RV	5′TACAGTCCTCGTAGCTCTTGC3′	
*Rex1* FW	5′TCTTCTCTCAATAGAGTGAGTGTGC3′	
*Rex1* RV	5′GCTTTCTTCTGTGTGCAGGA3′	
*Dppa5a* FW	5′ATGATGGTGACCCTCGTGAC3′	
*Dppa5a* RV	5′ACCTCGATAAGTTCTTCGGGAG3′	
Homo sapiens *ACTB* FW	5′CCACGAAACTACCTTCAACTCC3′	
Homo sapiens *ACTB* RV	5′GTGATCTCCTTCTGCATCCTGT3′	
Homo sapiens *OCT4* FW	5′GCTCGAGAAGGATGTGGTCC3′	
Homo sapiens *OCT4* RV	5′CGTTGTGCATAGTCGCTGCT3′	
Homo sapiens *NANOG* FW	5′GCAGAAGGCCTCAGCACCTA3′	
Homo sapiens *NANOG* RV	5′AGGTTCCCAGTCGGGTTCA3′	
Homo sapiens *PRDM14* FW	5′CGTTCTGTACGGGGTCACTC3′	
Homo sapiens *PRDM14* RV	5′TTGAGGAAGAGAATCAGATCCAG3′	
**Chemicals, enzymes and other reagents**		
Neurobasal	Gibco	21103-049
DMEM/F12 (50/50)	Hyclone	10-092-CVRC
Advanced DMEM/F12	Gibco	12634028
GlutaMAX (100X)	Gibco	35050-061
NEAA (100X)	Corning	25-025-CL
Sodium pyruvate	Corning	25-000-CL
Penicillin/streptomycin	Hyclone	SV30010
β-Mercaptoethenol	Gibco	2198503
N2 supplement 100X	Gibco	17502048
B27 supplement 50X	Gibco	17504044
PD0325901	StemRD	PD-50
CHIR99021	StemRD	CHIR-50
Mouse leukemia inhibitory factor (mLif)	Lofetech	L00100G
Human LIF	Peprotech	300-05
DZNep	Selleck	S7120
TSA	Vetec	V900931
IWR-1	Sigma-Aldrich	I0161
ACTIVIN A	Peprotech	120-14E
L-ascorbic acid	Sigma-Aldrich	A8960
Geltrex	Thermo Fisher Scientific	A1413302
Y-27632	Axon	1683
DMEM High Glucose	HyClone	10-017CV
FBS	Biowest	S1580-500
mTeSR^TM^1 medium	STEMCELL Technologies	85850
DMEM, without glucose, glutamine, and phenol red medium	Thermo Fisher Scientific	A1443001
Neurobasal™-A Medium, without D-glucose and sodium pyruvate	Thermo Fisher Scientific	A2477501
Opti-DMEM	Thermo Fisher Scientific	31985070
KSOM + AA	Caisson labs	IVL04
XF Base Medium	Seahorse	102353-100
Trypsin 0.25%	Gibco	25200-056
TrypLE™ Express Enzyme (1X), no phenol red	Thermo Fisher Scientific	12604013
DMSO	Sigma-Aldrich	D1435
Rotenone	Sigma-Aldrich	R8875
Pericidine A	Sigma-Aldrich	PHR1084
Antimycin A	Abcam	ab141904
Myxothiazol	Sigma-Aldrich	T5580
Oligomycin A	Selleck	S1478
Dimethyl malonate (DMM)	Sigma-Aldrich	136441
Sodium azide (NaN3)	Sigma-Aldrich	S2002
α-Ketobutyric acid	Sigma-Aldrich	K401
L-aspartic acid dimethyl ester hydrochloride	Sigma-Aldrich	456233
L-Aspartic acid	Sigma-Aldrich	A9256
L-Threonine	Sigma-Aldrich	T8625
NAD^+^	Sigma-Aldrich	N0632
NHI-2	Sigma-Aldrich	SML1463
Glucose	Sigma-Aldrich	G7021
2-Deoxy-D-glucose	Sigma-Aldrich	D8375
QC1	Sigma-Aldrich	SML048
DS18561882	MedChemExpress	HY-130251
SHIN1	MedChemExpress	HY-112066
AZD7545	Selleck	Cat# S7517
UK5099	Sigma-Aldrich	PZ0160
β-Fluoropyruvic acid sodium salt monohydrate (3-FP)	Sigma-Aldrich	F4004
Sodium acetate anhydrous	Sigma-Aldrich	71183
N,N‑Dimethylglycine hydrochloride	Sigma-Aldrich	D6382
Puromycin	Selleck	S7417
Polybrene	Sigma-Aldrich	107689
BSA	Sigma-Aldrich	B2064
SYBR Premix ExTaq green	Takara	RR420A
KOD One TM PCR Master Mix	TOYOBO	KMM-101
SDS	LabLead Amersco	GS286
PVDF membrane	Merck Millipore	IPVH00010
Nylon transfer membrane	AMERSHAM HYBOND	RPN303B
Tween 20	LabLead Amersco	0777
Western Bright ECL	Advansta	K-12045-D50
DPBS	Yeasen Biotechnology	60152ES76
NaOH	Macklin	S817977
Methylene blue	Sigma-Aldrich	M9140
Triton X-100	Macklin	T6328
Sucrose	Sigma-Aldrich	S0389
SYBR^TM^ Gold	Invitrogen	S11494
Nocodazole	Selleck	S2775
KCL	Macklin	P816348
VECTASHIELD® Vibrancerm Antifade Mounting Medium with DAPl	Vector laboratories	H-1800-10
**Critical commercial assays**		
NAD^+^/NADH quantitation colorimetric kit	BioVision	K337
Seahorse XFe24 FluxPak	Seahorse/Agilent Technologies	102340-100
Xfe 24V7PS cell culture microplates	Seahorse/Agilent Technologies	100777-004
XF cell mito stress kit	Seahorse/Agilent Technologies	103015-100
Genomic DNA extraction kit	Promega	A1120
RNAzol^®^ RT kit	Molecular Research Center (MRC)	RN190
DCFH-DA	Beyotime	S0033
Lipofectamine 3000 transfection kit	Thermo Fisher Scientific	L3000015
**Software**		
GraphPad Prism, v8	GraphPad	https://www.graphpad.com/scientificsoftware/prism/
FlowJo_v10	FlowJo LLC	https://docs.flowjo.com:443
Comet score 2.0, 2017	Comet score 2.0	http://rexhoover.com/index.php?id=cometscore
ImageJ V1.51	ImageJ	https://imagej.net/ij/

### Animal experiments and ethics statement

All animal experiments in this study were approved by the Animal Care and Use Committee of the Guangzhou Institutes of Biomedicine and Health (GIBH), Chinese Academy of Sciences (CAS), under license number 2016012. The wild-type mice C57BL/6 (stock no. 493) and ICR (stock no. 2021-210) were purchased from Charles River Laboratory. The mice were kept in a 12-h light/dark cycle at 22–24 °C in a pathogen-free environment with a relative humidity of 40–70%. Female mice, aged 3–4 months, and male mice, aged 8–12 months, were used in the experiments. All hESCs studies were approved by the Guangzhou Institutes of Biomedicine and Health under license number GIBH-LMEC2023-001-01(AL).

### Cell culture

mESCs (E14gt2a, E14) were cultured on 0.2% gelatin precoated plates in a mixture of neurobasal (Gibco, cat. 21103-049) and DMEM/F12 (Hyclone, cat. 10-092-CVRC) (1:1 ratio), supplemented with 1% GlutaMax (Gibco, cat. 35050-061), 1% non-essential amino acids (NEAA) (Corning, cat. 25-025-CL), 1% sodium pyruvate (Corning, cat. 25-000-CL), 0.5% penicillin-streptomycin (P/S) (Hyclone, cat. SV30010), 0.1 mM β-mercaptoethanol (Gibco, cat. 2198503), 0.5% N2 (Gibco, cat. 17502048), 1% B27 supplements (Gibco, cat. 17504044),1 µM PD0325901 (StemRD, cat. PD-50), 3 µM CHIR99021 (StemRD, cat. CHIR-50), and 1000 U/ml mLIF (Lofetech, cat. L00100G). Human primed H9 ESCs were maintained on Geltrex (Thermo Fisher Scientific) with mTeSR^TM^ 1 medium (STEMCELL Technologies, cat. 85850). NIH3T3, HEK293T, and ICR mouse embryonic fibroblast-derived feeder cells (mitomycin-treated) were cultured in DMEM with high glucose (HyClone, cat. 10-017CV), supplemented with 10% fetal bovine serum (FBS, Biowest, cat. S1580-500), 1% GlutaMax, 1% NEAA, 0.5% P/S, and 0.1 mM β-mercaptoethanol. For the induction of 4CL naive hESCs, primed hESCs were washed with DPBS (Yeasen Biotechnology, cat. 60152ES76), digested into single cells using TrypLE (Thermo Fisher Scientific, cat. 12604013), and plated at a density of 50,000 cells/well in 12-well plates on feeder cells in mTeSR^TM^ 1 medium with 10 μM Y-27632 (Axon. 1683). After 24 h, the medium was replaced with 4CL medium, composed of a 1:1 mixture of neurobasal medium and advanced DMEM/F12 (Gibco, cat. 12634028), supplemented with 1% sodium pyruvate, 1% NEAA, 1% GlutaMax, 0.5% P/S, 0.5% N2, 1% B27 supplements, 1 µM PD0325901, 5 nM TSA (Vetec, cat. V900931), 20 ng/ml human LIF (PeproTech, cat. 300-05), 10 nM DZNep (Selleck, cat. S7120), 5 µM IWR-1 (Sigma-Aldrich, cat. I0161), 20 ng/ml ACTIVIN A (Peprotech, cat. 120-14E), 50 µg/ml L-ascorbic acid (Sigma-Aldrich, cat. A8960), and 0.2% (v/v) Geltrex^TM^ (Thermo Fisher Scientific, cat. A1413302) or Matrigel® (Corning, cat. 354277). The 4CL medium was refreshed daily, and cells were passaged as single cells (1:5 to 1:8) every 3 to 4 days. Optionally, 5 μM Y-27632 was added for the first 24 h. Cells were cultured until four passages, and the induction of naive pluripotency was confirmed by analyzing the naive pluripotency gene expression with RT-qPCR. All cells were routinely checked for mycoplasma contamination (MycoAlert, Lonza, cat. LT07-318).

### Molecular cloning and production of lentivirus particles

*Ndi1* (cat. 72876), Cyto-*Lb*Nox (cat. 75285), and Mito-*Lb*NOX (cat. 74448) were purchased from Addgene and SoNar cDNA was gifted by Prof. Yi Yang and Prof. Yuzheng Zhao (East China University of Science and Technology, Shanghai, China). pBabe.hygro-mCherry-GFP FIS (101–152) [DU 40799], was gifted by I. G. Ganley (The University of Dundee, UK). cDNAs were sub-cloned into the pKD-IRES- Puro vector. Mouse Risp oligonucleotides were annealed and cloned into the pLKO.1 vector for shRNA. For the knock-in mutation of *Polg*, sgRNAs targeting the *Polg* mutation site (D257A) were designed using the CRISPR tool and cloned into the lenti-CRISPR v2 vector containing Cas9. The mutant (D257A) *Polg* knock-in donor was inserted into the pMD19-T vector. All shRNA and sgRNA sequences are listed in the Reagents and tools table. Overexpression vectors were transfected into HEK293T cells with packaging plasmids psPAX2 and pMD.2 G to generate lentivirus particles using Lipofectamine® 3000. After 12 h, the medium was replaced with fresh medium and incubated for 36 h. The virus particles were collected, filtered through 0.22 µm filters, and stored at −80 °C.

### RNA extraction and RT-qPCR

Total RNA was extracted using the RNAzol^®^ RT kit (Molecular Research Center (MRC), cat. RN190) according to the manufacturer’s instructions. Briefly, 2 µg of RNA was used for cDNA synthesis with a reverse transcription kit (Takara, cat. RR037A). RT-qPCR was performed in triplicate using an ABI7500 machine with SYBR Premix ExTaq green (Takara, cat. RR420A). Data were normalized to *Actb* expression, and primer sequences are listed in the Reagents and tools table.

### Western blotting

Cultured cells were washed twice with DPBS, digested, and counted. At least 1 million cells were collected in lysis buffer (50 mM Tris-HCl, pH 6.8, 2% SDS, 1% bromophenol blue, and 10% glycerol). Samples were boiled for 5 min, subjected to SDS–PAGE, and transferred to a 0.45 µm PVDF membrane (Merck Millipore, cat. IPVH00010). The membrane was blocked with 5% milk (Merck Millipore, cat. 20-200) or BSA (Sigma-Aldrich, cat. B2064) in TBST (50 mM Tris-HCl, pH 8.0, 150 mM NaCl, and 0.1% Tween 20 (LabLead Amresco, cat. 0777) and incubated with primary antibodies diluted 1:1000 for at least 2 h at room temperature or overnight at 4 °C. After washing five times with TBST, the membrane was incubated with secondary antibodies diluted 1:2000 for 1 h at room temperature. The membrane was washed again, and signals were detected using Western Bright ECL (Advansta cat. K-12045-D50), visualized with the FUSION SOLO 4 M machine (Vilber Lourmat). Primary antibodies used were: anti-ACTIN (Sigma, cat. A2066), anti-OCT3/4 (Santa Cruz, cat. sc-8628), anti-NANOG (BETHYL, cat. A300-397A), anti-SOX2 (R&D, cat. MAB2018), Anti-Flag (Sigma, cat. F1804), anti-H3K4me1 (Abcam, cat. ab8895), anti-H3K4me3 (Abcam, cat. ab8580), anti-H3K9me3 (Abcam, cat. ab8898), anti-H3K9ac (Abcam, cat. ab10812), anti-H3K27ac (Abcam, cat. ab4729), anti-H3K27me3 (Millipore, cat. 07-449), anti-H3ac (Millipore, cat. 06-599), anti-H4ac (Millipore, cat. 06-866), anti-Histone H3 (Abcam, cat. ab1791), anti-PDH (Cell signaling, cat. 3205), anti-p-PDH-E1α-Ser232 (Millipore, cat. AP1063). Primary antibodies were diluted in a 1:1000 ratio except for histone H3 (1:3000).

### Mouse embryo culture

Fertilized eggs or pre-implantation embryos were collected from super-ovulated F1 female mice. Female mice were injected intraperitoneally with 7.5 IU of pregnant mare serum gonadotropin (PMSG, Ningbo Sansheng Biological Technology Co., Ltd.) to stimulate egg production. After 48 h, 7.5 IU of human chorionic gonadotropin (HCG, Ningbo Sansheng Biological Technology Co., Ltd.) was injected intraperitoneally, and females were mated with males in a 1:3 ratio. Mating was confirmed by the presence of a vaginal plug, and females were euthanized 18 h post-HCG. The uterus was separated, and zygotes were isolated for experiments or cultured for blastocyst generation in KSOM + AA (Caisson labs, cat. IVL04) medium at 37 °C in 5% CO_2_. For blastocyst pausing, blastocysts were treated with DMSO (Sigma-Aldrich) or Rotenone (Sigma-Aldrich) at the indicated concentrations. After 3 days of Rot treatment, the blastocysts were injected back into surrogate mothers for live pups or continued in vitro culture. For chimera generation, blastocysts were isolated from ICR mice at day 3.5. Rot-treated mESCs were injected into the blastocysts through microinjection. After 2 h, the blastocysts were transplanted into surrogate mothers, and the results were analyzed after birth.

### Metabolic measurements

#### Non-targeted metabolomics

E14 mESCs were washed twice with precooled DPBS, digested, and collected in a 15 ml tube. The samples were washed again with 0.9% NaCl saline and suspended in lysis buffer (methanol: acetonitrile:ultrapure water, 2:2:1, v/v). Samples were collected in a 1.5 ml EP tube, flash-frozen in liquid nitrogen, and transferred to −80 °C until sent for metabolomics measurement at Shanghai Applied Protein Technology and Biotree Biotech Co, Ltd, Shanghai, China. Each sample was thawed slowly at 4 °C and vortexed thoroughly. Samples were incubated at −20 °C for 60 min to precipitate proteins, then centrifuged at 13,000 rpm at 4 °C for 15 min. The supernatants were collected, dried under vacuum, and stored at −80 °C. Samples were dissolved in 100 µL of acetonitrile/water (1:1, v/v), vortexed, and centrifuged at 14,000 rpm at 4 °C for 15 min. The supernatants were collected for LC-MS/MS analysis. The analysis was performed using a UHPLC (1290 Infinity LC, Agilent Technologies) attached to a quadrupole time-of-flight (AB Sciex Triple TOF 6600). For HILIC partitioning, samples were passed through a 2.1 mm × 100 mm ACQUITY UPLC BEH 1.7 µm column (Waters, Ireland). For metabolite separation, mobile phase A consisted of 95:5 water: acetonitrile with 25 mM ammonium acetate and 25 mM ammonium hydroxide, while mobile phase B was acetonitrile. The gradient of mobile phase B started at 85% for 1 min, linearly decreased to 65% over 11 min, gradually reduced to 40% in 0.1 min, held for 4 min, then returned to 85% in 0.1 min, followed by a 5-min re-equilibration. The ESI source conditions were set with ion source gases 1 and 2 at 60 psi, curtain gas at 30 psi, a temperature of 600 °C, and an ion spray voltage of ±5500 V. The MS instrument acquisition m/z range was 60–1000 Da, with an accumulation time of 0.2 s/spectra for the TOF MS scan. For auto MS/MS acquisition, the m/z range was 25–1000 Da, with a production time of 0.05 s/spectra for the product scan. Information-dependent acquisition and high-sensitivity modes were used for scanning product ions. Collision energy was fixed at 35 V ± 15 eV, with declustering potential set to ±60 V. ProteoWizard software was used to convert raw MS data into an MzXML file, and XCMS was employed for feature detection, alignment, and retention time correction. Metabolites were identified by accurate mass (<25 ppm), and MS/MS data were matched against a standards database. In feature ion extraction, variables with over 50% nonzero values in at least one group were retained. Differential metabolites were identified using a >1.5-fold change with a *p* value <0.05. The metabolomics data were normalized to cell number.

#### Total NADH/NAD^+^ measurement

The NADH/NAD^+^ ratio was measured using the NADH/NAD^+^ measurement kit (BioVision, cat. K337), following the manufacturer’s instructions. Cells were washed twice with precooled DPBS, and at least 0.5 million cells were lysed in 400 µl of NADH/NAD^+^ extraction buffer, homogenized, and vortexed for 20 s. Samples were centrifuged at 14,000 rpm for 5 min, and the supernatant was collected. For total NADt (NADH and NAD^+^), 50 µl of the supernatant was transferred to 96-well plates. For NADH detection, 200 µl of the supernatant was transferred to an EP tube and heated at 60 °C for 30 min. After cooling, 50 µl of the sample was transferred to 96-well plates. A reaction mixture was prepared by adding 98 µl of NAD^+^ cycling buffer and 2 µl of NAD^+^ cycling enzyme mixture per sample. About 100 µl of the reaction mixture was added to each sample, mixed, and incubated at room temperature for 5 min. Then, 10 µl of NADH developer was added to each sample and incubated for 4 h at room temperature. Optical density (OD) was measured at 450 nm using a microplate reader (Promega). The NADH/NAD^+^ ratio was calculated as (NADH/[NADt − NADH]) and normalized to cell number.

#### Reactive oxygen species (ROS) measurement

Total ROS levels were measured using a DCFH-DA kit (Beyotime, cat. S0033), following the manufacturer’s instructions. A 10 mM stock solution of DCFH-DA was diluted 1000 times in DPBS. Cells were digested, counted in basal medium, and washed twice with DPBS. 0.5 million cells were incubated in 500 µl of DCFH-DA at 37 °C for 30 min with occasional shaking. Samples were then centrifuged, the DCFH-DA was removed, and cells were washed twice with DPBS. Fluorescence was measured using BD6 flow cytometry.

#### SoNar measurement

E14 mESCs, H9 human primed ESCs, H9 human naive ESCs, and NIH3T3 cells were transduced with pKD-IRS-SoNar-Puro, lentivirus diluted in a 1:1 ratio with culture medium in the presence of polybrene (Sigma-Aldrich, cat. 107689) (8 µg/ml). After 12 h, the medium was replaced with fresh medium, and cells were selected with puromycin (Selleck, cat. S7417) (1 µg/ml) for 2 days. To evaluate the SoNar fluorescence ratio, cells were excited at 405 and 488 nm, and emission was measured at 525 nm using a BD LSRFortessa™ Cell Analyzer. FlowJo software v10 was used to calculate the mean fluorescence intensities of SoNar cells and normalize them against blank cells.

#### TDH enzymatic activity measurement

TDH enzymatic activity was measured following the method of Jian Wang (Wang et al, 2009) with some modifications. Briefly, E14 mESCs were washed, digested, and counted. Ten million cells were suspended in 10 mM Tris-HCl (pH 7.5), 250 mM sucrose, and 2 mM EDTA and homogenized using a pestle douncer on ice. The lysate was centrifuged at 600 × *g*, 4 °C for 10 min. The supernatant was collected and suspended in a mitochondrial suspension buffer (100 mM KH_2_PO_4_ (pH 7.4), 0.1% NP-40, 10 mM DTT, and 1x protease inhibitor cocktail) and centrifuged at 11,000 × *g*, 4 °C for 10 min. The pellet was suspended in 50 µl of mitochondrial suspension buffer. TDH activity was determined by the formation of NADH from NAD^+^ at 25 °C. The assay mixture contained 100 mM Tris-HCl (pH 8.0), 25 mM L-threonine, 5 mM NAD^+^, 25 mM NaCl, and 3 µl of mitochondrial protein crude extract in a final volume of 100 µl. The absorbance was measured with a microplate reader (Promega) at 340 nm in 5-min intervals.

#### OCR/ECAR measurement

The oxygen consumption rate (OCR) was measured using a Seahorse XFe24 analyzer according to the manufacturer’s instructions (Seahorse XFe24 FluxPak, cat. 102340-100, Xfe 24V7PS cell culture microplates, (cat. 100777-004) and XF cell mito stress kit, cat. 103015-100). Briefly, 1 ml of XF calibrant buffer was added to each well of the utility plate and incubated at 37 °C overnight in a non-CO_2_ incubator. For OCR measurement, the XF basal medium was supplemented with 10 mM glucose (Sigma-Aldrich, cat. G7021), 1 mM sodium pyruvate, and 2 mM glutamine, and the pH was adjusted to 7.4 using 1 N NaOH. The medium was filtered using a 0.22 µm filter. About 8 µM FCCP and 10 µM Rotenone/Antimycin A were prepared independently in OCR XF medium. Then, 75 µl of FCCP was loaded into Port A, 75 µl of Rotenone/Antimycin A into Port B, and 75 µl of XF medium into Ports C and D. The cartridge was loaded into the XFe24 analyzer for calibration. After digestion, 0.3 million cells were seeded in each well of a 24 XF plate with XF OCR medium, and the volume was adjusted to 525 µl. The cell plate was incubated in a non-CO_2_ incubator for 1 h. After cartridge calibration, the utility plate was replaced with the cell plate and loaded into the XF24 analyzer. For the ECAR measurements, the XF glycolysis stress test kit (cat. 102194-100) was used. XF basal medium was supplemented with 2 mM glutamine was prepared and the pH was adjusted to 7.4 using 1 N NaOH. About 80 mM glucose and 900 mM 2-DG (Sigma, cat. D8375) were prepared independently in ECAR XF medium. Then, 75 µl of glucose into Port A, 75 µl of 2-DG into Port B, and 75 µl of XF medium into Ports C and D were loaded. The other procedure was followed as an OCR measurement. OCR and ECAR were normalized with the cell number. The results were analyzed following the completion of the assay.

### Cell competition

E14 mESCs were transduced with either blank or DsRed^+^ lentivirus vector diluted in a 1:1 ratio with culture medium in the presence of polybrene (8 µg/ml). After 12 h, the medium was replaced with fresh medium, and cells were selected with G418 (2 µg/ml) (Sigma cat. A1720) for 3 days. After antibiotic selection, cells were passaged and transduced with sh*Luc* or sh*Risp* lentivirus and selected with puromycin (1 µg/ml). DsRed^+^ cells were mixed with blank cells (blank vector) in a 1:1 ratio and passaged for P1–P3 to evaluate the cell competition. From each passage, cells were digested, centrifuged and collected in culture media and subjected to BD6 flow cytometry using the PE-A channel. The percentage of DsRed^+^ cells was determined against the blank cells.

### mtDNA sequencing

For mtDNA analysis, total DNA was extracted using a Promega DNA extraction kit (Promega, cat. A1120). Three pairs of primers were used to amplify the regions 1872–6222, 6203–10,627, and 10,622–1871, covering the entire mtDNA genome (Yang et al, 2020b). The primer sequences are listed in the Reagents and tools table. PCR was performed using KOD enzymes (TOYOBO, cat. KMM-101) for 34 cycles. Subsequently, the three amplified sequences were combined and sent for sequencing at GENEWIZ. A double-digest restriction-associated DNA (ddRAD) library was prepared and sequenced on the Illumina HiSeq4000 platform using 150-bp paired-end reads for 3G flux. The quality-filtered reads of mtDNA were aligned to the reference mitochondrial genome of Mus musculus strain C57BL/6 J mitochondrion (GenBank: AY172335.1) using default parameters. All analyses were conducted using Python, and figures were plotted using the Python packages (matplotlib and seaborn).

### Global DNA methylation quantification by dot blot

Cultured cells were washed with DPBS, and at least 2 million cells were collected for DNA extraction. DNA was extracted using a Promega DNA extraction kit. About 1 µg of DNA was diluted in 50 µl of H_2_O, denatured at 95 °C for 10 min, and cooled on ice for 10 min. The denatured DNA was subjected to serial dilution (1, 0.5, 0.25, and 0.125 µg/50 µl). A nylon transfer membrane (0.45 µm) was placed on the dot blot machine. First, 200 µl of H_2_O was added to each well. Next, 50 µl of the diluted DNA solutions were added to each well. The apparatus operated continuously throughout the procedure to ensure complete permeation of all liquids (water or DNA solution) through the membrane. The membrane was cross-linked to the DNA using an ultraviolet lamp for 2 min, blocked with 5% low-fat milk for 1 h at room temperature with gentle shaking, and washed with PBST (0.1% Tween 20 in DPBS v/v) five times (5 min each on a shaker incubator). The membrane was then incubated with a primary antibody for 5mC (Cell signaling, cat. D3S2Z) for at least 2 h in 5% BSA at room temperature or overnight at 4 °C. After washing five times, the membrane was incubated with a secondary antibody in 5% BSA for 1 h. The membrane was washed and incubated in exposure solution Western Bright ECL for 5 min and visualized using the FUSION SOLO 4 M machine (Vilber Lourmat). Methylene blue (0.02%) staining was used as an internal reference for loading the DNA sample. 5mC quantification was performed using ImageJ software (NIH).

### DNA double-strand breaks measurement by neutral comet assay

Growing cells were digested into single cells, and 0.3 million cells/ml were suspended in precooled DPBS. Cells were mixed with 0.8% low melting agarose gel at a ratio of 1:10 (v/v) and incubated at 37 °C. Cells were spread on precoated (1% agarose gel) glass slides and placed at 4 °C for 30 min. The slides were then transferred into 1x neutral lysis buffer (2.5 M NaCl, 100 mM Na_2_EDTA, 10 mM Tris, pH 9.5, and 1% Triton X-100) for 30 min at room temperature. After lysis, slides were washed twice with H_2_O (5 min each) and incubated in 1x precooled neutral electrophoresis buffer (300 mM sodium acetate, 100 mM Tris, pH 8.3) for 1 h at 4 °C. Electrophoresis was conducted at 30 V, 85 mA for 30 min at 4 °C. The slides were washed three times with H_2_O, fixed in 70% ethanol for 10 min, and dried at room temperature. The slides were then incubated in 2 ml SYBR Gold (Invitrogen, cat. S11494) (0.003% SYBR Gold nucleic acid gel stain in DPBS) for 1 h in the dark. Slides were gently washed with H_2_O, dried at room temperature, or stored at 4 °C overnight. After 24 h, images were captured using a fluorescence microscope, and the olive tail moment was calculated using Comet Score 2.0 software. At least 100 cells were analyzed for each condition.

### Karyotyping

After ten passages of mESCs in 2iL, 1 million cells were seeded in a 10-cm dish for 2.5 days in SL medium (DMEM with high glucose, supplemented with 15% FBS, 1% GlutaMax, 1% NEAA, 1% sodium pyruvate, 0.5% P/S, 0.1 mM β-mercaptoethanol, and 1000 U/ml mLIF). Before lysis, cells were cultured in fresh medium containing 0.5 μg/ml nocodazole (Selleck, cat. S2775) for 2 h to accumulate cells in metaphase in a 37 °C CO_2_ incubator. Cells were washed with DPBS, trypsinized, and centrifuged at 1200 rpm for 5 min. Cells were then suspended in 2 ml of preheated 75 mM KCl, and the volume was adjusted to 7 ml. The suspended cells were incubated in a 37 °C water bath for 30 min. After centrifugation at 1200 rpm for 5 min, the supernatant was removed, and 2 ml of precooled fixative solution (glacial acetic acid and methanol, 3:1 v/v) was added. The volume was adjusted to 7 ml, and the cells were incubated for 40 min in a 37 °C water bath. After centrifugation at 1200 rpm, the fixative solution was partially removed. The cells were suspended and mounted on precooled glass slides from 30 cm. The slides were dried at 65 °C for 5 min, stained with 0.1 ng/ml DAPI (Vector Laboratories, cat. H-1800-10) for 5 min, washed with H_2_O, and dried. The chromosome number of the separated nucleus was counted under an upright fluorescence microscope. For each sample, at least 20 randomly selected metaphase spreads were analyzed.

### Statistical analysis

It was performed using a two-tailed unpaired Student’s *t*-test (*p < 0.05; **p < 0.01; ***p < 0.001; ****p < 0.0001). Values are shown as the mean ± standard deviation of mean (SD) and were analyzed with GraphPad Prism 8 from multiple independent experiments. Detailed *n* values for each experiment in the figures are stated in the corresponding legends.

## Supplementary information

Table EV1

Peer Review File

Source data Fig. 1

Source data Fig. 2

Source data Fig. 3

Source data Fig. 4

Expanded View Figures

## Data Availability

All data supporting the current study are available within the paper and Supplementary Information. No large-scale data amenable to data repository deposition were generated in this study. The source data of this paper are collected in the following database record: biostudies:S-SCDT-10_1038-S44318-026-00784-2.
