# Supplementary figures and images for: Mitochondrial NADH-redox inflexibility constrains genomic and epigenetic stability in pluripotent stem cells
Source: EMBO J. 2026 May 27;45(13):4417–46. doi: 10.1038/s44318-026-00784-2 (PMC13324003; doi:10.1038/s44318-026-00784-2)

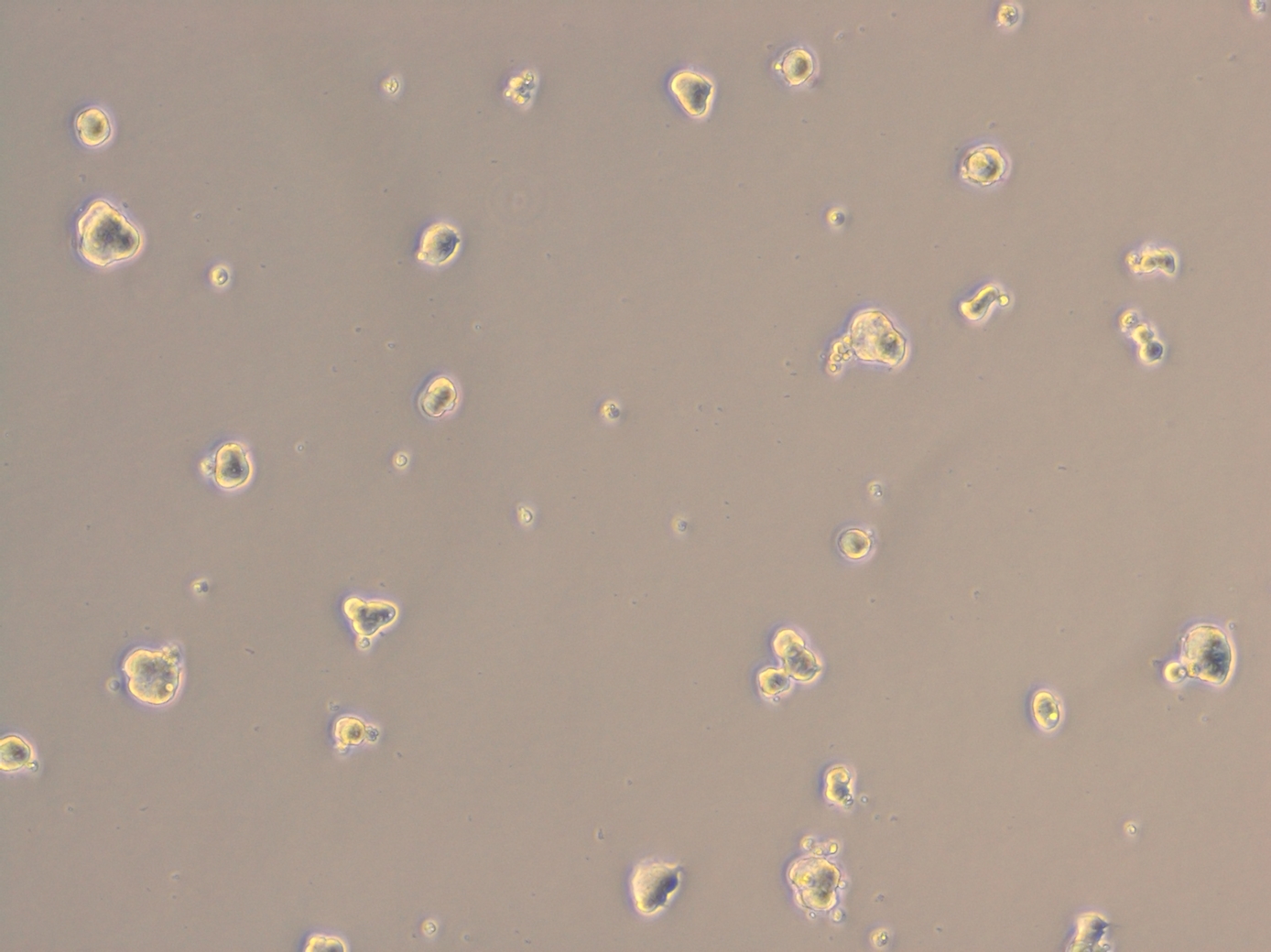

Supplement: Supplementary file 3 — Source data Fig. 1 [file 44318_2026_784_MOESM3_ESM.zip › Figure 1/A/Ant+Asp .jpg]

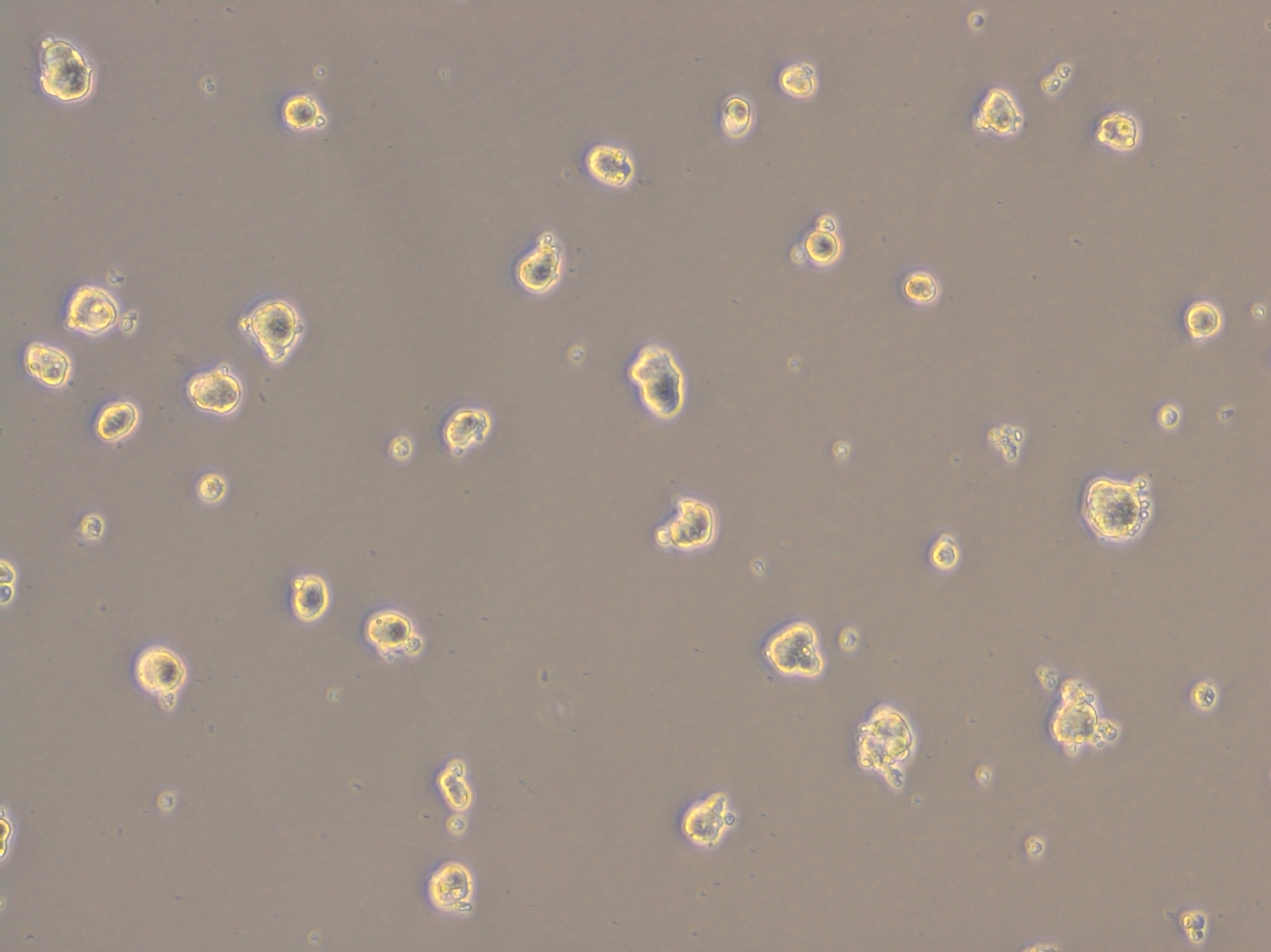

Supplement: Supplementary file 3 — Source data Fig. 1 [file 44318_2026_784_MOESM3_ESM.zip › Figure 1/A/Ant-Asp .jpg]

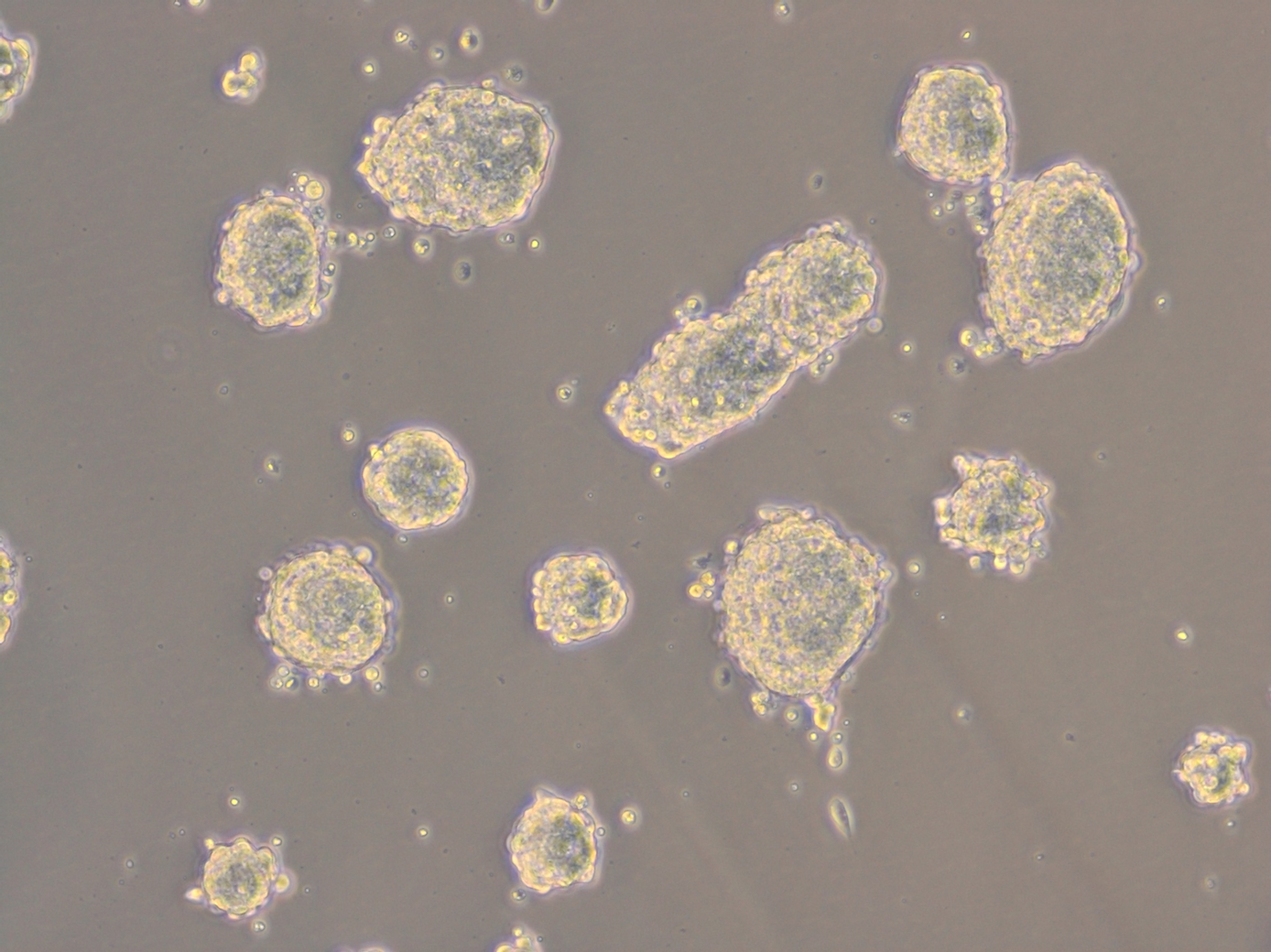

Supplement: Supplementary file 3 — Source data Fig. 1 [file 44318_2026_784_MOESM3_ESM.zip › Figure 1/A/Ctrl+Asp .jpg]

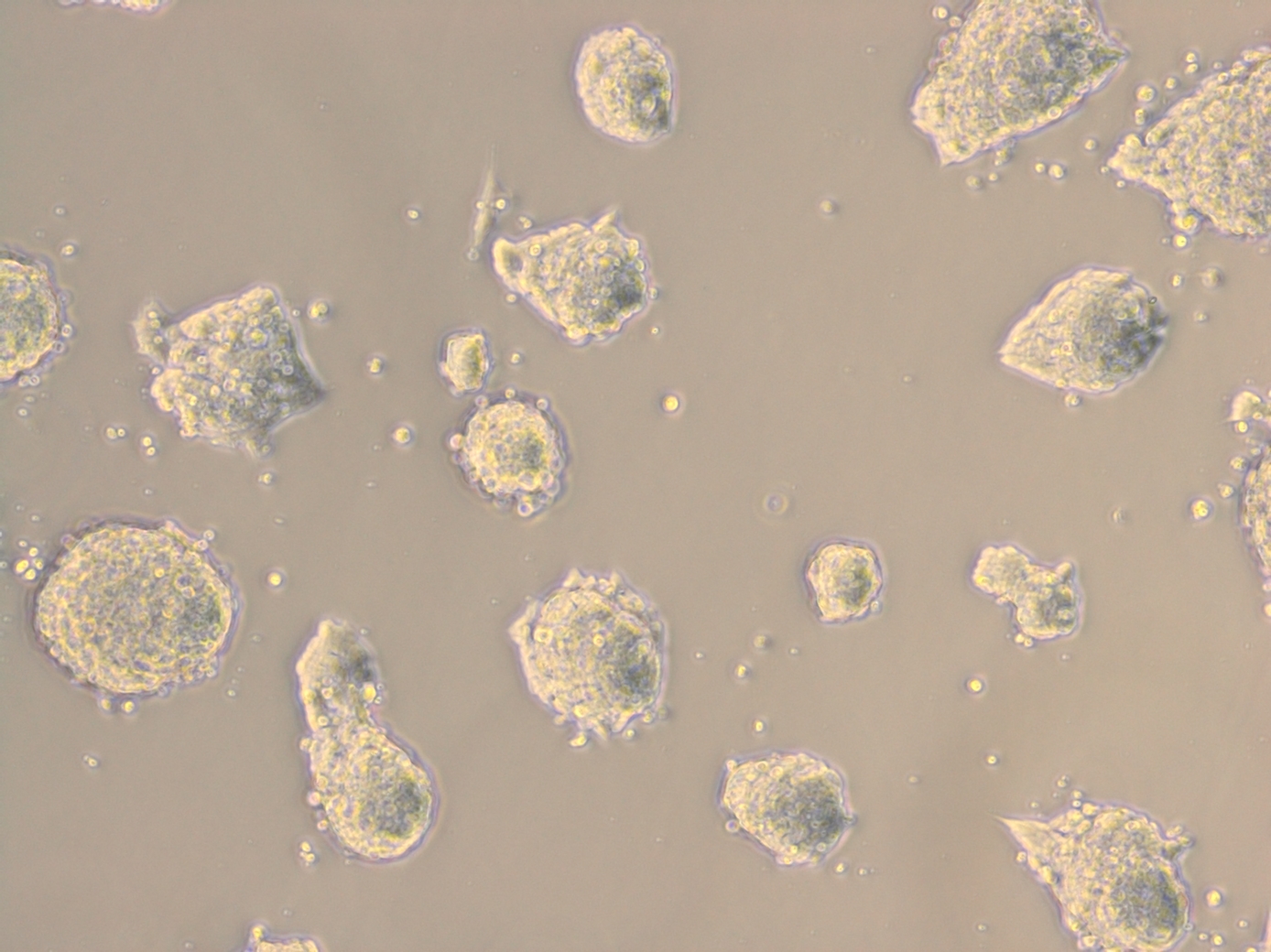

Supplement: Supplementary file 3 — Source data Fig. 1 [file 44318_2026_784_MOESM3_ESM.zip › Figure 1/A/Ctrl-Asp .jpg]

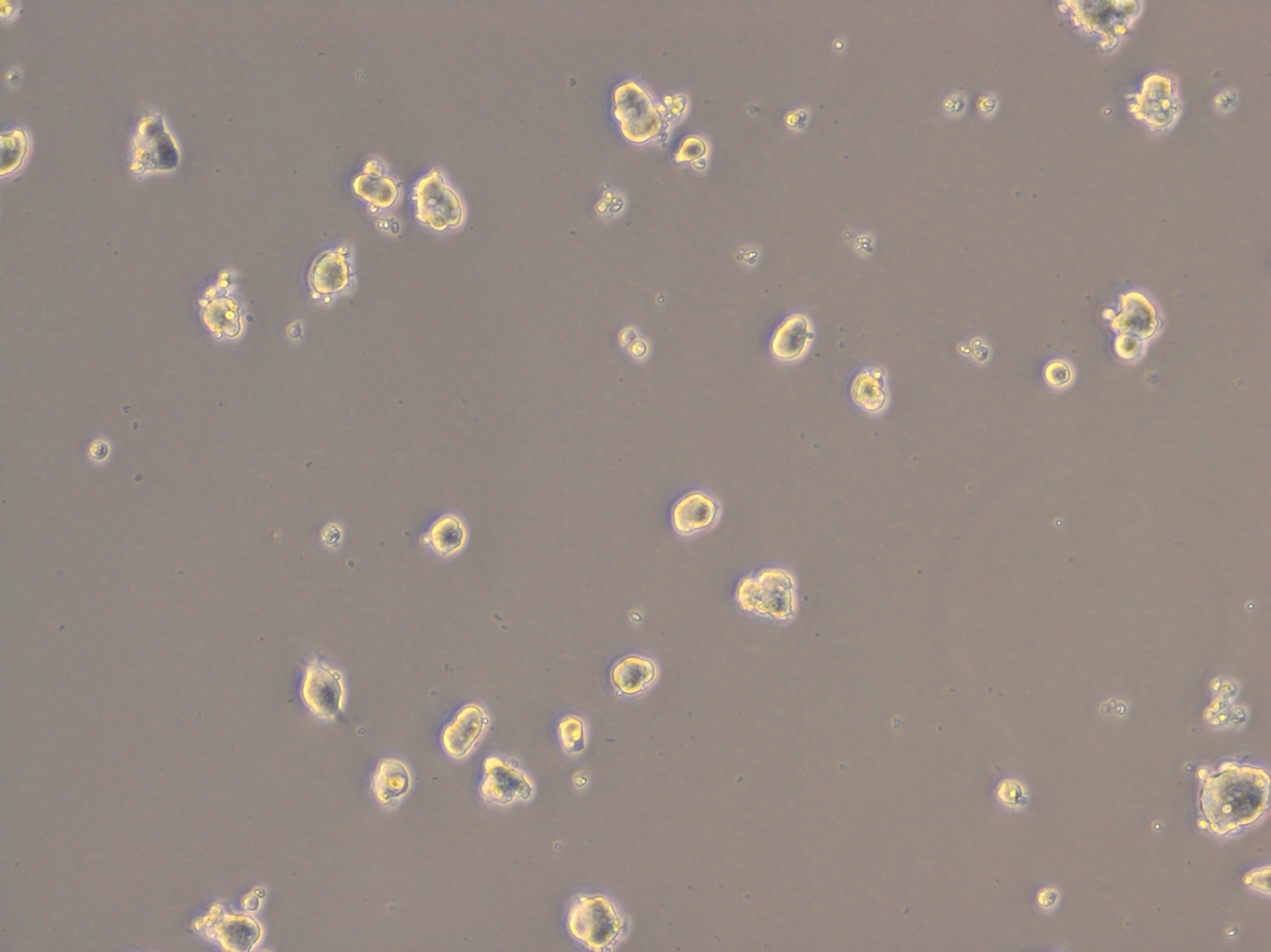

Supplement: Supplementary file 3 — Source data Fig. 1 [file 44318_2026_784_MOESM3_ESM.zip › Figure 1/A/Rot+Asp .jpg]

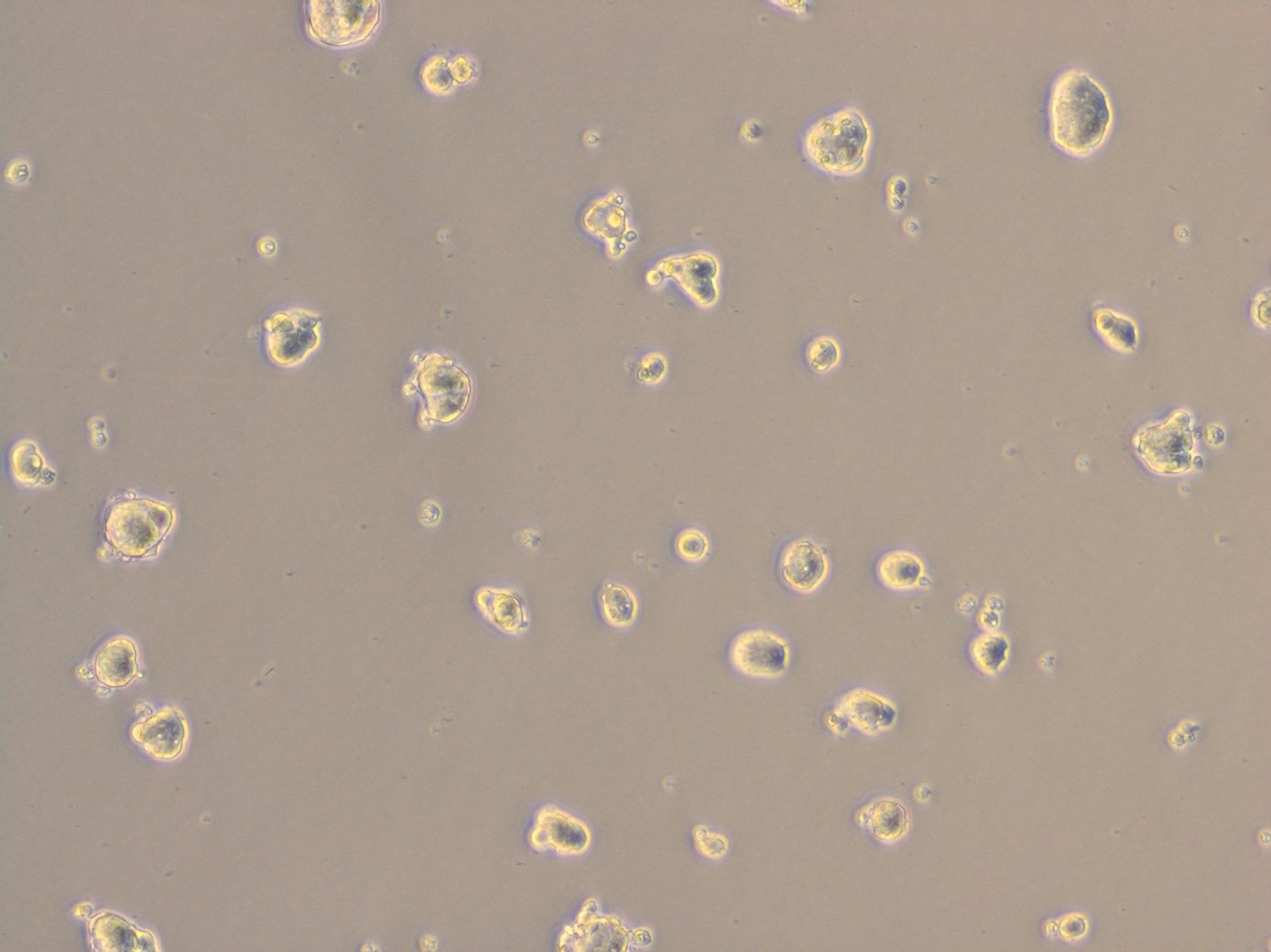

Supplement: Supplementary file 3 — Source data Fig. 1 [file 44318_2026_784_MOESM3_ESM.zip › Figure 1/A/Rot-Asp .jpg]

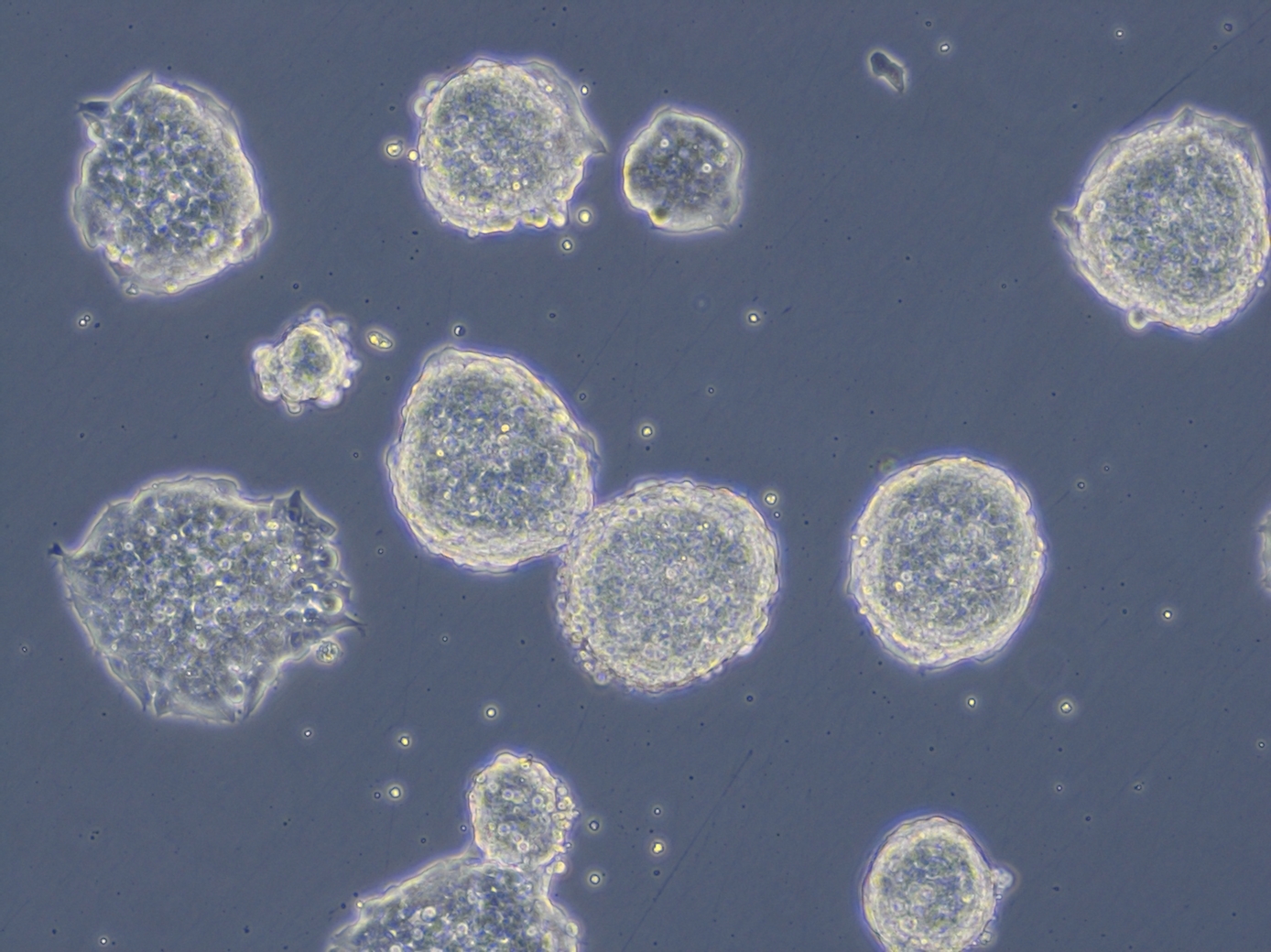

Supplement: Supplementary file 3 — Source data Fig. 1 [file 44318_2026_784_MOESM3_ESM.zip › Figure 1/G/+Ndi1+Rot.jpg]

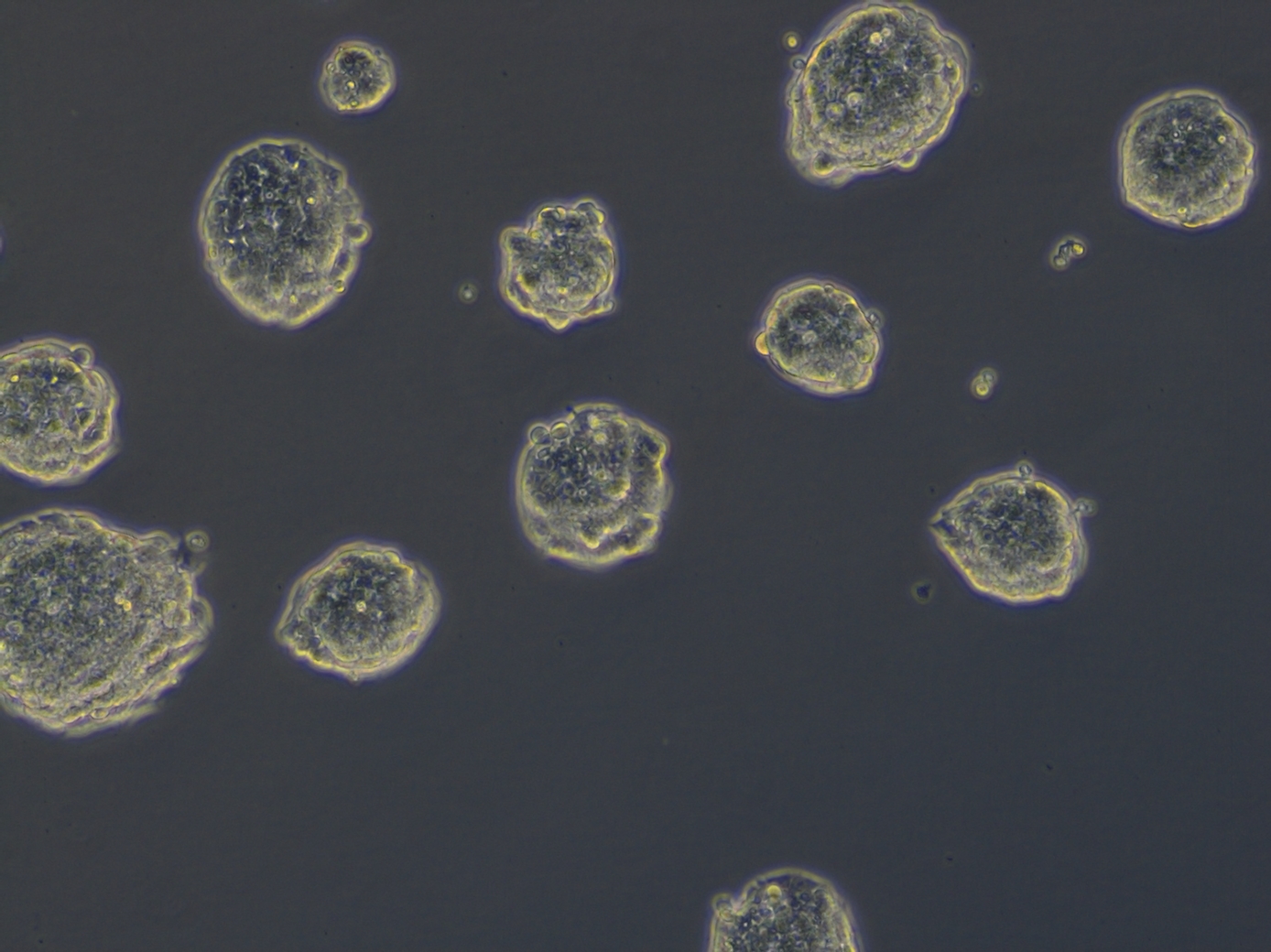

Supplement: Supplementary file 3 — Source data Fig. 1 [file 44318_2026_784_MOESM3_ESM.zip › Figure 1/G/+Ndi1-Rot.jpg]

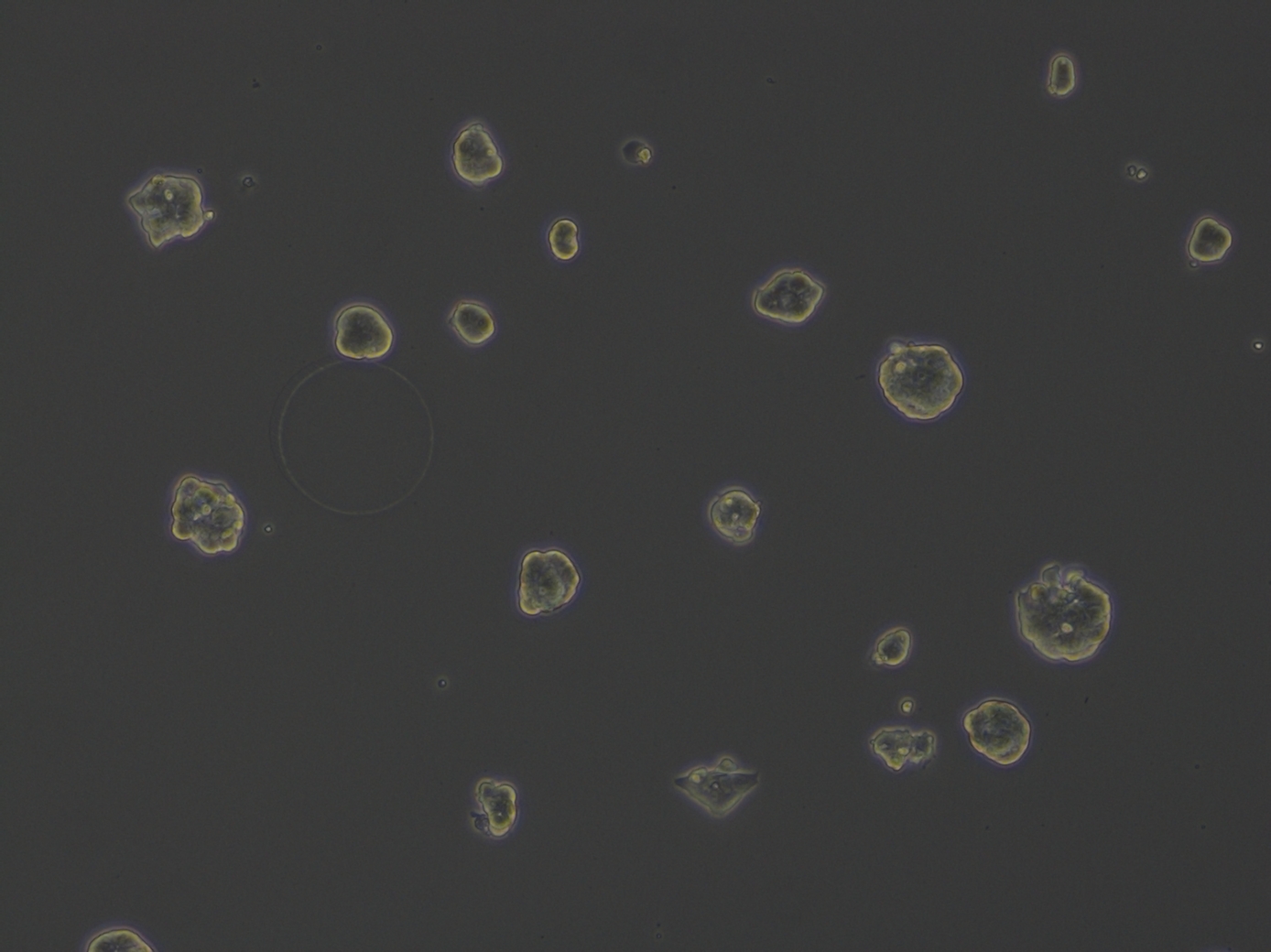

Supplement: Supplementary file 3 — Source data Fig. 1 [file 44318_2026_784_MOESM3_ESM.zip › Figure 1/G/Ctrl+Rot.jpg]

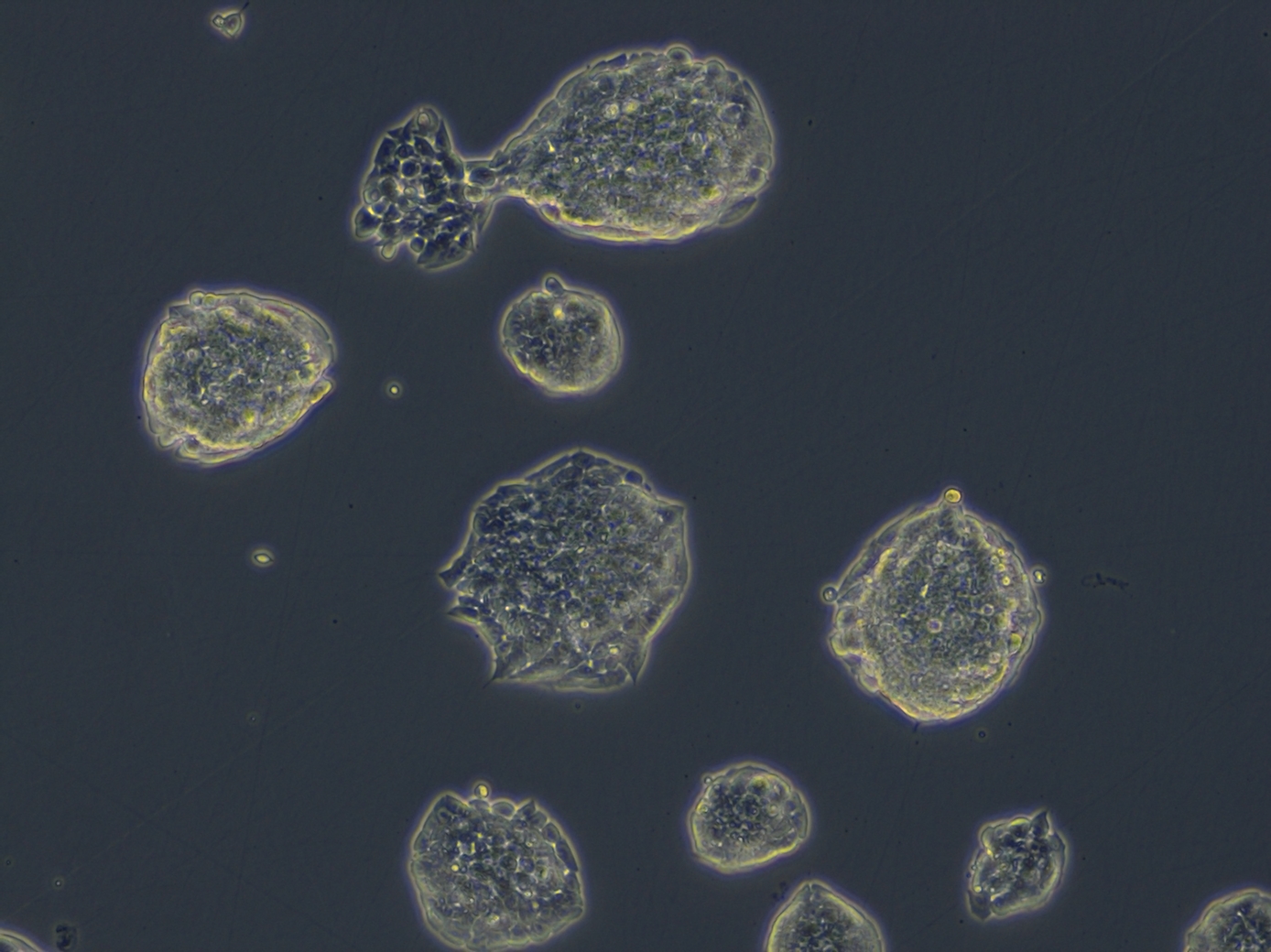

Supplement: Supplementary file 3 — Source data Fig. 1 [file 44318_2026_784_MOESM3_ESM.zip › Figure 1/G/Ctrl-Rot.jpg]

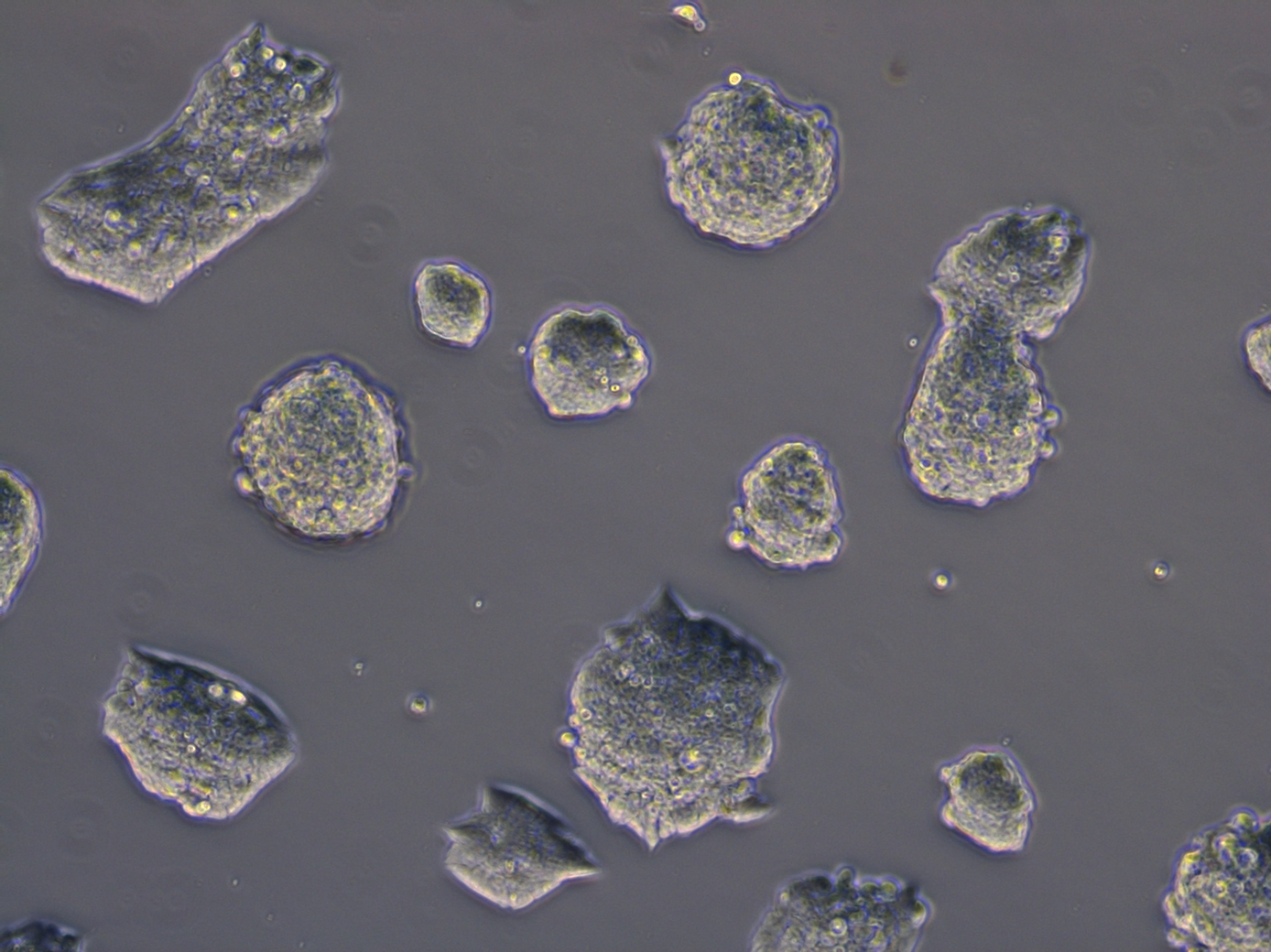

Supplement: Supplementary file 3 — Source data Fig. 1 [file 44318_2026_784_MOESM3_ESM.zip › Figure 1/K/QC1 0uM.tiff]

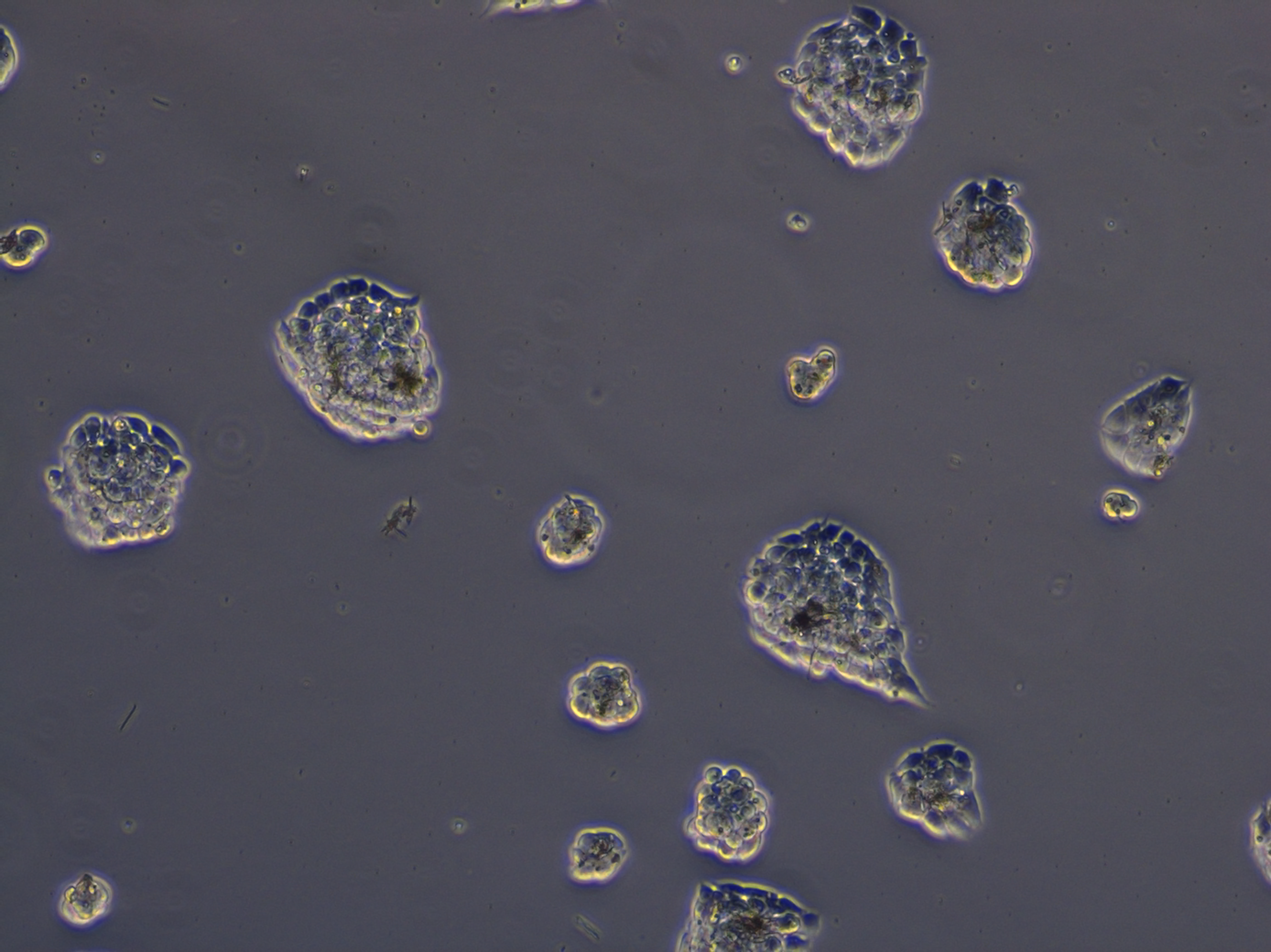

Supplement: Supplementary file 3 — Source data Fig. 1 [file 44318_2026_784_MOESM3_ESM.zip › Figure 1/K/QC1 10uM.tiff]

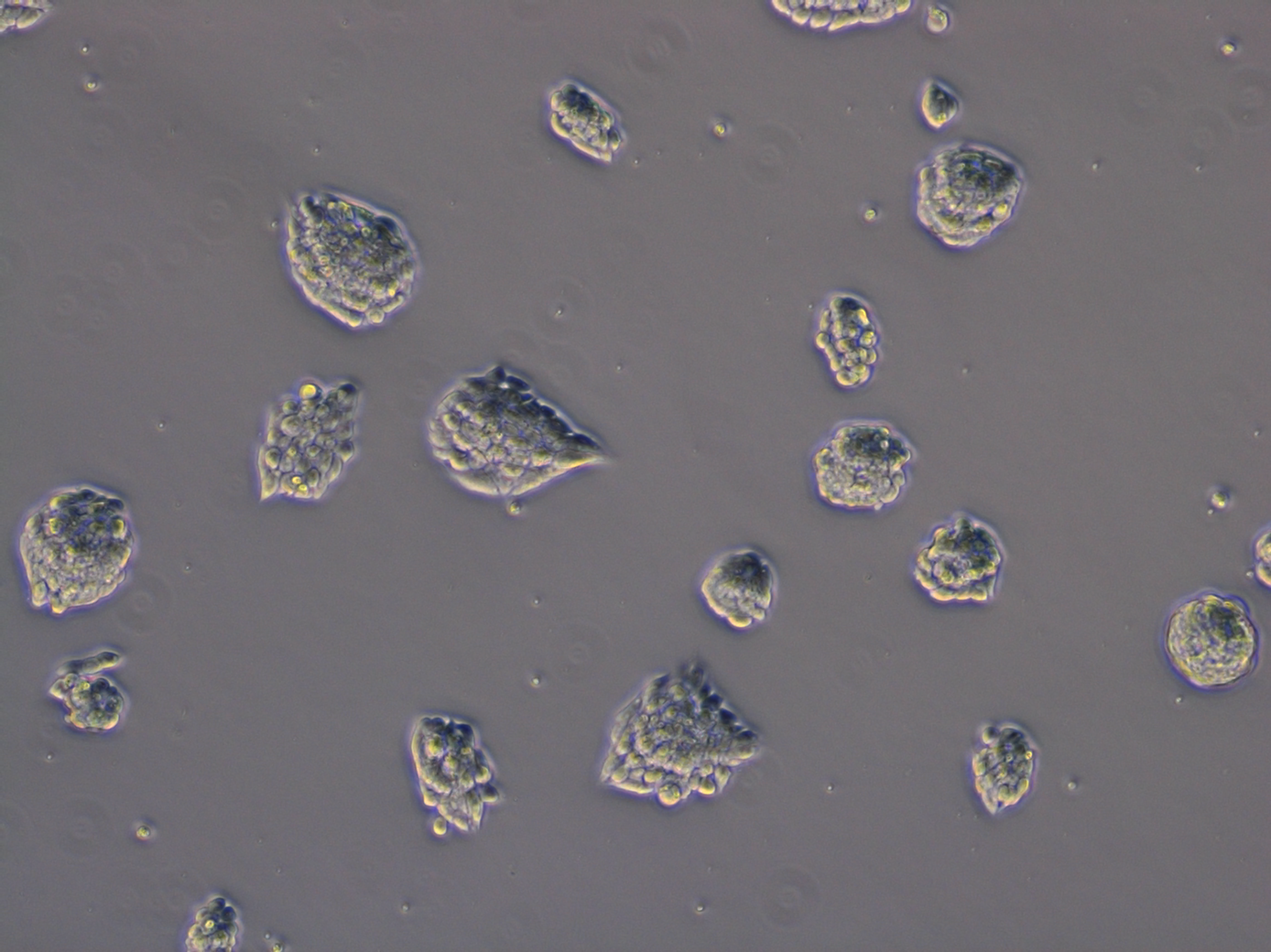

Supplement: Supplementary file 3 — Source data Fig. 1 [file 44318_2026_784_MOESM3_ESM.zip › Figure 1/K/QC1 5uM.tiff]

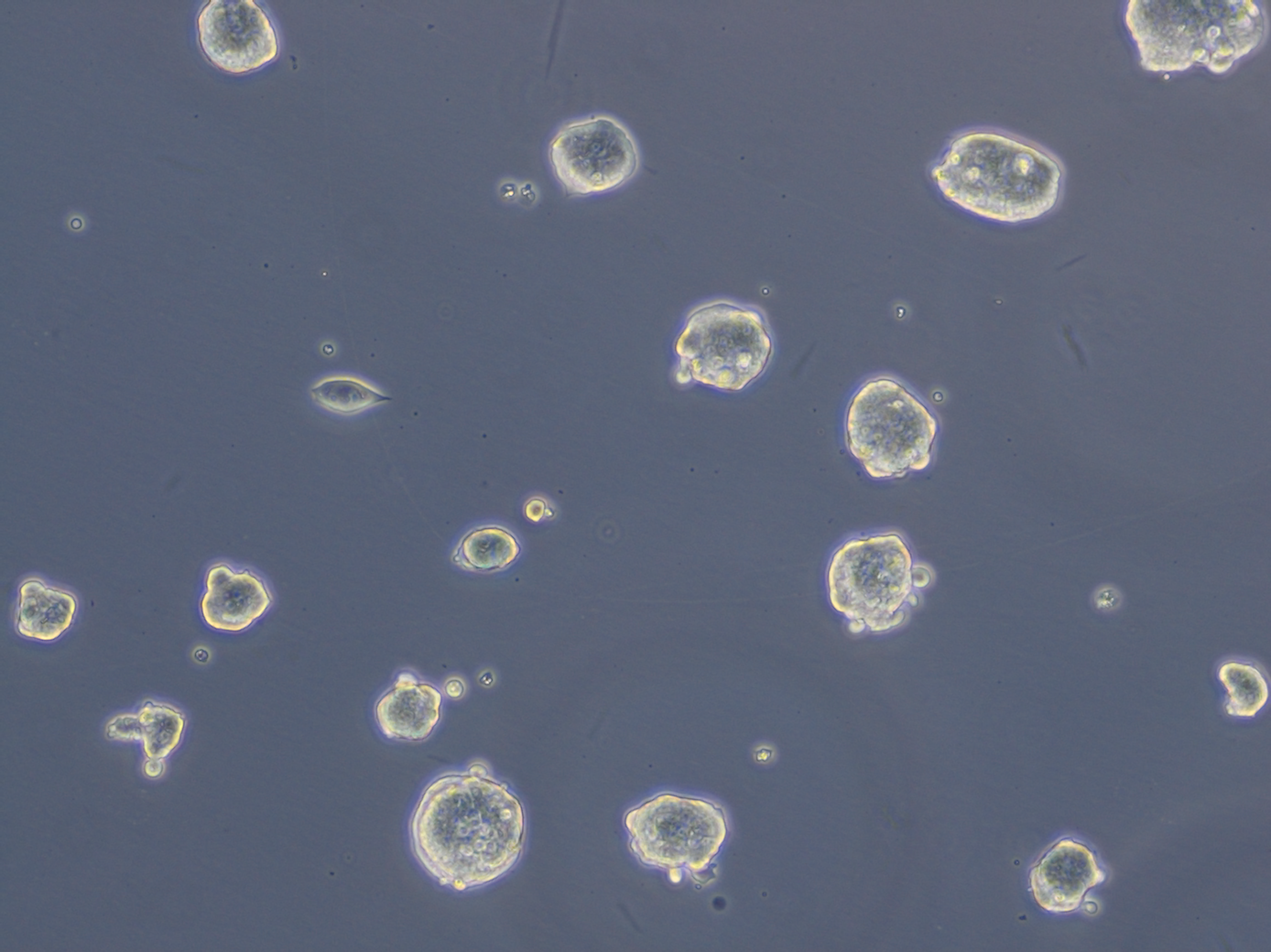

Supplement: Supplementary file 3 — Source data Fig. 1 [file 44318_2026_784_MOESM3_ESM.zip › Figure 1/P/Ant+QC1(5uM).tif]

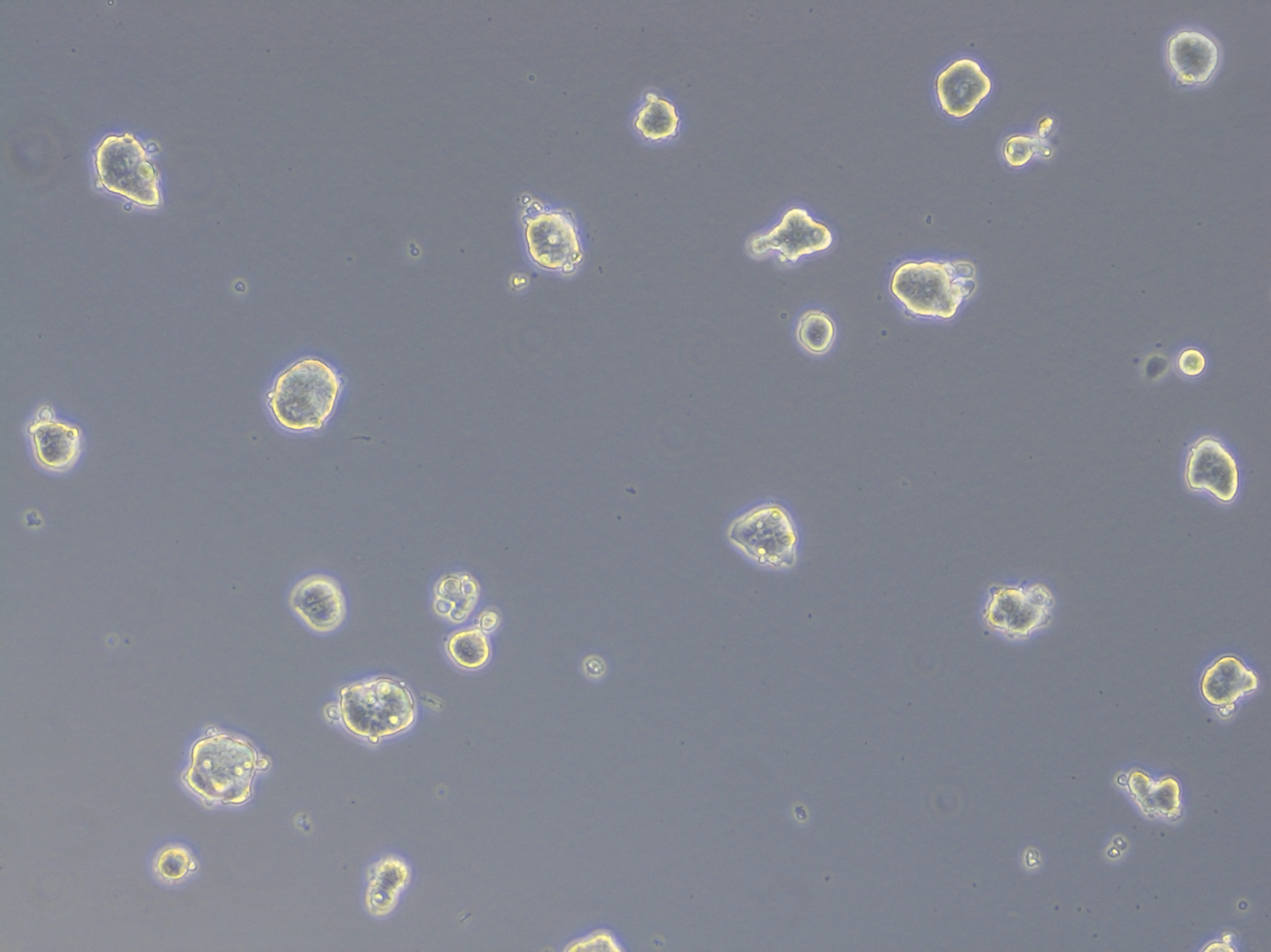

Supplement: Supplementary file 3 — Source data Fig. 1 [file 44318_2026_784_MOESM3_ESM.zip › Figure 1/P/Ant-QC1.tif]

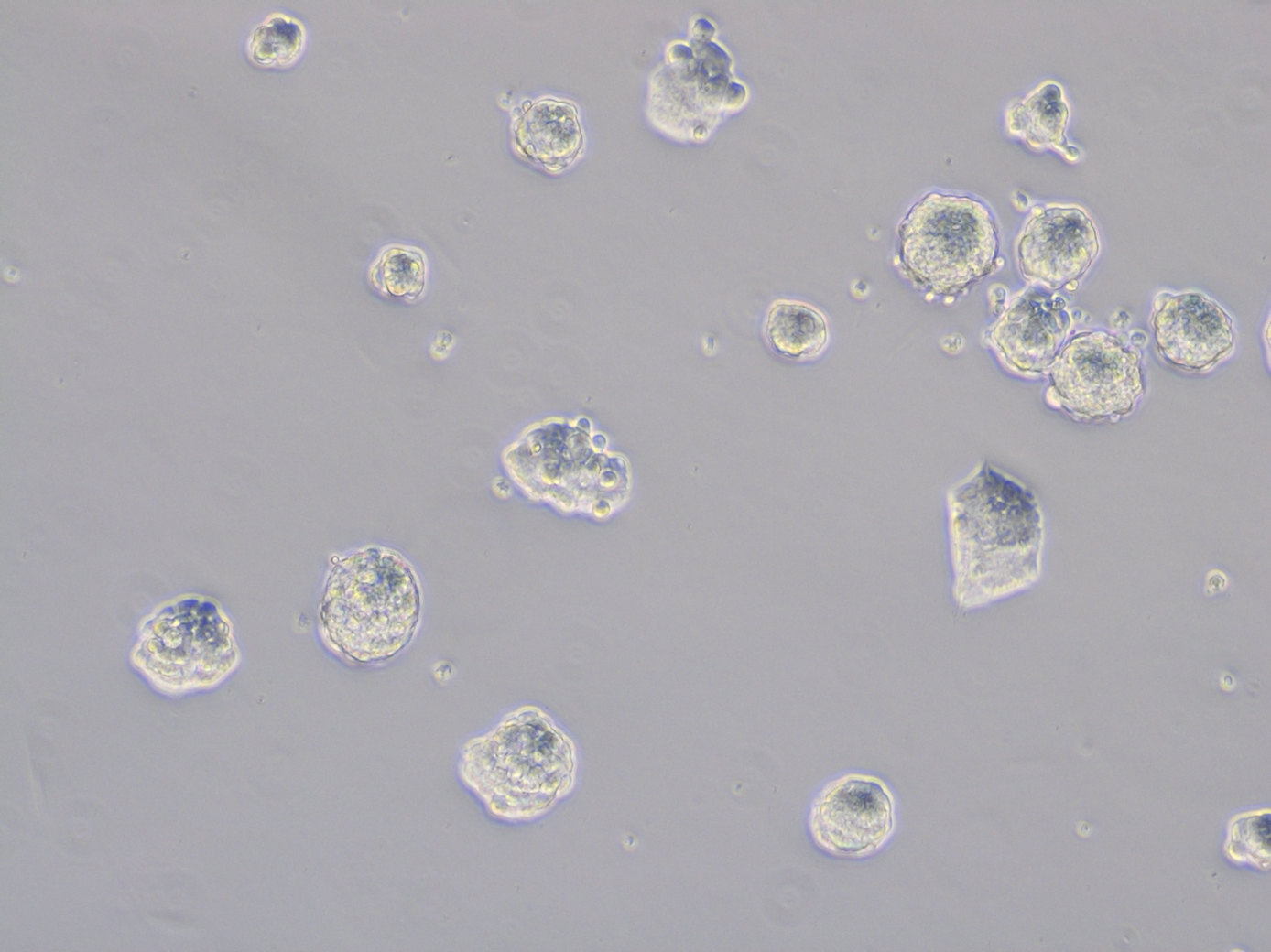

Supplement: Supplementary file 3 — Source data Fig. 1 [file 44318_2026_784_MOESM3_ESM.zip › Figure 1/P/Ctrl+QC1(5uM).tif]

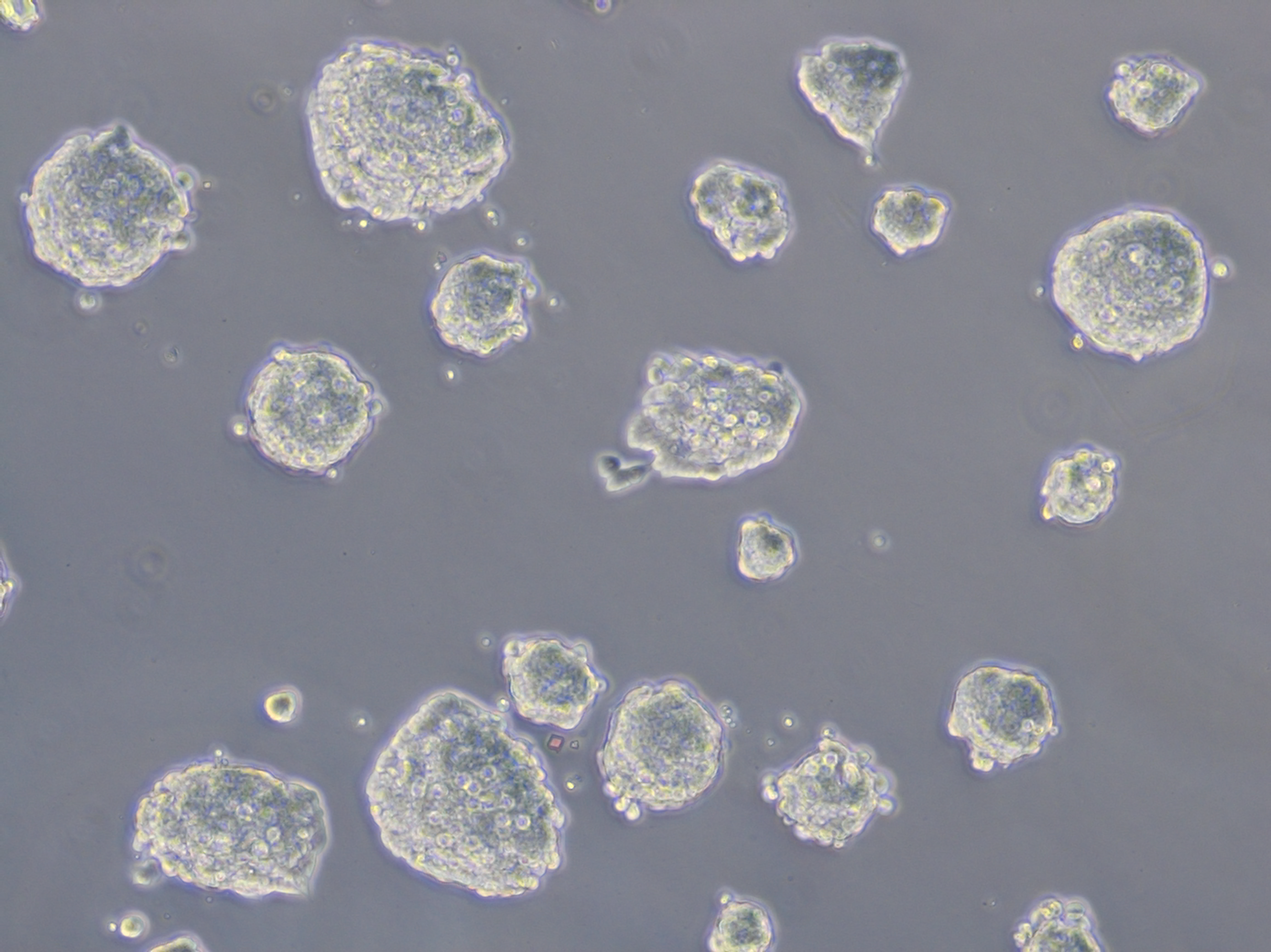

Supplement: Supplementary file 3 — Source data Fig. 1 [file 44318_2026_784_MOESM3_ESM.zip › Figure 1/P/Ctrl-QC1.tif]

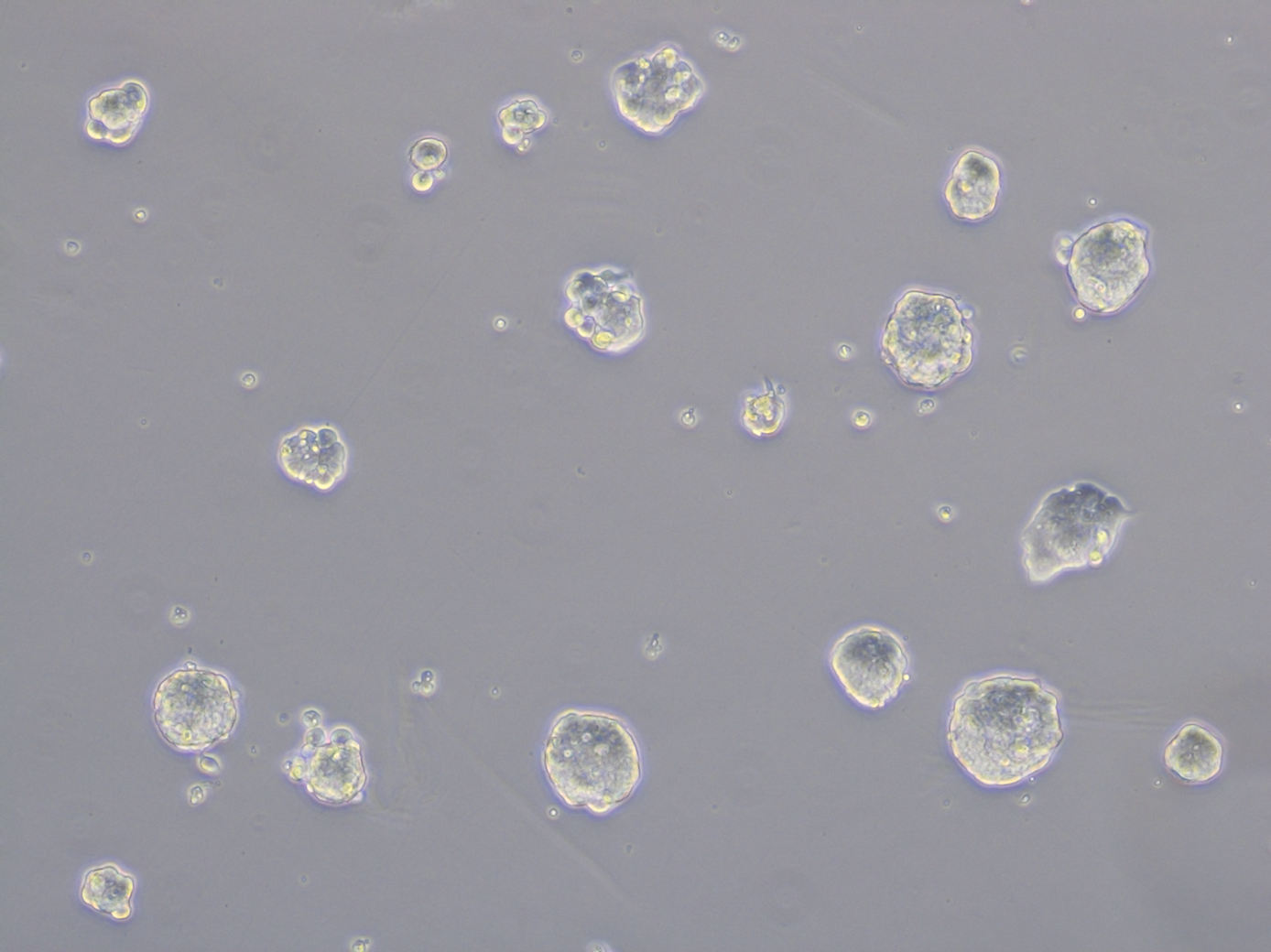

Supplement: Supplementary file 3 — Source data Fig. 1 [file 44318_2026_784_MOESM3_ESM.zip › Figure 1/P/Rot+QC1(5uM).tif]

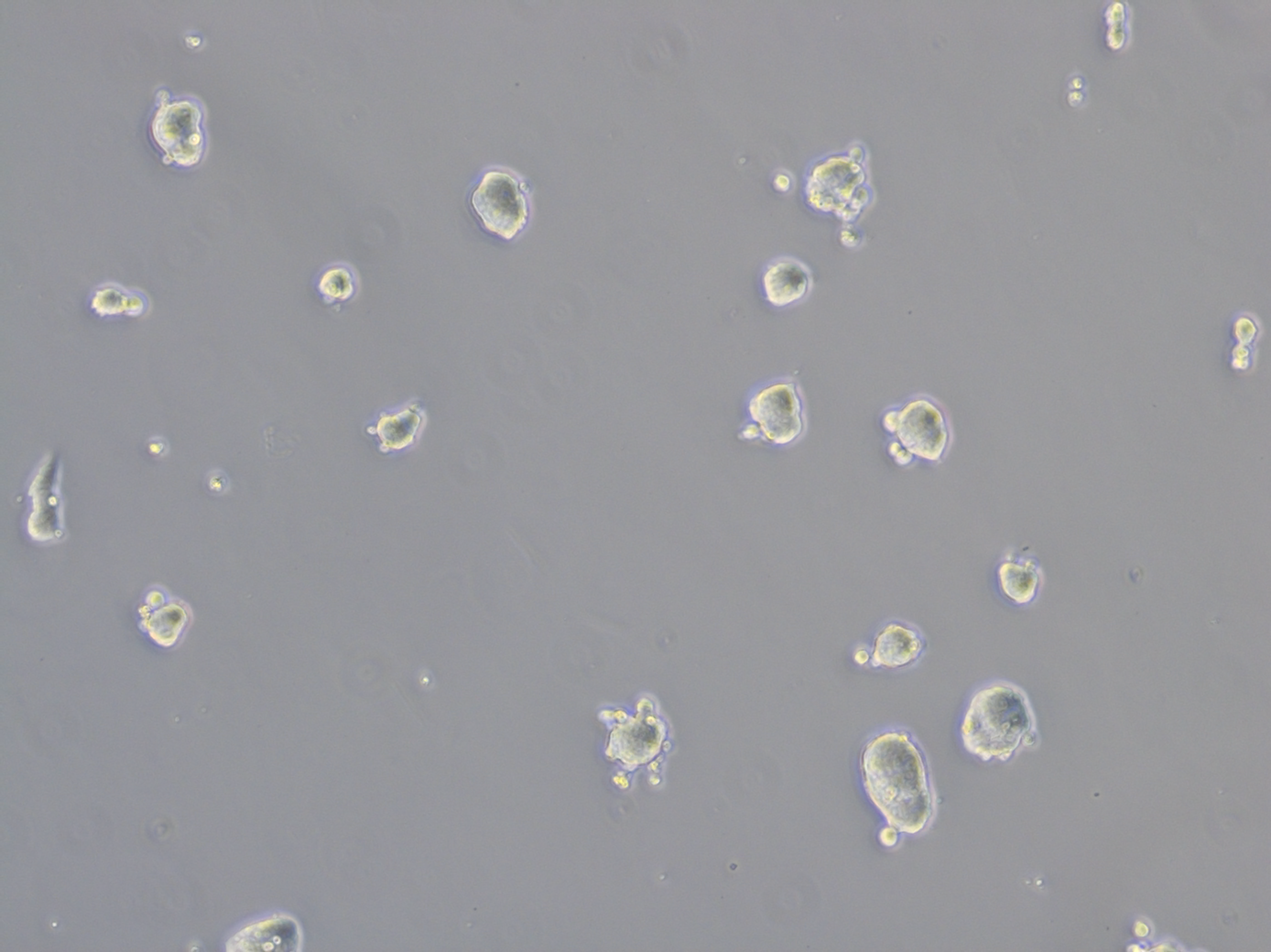

Supplement: Supplementary file 3 — Source data Fig. 1 [file 44318_2026_784_MOESM3_ESM.zip › Figure 1/P/Rot-QC1.tif]

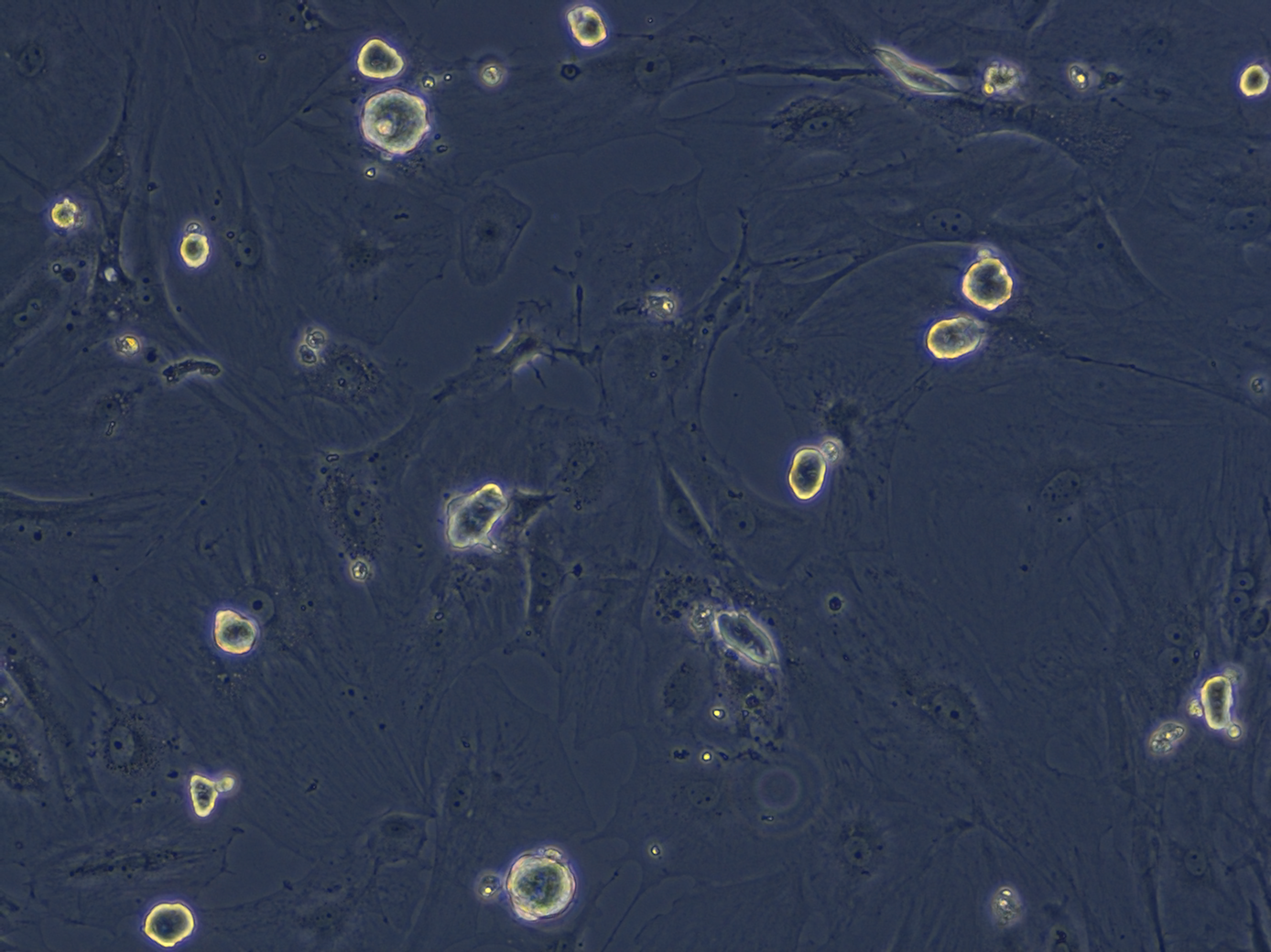

Supplement: Supplementary file 3 — Source data Fig. 1 [file 44318_2026_784_MOESM3_ESM.zip › Figure 1/S/Ant .tiff]

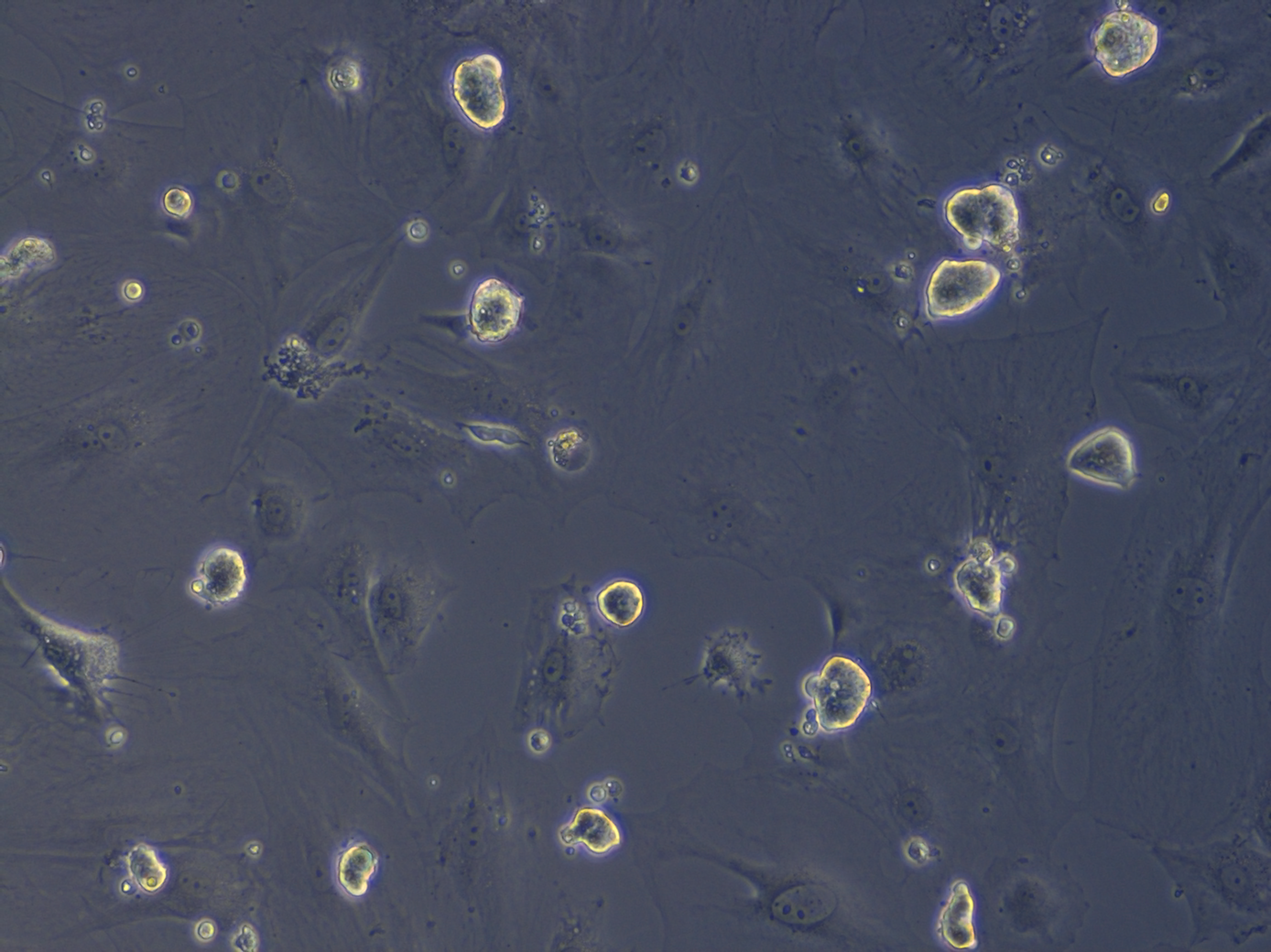

Supplement: Supplementary file 3 — Source data Fig. 1 [file 44318_2026_784_MOESM3_ESM.zip › Figure 1/S/Ant+Pyr.tiff]

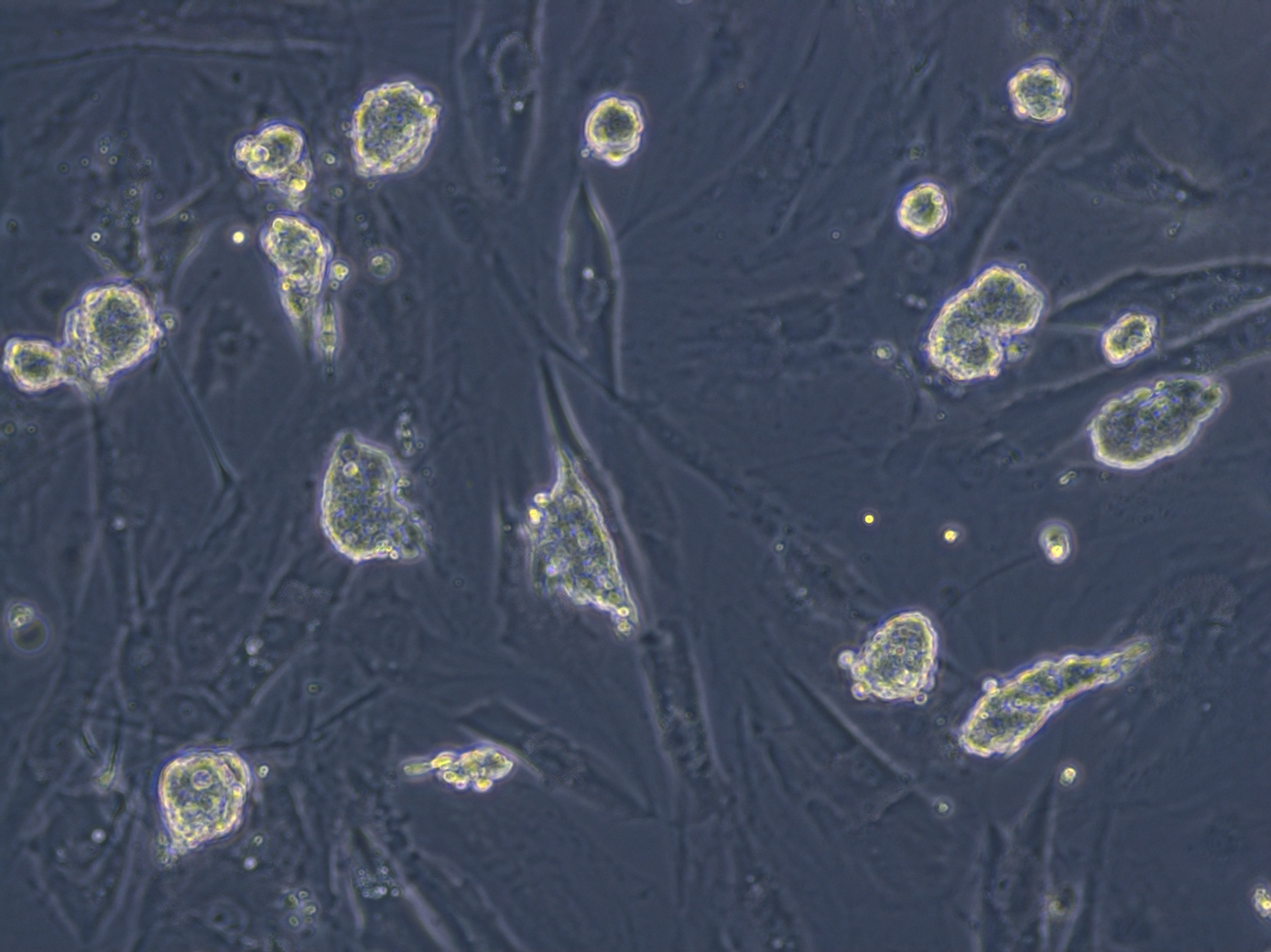

Supplement: Supplementary file 3 — Source data Fig. 1 [file 44318_2026_784_MOESM3_ESM.zip › Figure 1/S/Ant+QC1(5uM)+Pyr.tiff]

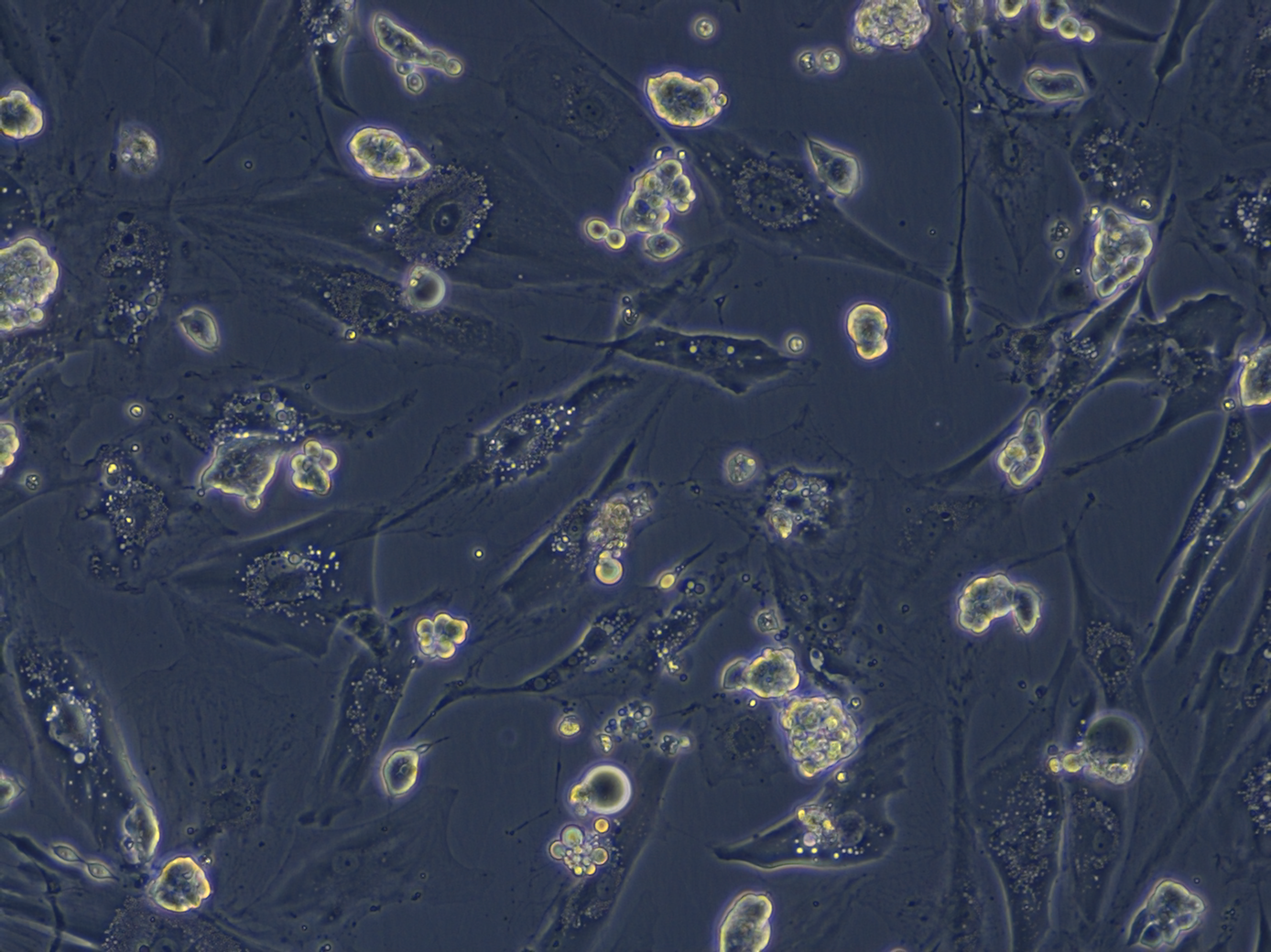

Supplement: Supplementary file 3 — Source data Fig. 1 [file 44318_2026_784_MOESM3_ESM.zip › Figure 1/S/Ant+QC1(5uM).tiff]

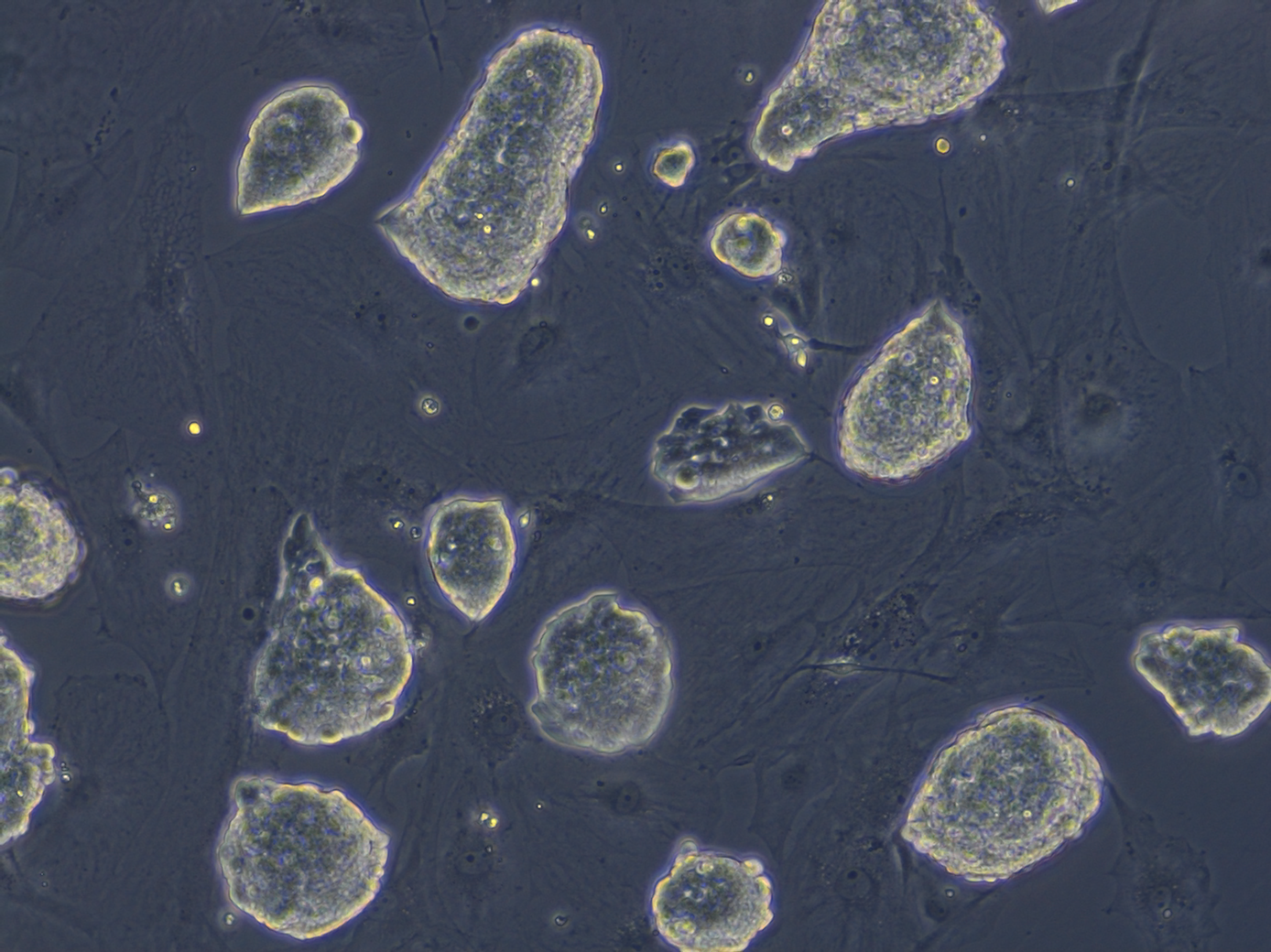

Supplement: Supplementary file 3 — Source data Fig. 1 [file 44318_2026_784_MOESM3_ESM.zip › Figure 1/S/Ctrl+Pyr.tiff]

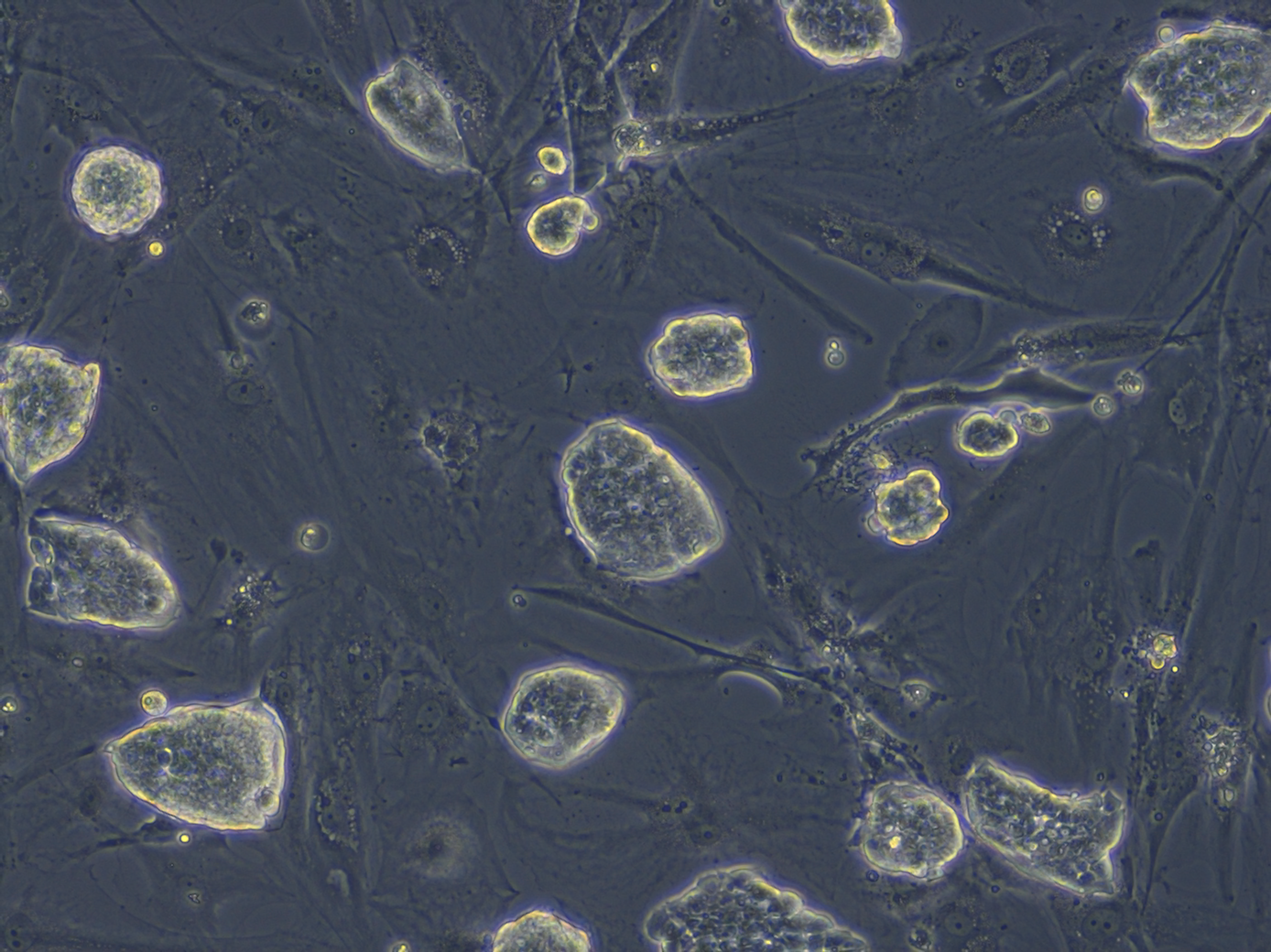

Supplement: Supplementary file 3 — Source data Fig. 1 [file 44318_2026_784_MOESM3_ESM.zip › Figure 1/S/Ctrl+QC1(5uM)+Pyr.tiff]

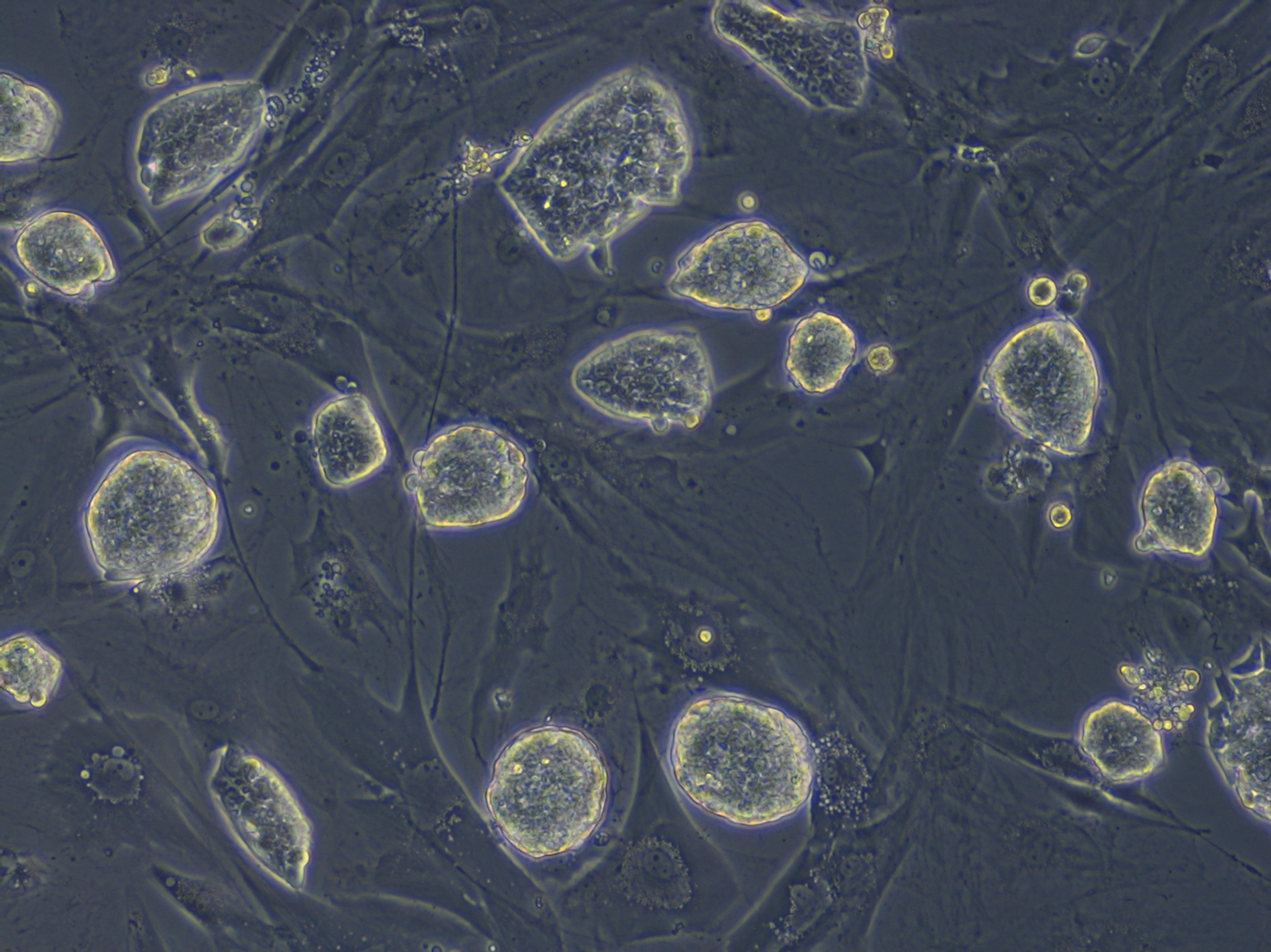

Supplement: Supplementary file 3 — Source data Fig. 1 [file 44318_2026_784_MOESM3_ESM.zip › Figure 1/S/Ctrl+QC1(5uM).tiff]

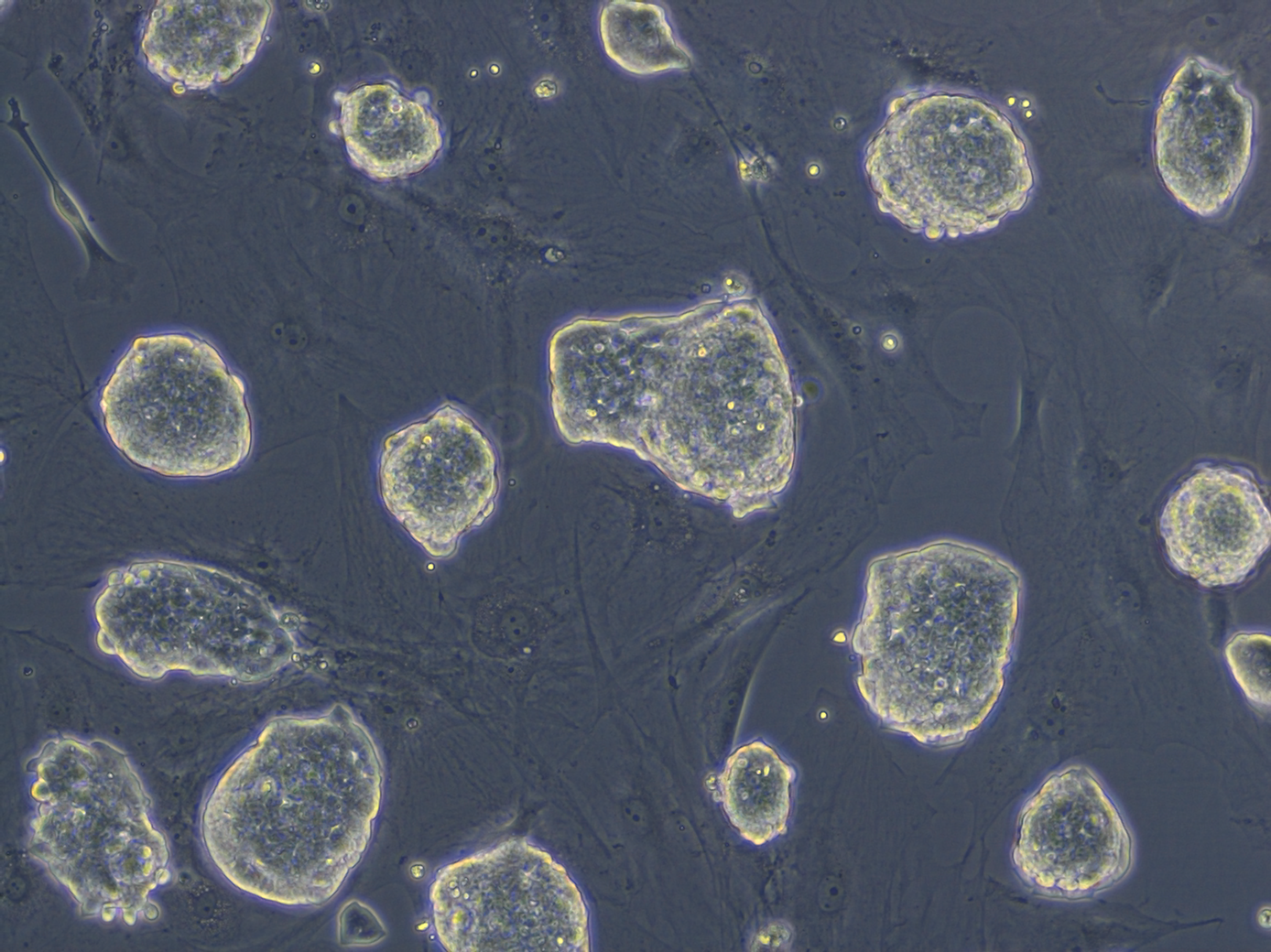

Supplement: Supplementary file 3 — Source data Fig. 1 [file 44318_2026_784_MOESM3_ESM.zip › Figure 1/S/Ctrl.tiff]

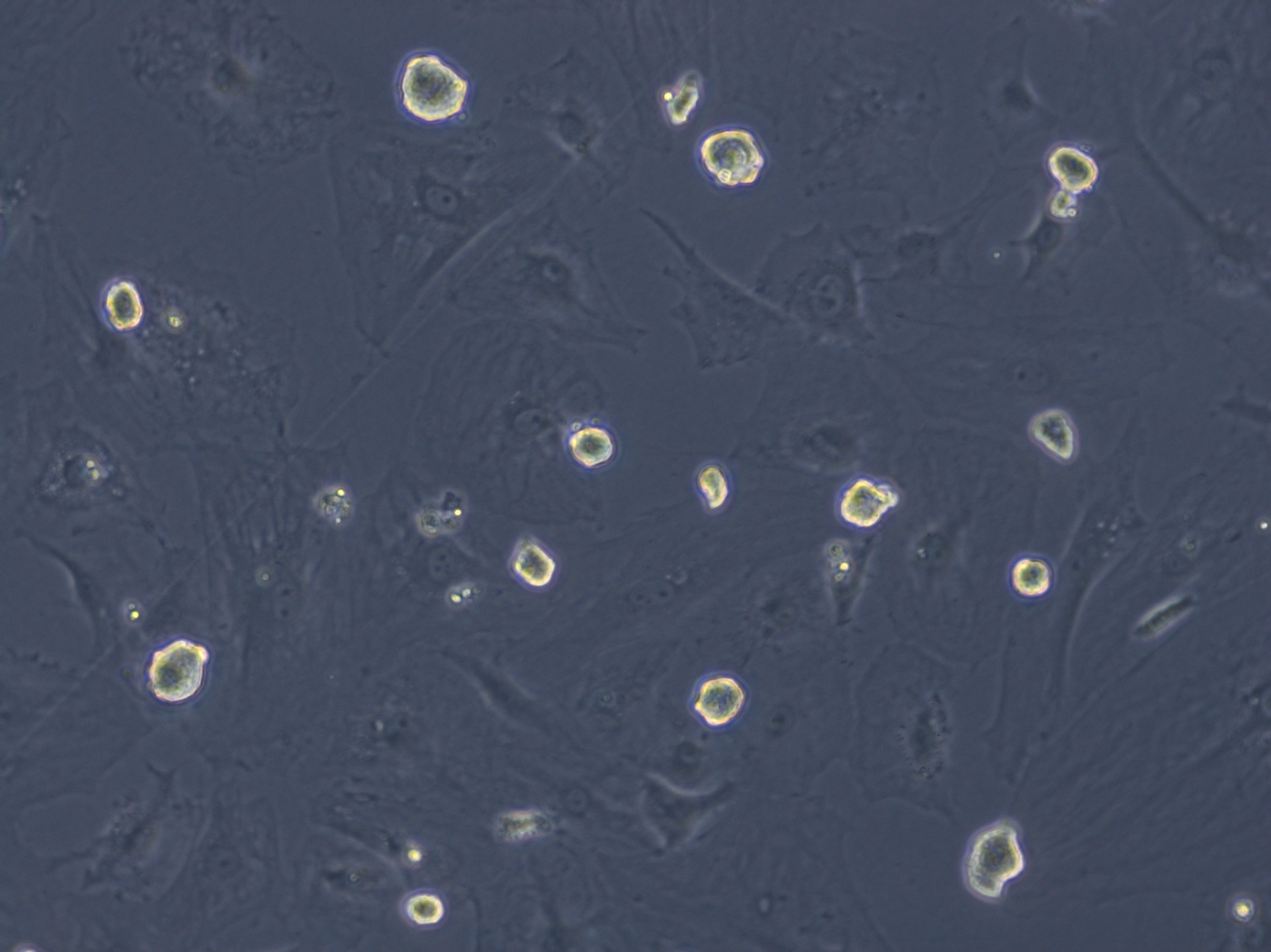

Supplement: Supplementary file 3 — Source data Fig. 1 [file 44318_2026_784_MOESM3_ESM.zip › Figure 1/S/Rot .tiff]

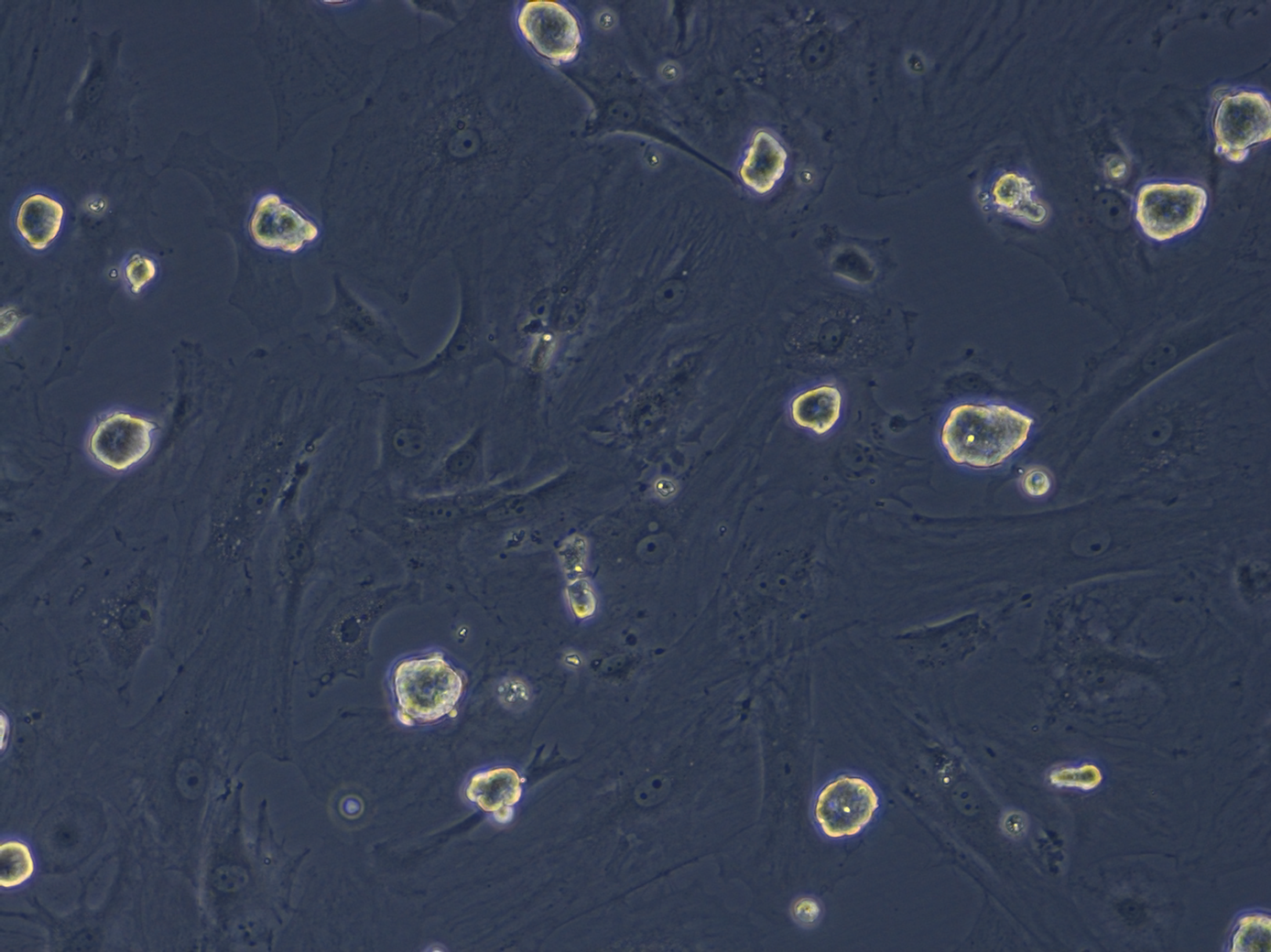

Supplement: Supplementary file 3 — Source data Fig. 1 [file 44318_2026_784_MOESM3_ESM.zip › Figure 1/S/Rot+Pyr .tiff]

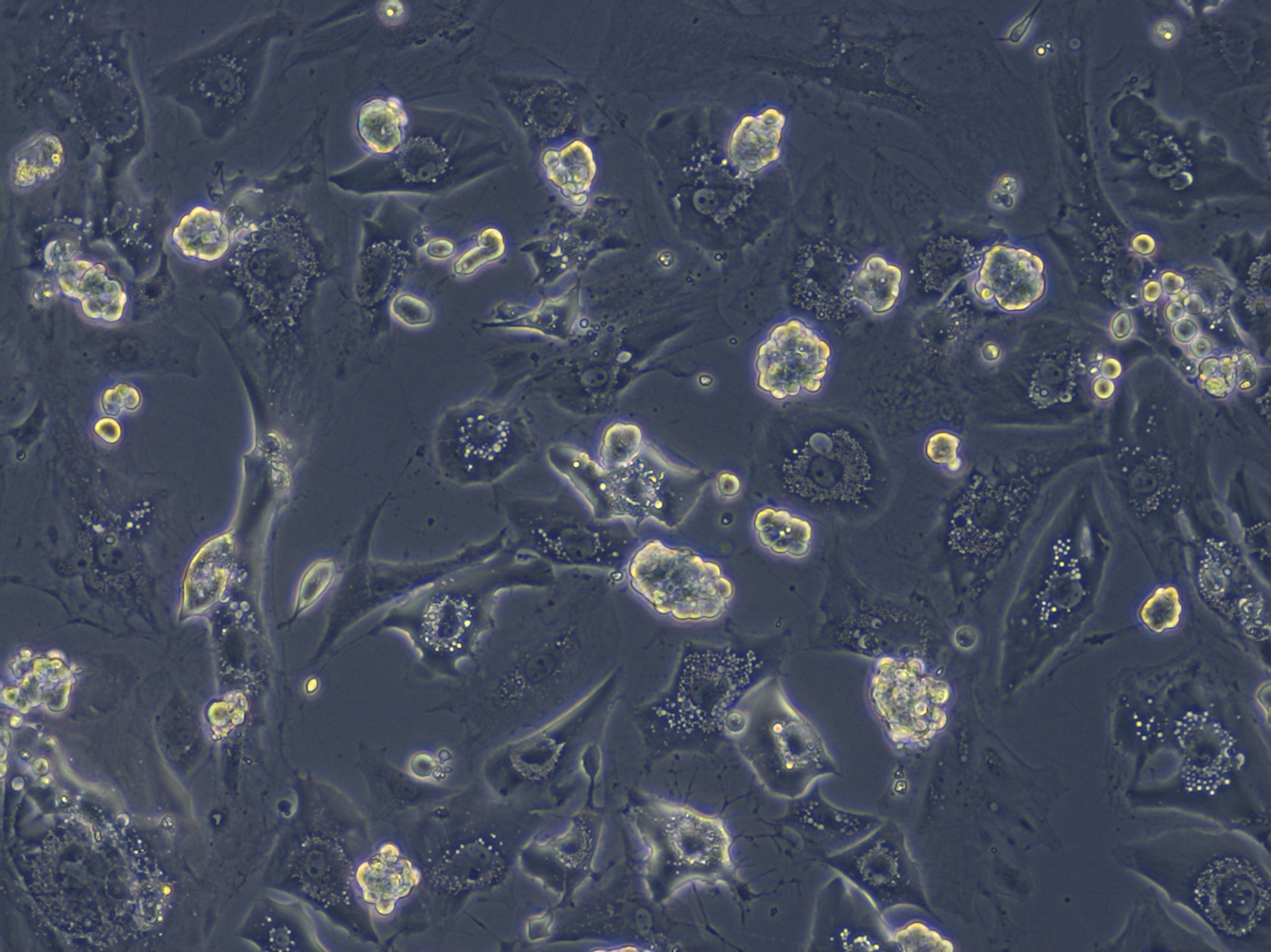

Supplement: Supplementary file 3 — Source data Fig. 1 [file 44318_2026_784_MOESM3_ESM.zip › Figure 1/S/Rot+QC1(5uM) .tiff]

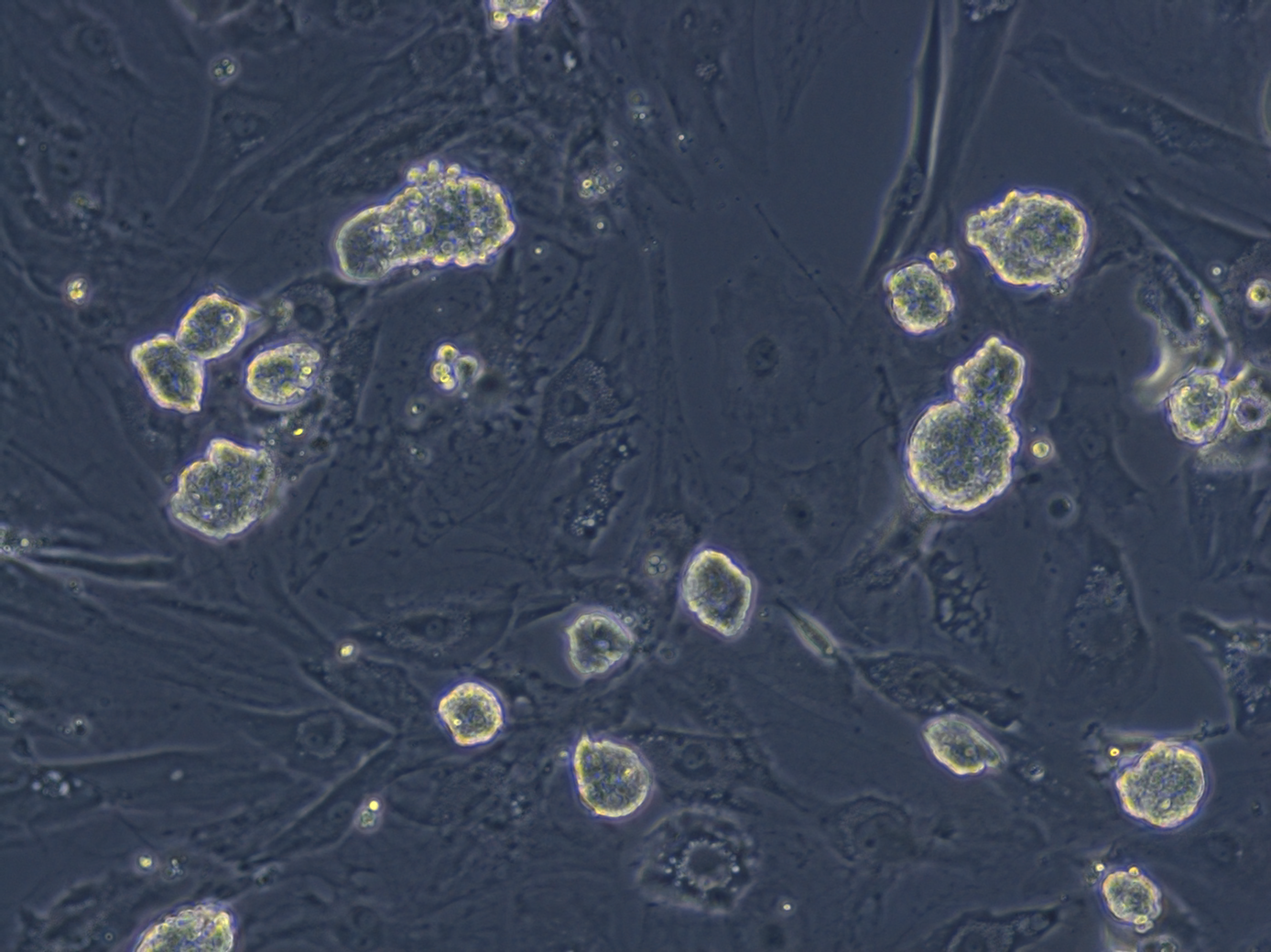

Supplement: Supplementary file 3 — Source data Fig. 1 [file 44318_2026_784_MOESM3_ESM.zip › Figure 1/S/Rot+QC1(5uM)+Pyr .tiff]

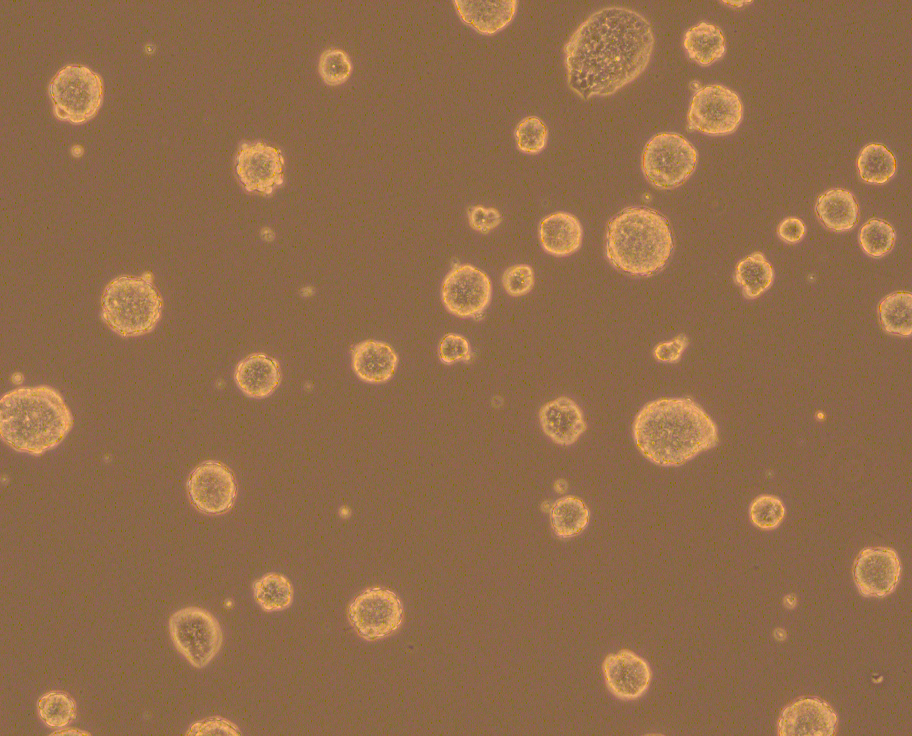

Supplement: Supplementary file 5 — Source data Fig. 3 [file 44318_2026_784_MOESM5_ESM.zip › Figure 3/A/P1/+QC1.tiff]

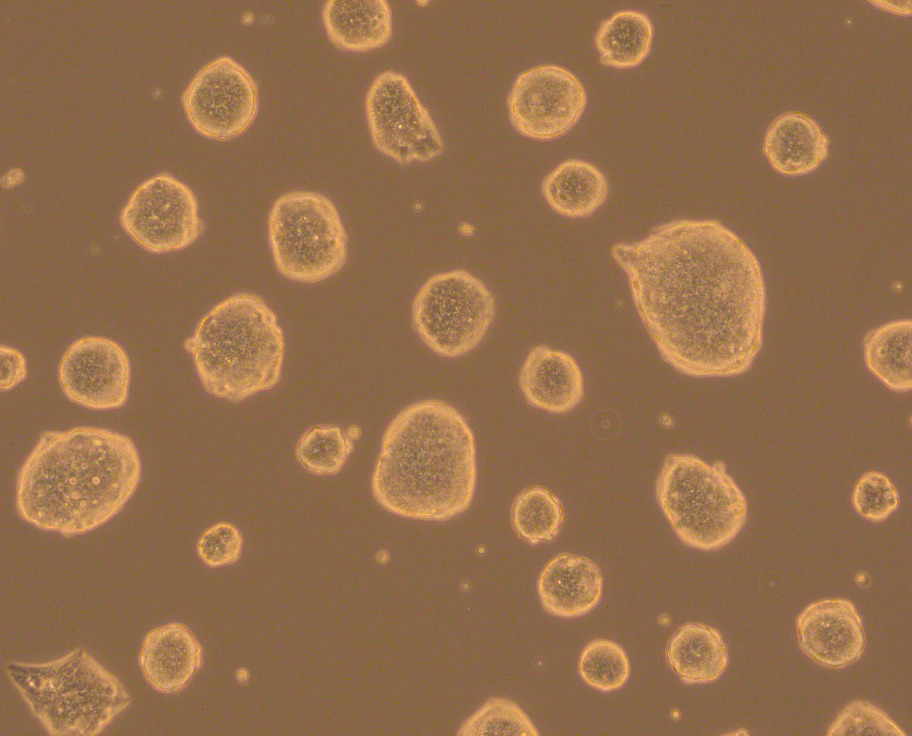

Supplement: Supplementary file 5 — Source data Fig. 3 [file 44318_2026_784_MOESM5_ESM.zip › Figure 3/A/P1/-QC1.tiff]

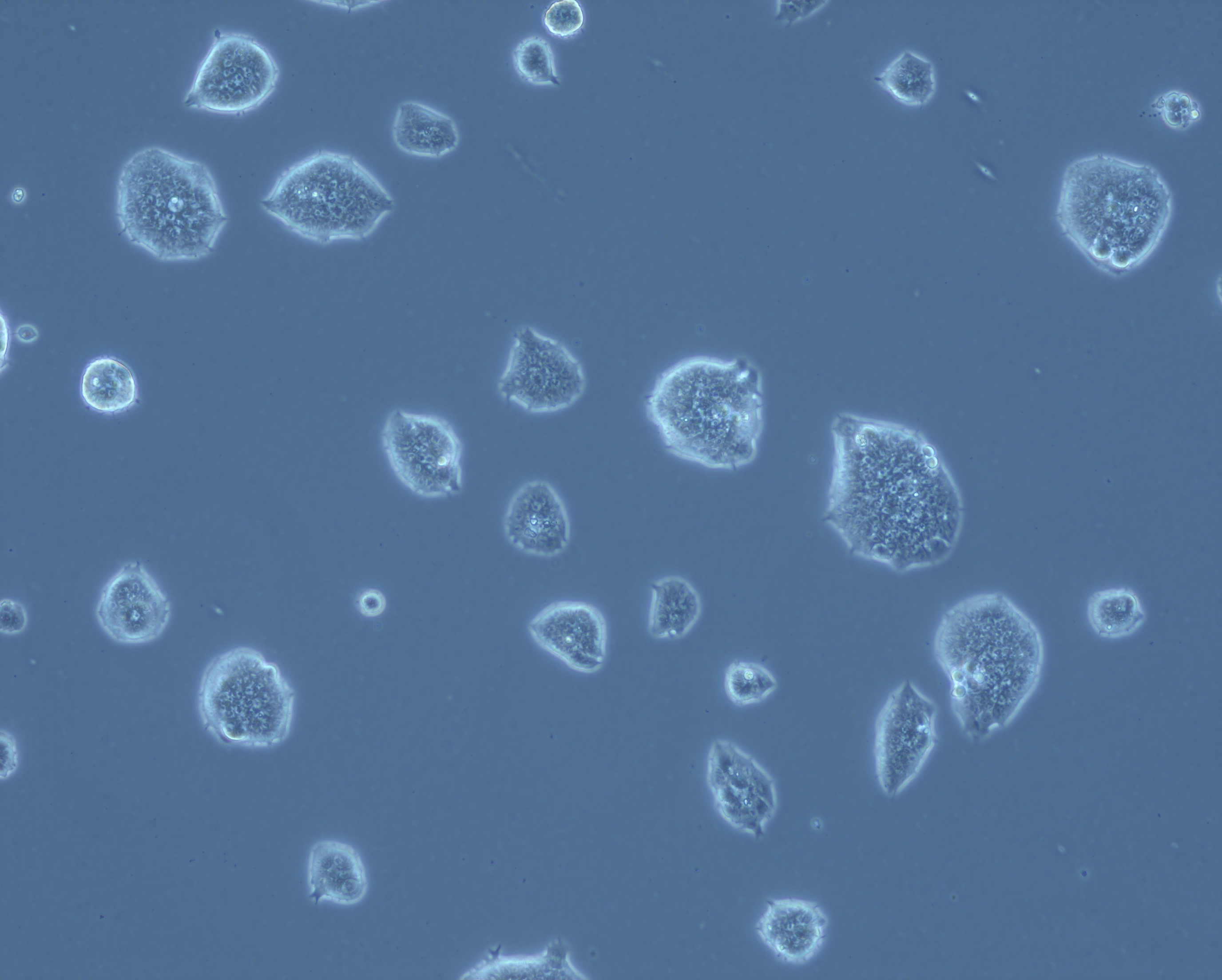

Supplement: Supplementary file 5 — Source data Fig. 3 [file 44318_2026_784_MOESM5_ESM.zip › Figure 3/A/P2/+QC1.jpg]

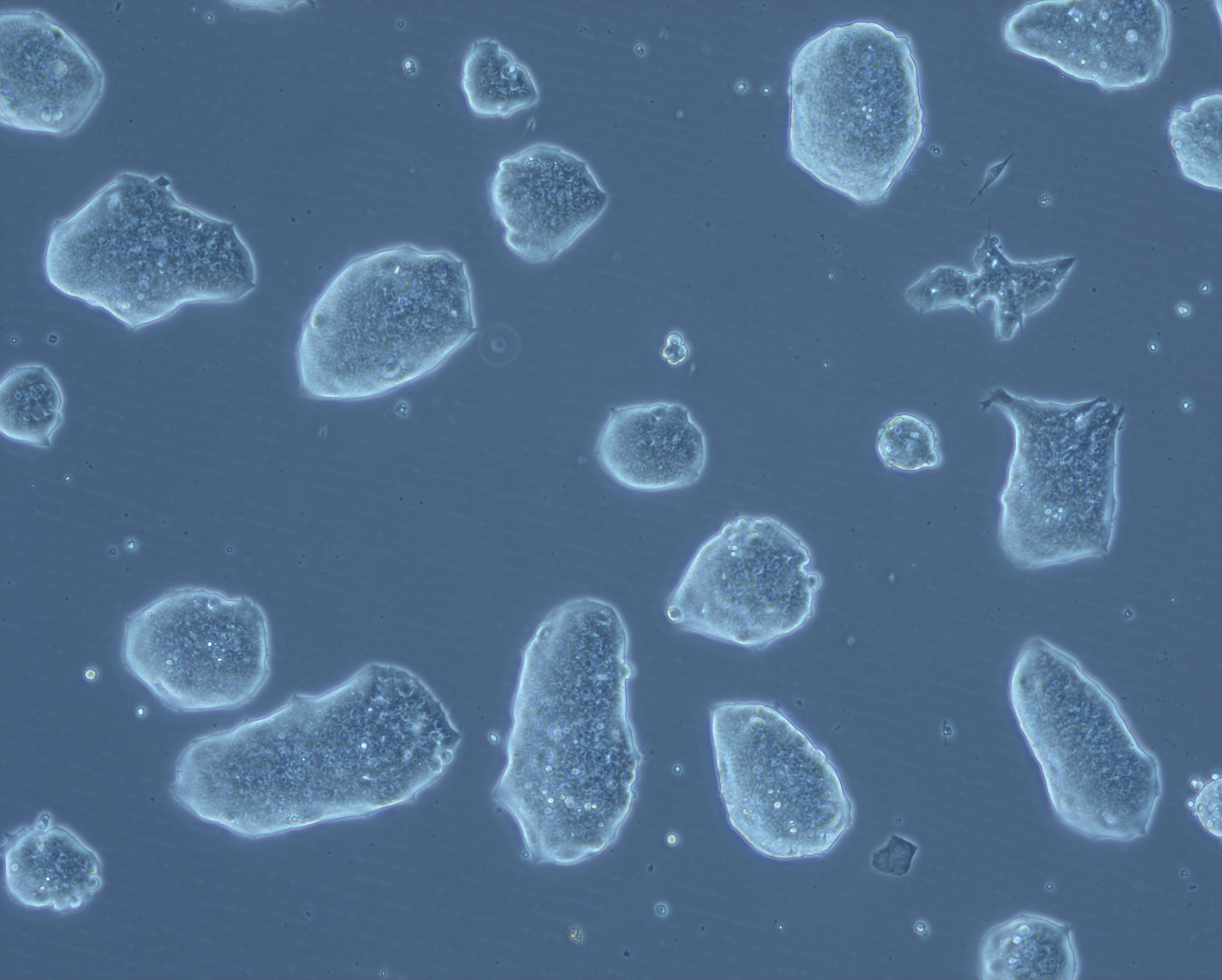

Supplement: Supplementary file 5 — Source data Fig. 3 [file 44318_2026_784_MOESM5_ESM.zip › Figure 3/A/P2/-QC1.jpg]

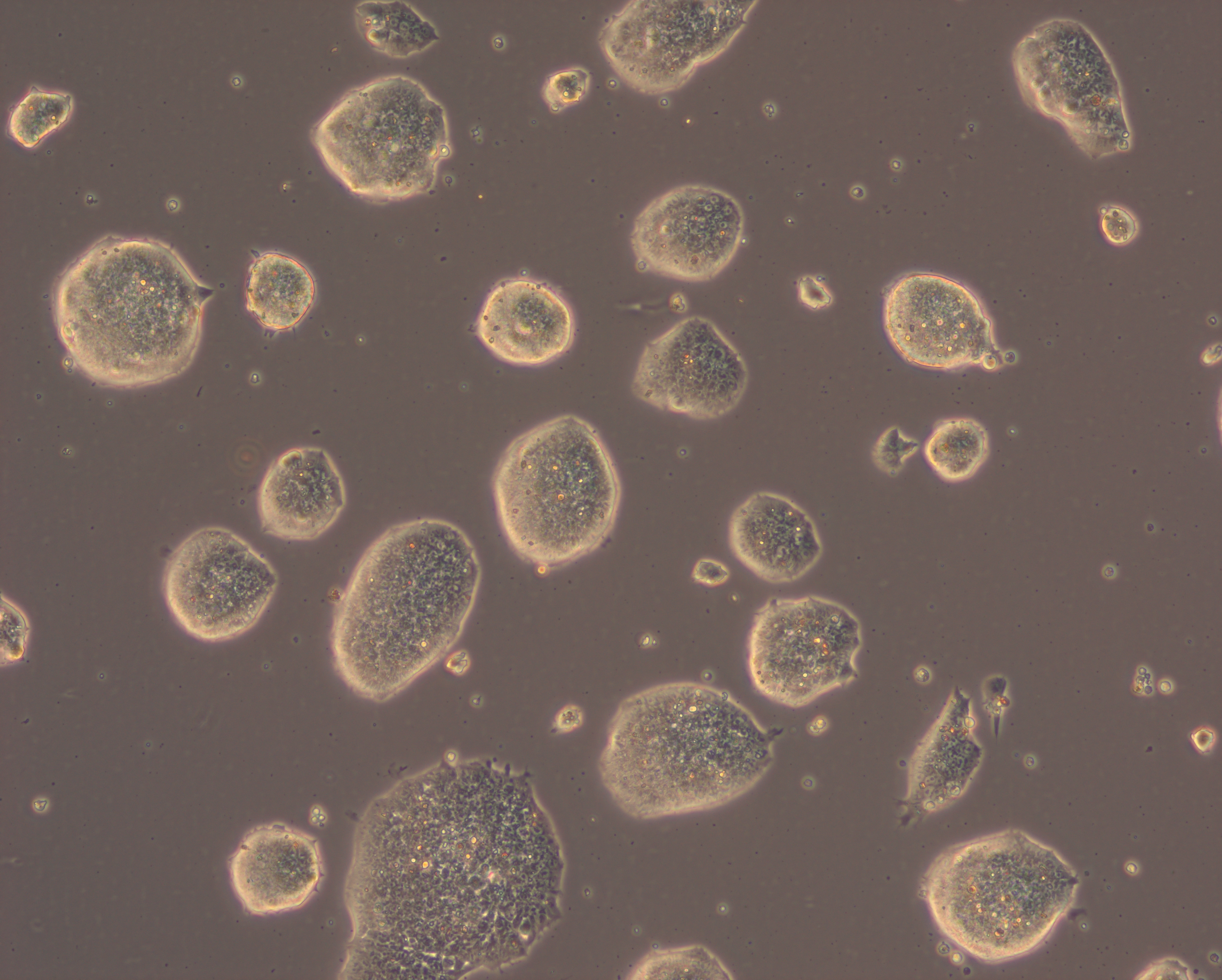

Supplement: Supplementary file 5 — Source data Fig. 3 [file 44318_2026_784_MOESM5_ESM.zip › Figure 3/A/P3/+QC1.tif]

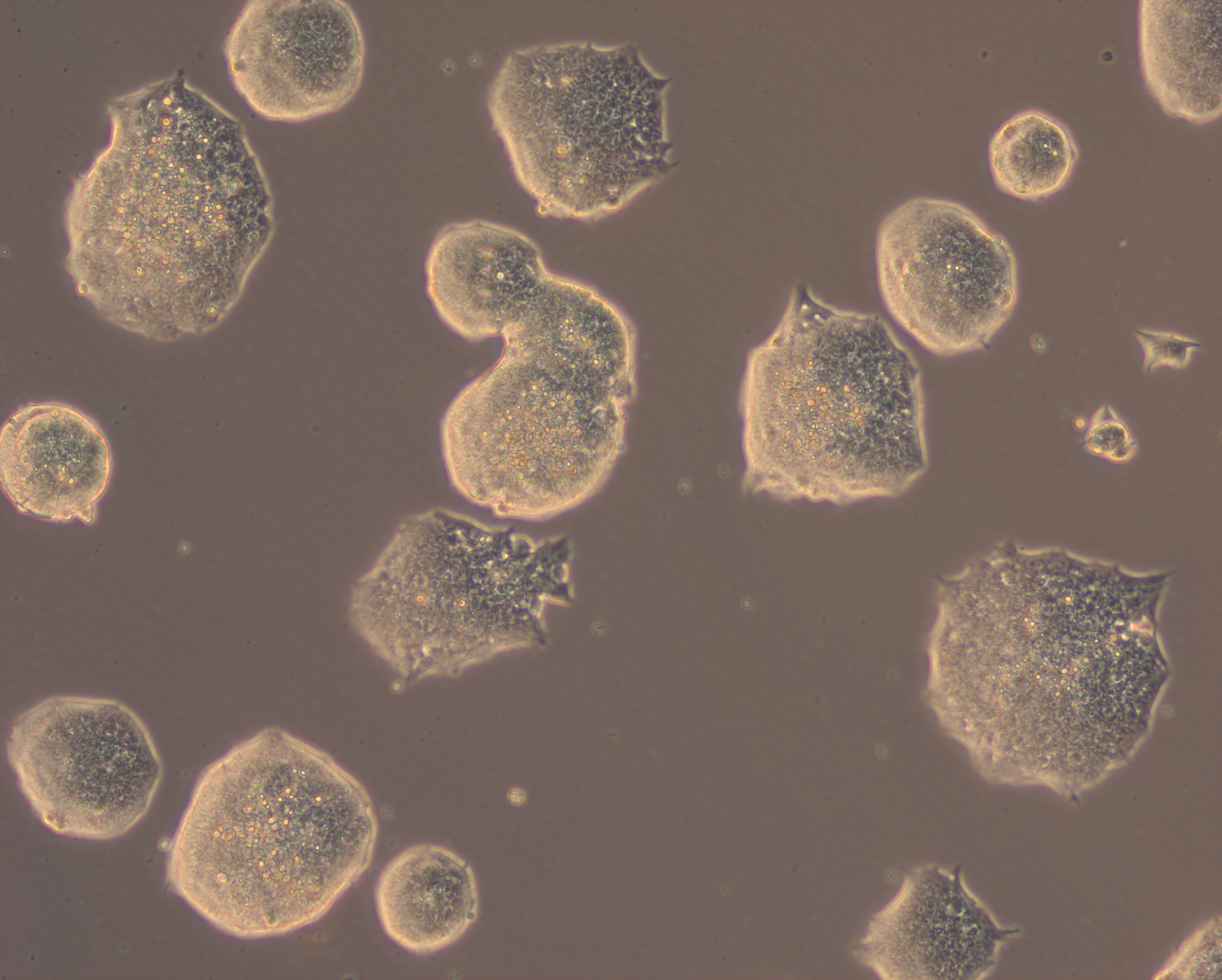

Supplement: Supplementary file 5 — Source data Fig. 3 [file 44318_2026_784_MOESM5_ESM.zip › Figure 3/A/P3/-QC1.tif]

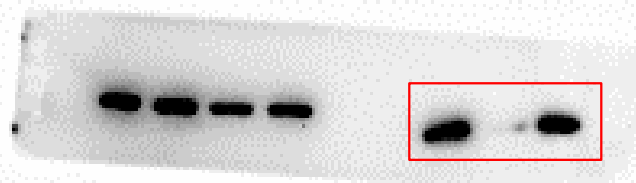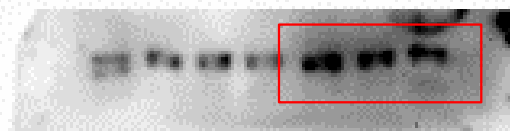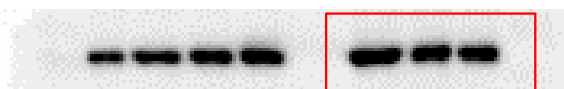

Supplement: Supplementary file 5 — Source data Fig. 3 [file 44318_2026_784_MOESM5_ESM.zip › Figure 3/C/Highlighted PDF Fig.3C.pdf]

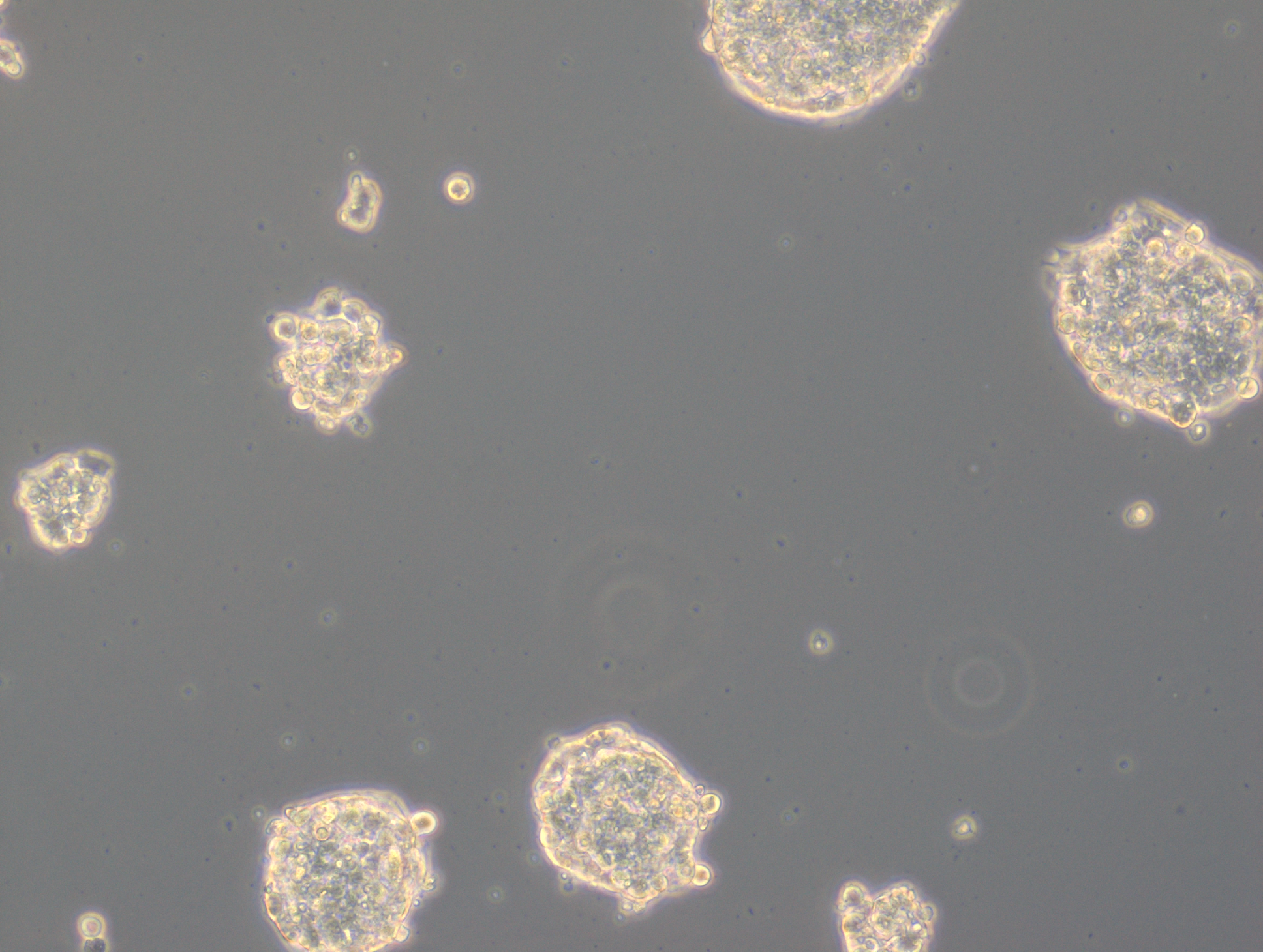

Supplement: Supplementary file 5 — Source data Fig. 3 [file 44318_2026_784_MOESM5_ESM.zip › Figure 3/D/+QC1+3-FP+Acetate.tif]

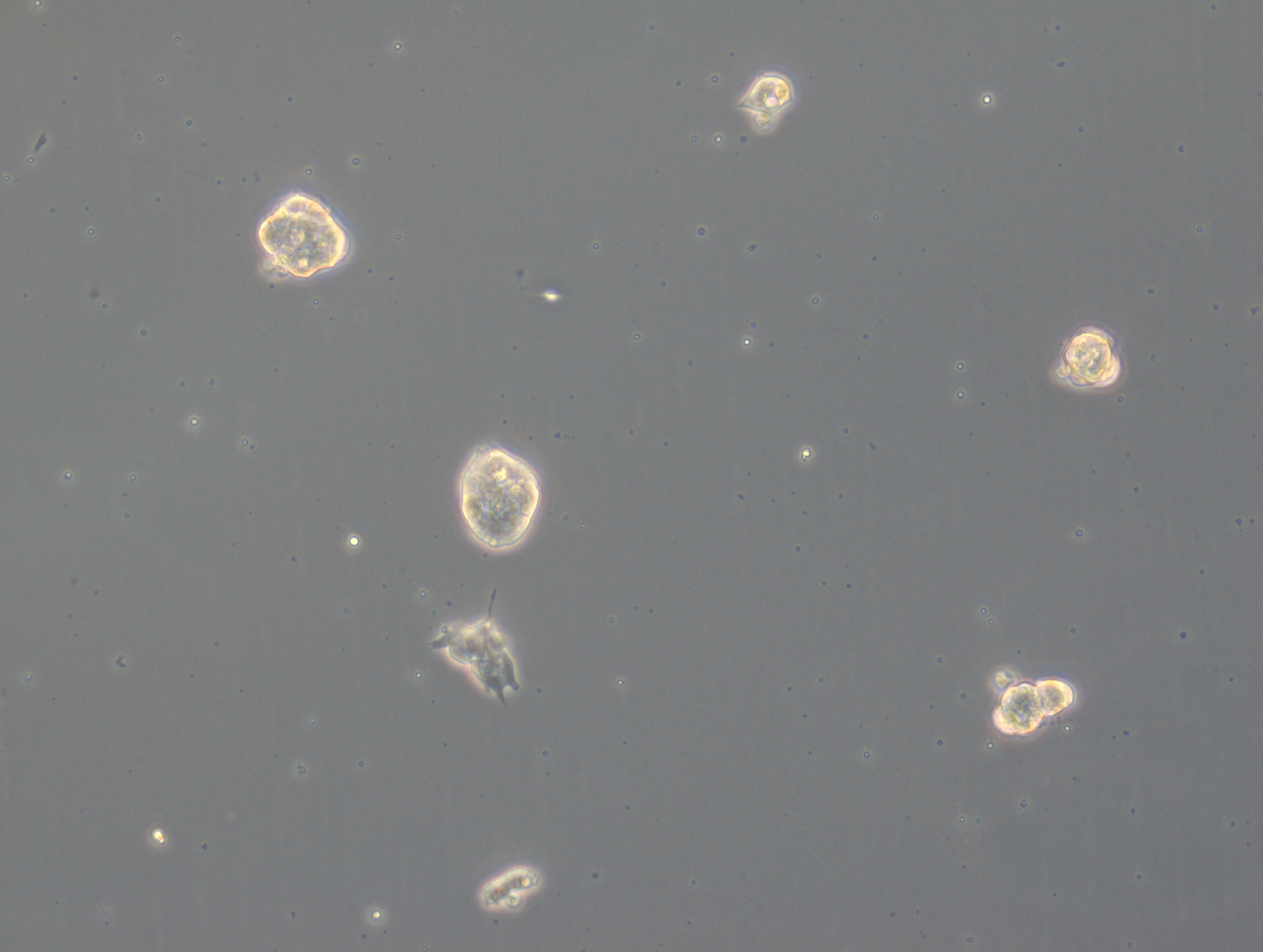

Supplement: Supplementary file 5 — Source data Fig. 3 [file 44318_2026_784_MOESM5_ESM.zip › Figure 3/D/+QC1+3-FP.tif]

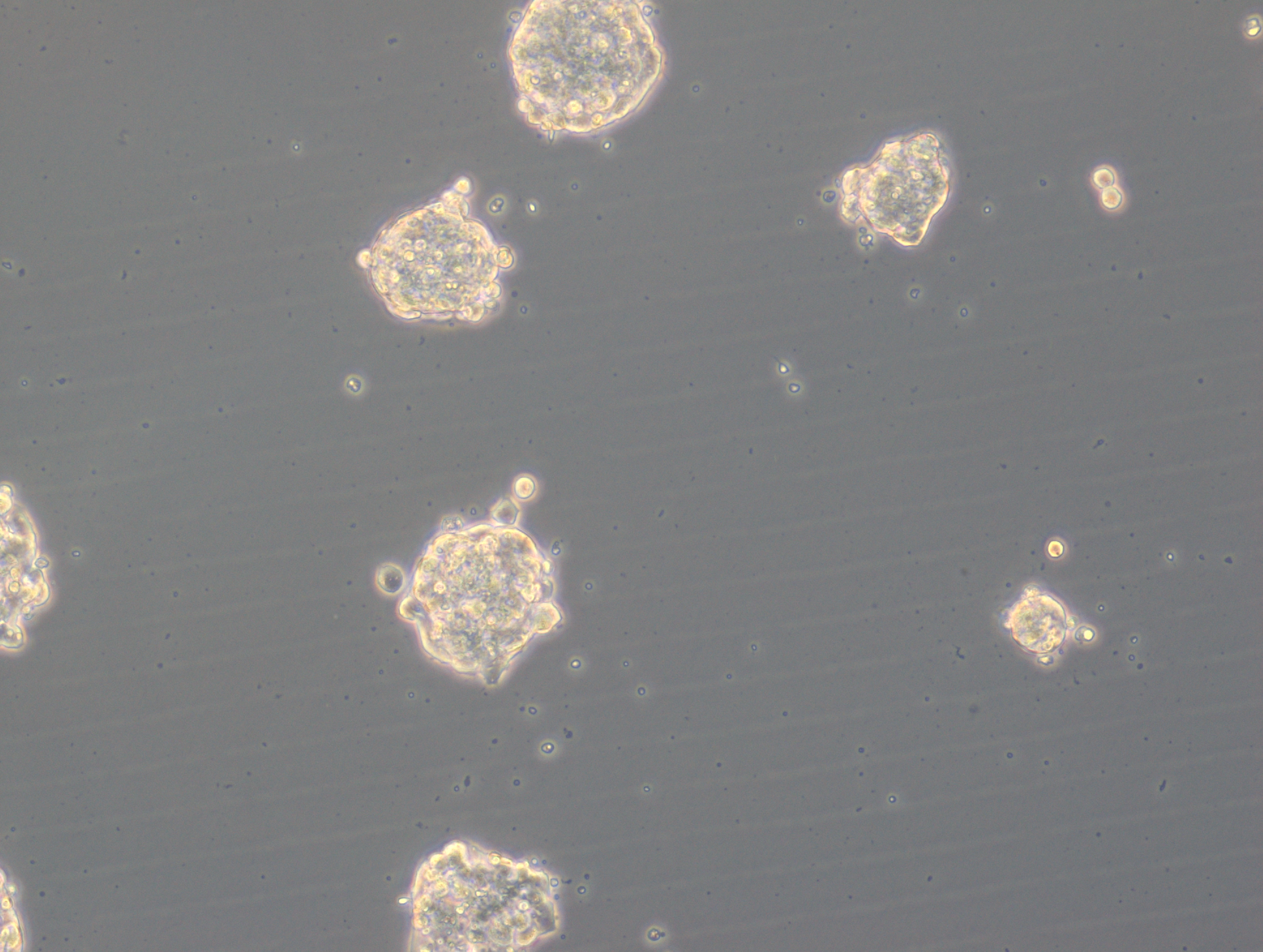

Supplement: Supplementary file 5 — Source data Fig. 3 [file 44318_2026_784_MOESM5_ESM.zip › Figure 3/D/+QC1+UK+Acetate.tif]

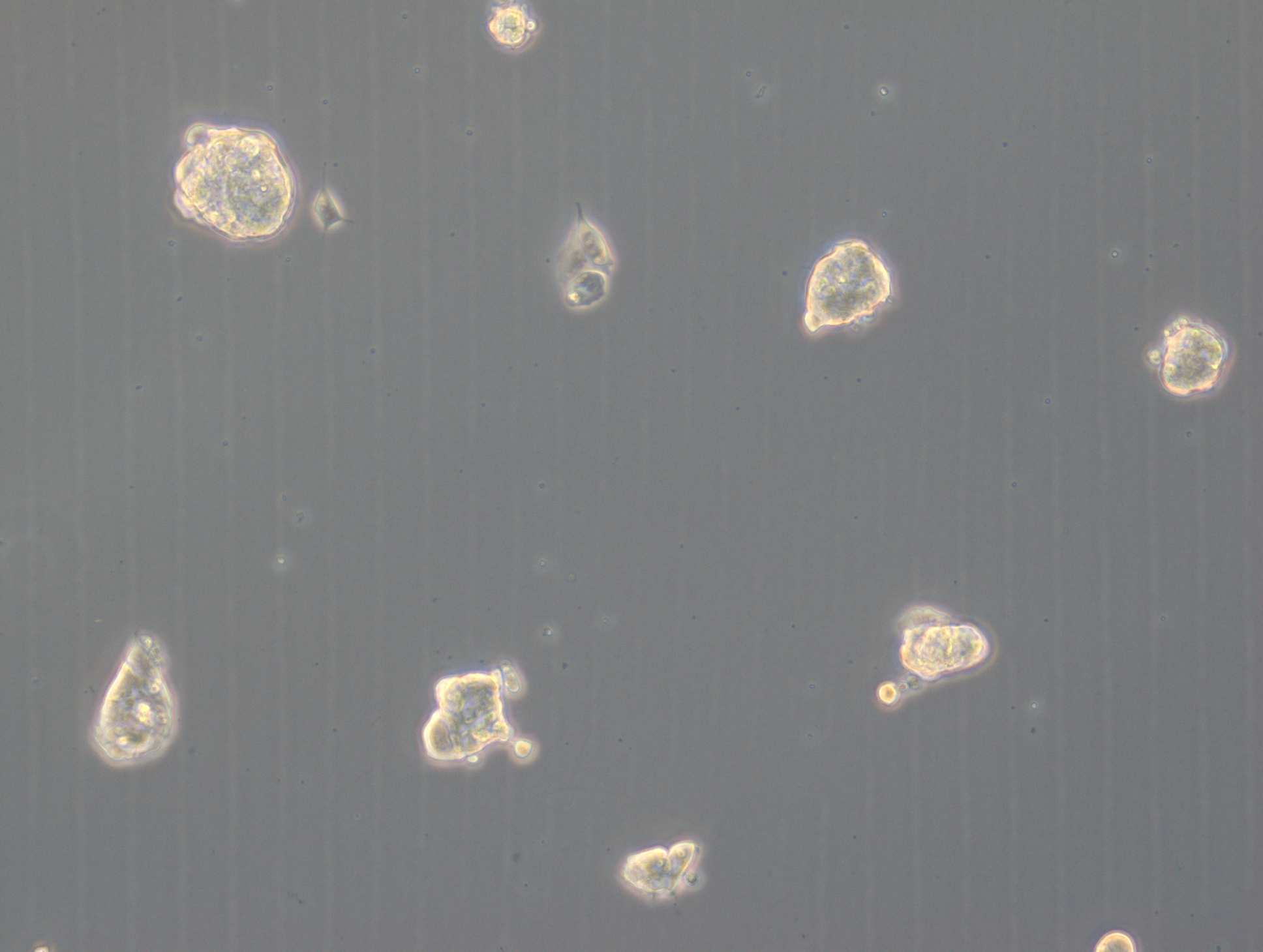

Supplement: Supplementary file 5 — Source data Fig. 3 [file 44318_2026_784_MOESM5_ESM.zip › Figure 3/D/+QC1+UK.tif]

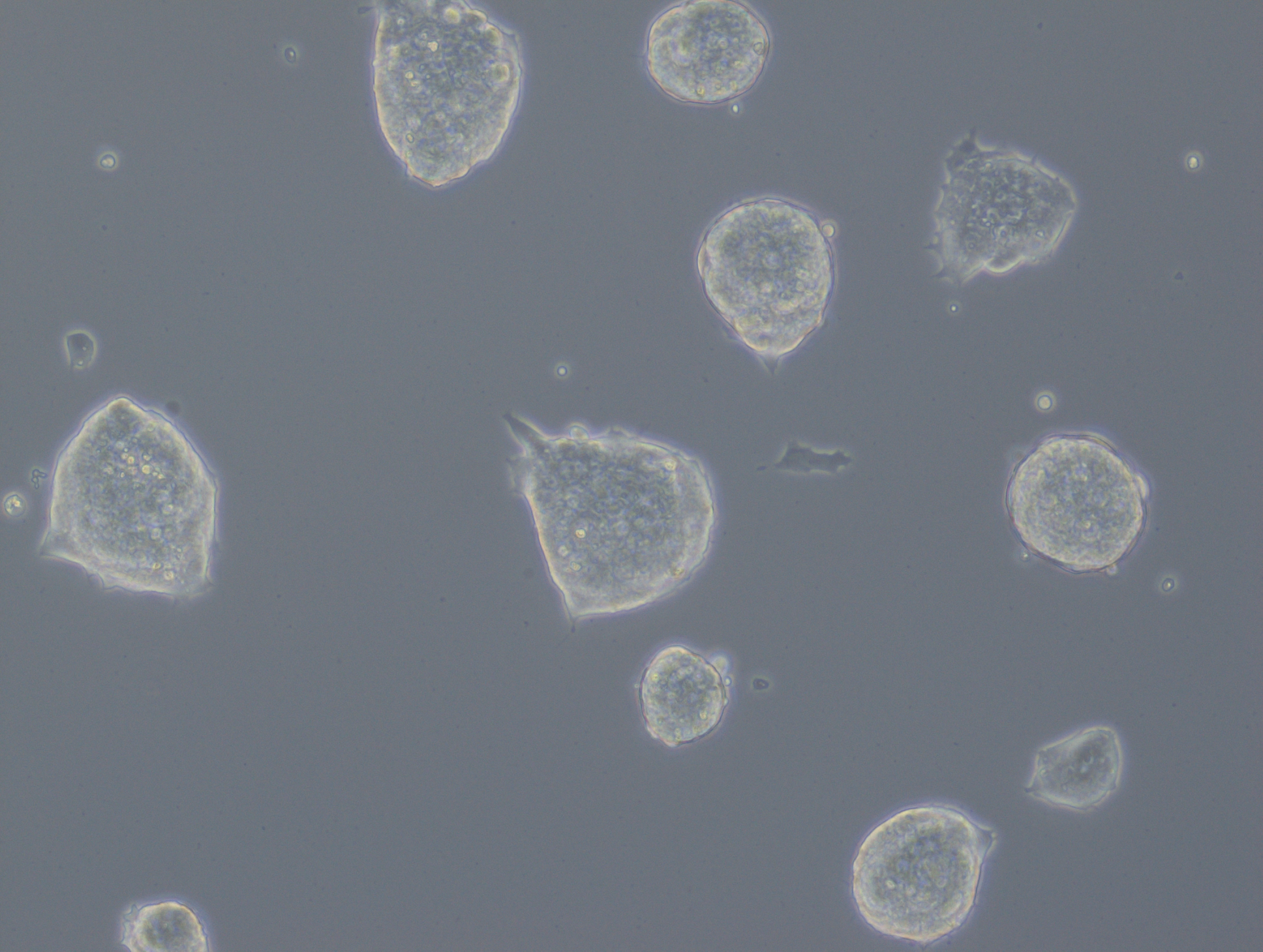

Supplement: Supplementary file 5 — Source data Fig. 3 [file 44318_2026_784_MOESM5_ESM.zip › Figure 3/D/+QC1.tif]

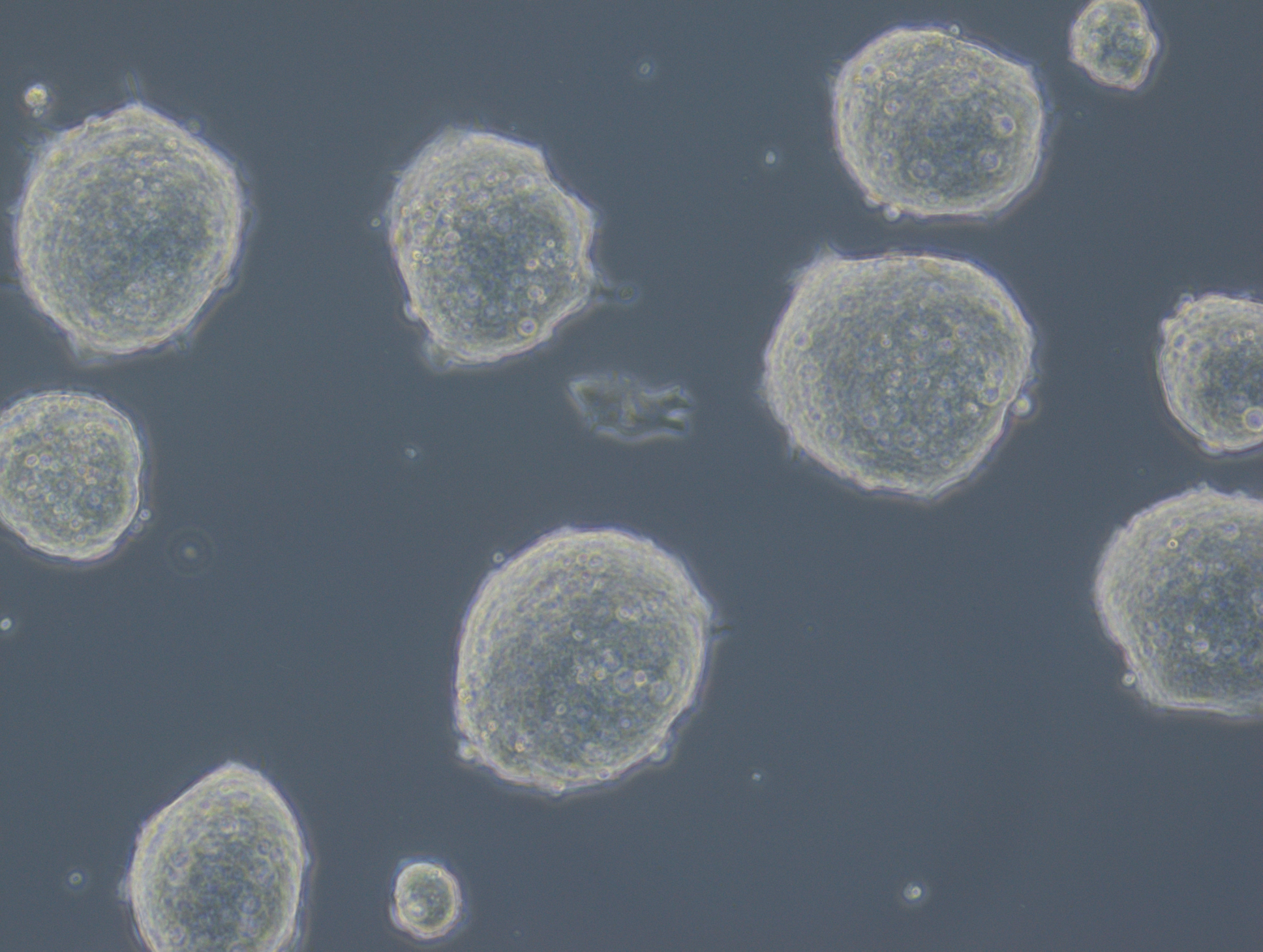

Supplement: Supplementary file 5 — Source data Fig. 3 [file 44318_2026_784_MOESM5_ESM.zip › Figure 3/D/-QC1.tif]

H3K27me3

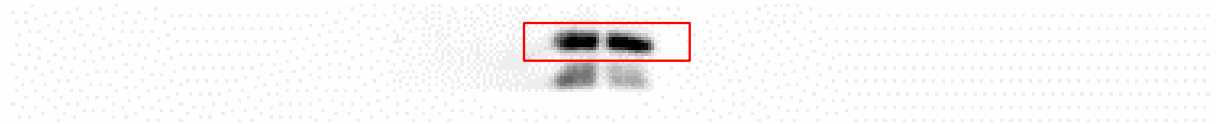

H4ac

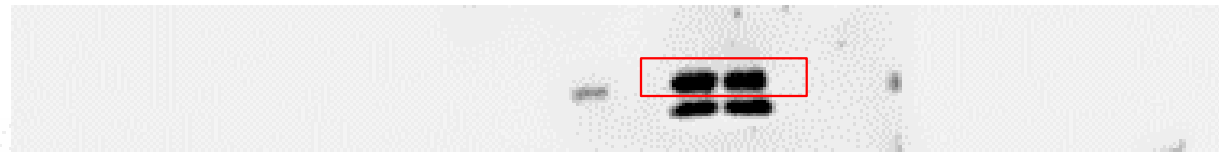

H3

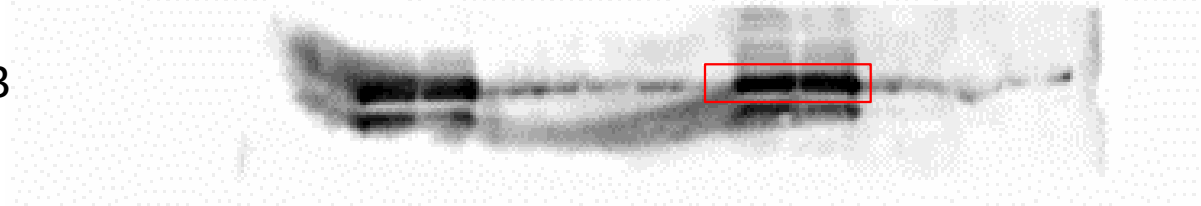

Supplement: Supplementary file 5 — Source data Fig. 3 [file 44318_2026_784_MOESM5_ESM.zip › Figure 3/E/Highlighted PDF Fig.3E.pdf]

5mC

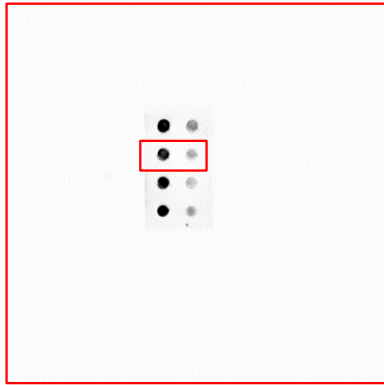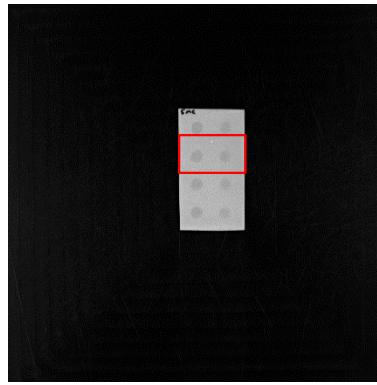

MB

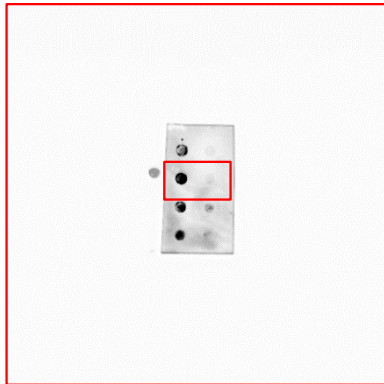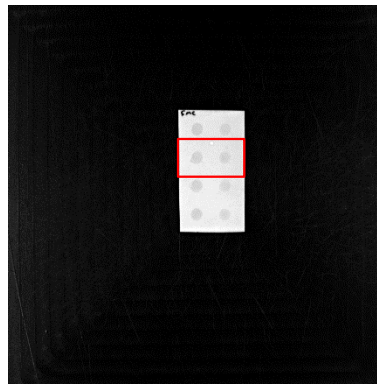

MB

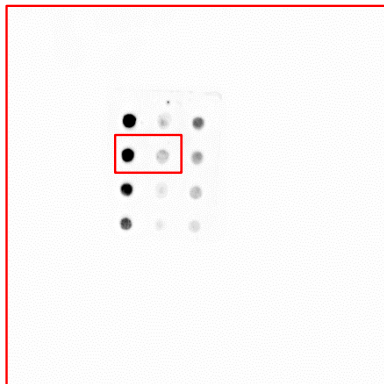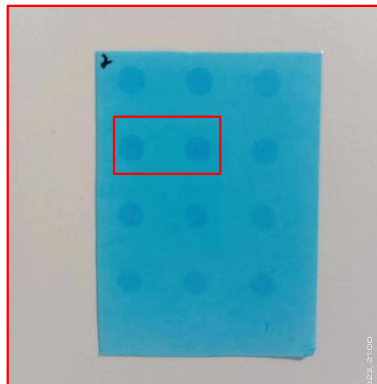

MB

Supplement: Supplementary file 5 — Source data Fig. 3 [file 44318_2026_784_MOESM5_ESM.zip › Figure 3/F/Highlighted PDF Fig.3F.pdf]

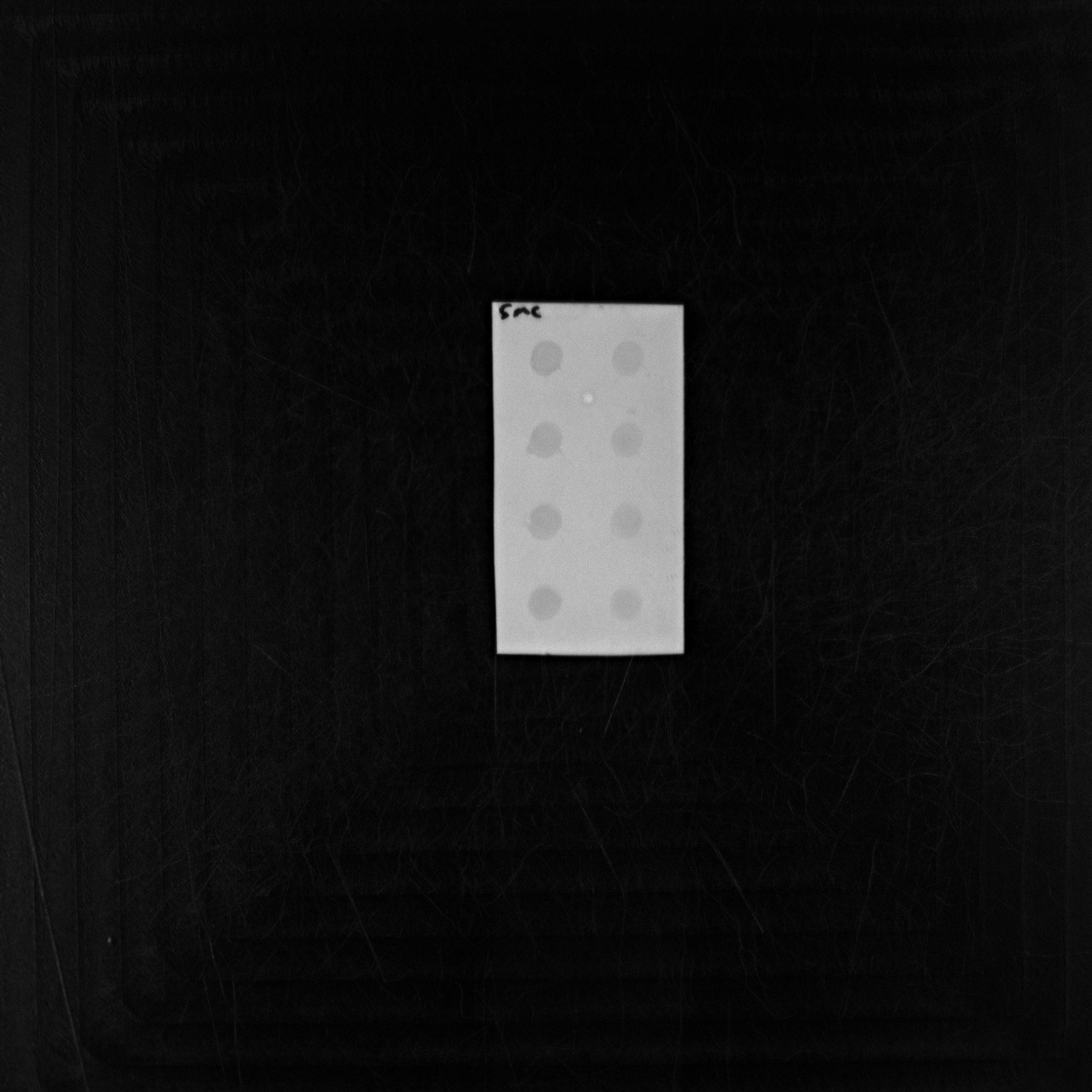

Supplement: Supplementary file 5 — Source data Fig. 3 [file 44318_2026_784_MOESM5_ESM.zip › Figure 3/F/MB-R1.Tif]

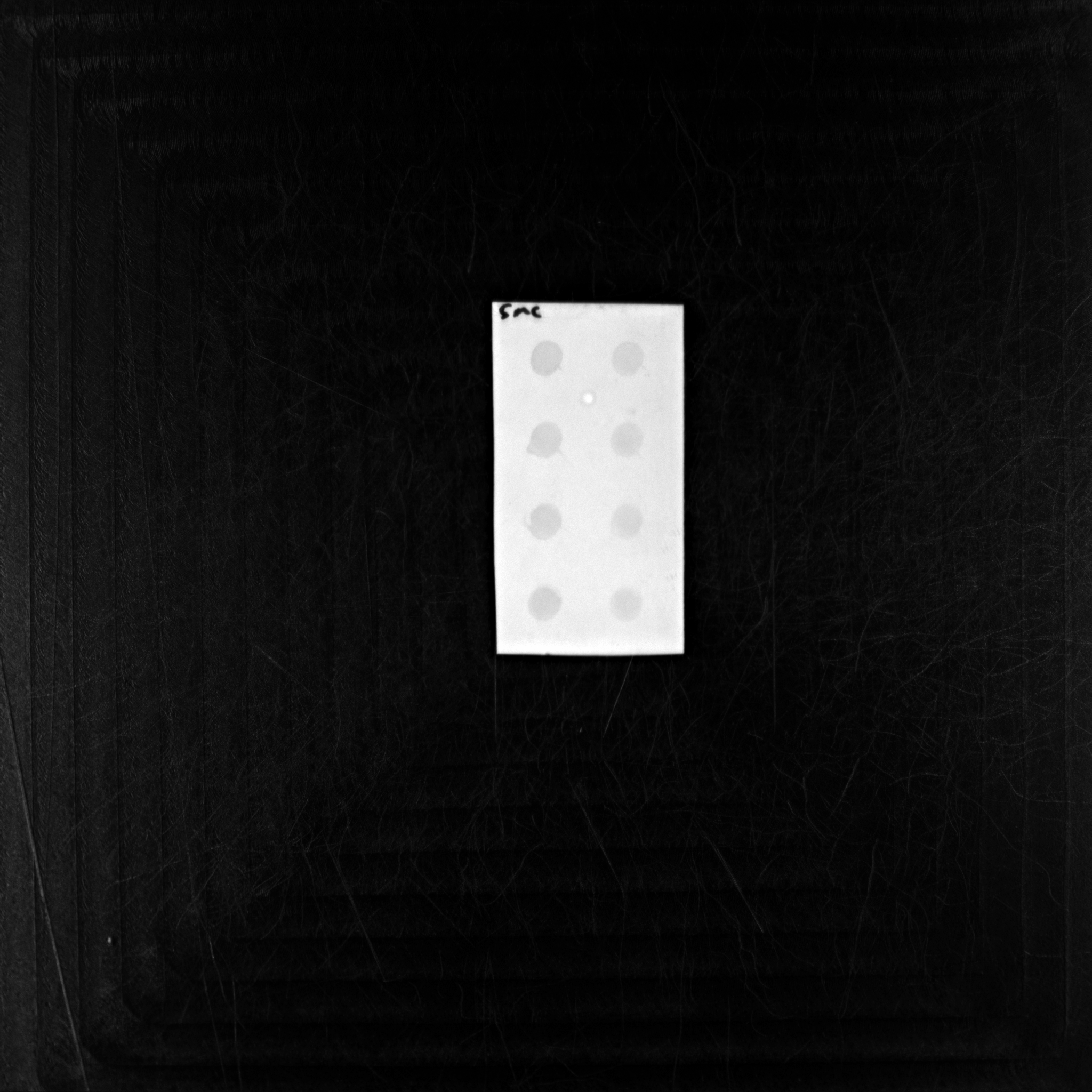

Supplement: Supplementary file 5 — Source data Fig. 3 [file 44318_2026_784_MOESM5_ESM.zip › Figure 3/F/MB-R2.Tif]

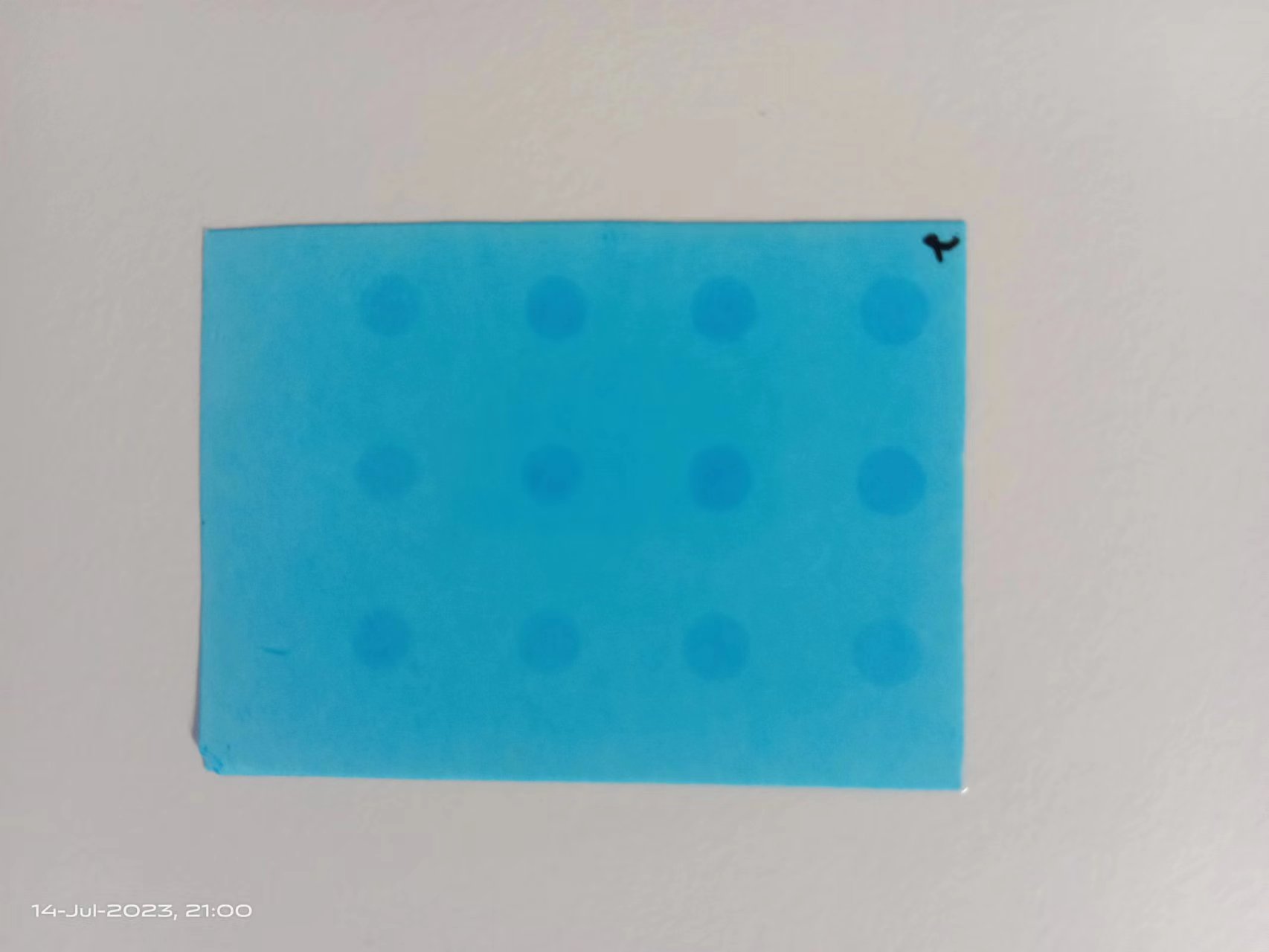

Supplement: Supplementary file 5 — Source data Fig. 3 [file 44318_2026_784_MOESM5_ESM.zip › Figure 3/F/MB-R3.jpg]

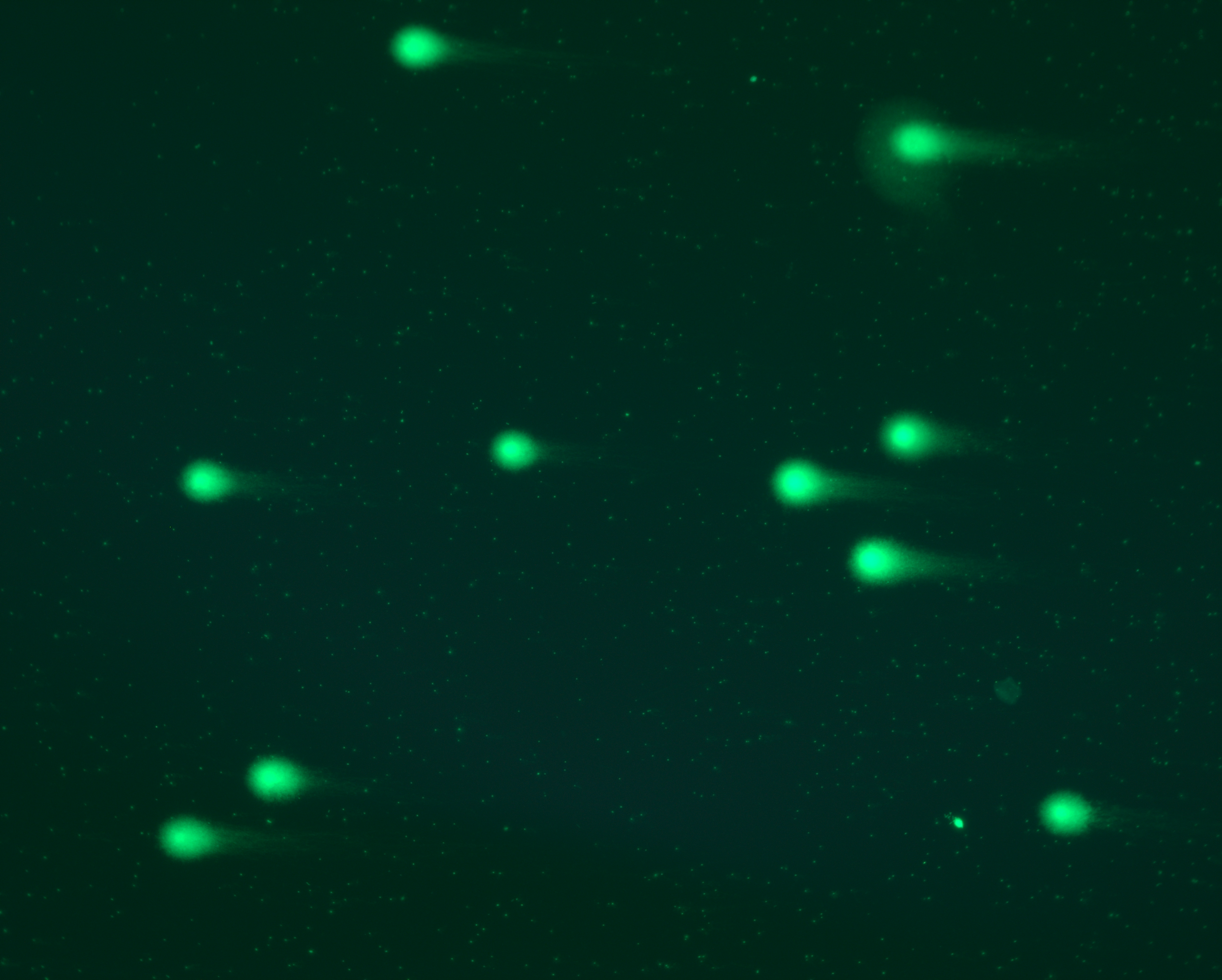

Supplement: Supplementary file 5 — Source data Fig. 3 [file 44318_2026_784_MOESM5_ESM.zip › Figure 3/G/+QC1.tif]

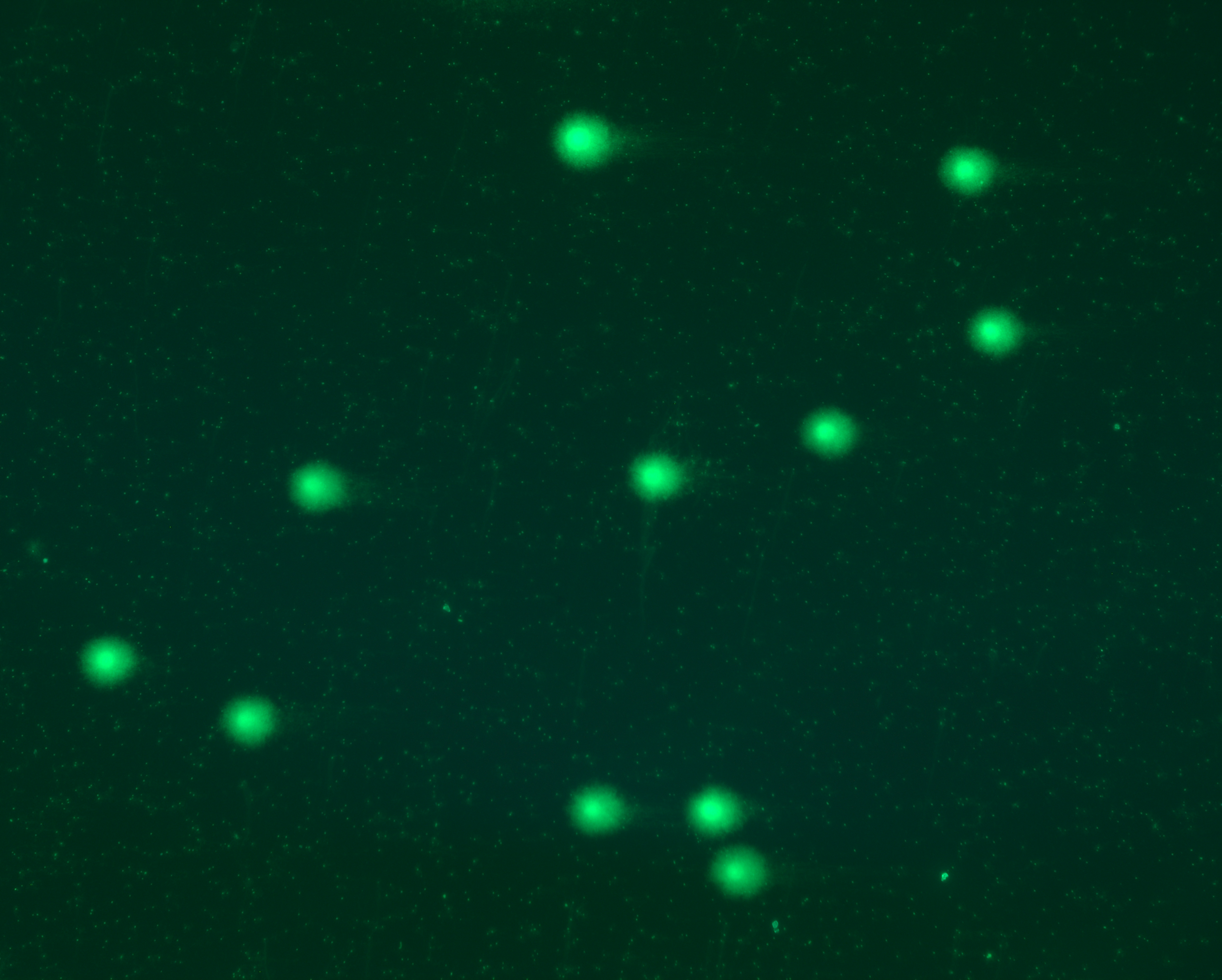

Supplement: Supplementary file 5 — Source data Fig. 3 [file 44318_2026_784_MOESM5_ESM.zip › Figure 3/G/-QC1.tif]

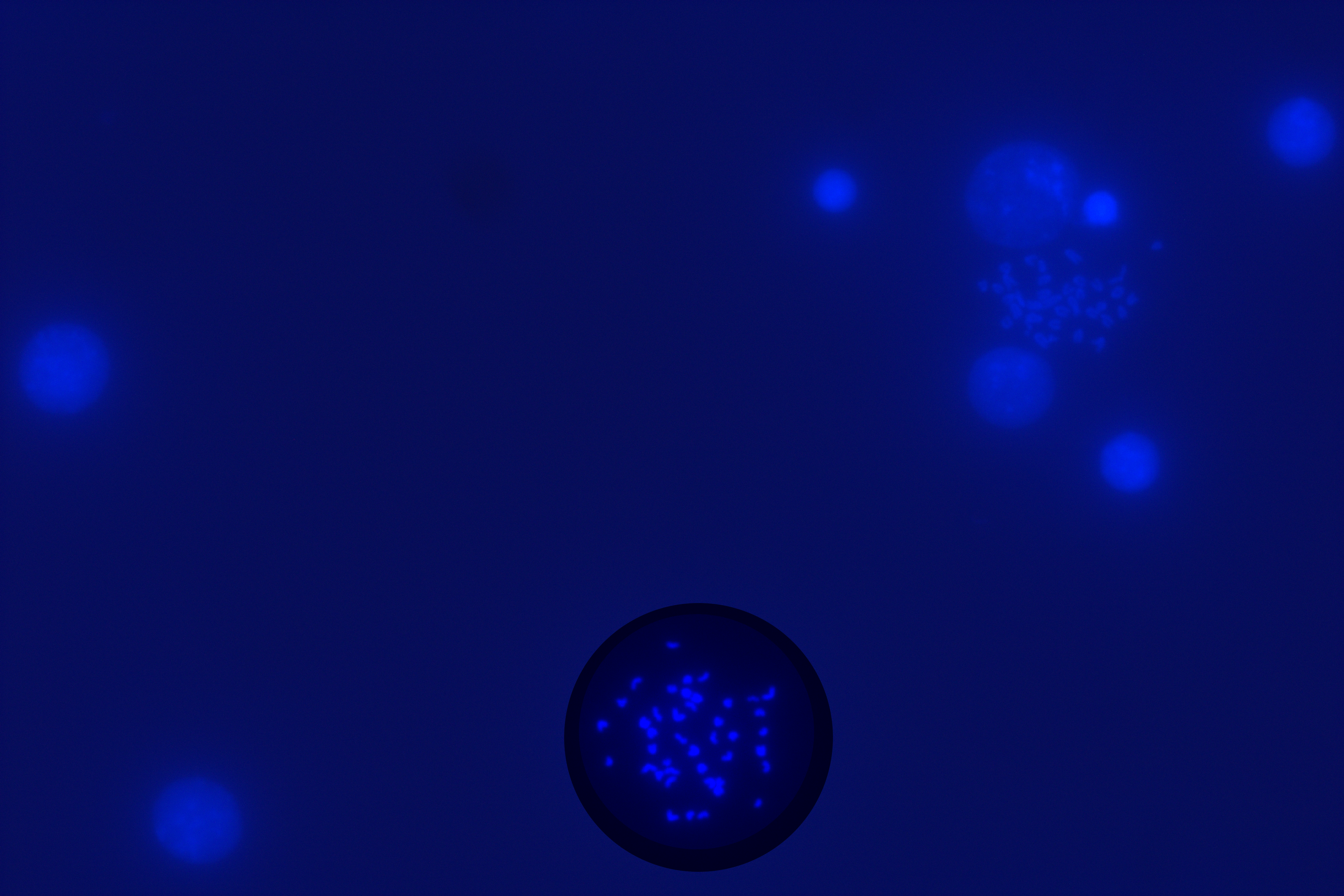

Supplement: Supplementary file 5 — Source data Fig. 3 [file 44318_2026_784_MOESM5_ESM.zip › Figure 3/H/+QC1 Photoshop.tif]

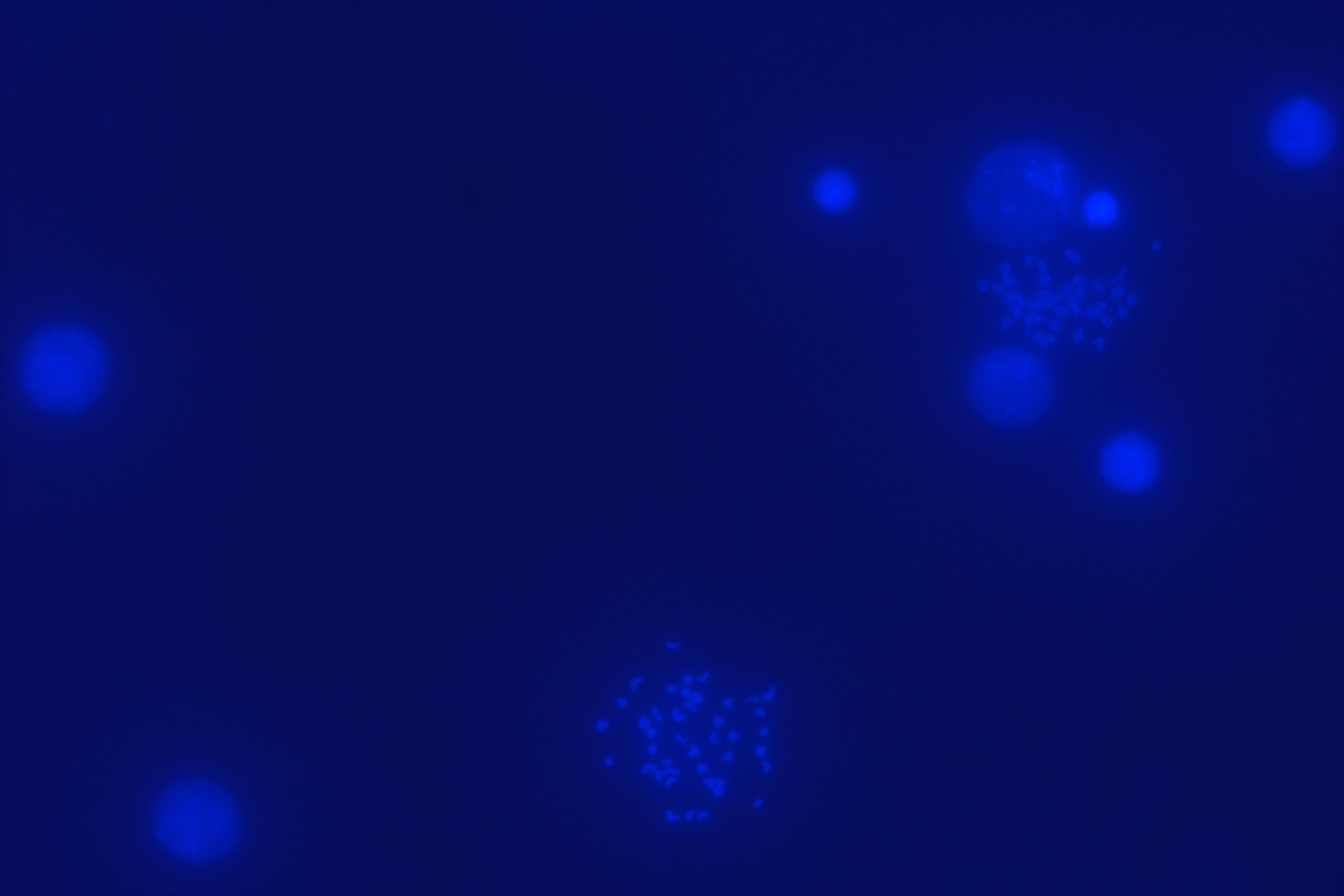

Supplement: Supplementary file 5 — Source data Fig. 3 [file 44318_2026_784_MOESM5_ESM.zip › Figure 3/H/+QC1.tif]

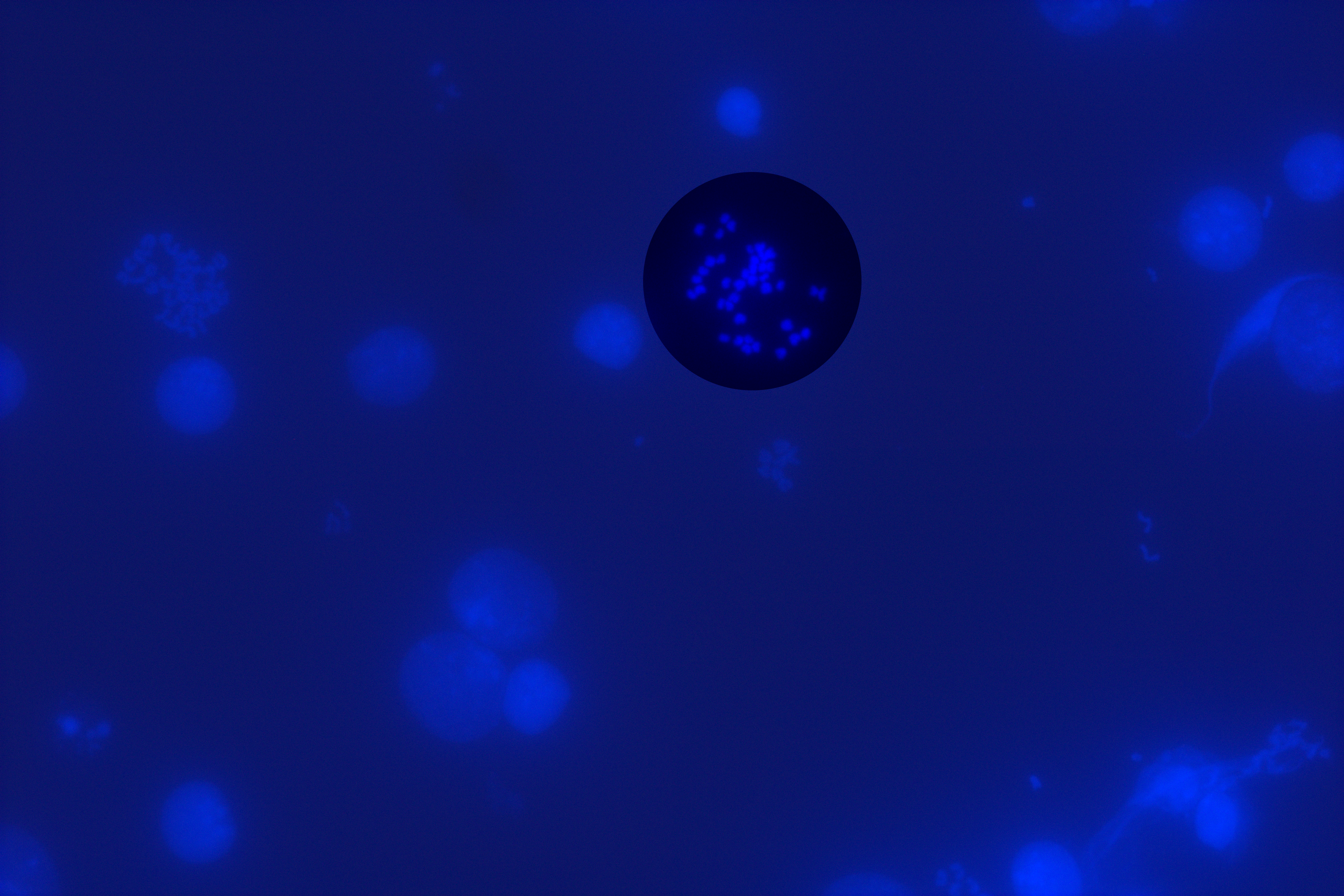

Supplement: Supplementary file 5 — Source data Fig. 3 [file 44318_2026_784_MOESM5_ESM.zip › Figure 3/H/-QC1 photoshop.tif]

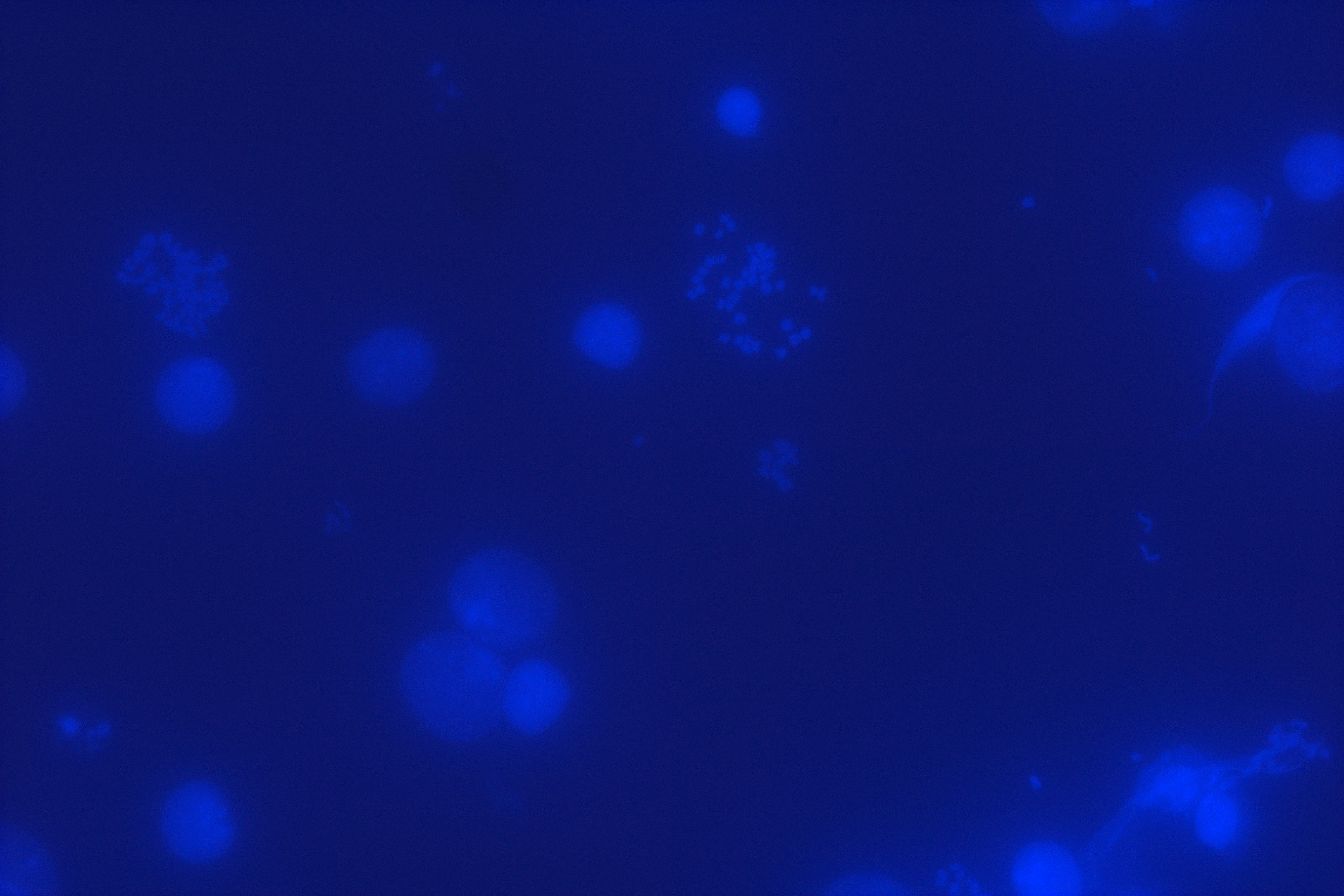

Supplement: Supplementary file 5 — Source data Fig. 3 [file 44318_2026_784_MOESM5_ESM.zip › Figure 3/H/-QC1.tif]

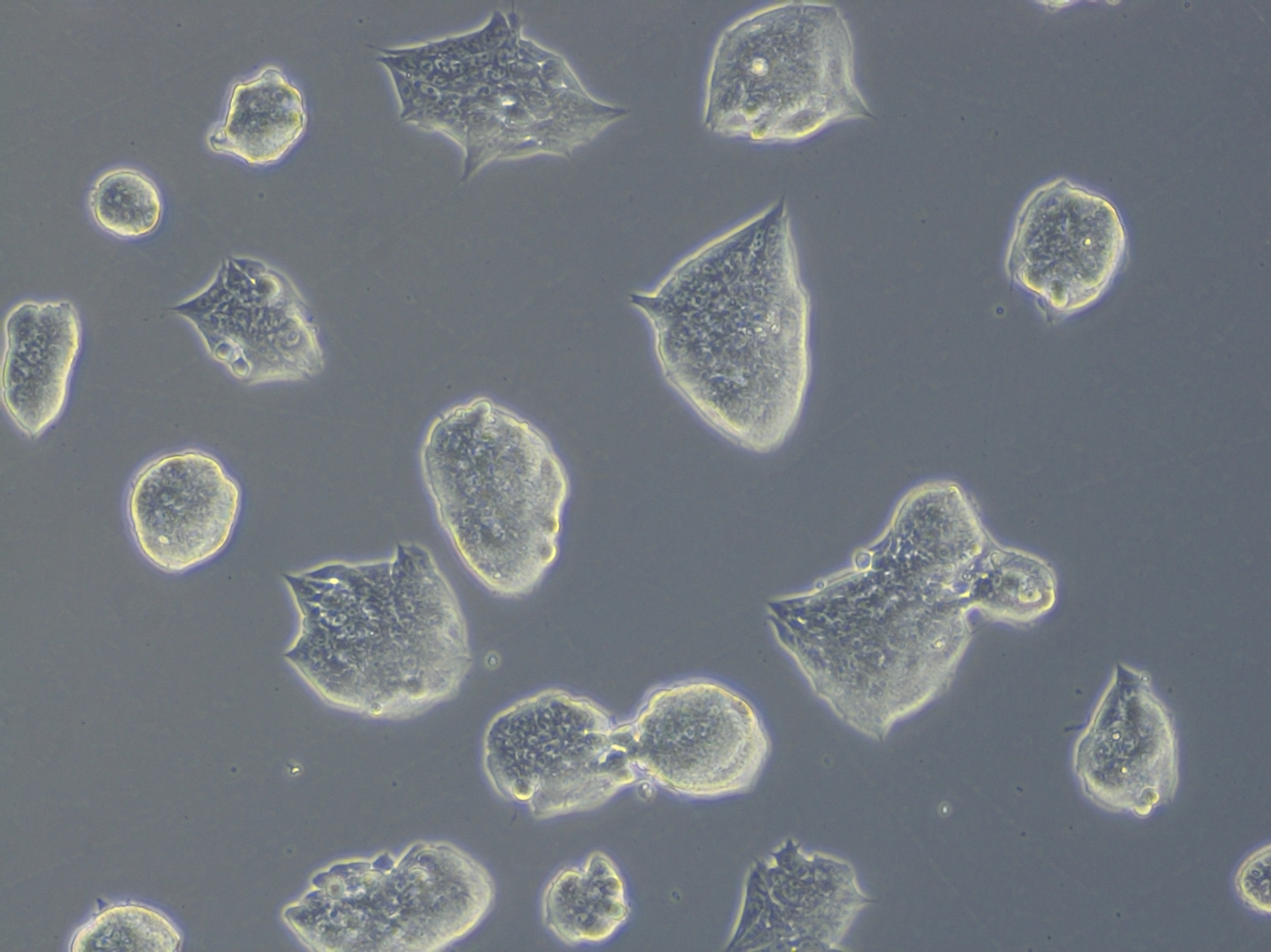

Supplement: Supplementary file 5 — Source data Fig. 3 [file 44318_2026_784_MOESM5_ESM.zip › Figure 3/J/Ctrl.jpg]

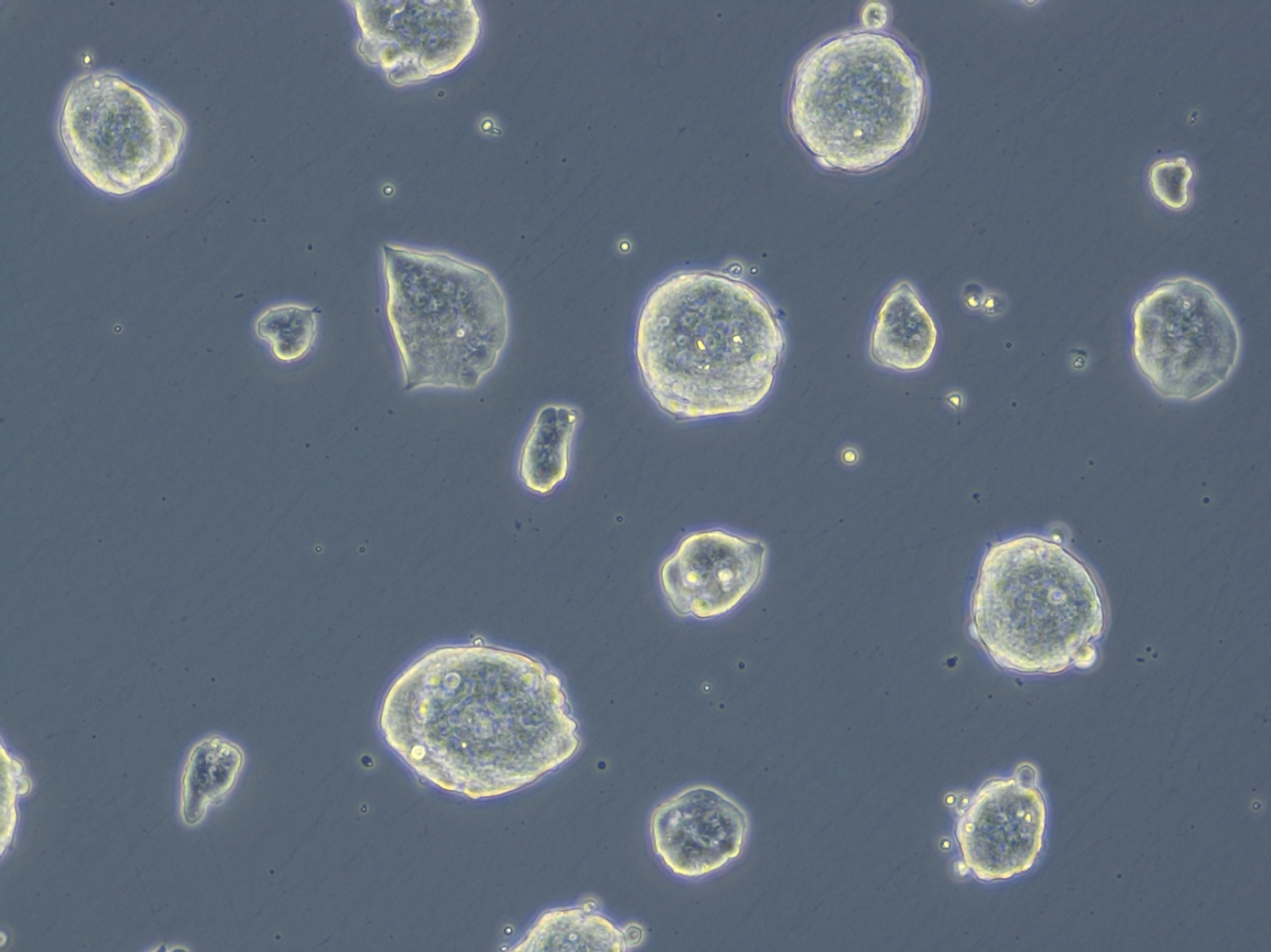

Supplement: Supplementary file 5 — Source data Fig. 3 [file 44318_2026_784_MOESM5_ESM.zip › Figure 3/J/Ndi1.jpg]

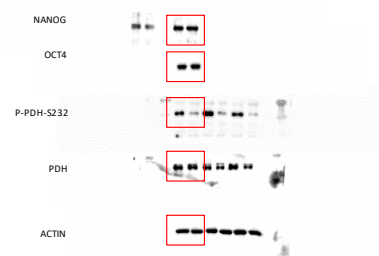

Supplement: Supplementary file 5 — Source data Fig. 3 [file 44318_2026_784_MOESM5_ESM.zip › Figure 3/K/Highlighted PDF Fig.3K.pdf]

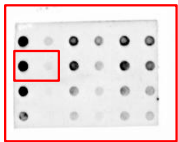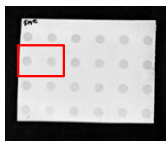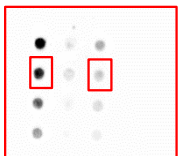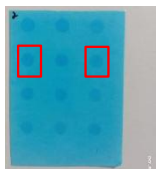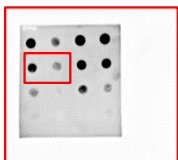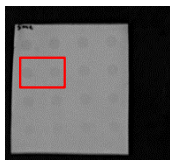

Supplement: Supplementary file 5 — Source data Fig. 3 [file 44318_2026_784_MOESM5_ESM.zip › Figure 3/M/Highlighted PDF Fig.3M.pdf]

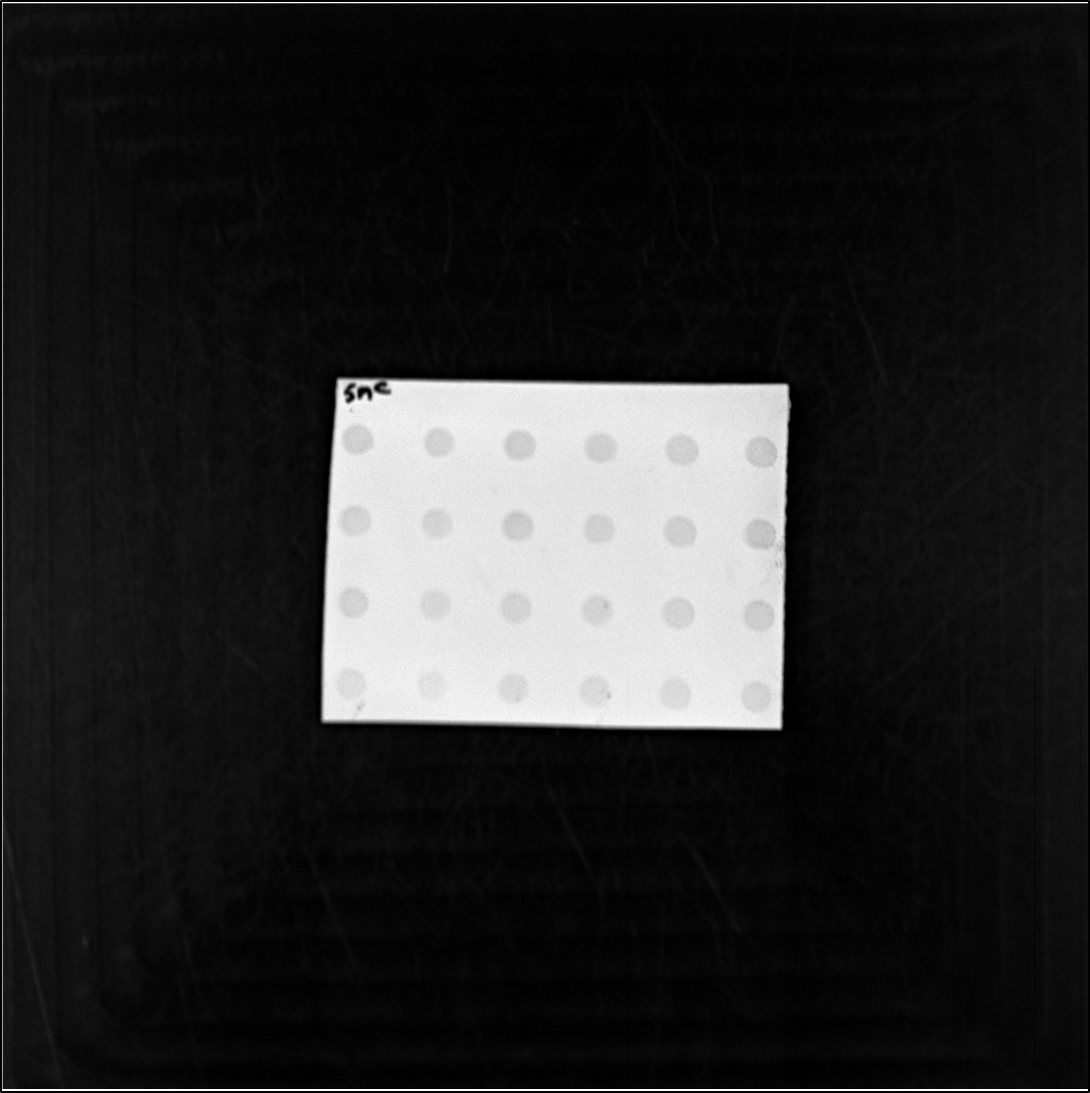

Supplement: Supplementary file 5 — Source data Fig. 3 [file 44318_2026_784_MOESM5_ESM.zip › Figure 3/M/Ndi1 5mC MB R1.tif]

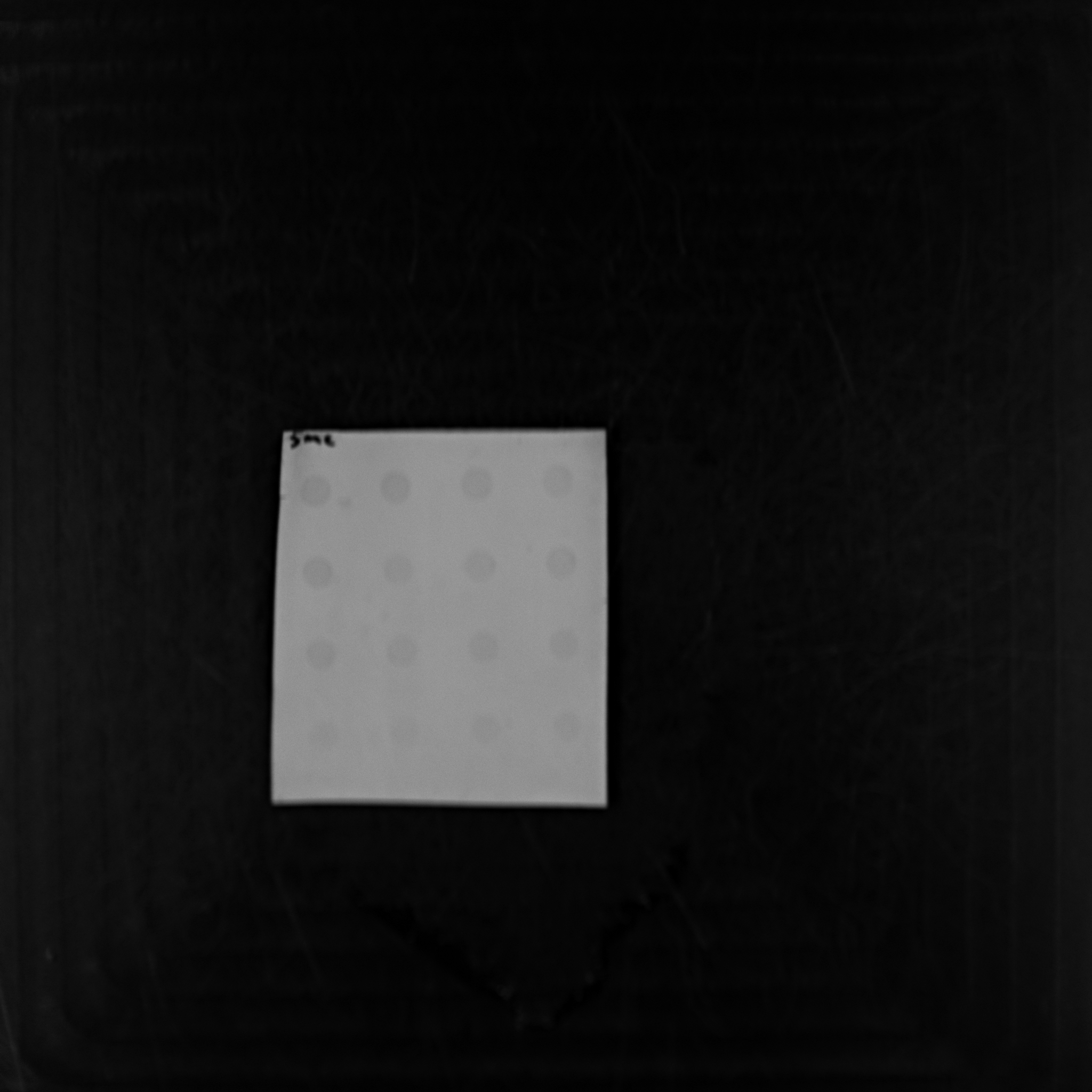

Supplement: Supplementary file 5 — Source data Fig. 3 [file 44318_2026_784_MOESM5_ESM.zip › Figure 3/M/Ndi1 5mC MB R3.Tif]

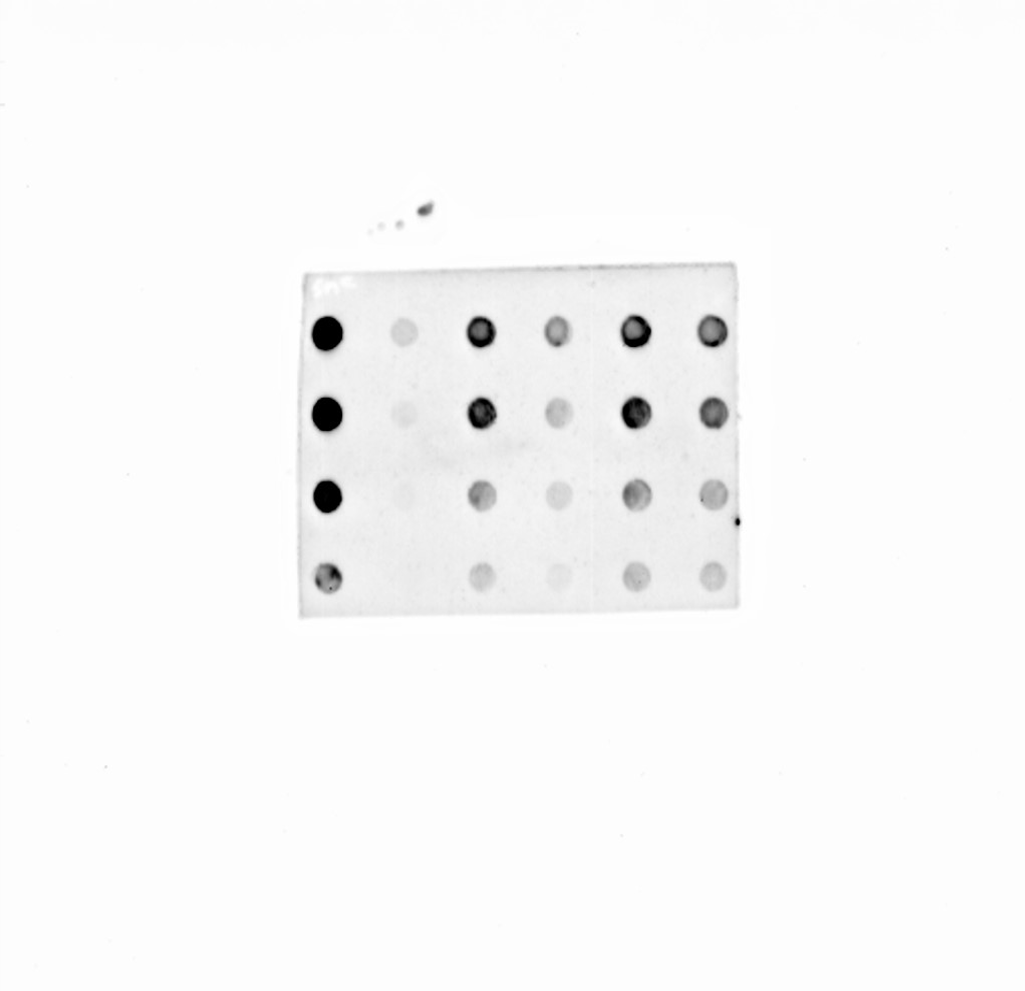

Supplement: Supplementary file 5 — Source data Fig. 3 [file 44318_2026_784_MOESM5_ESM.zip › Figure 3/M/Ndi1 5mC R1.tif]

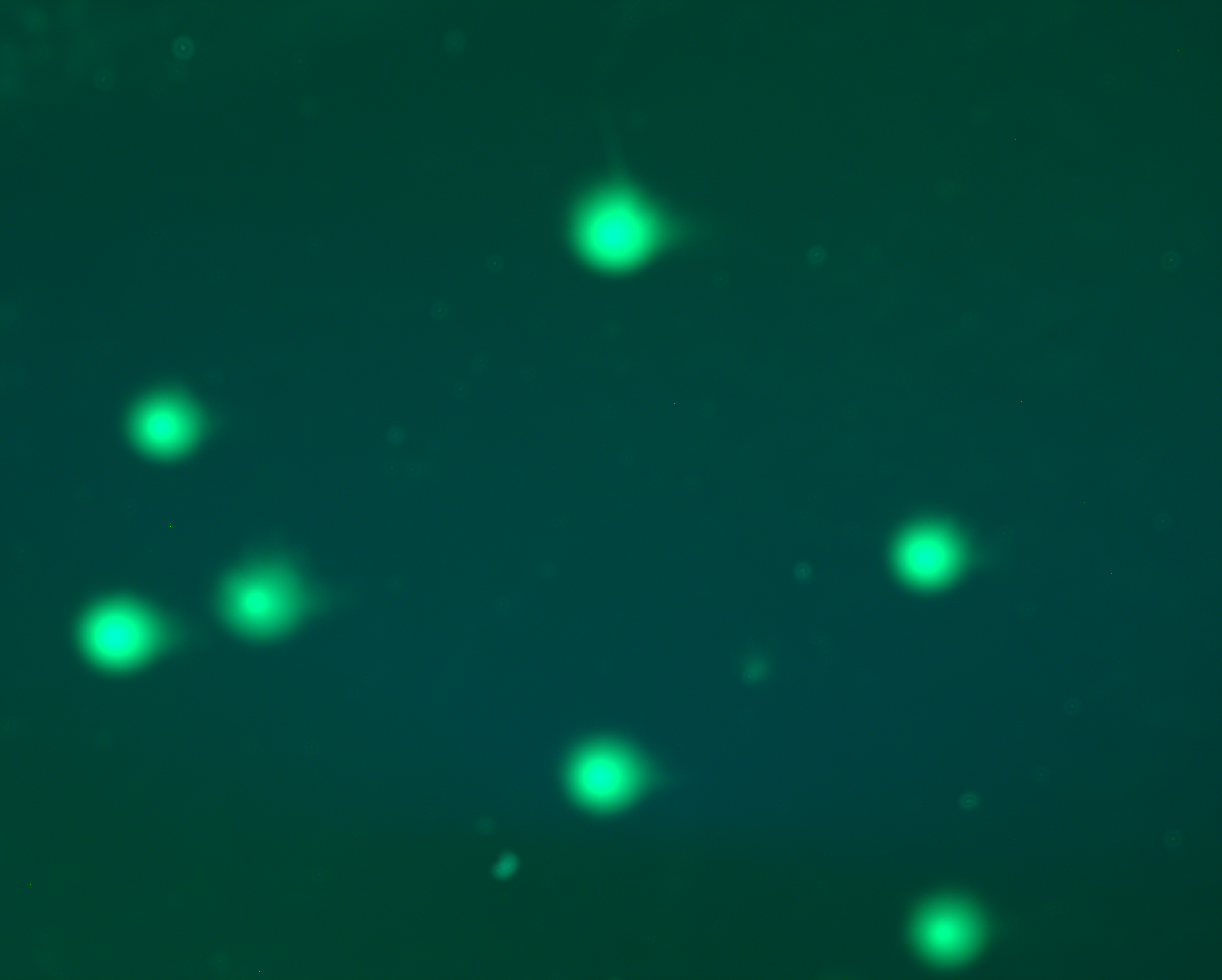

Supplement: Supplementary file 5 — Source data Fig. 3 [file 44318_2026_784_MOESM5_ESM.zip › Figure 3/N/Ctrl.tif]

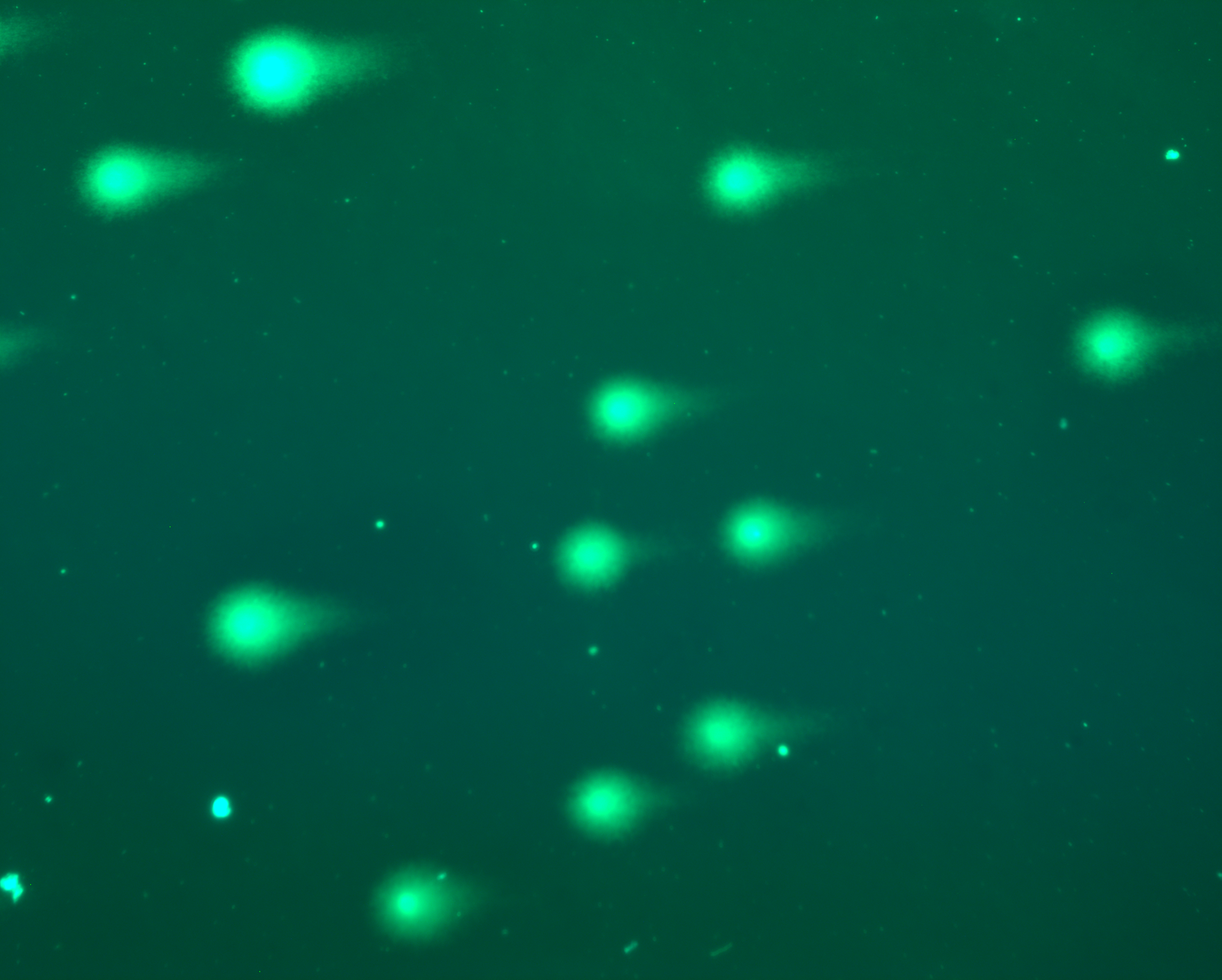

Supplement: Supplementary file 5 — Source data Fig. 3 [file 44318_2026_784_MOESM5_ESM.zip › Figure 3/N/Ndi1.tif]

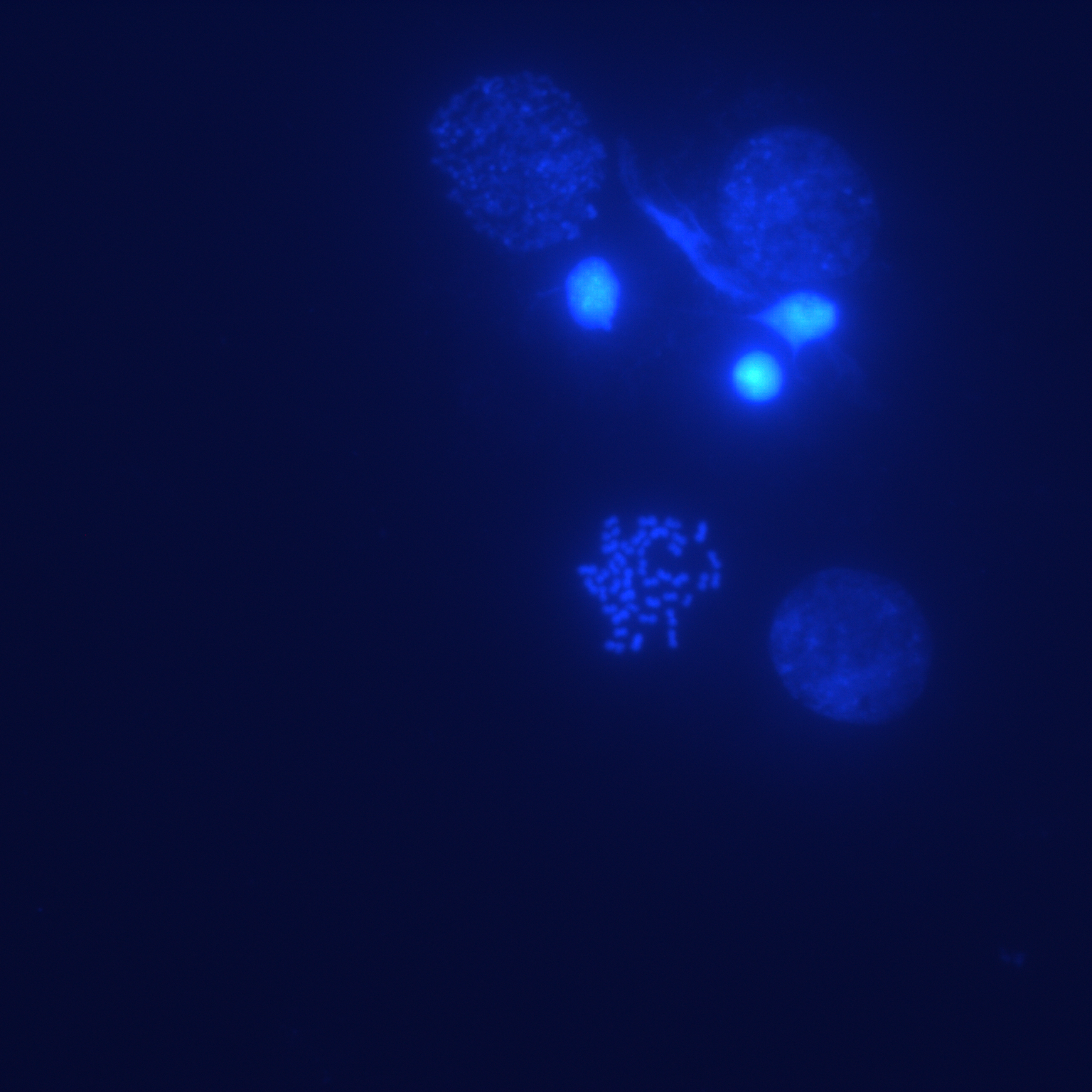

Supplement: Supplementary file 5 — Source data Fig. 3 [file 44318_2026_784_MOESM5_ESM.zip › Figure 3/O/Ctrl.tif]

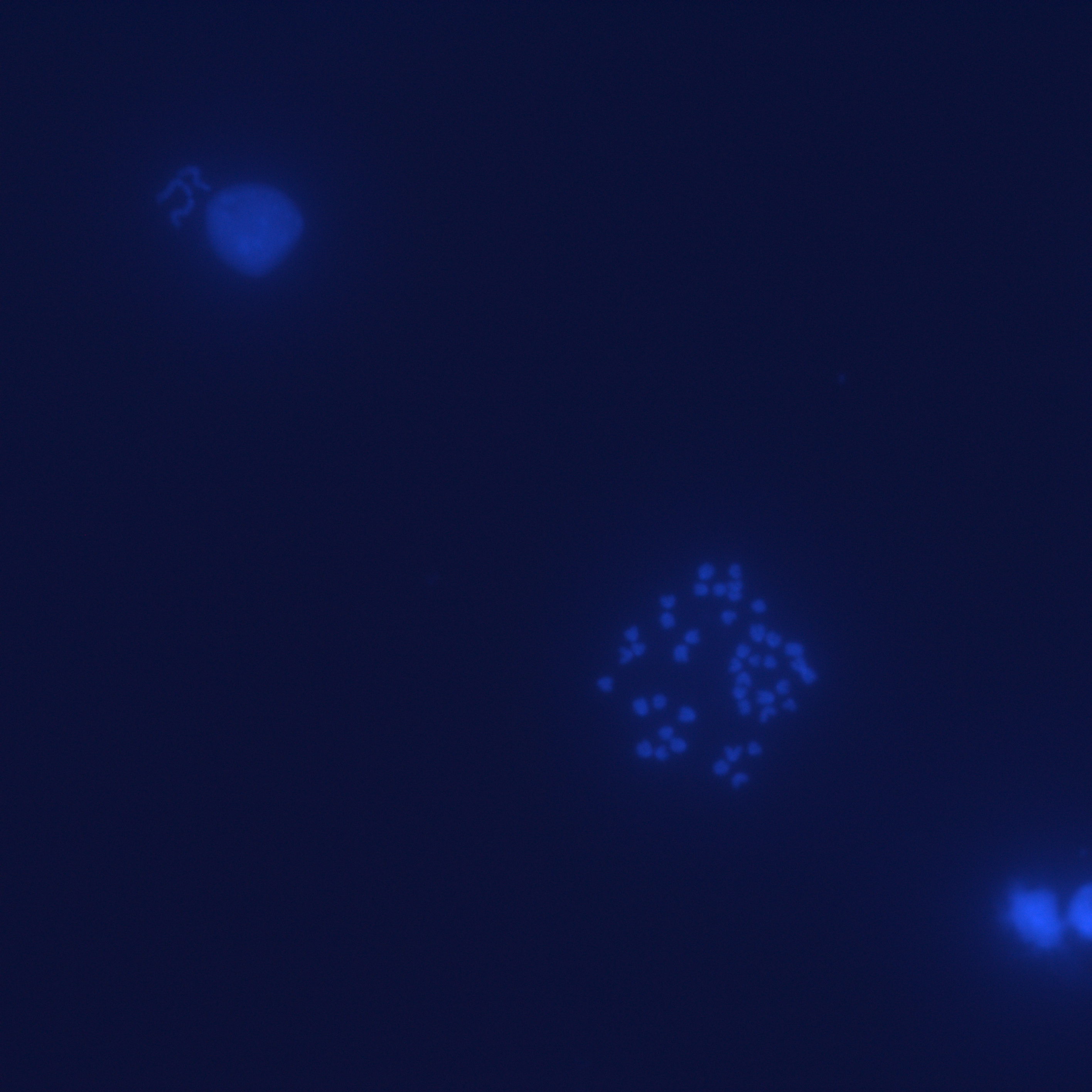

Supplement: Supplementary file 5 — Source data Fig. 3 [file 44318_2026_784_MOESM5_ESM.zip › Figure 3/O/Ndi1.tif]

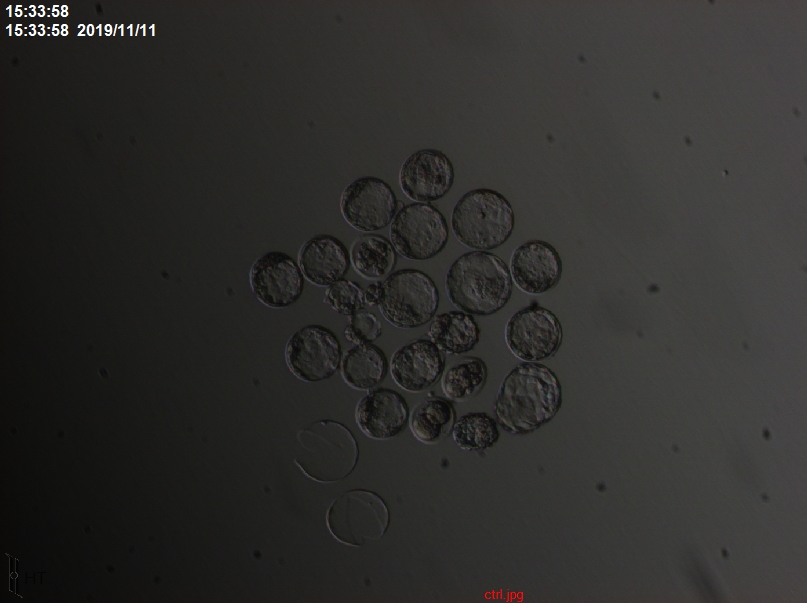

Supplement: Supplementary file 6 — Source data Fig. 4 [file 44318_2026_784_MOESM6_ESM.zip › Figure 4/B/120hrs/Rot 0nM.jpg]

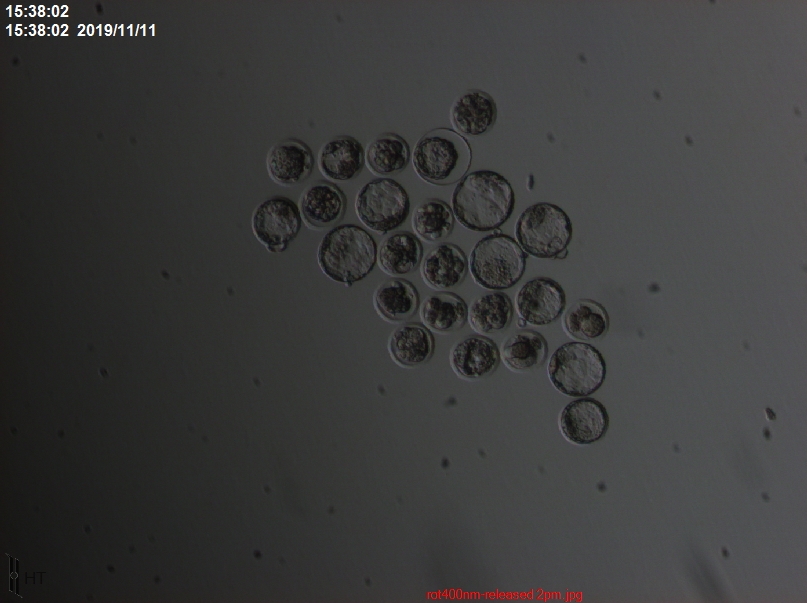

Supplement: Supplementary file 6 — Source data Fig. 4 [file 44318_2026_784_MOESM6_ESM.zip › Figure 4/B/120hrs/Rot 400nM-released.jpg]

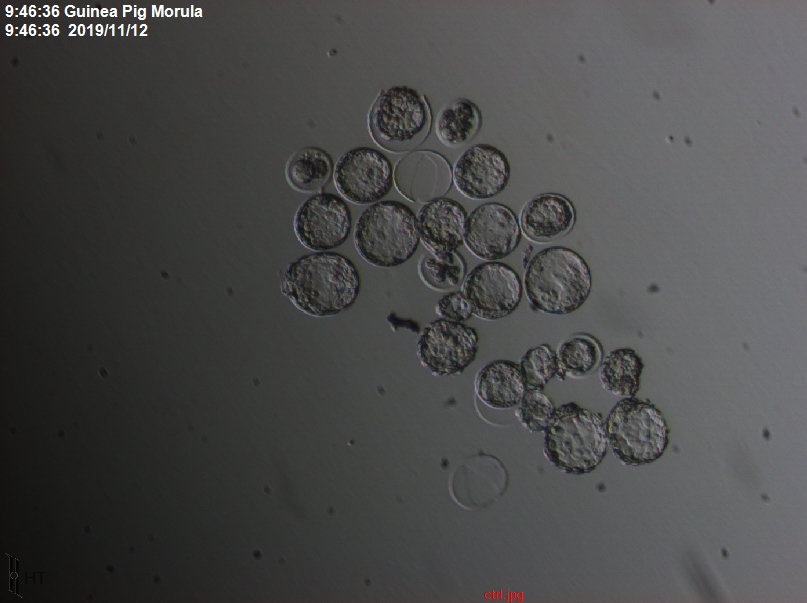

Supplement: Supplementary file 6 — Source data Fig. 4 [file 44318_2026_784_MOESM6_ESM.zip › Figure 4/B/144hrs/Rot 0nM.jpg]

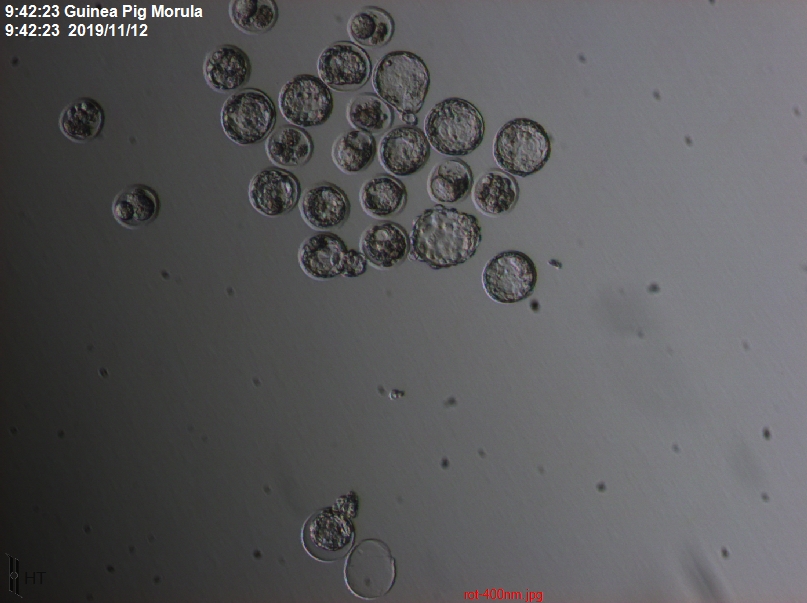

Supplement: Supplementary file 6 — Source data Fig. 4 [file 44318_2026_784_MOESM6_ESM.zip › Figure 4/B/144hrs/Rot 400nM-released.jpg]

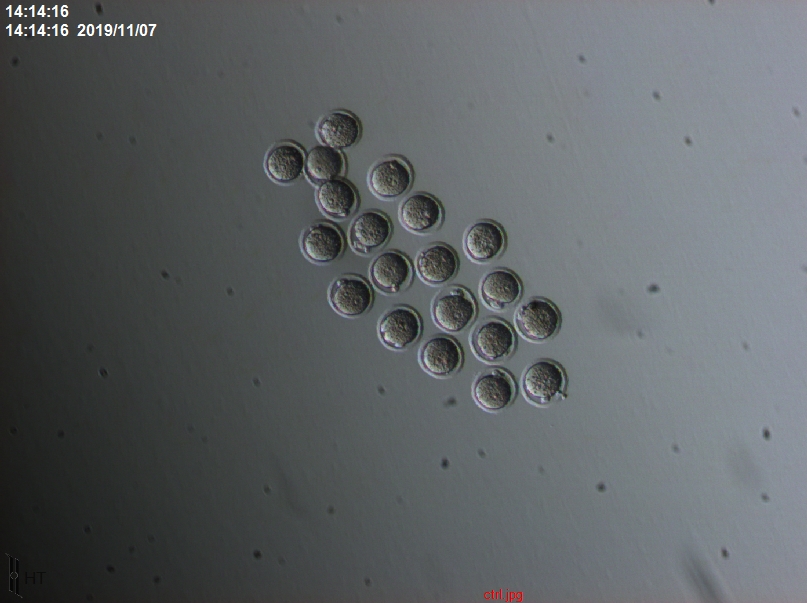

Supplement: Supplementary file 6 — Source data Fig. 4 [file 44318_2026_784_MOESM6_ESM.zip › Figure 4/B/24hrs/Rot 0nM.jpg]

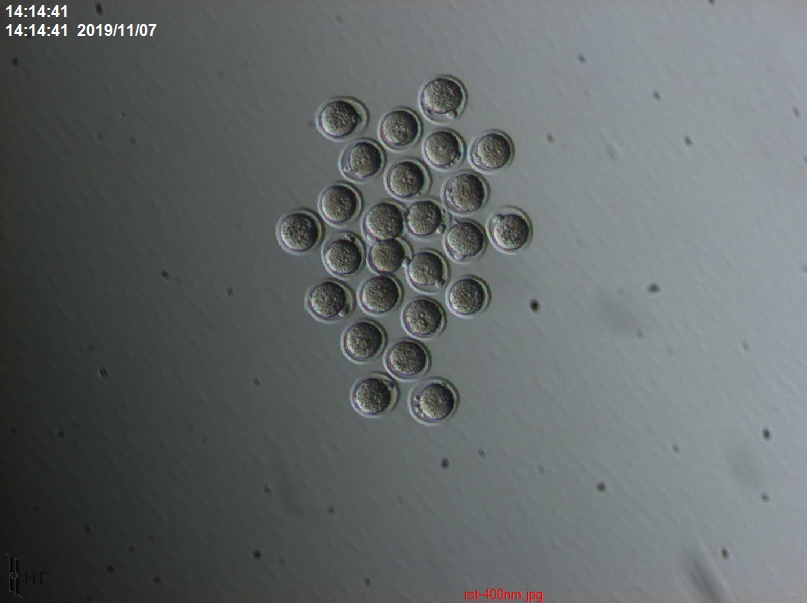

Supplement: Supplementary file 6 — Source data Fig. 4 [file 44318_2026_784_MOESM6_ESM.zip › Figure 4/B/24hrs/Rot 400nM.jpg]

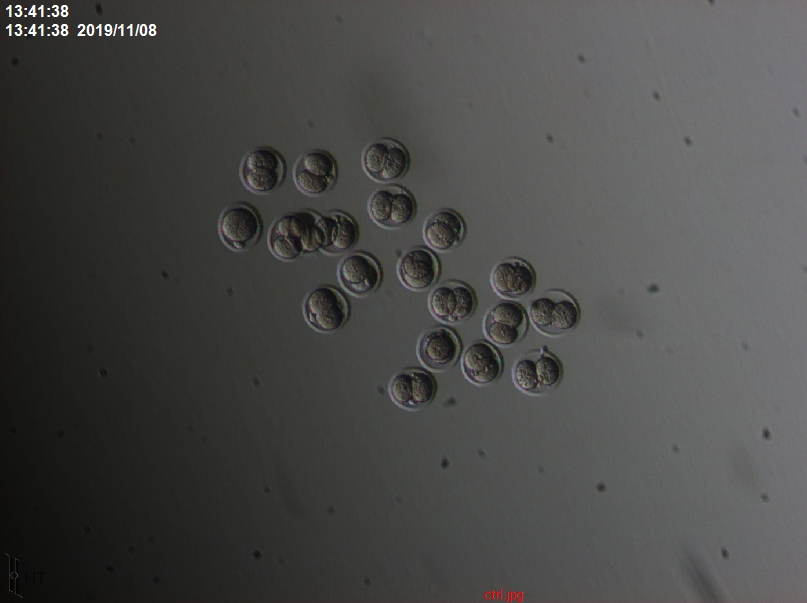

Supplement: Supplementary file 6 — Source data Fig. 4 [file 44318_2026_784_MOESM6_ESM.zip › Figure 4/B/48hrs/Rot 0nM.jpg]

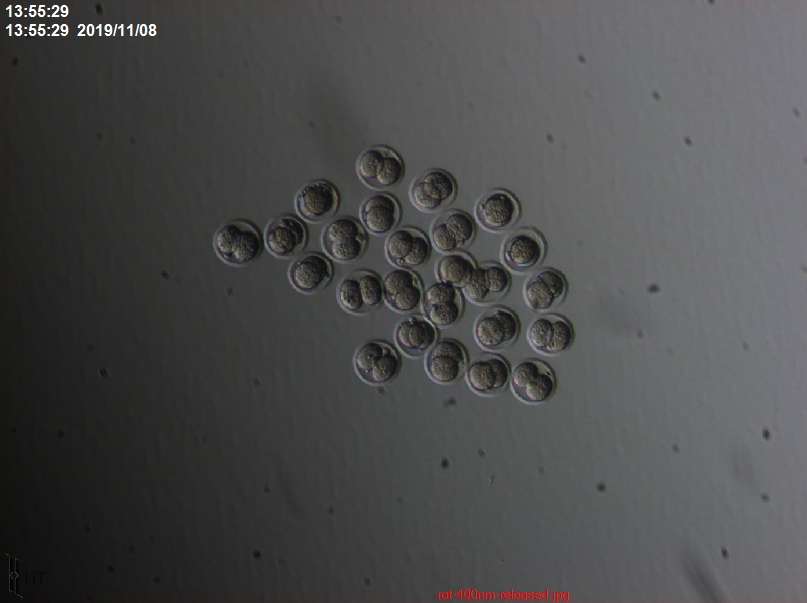

Supplement: Supplementary file 6 — Source data Fig. 4 [file 44318_2026_784_MOESM6_ESM.zip › Figure 4/B/48hrs/Rot 400nM.jpg]

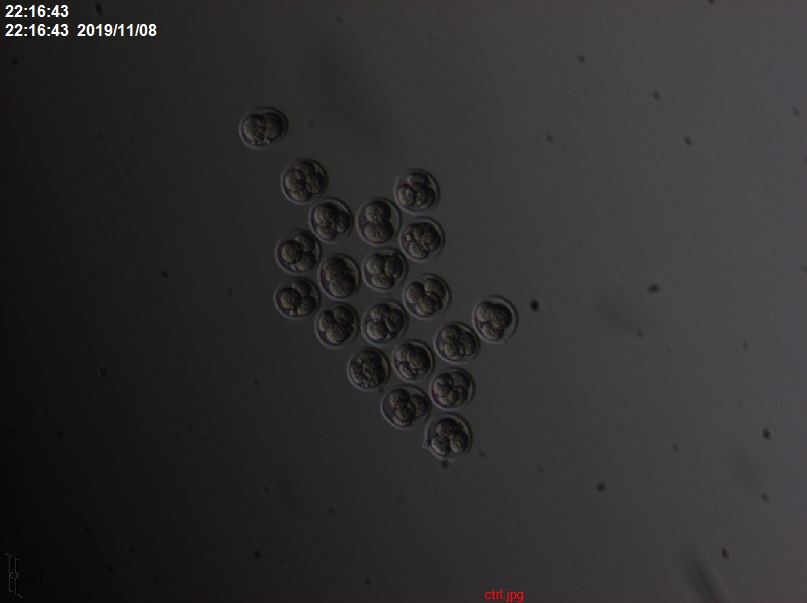

Supplement: Supplementary file 6 — Source data Fig. 4 [file 44318_2026_784_MOESM6_ESM.zip › Figure 4/B/56hrs/Rot 0nM.jpg]

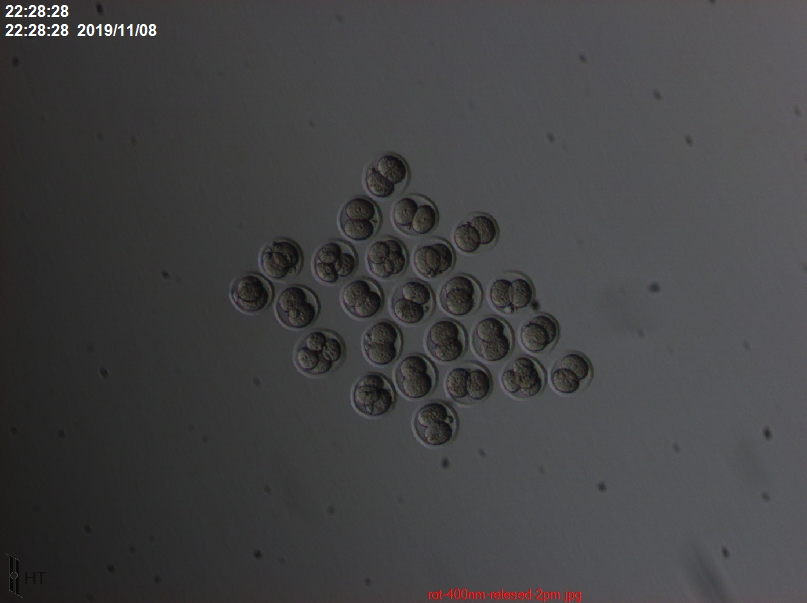

Supplement: Supplementary file 6 — Source data Fig. 4 [file 44318_2026_784_MOESM6_ESM.zip › Figure 4/B/56hrs/Rot 400nM-relesed.jpg]

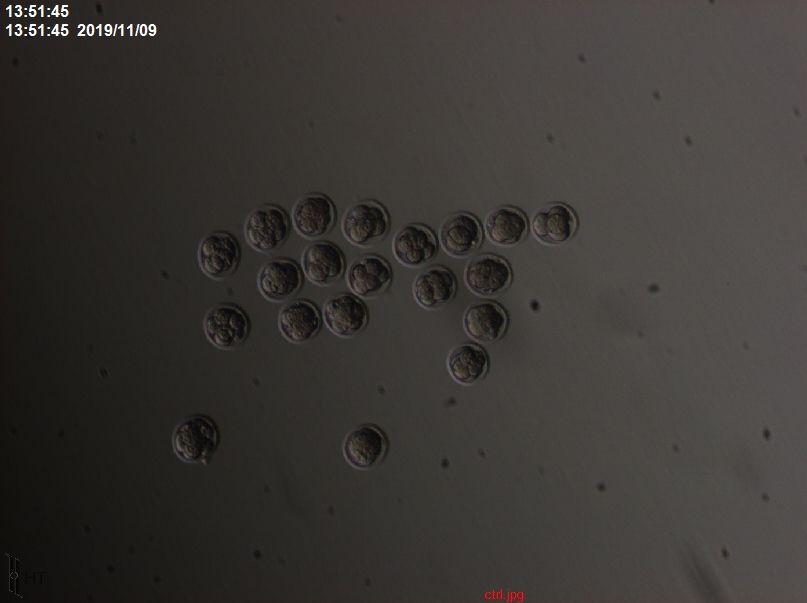

Supplement: Supplementary file 6 — Source data Fig. 4 [file 44318_2026_784_MOESM6_ESM.zip › Figure 4/B/72hrs/Rot 0nM.jpg]

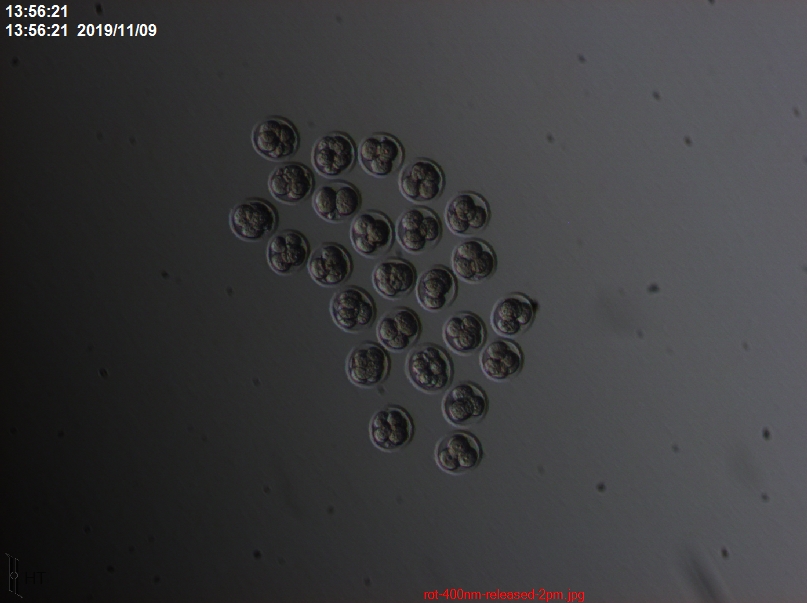

Supplement: Supplementary file 6 — Source data Fig. 4 [file 44318_2026_784_MOESM6_ESM.zip › Figure 4/B/72hrs/Rot 400nM-released.jpg]

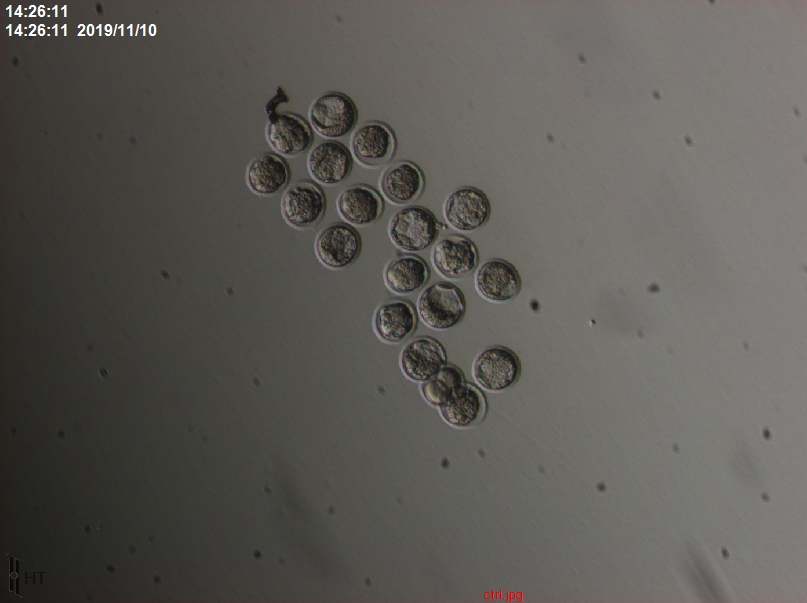

Supplement: Supplementary file 6 — Source data Fig. 4 [file 44318_2026_784_MOESM6_ESM.zip › Figure 4/B/96hrs/Rot 0nM.jpg]

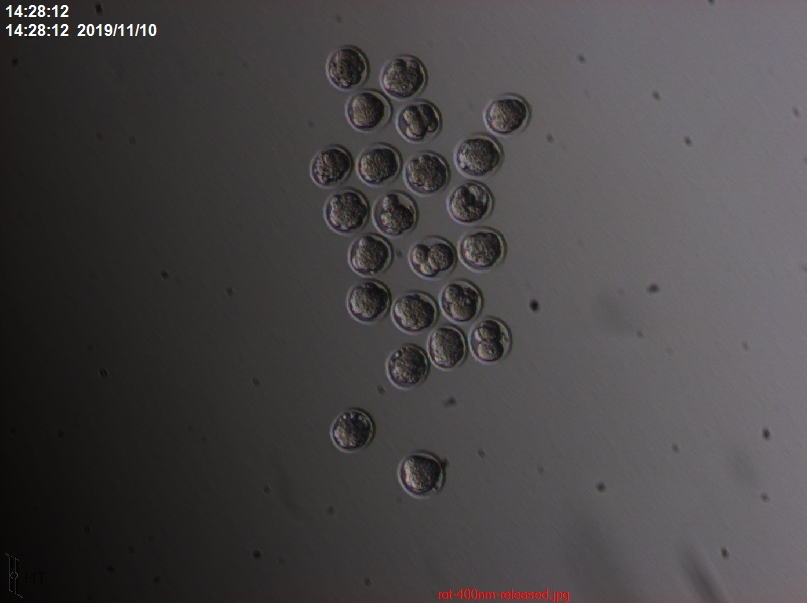

Supplement: Supplementary file 6 — Source data Fig. 4 [file 44318_2026_784_MOESM6_ESM.zip › Figure 4/B/96hrs/Rot 400nM-released.jpg]

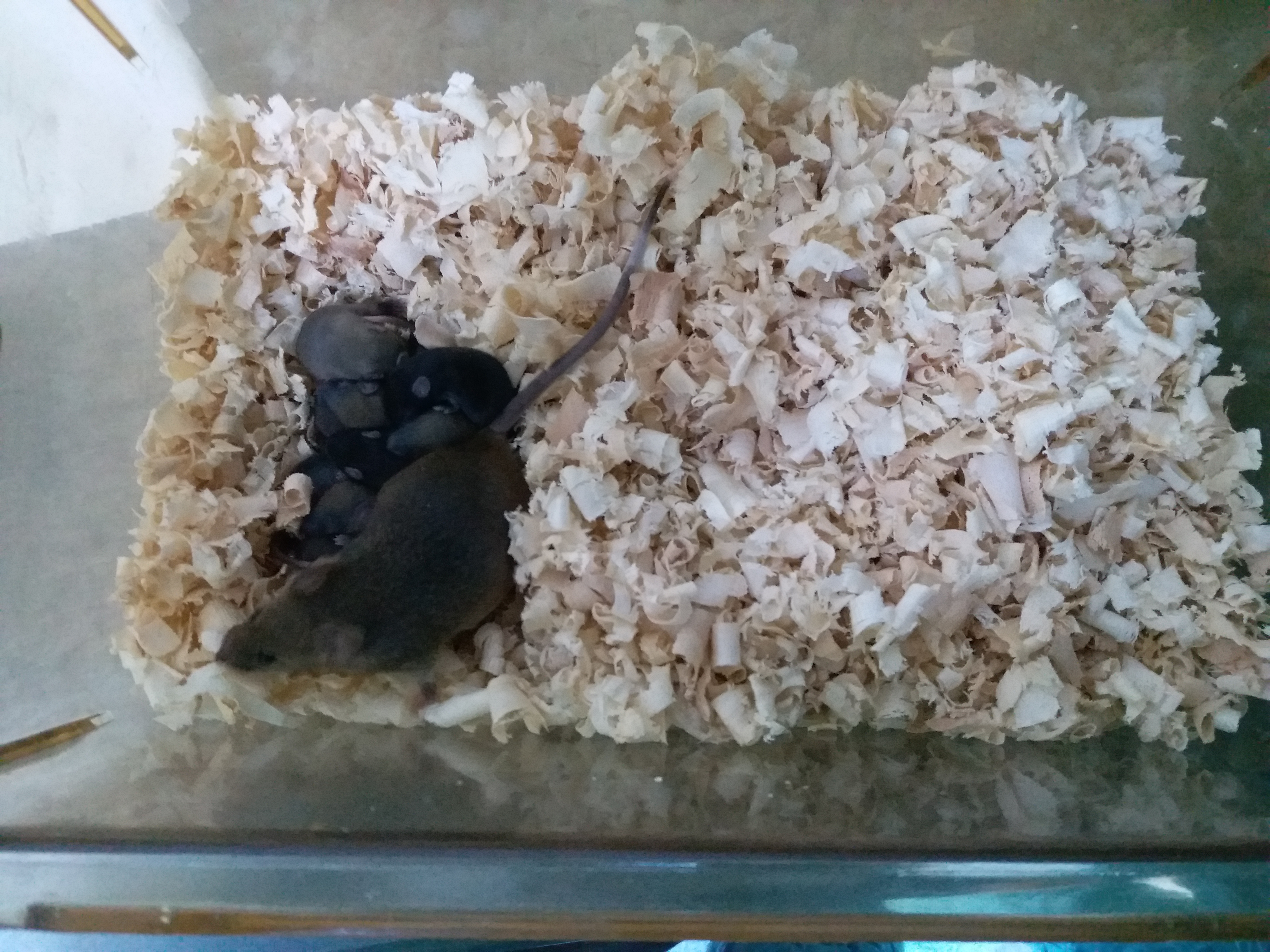

Supplement: Supplementary file 6 — Source data Fig. 4 [file 44318_2026_784_MOESM6_ESM.zip › Figure 4/D/F1 from Rot 400nM-relesed.jpg]

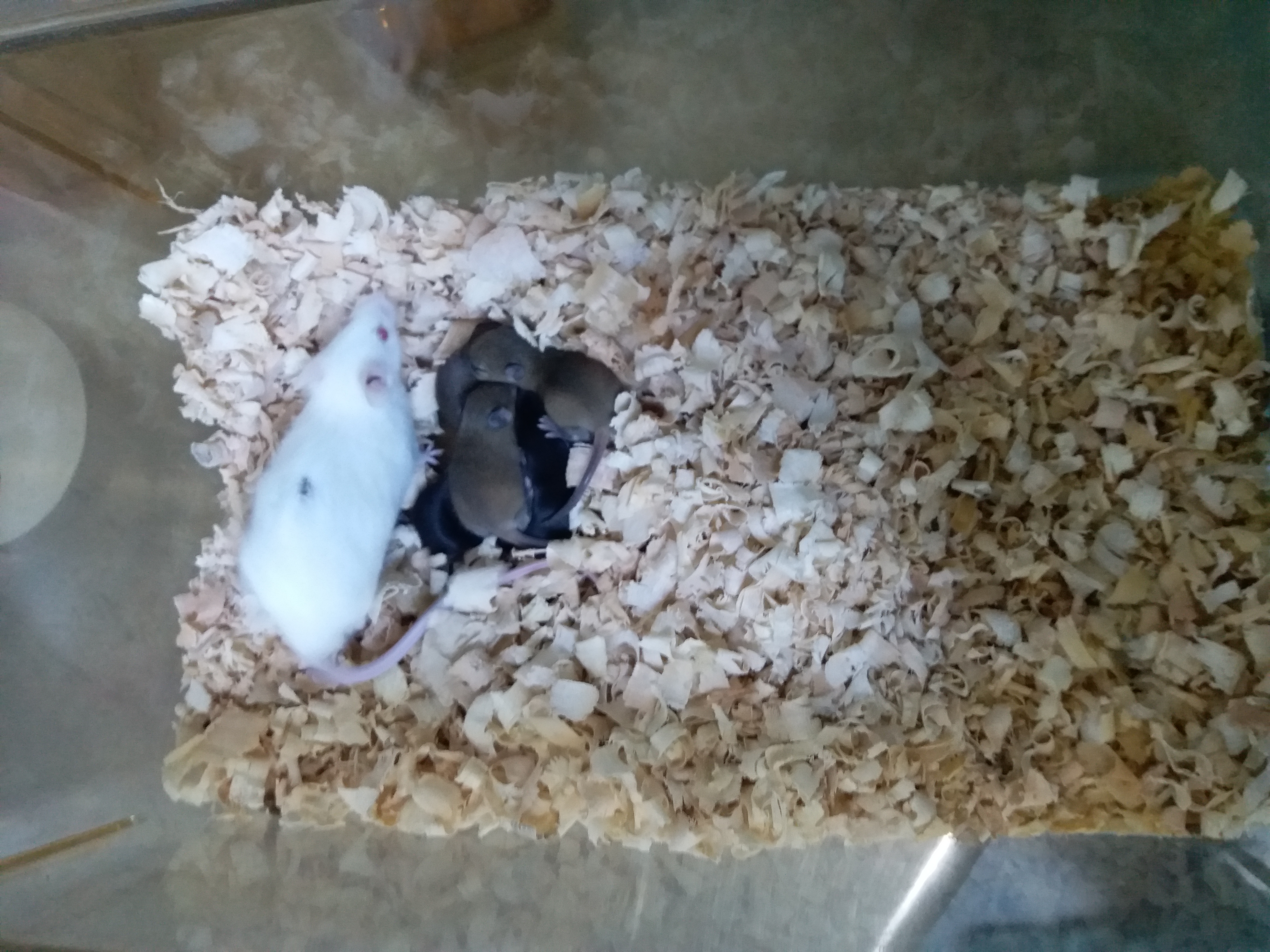

Supplement: Supplementary file 6 — Source data Fig. 4 [file 44318_2026_784_MOESM6_ESM.zip › Figure 4/D/Rot 400nM-relesed.jpg]

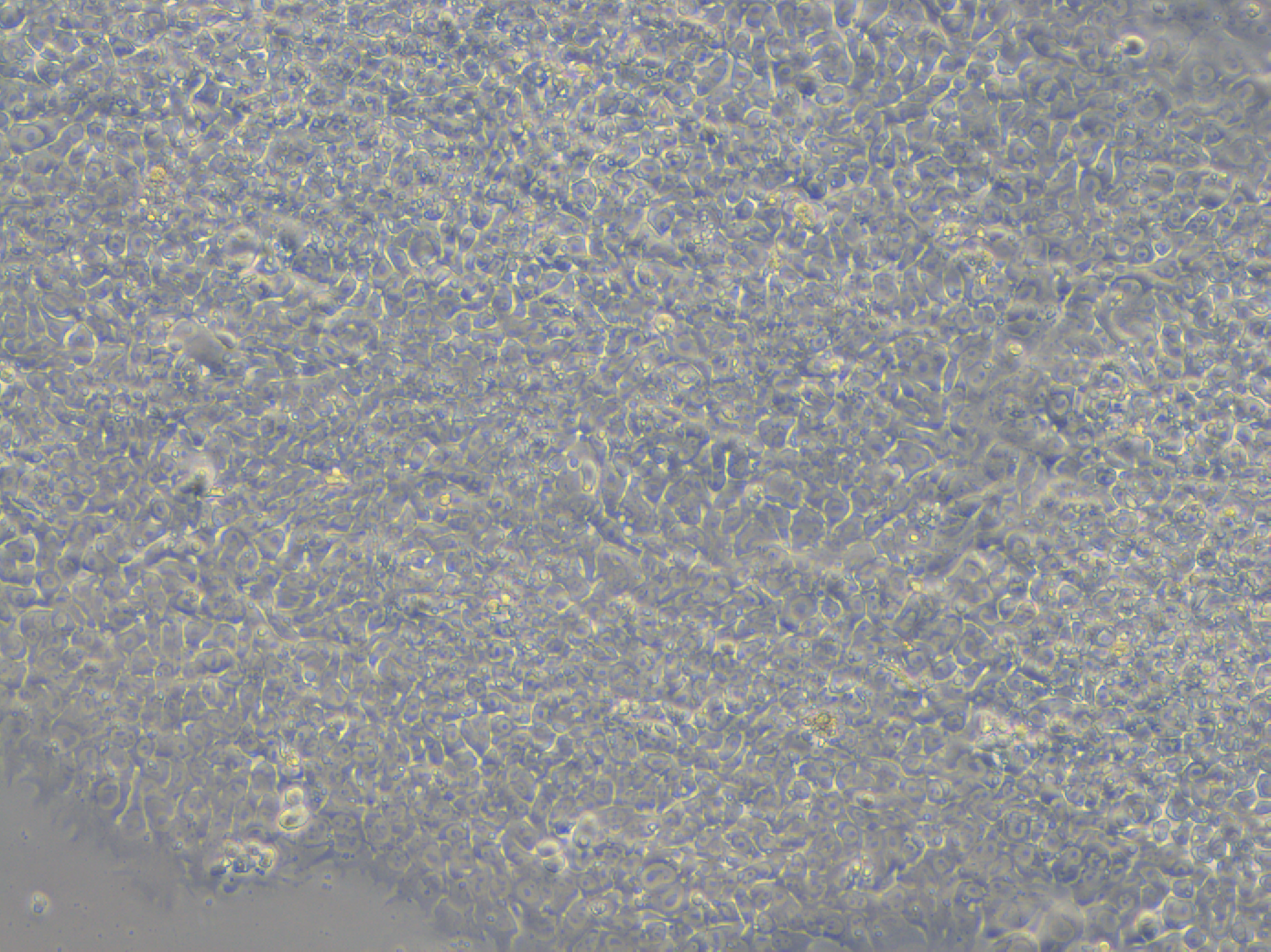

Supplement: Supplementary file 6 — Source data Fig. 4 [file 44318_2026_784_MOESM6_ESM.zip › Figure 4/E/Ant Release 3 days.tiff]

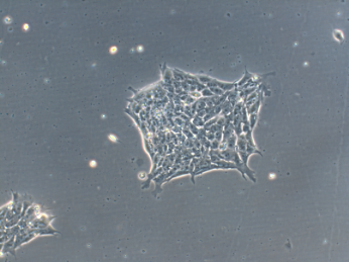

Supplement: Supplementary file 6 — Source data Fig. 4 [file 44318_2026_784_MOESM6_ESM.zip › Figure 4/E/Ant.tif]

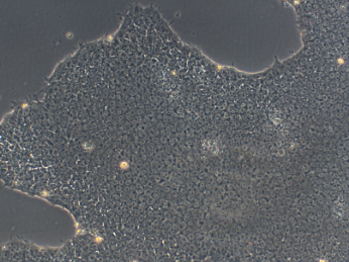

Supplement: Supplementary file 6 — Source data Fig. 4 [file 44318_2026_784_MOESM6_ESM.zip › Figure 4/E/Ctrl.tif]

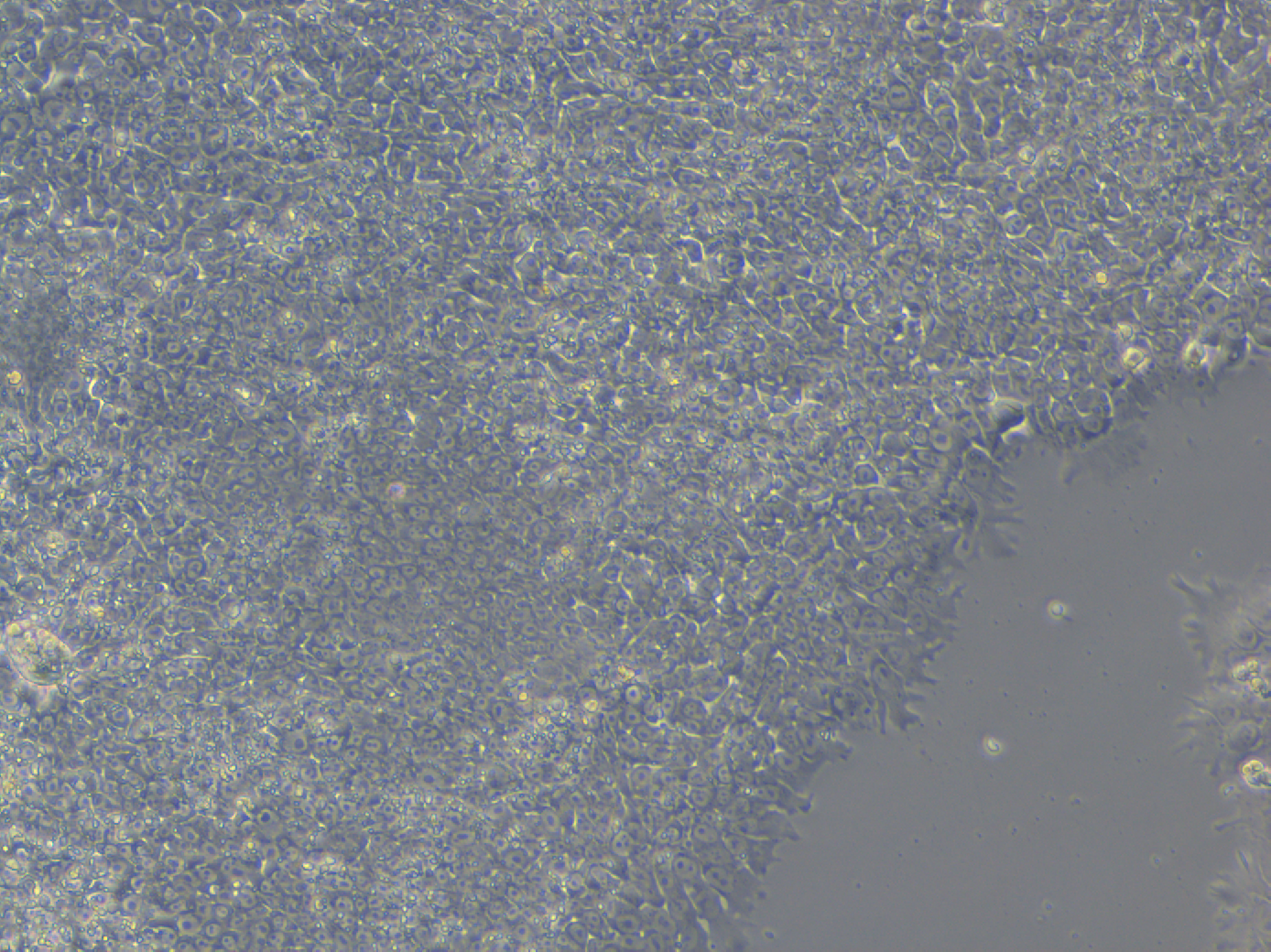

Supplement: Supplementary file 6 — Source data Fig. 4 [file 44318_2026_784_MOESM6_ESM.zip › Figure 4/E/Rot Released 3 days.tiff]

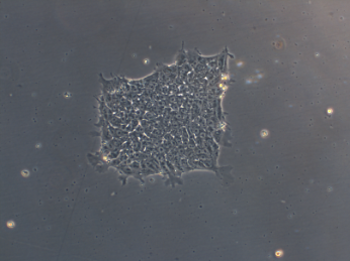

Supplement: Supplementary file 6 — Source data Fig. 4 [file 44318_2026_784_MOESM6_ESM.zip › Figure 4/E/Rot.tif]

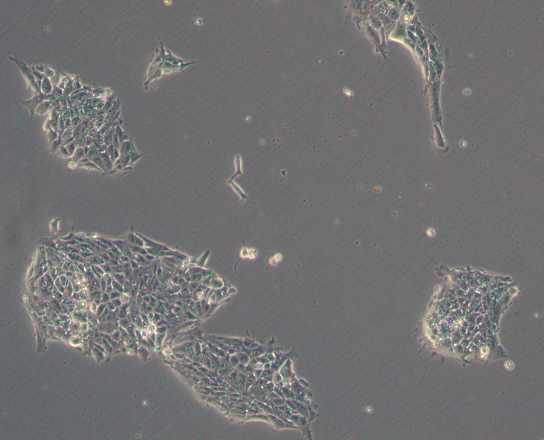

Supplement: Supplementary file 6 — Source data Fig. 4 [file 44318_2026_784_MOESM6_ESM.zip › Figure 4/H/Ctrl +Rot.tiff]

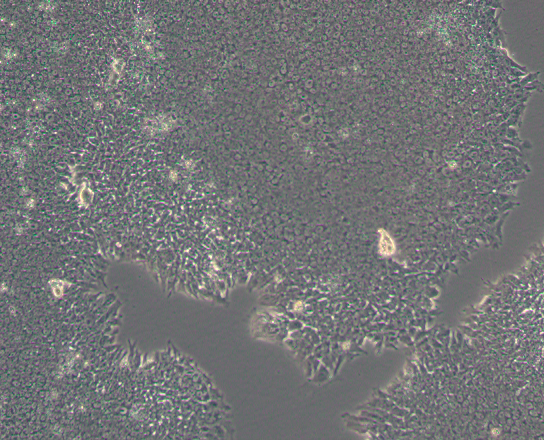

Supplement: Supplementary file 6 — Source data Fig. 4 [file 44318_2026_784_MOESM6_ESM.zip › Figure 4/H/Ctrl -Rot.tiff]

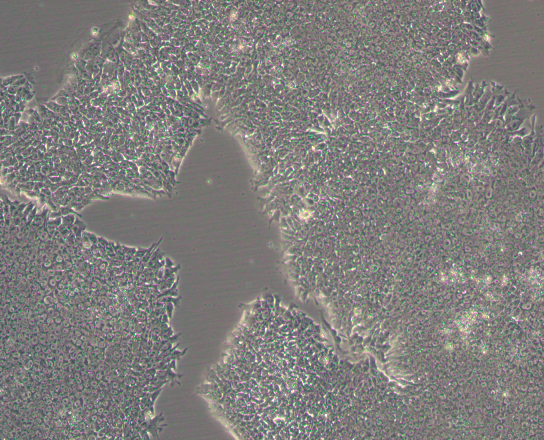

Supplement: Supplementary file 6 — Source data Fig. 4 [file 44318_2026_784_MOESM6_ESM.zip › Figure 4/H/Ndi1 -Rot.tiff]

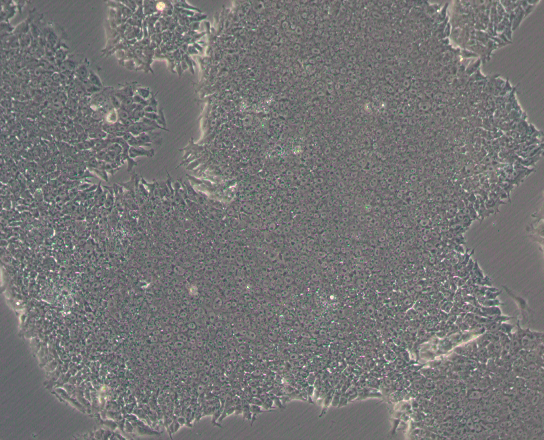

Supplement: Supplementary file 6 — Source data Fig. 4 [file 44318_2026_784_MOESM6_ESM.zip › Figure 4/H/Ndi1+Rot.tiff]

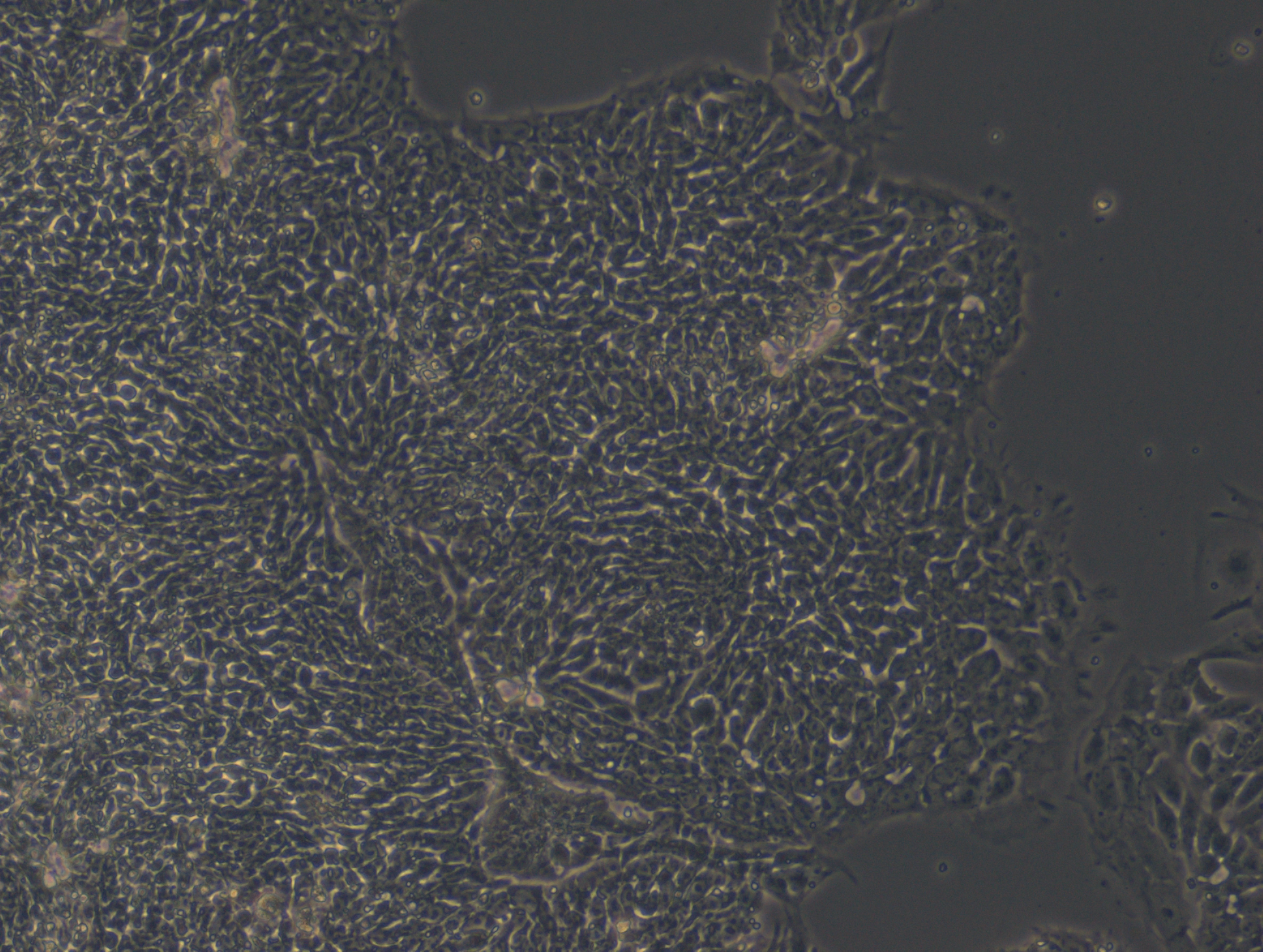

Supplement: Supplementary file 6 — Source data Fig. 4 [file 44318_2026_784_MOESM6_ESM.zip › Figure 4/L/Ctrl -Rot.tif]

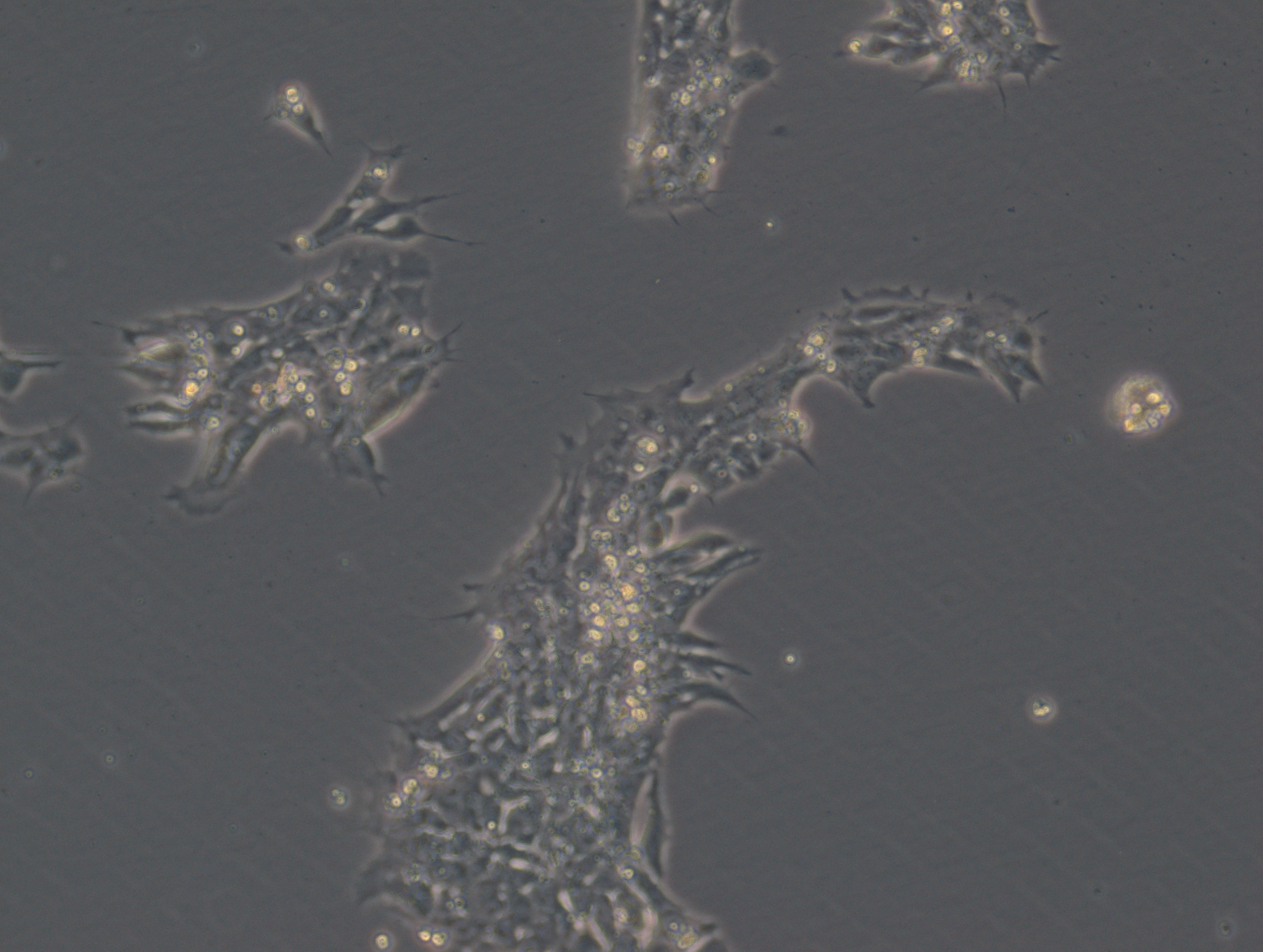

Supplement: Supplementary file 6 — Source data Fig. 4 [file 44318_2026_784_MOESM6_ESM.zip › Figure 4/L/Ctrl+Rot.tif]

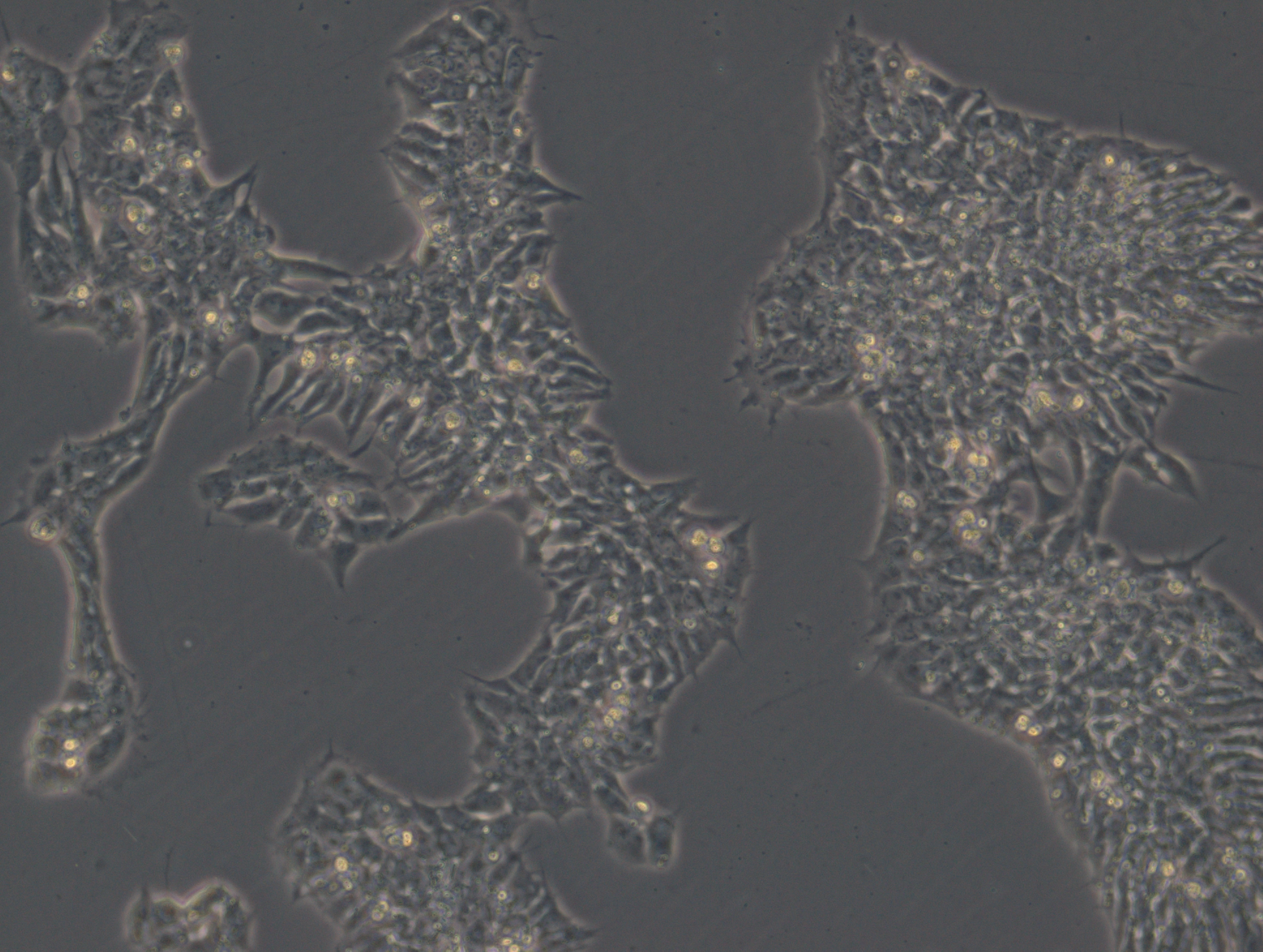

Supplement: Supplementary file 6 — Source data Fig. 4 [file 44318_2026_784_MOESM6_ESM.zip › Figure 4/L/DS+SHIN1+Rot.tif]

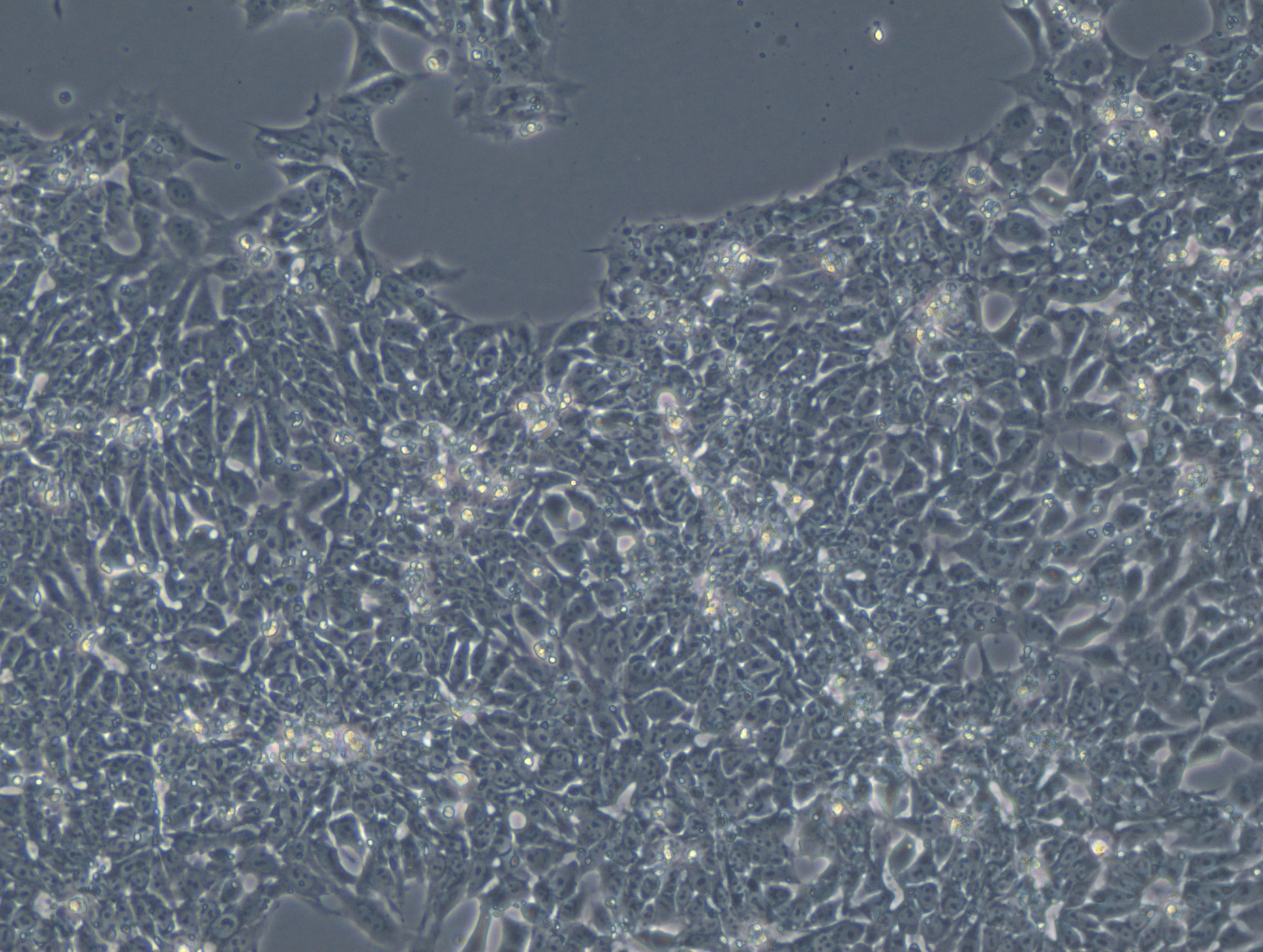

Supplement: Supplementary file 6 — Source data Fig. 4 [file 44318_2026_784_MOESM6_ESM.zip › Figure 4/L/DS+SHIN1-Rot.tif]
