# Supplementary material for: Mitochondrial NADH-redox inflexibility constrains genomic and epigenetic stability in pluripotent stem cells
Source: EMBO J. 2026 May 27;45(13):4417–46. doi: 10.1038/s44318-026-00784-2 (PMC13324003; doi:10.1038/s44318-026-00784-2)
Supplement: Supplementary file 7 — Expanded View Figures [file 44318_2026_784_MOESM7_ESM.pdf]

Expanded View Figures

Figure EV1. TDH sustains reductive stress and contributes to ETC C1 inhibition-induced pausing in mESCs, related to Fig. 1.

(**A**) Schematic depiction of ETC complex (C1-5) and their functions with respective complex specific inhibitors. (**B**, **C**) Cell images (**B**) and cell number (**C**) of E14 mESCs treated with rotenone (Rot 150 nM), dimethyl malonate (DMM 15 µM), antimycin A (Ant 10 nM), sodium azide (NaN3 500 µM), and oligomycin A (Oligo 10 nM) for 4 days in 2iL and released for 4 days (mean ± SD, $n = 3$ each with biological replicates), scale bar, 200 µm. (**D**) Image of chimeric mice from Rot-induced paused mESCs for 4 days and released for 4 days. (**E**) Relative cell number and relative SoNar levels of E14 mESCs treated with Rot or Ant with and without pyruvate or AKB in pyruvate-free medium for 4 days (mean ± SD, $n = 3$ each with biological replicates). (**F**) Relative cell number of 3T3 treated with Rot (200 nM) or Ant (1 µM) with or without dimethyl-aspartate (Asp 10 mM) for 4 days in MEF medium (mean ± SD, $n = 3$ each with biological replicates). (**G**) Heat maps showing relative differential metabolites from E14 mESCs treated with Rot or Ant for 3 days. (**H**) Western blot analysis from 3T3 and E14 mESCs transduced with Flag, or cyto-*Lb*NOX or mito-*Lb*NOX. ACTIN was used as a loading control. (**I, J**) Relative cell number (**I**) and relative SoNar levels (**J**) from 3T3 cells transduced with Flag and cyto-*Lb*NOX or mito-*Lb*NOX treated with or without Rot or Ant for 4 days (mean ± SD, $n = 3$ each with biological replicates). (**K**, **L**) Relative cell number (**K**) and relative SoNar levels (**L**) from E14 cells transduced with Flag or cyto-*Lb*NOX or mito-*Lb*NOX treated with or without Rot or Ant for 4 days (mean ± SD, $n = 3$ each with biological replicates). (**M**, **N**) Relative cell number (**M**) and relative SoNar levels (**N**) from E14 mESCs treated with Rot or Ant and with and without lactate (10 mM) for 4 days (mean ± SD, $n = 3$ each with biological replicates). (**O–R**) Relative cell number (**O**) and relative SoNar levels of 3T3 (**P**), relative cell number (**Q**), and relative SoNar levels of E14 (**R**), treated with the indicated treatments for 4 days (mean ± SD, $n = 3$ each with biological replicates). Data information, in Fig. EV1E–H, K–R, columns represent mean, error bars are standard deviation, points are individual biological replicates; lines indicate comparisons between different groups; stars indicate statistical significance. *$p < 0.05$, **$p < 0.01$, ***$p < 0.001$, ****$p < 0.0001$; ns, not significant by unpaired two-tailed Student's *t*-test. Individual *p* values are provided in Table EV1.

© The Author(s)

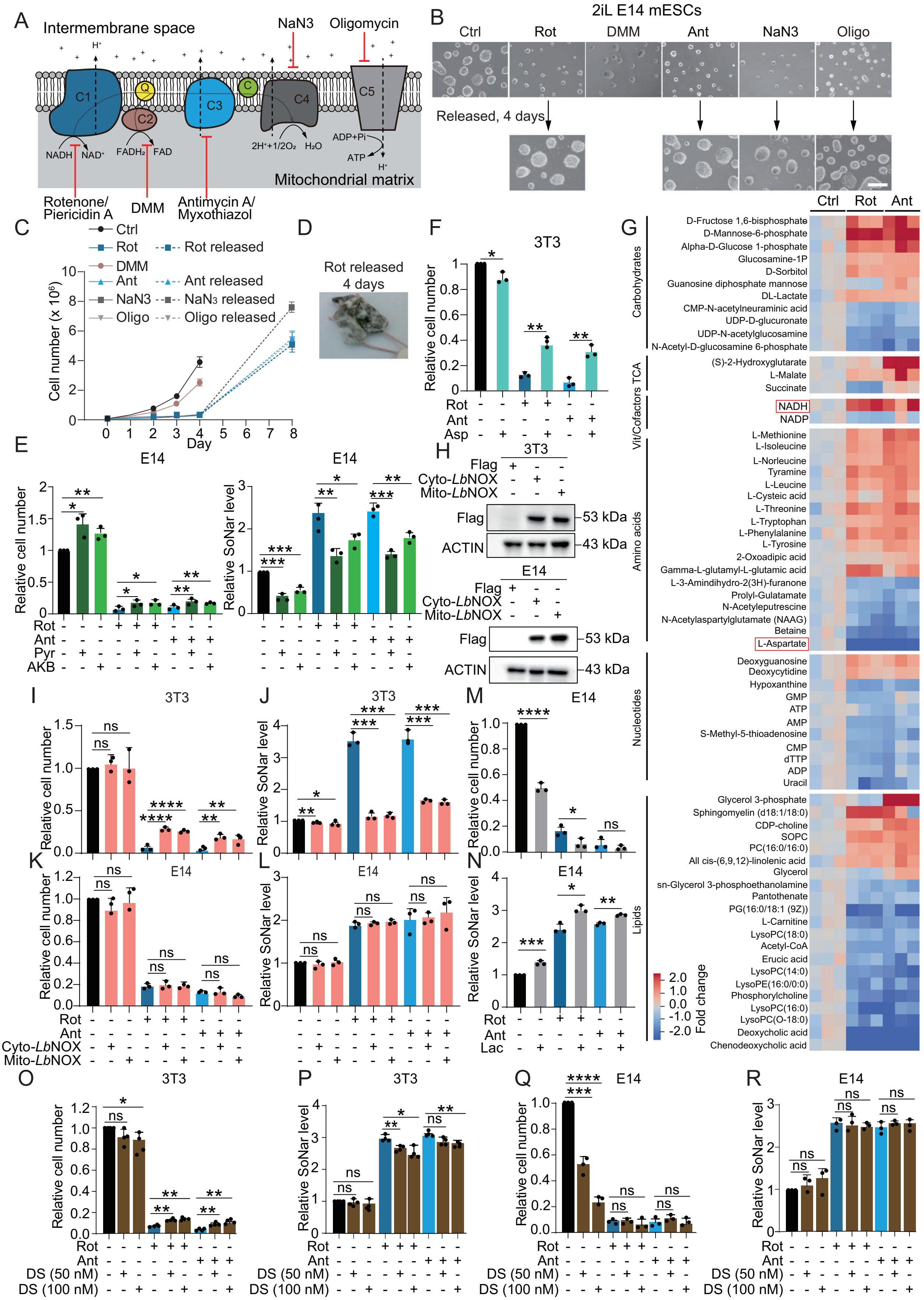
A
Intermembrane space
NaN3
Oligomycin
C1
Q
C2
C3
C
C4
C5
NADH
NAD+
FADH2
FAD
2H++1/2O2
H2O
ADP+Pi
ATP
Mitochondrial matrix
Rotenone/
Piericidin A
DMM
Antimycin A/
Myxothiazol
B
2iL E14 mESCs
Ctrl
Rot
DMM
Ant
NaN3
Oligo
Released, 4 days
C
Ctrl
Rot
Rot released
DMM
Ant
Ant released
NaN3
NaN3 released
Oligo
Oligo released
Cell number (x 10^6)
Day
D
Rot released
4 days
E
E14
Relative cell number
Relative SoNar level
Rot
Ant
Pyr
AKB
F
3T3
Relative cell number
Rot
Ant
Asp
G
Ctrl
Rot
Ant
Carbohydrates
D-Fructose 1,6-bisphosphate
D-Mannose-6-phosphate
Alpha-D-Glucose 1-phosphate
Glucosamine-1P
D-Sorbitol
Guanosine diphosphate mannose
DL-Lactate
CMP-N-acetylneuraminic acid
UDP-D-glucuronate
UDP-N-acetylglucosamine
N-Acetyl-D-glucosamine 6-phosphate
TCA
(S)-2-Hydroxyglutarate
L-Malate
Succinate
Vit/Cofactors
NADH
NADP
Amino acids
L-Methionine
L-Isoleucine
L-Norleucine
Tyramine
L-Leucine
L-Cysteic acid
L-Threonine
L-Tryptophan
L-Phenylalanine
L-Tyrosine
2-Oxoadipic acid
Gamma-L-glutamyl-L-glutamic acid
L-3-Amindihydro-2(3H)-furanone
Prolyl-Gulatamate
N-Acetyleputrescine
N-Acetylaspartylglutamate (NAAG)
Betaine
L-Aspartate
Nucleotides
Deoxyguanosine
Deoxycytidine
Hypoxanthine
GMP
ATP
AMP
S-Methyl-5-thioadenosine
CMP
dTTP
ADP
Uracil
Lipids
Glycerol 3-phosphate
Sphingomyelin (d18:1/18:0)
CDP-choline
SOPC
PC(16:0/16:0)
All cis-(6,9,12)-linolenic acid
Glycerol
sn-Glycerol 3-phosphoethanolamine
Pantothenate
PG(16:0/18:1 (9Z))
L-Carnitine
LysoPC(18:0)
Acetyl-CoA
Erucic acid
LysoPC(14:0)
LysoPE(16:0/0:0)
Phosphorylcholine
LysoPC(16:0)
LysoPC(O-18:0)
Deoxycholic acid
Chenodeoxycholic acid
Fold change
H
3T3
Flag
Cyto-LbNOX
Mito-LbNOX
ACTIN
53 kDa
43 kDa
E14
I
3T3
Relative cell number
J
3T3
Relative SoNar level
M
E14
Relative cell number
K
E14
Relative cell number
L
E14
Relative SoNar level
N
E14
Relative SoNar level
Rot
Ant
Cyto-LbNOX
Mito-LbNOX
Lac
O
3T3
Relative cell number
P
3T3
Relative SoNar level
Q
E14
Relative cell number
R
E14
Relative SoNar level
DS (50 nM)
DS (100 nM)

© The Author(s)

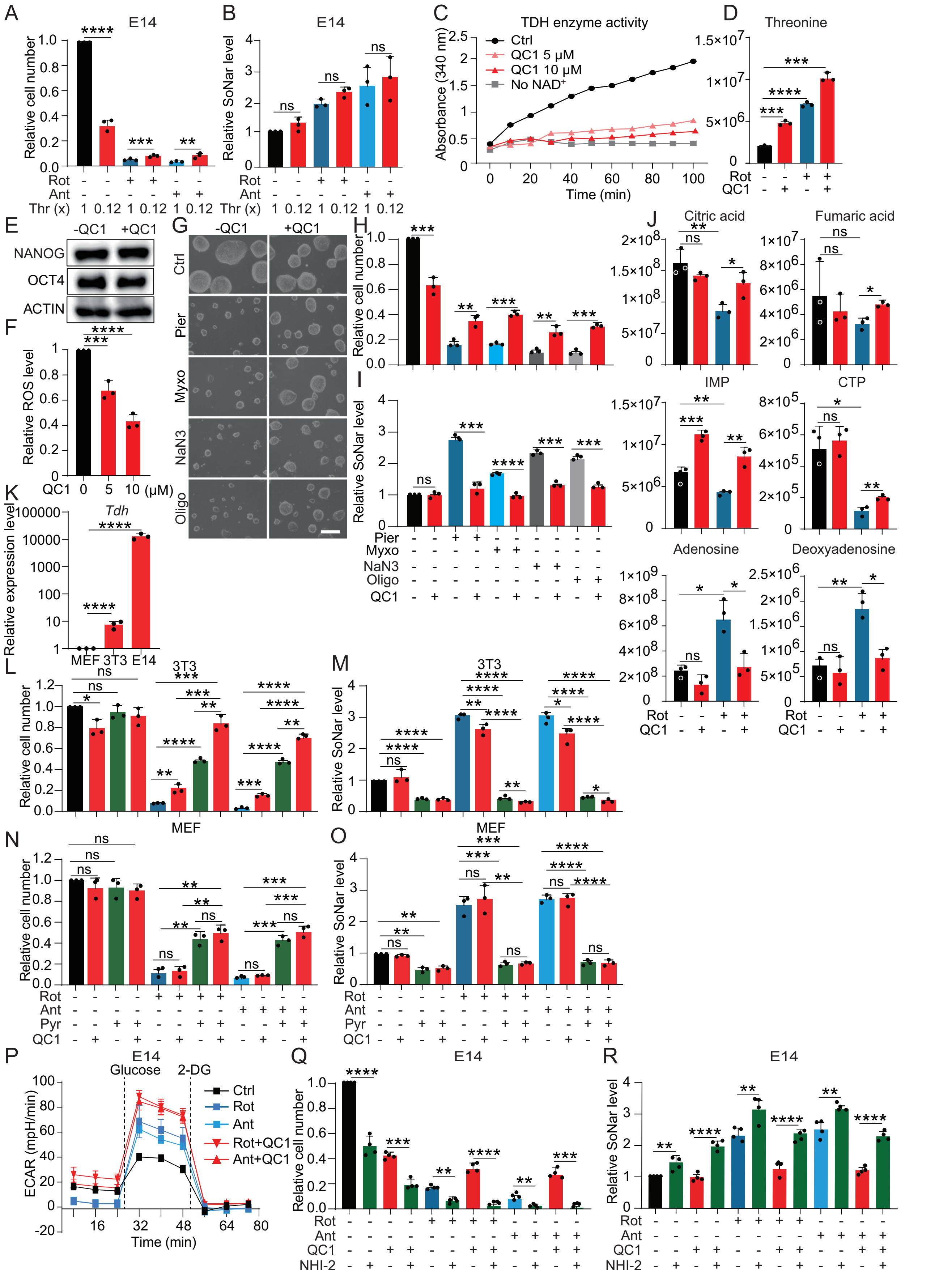
A
E14
Relative cell number
Rot
Ant
Thr (x)
B
E14
Relative SoNar level
C
TDH enzyme activity
Ctrl
QC1 5 μM
QC1 10 μM
No NAD+
Absorbance (340 nm)
Time (min)
D
Threonine
QC1
E
-QC1
+QC1
NANOG
OCT4
ACTIN
F
Relative ROS level
QC1 0 5 10 (μM)
G
Ctrl
Pier
Myxo
NaN3
Oligo
H
I
Pier
Myxo
NaN3
Oligo
J
Citric acid
Fumaric acid
IMP
CTP
Adenosine
Deoxyadenosine
K
Tdh
Relative expression level
MEF 3T3 E14
L
3T3
M
N
MEF
Pyr
O
P
E14
Glucose
2-DG
ECAR (mpH/min)
Ctrl
Rot
Ant
Rot+QC1
Ant+QC1
Time (min)
Q
NHI-2
R

Figure EV2. TDH sustains reductive stress and contributes to ETC C1 inhibition-induced pausing in mESCs, related to Fig. 1.

(**A**, **B**) Relative cell number (**A**) and relative SoNar levels (**B**) of E14 mESCs with Rot and Ant with indicated doses of threonine for 4 days in threonine-free 2iL media (mean ± SD, $n = 3$ each with biological replicates). (**C**) TDH enzyme activity assay for E14 mESCs treated with different doses of QC1. Enzyme activity was determined by absorbance at 340 nm over time to monitor the conversion of NAD^+ to NADH, and no NAD^+ acts as a negative control. (**D**) Intracellular threonine levels of metabolomics from E14 mESCs treated with Rot either alone or in combination with QC1 5 µM for 3 days (mean ± SD, $n = 3$ each with biological replicates). (**E**) Western blotting for OCT4 and NANOG in E14 mESCs treated with QC1 5 µM for 4 days. ACTIN was used as the loading control. (**F**) Relative reactive oxygen species (ROS) of E14 mESCs with the indicated doses of QC1 for 4 days (mean ± SD, $n = 3$ each with biological replicates). (**G–I**) Cell images (**G**), relative cell number (**H**), and relative SoNar levels (**I**) from E14 mESCs treated with Piericidin (Pier 2 µM) for C1, Myxothiazol (Myxo 5 nM) for C3, sodium azide (NaN3 500 µM) for C4, and oligomycin A (Oligo 10 nM) for C5 and examined rescue by QC1 for 4 days (mean ± SD, $n = 3$ each with biological replicates), scale bar, 200 µm. (**J**) Intracellular metabolites citrate, fumarate, IMP, CTP, adenosine, and deoxyadenosine of metabolomics from E14 mESCs treated with Rot either alone or in combination with QC1 for 3 days (mean ± SD, $n = 3$ each with biological replicates). (**K**) Relative expression level of *Tdh* in somatic cells (MEFs and 3T3) and E14 mESCs. (**L–O**) Relative cell number (**L**), relative SoNar levels of 3T3 (**M**), relative cell number (**N**), and relative SoNar levels of MEF (**O**), treated with the indicated treatments for 4 days (mean ± SD, $n = 3$ each with biological replicates). (**P**) ECAR analysis of E14 mESCs treated with the indicated treatments for 4 days (Mean ± SD $n = 1$ biological replicate with four technical replicates are shown). (**Q**, **R**) Relative cell number (**Q**) and relative SoNar levels (**R**) of E14 mESCs treated with the indicated treatments for 4 days (mean ± SD, $n = 3$ each with biological replicates). Data information, in Fig. EV2A, D, F, H–O, Q, R, columns represent mean, error bars are standard deviation, points are individual biological replicates; lines indicate comparisons between different groups; stars indicate statistical significance. $^*p < 0.05$, $^{**}p < 0.01$, $^{***}p < 0.001$, $^{****}p < 0.0001$; ns, not significant by unpaired two-tailed Student's t-test. Individual p values are provided in Table EV1.

 © The Author(s)

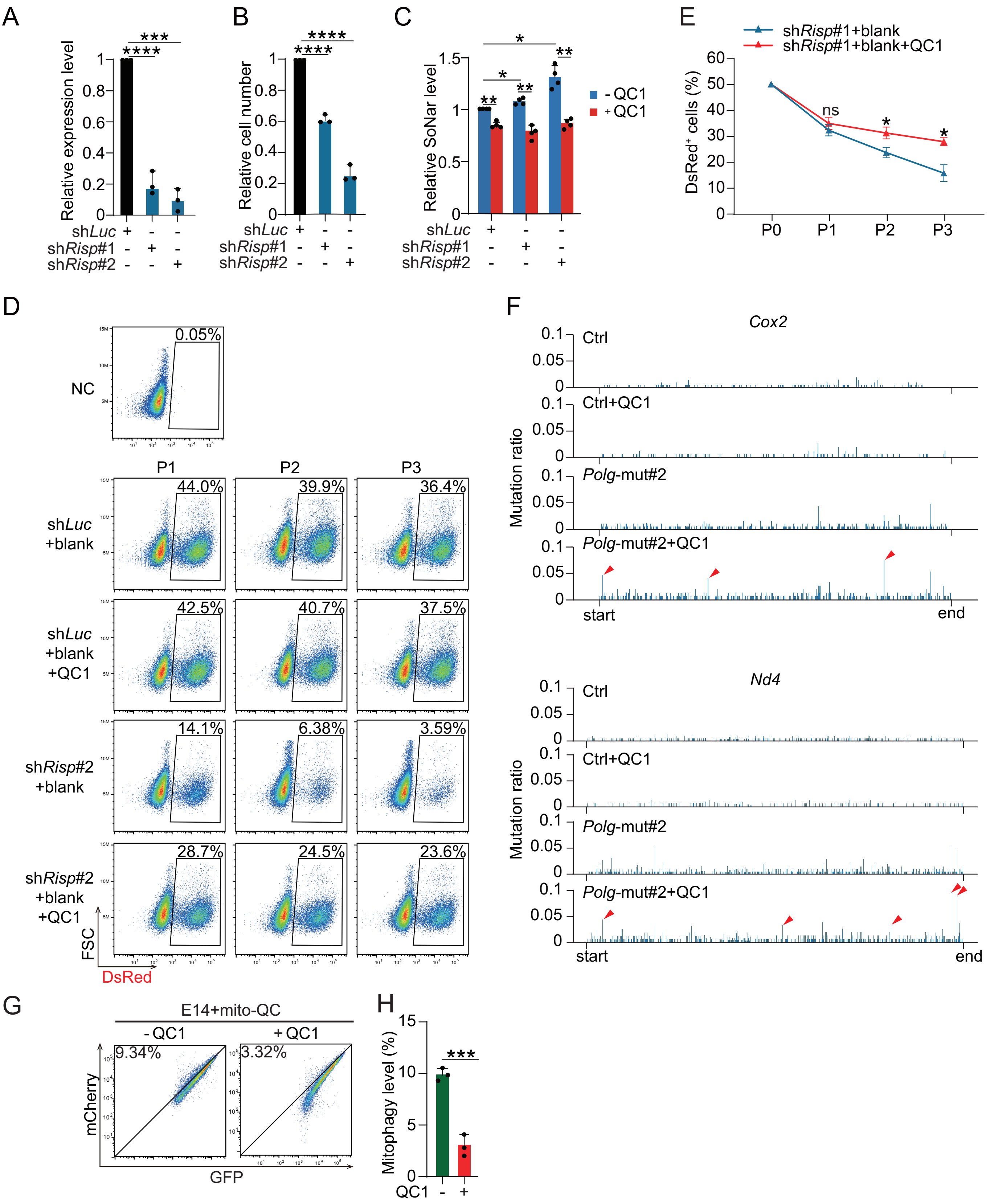
A
Relative expression level
shLuc
shRisp#1
shRisp#2
B
Relative cell number
C
Relative SoNar level
- QC1
+ QC1
E
shRisp#1+blank
shRisp#1+blank+QC1
DsRed+ cells (%)
P0
P1
P2
P3
ns
D
NC
0.05%
P1
P2
P3
shLuc +blank
44.0%
39.9%
36.4%
shLuc +blank +QC1
42.5%
40.7%
37.5%
shRisp#2 +blank
14.1%
6.38%
3.59%
shRisp#2 +blank +QC1
28.7%
24.5%
23.6%
FSC
DsRed
F
Cox2
Ctrl
Ctrl+QC1
Polg-mut#2
Polg-mut#2+QC1
Mutation ratio
start
end
Nd4
G
E14+mito-QC
- QC1
+ QC1
9.34%
3.32%
mCherry
GFP
H
Mitophagy level (%)
QC1

Figure EV3. TDH-driven reductive stress during ETC inhibition creates selection pressure against cells with mitochondrial impairment in mESCs, related to Fig. 2.

(**A**) RT-qPCR analysis showing the knockdown efficiency of sh*Risp* relative to sh*Luc* in E14 mESCs (mean ± SD, $n = 3$ each with biological replicates). (**B**) Relative cell number of E14 mESCs transduced with sh*Luc*, or sh*Risp* from passage 1 (mean ± SD, $n = 3$ each with biological replicates). (**C**) Relative SoNar levels of E14 mESCs transduced with sh*Luc*, or sh*Risp* with or without QC1 (5 µM) from passage 1 (mean ± SD, $n = 4$ each with biological replicates). (**D**) Represented flow cytometry analysis of DsRed$^+$ E14 mESCs in the indicated conditions. (**E**) Curves showing the percentage of DsRed$^+$ E14 mESCs transduced with sh*Luc* or sh*Risp* in co-culture with sh*Luc* blank E14 mESCs from passages 1 to 3 with or without QC1 (5 µM) ($n = 4$ each with biological replicates, points represent mean, error bars are standard deviation; stars indicate statistical significance of sh*Risp* with and without QC1 from P1 to 3. (**$p < 0.01$, ***$p < 0.001$, ****$p < 0.0001$, by unpaired two-tailed Student's *t*-test. Individual *p* values are provided in Table EV1). (**F**) The mutation ratio of two representative mitochondrial genes from the indicated conditions at passage 10. (**G**, **H**) Cell cytometry plots of mito-QC reporter (**G**), and its quantification (**H**) from E14 mESCs treated with or without QC1 (5 µM) from passage 10 (mean ± SD, $n = 3$ each with biological replicates). Columns represent mean, error bars are standard deviation, points are individual biological replicates; lines indicate comparisons between different groups; stars indicate statistical significance. Data information, in Fig. EV3A–C, H, columns represent mean, error bars are standard deviation, points are individual biological replicates; lines indicate comparisons between different groups; stars indicate statistical significance. *$p < 0.05$, **$p < 0.01$, ***$p < 0.001$, ****$p < 0.0001$; by unpaired two-tailed Student's *t*-test. Individual *p* values are provided in Table EV1.

 © The Author(s)

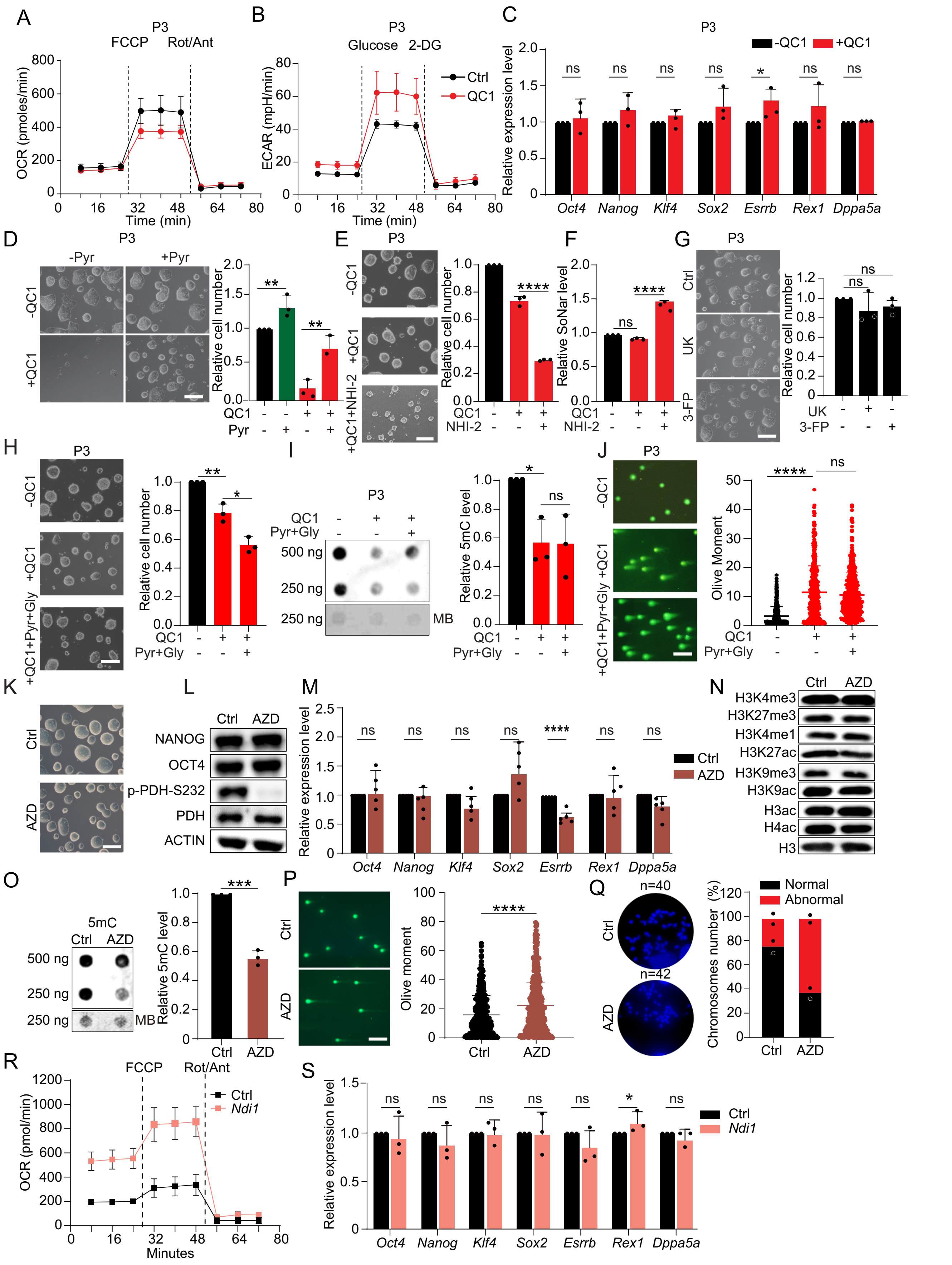
A
P3
FCCP Rot/Ant
OCR (pmoles/min)
Time (min)
B
P3
Glucose 2-DG
ECAR (mpH/min)
Ctrl
QC1
Time (min)
C
P3
-QC1 +QC1
Relative expression level
Oct4 Nanog Klf4 Sox2 Esrrb Rex1 Dppa5a
D
P3
-Pyr +Pyr
-QC1
+QC1
Relative cell number
QC1
Pyr
E
P3
-QC1
+QC1
+QC1+NHI-2
Relative cell number
QC1
NHI-2
F
Relative SoNar level
QC1
NHI-2
G
P3
Ctrl
UK
3-FP
Relative cell number
UK
3-FP
H
P3
-QC1
+QC1
+QC1+Pyr+Gly
Relative cell number
QC1
Pyr+Gly
I
P3
QC1
Pyr+Gly
500 ng
250 ng
250 ng
MB
Relative 5mC level
QC1
Pyr+Gly
J
P3
-QC1
+QC1
+QC1+Pyr+Gly
Olive Moment
QC1
Pyr+Gly
K
Ctrl
AZD
L
Ctrl AZD
NANOG
OCT4
p-PDH-S232
PDH
ACTIN
M
Relative expression level
Ctrl
AZD
Oct4 Nanog Klf4 Sox2 Esrrb Rex1 Dppa5a
N
Ctrl AZD
H3K4me3
H3K27me3
H3K4me1
H3K27ac
H3K9me3
H3K9ac
H3ac
H4ac
H3
O
5mC
Ctrl AZD
500 ng
250 ng
250 ng
MB
Relative 5mC level
Ctrl AZD
P
Ctrl
AZD
Olive moment
Ctrl AZD
Q
n=40
Ctrl
n=42
AZD
Chromosomes number (%)
Normal
Abnormal
Ctrl AZD
R
FCCP Rot/Ant
OCR (pmol/min)
Ctrl
Ndi1
Minutes
S
Relative expression level
Ctrl
Ndi1
Oct4 Nanog Klf4 Sox2 Esrrb Rex1 Dppa5a

Figure EV4. TDH-driven intrinsic reductive stress suppresses PDH activity and correlates with reduced genomic and epigenetic abnormalities in mESCs, related to Fig. 3.

(**A**) OCR analysis of E14 mESCs treated with and without QC1 (10 μM) in 2iL for three passages (Mean ± SD, $n = 1$, 5 technical replicates are shown). (**B**) ECAR analysis of E14 mESCs treated with and without QC1 (10 μM) in 2iL for three passages (Mean ± SD $n = 1$, 4 technical replicates are shown). (**C**) Relative expression levels of pluripotent markers in E14 mESCs treated with QC1 (10 μM) for three passages (mean ± SD, $n = 3$ each with biological replicates). (**D**) Cell images and relative cell number of E14 mESCs treated with or without QC1 in combination with or without pyruvate (2 mM) for 3 passages (mean ± SD, $n = 3$ each with biological replicates). (**E**, **F**) Cell images and relative cell number (**E**) and relative SoNar levels (**F**) of E14 mESCs treated with QC1, either alone or in combination with NHI-2 (10 μM), for three passages (mean ± SD, $n = 3$ each with biological replicates). (**G**) Cell images and relative cell number of E14 mESCs treated with and without UK5099 (15 μM) or 3-FP (700 nM) for three passages (mean ± SD, $n = 3$ each with biological replicates). (**H**) Cell images and relative cell number of E14 mESCs treated with and without QC1 in the absence or presence of a combination of pyruvate (10 mM) and glycine (6 mM), for ten passages (mean ± SD, $n = 3$ each with biological replicates). (**I**) Images and relative quantification of DNA dot blot of E14 mESCs treated with and without QC1, in the absence or presence of a combination of pyruvate and glycine for ten passages. Methylene blue was used as the loading control (mean ± SD, $n = 3$ each with biological replicates). (**J**) Representative images and quantification (olive moment) of neutral comet assay of E14 mESCs treated with and without QC1, in the absence or presence of a combination of pyruvate and glycine for ten passages (mean ± SD, $n = 3$ each with biological replicates), scale bar, 200 μm. (**K**) Cell images of E14 mESCs treated with AZD7545 (3 μM) for three passages, scale bar, 200 μm. (**L**) Western blotting for OCT4, NANOG, PDH, and p-PDH-S232 in E14 mESCs treated with AZD7545 for three passages. ACTIN was used as the loading control. (**M**) Relative expression levels of pluripotent markers in E14 mESCs treated with AZD7545 for three passages (mean ± SD, $n = 5$ each with biological replicates). (**N**) Western blotting for histone marks with indicated antibodies in E14 mESCs treated with AZD7545 for three passages. H3 was used as the loading control. (**O**) Images and relative quantification of the DNA dot blot of E14 mESCs treated with AZD7545 for three passages. Methylene blue was used as the loading control (mean ± SD, $n = 3$ each with biological replicates). (**P**) Representative images and quantification (olive moment) of neutral comet assay of E14 mESCs treated with AZD7545 for three passages (mean ± SD, $n = 3$ each with biological replicates), scale bar, 200 μm. (**Q**) Representative images and quantification (percentage of 41–43 chromosomes) of chromosomes of E14 mESCs treated with AZD7545 for ten passages ($n = 2$ each with biological replicates), scale bar, 40 μm. (**R**) OCR analysis of E14 mESCs transduced with Flag or *Ndi1* for three passages (Mean ± SD, $n = 1$, 5 technical replicates are shown). (**S**) Relative expression levels of pluripotent markers of E14 mESCs transduced with Flag or *Ndi1* for 3 passages (mean ± SD, $n = 3$ each with biological replicates). Data information, in Fig. EV4C–I, M, O, S; columns represent mean, points are individual biological replicates; and in (**J** and **P**), violin plots represent mean, points are individual cell DNA; error bars are standard deviation, lines indicate comparisons between different groups; stars indicate statistical significance. *$p < 0.05$, **$p < 0.01$, ***$p < 0.001$, ****$p < 0.0001$; ns, not significant by unpaired two-tailed Student's *t*-test. Individual *p* values are provided in Table EV1.

 © The Author(s)

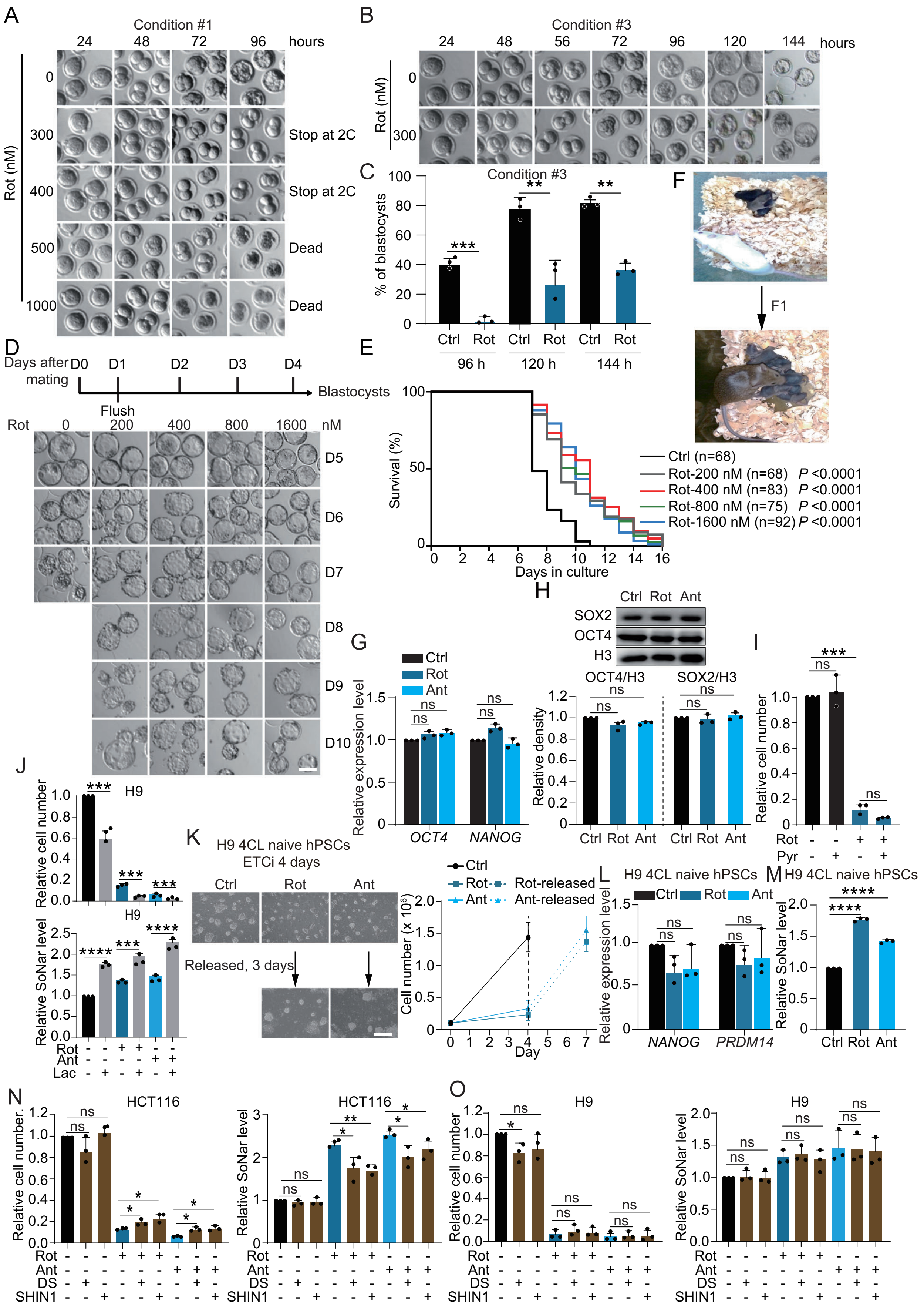
A
Condition #1
24 48 72 96 hours
Rot (nM)
0
300 Stop at 2C
400 Stop at 2C
500 Dead
1000 Dead
B
Condition #3
24 48 56 72 96 120 144 hours
Rot (nM)
0
300
C
Condition #3
% of blastocysts
Ctrl Rot Ctrl Rot Ctrl Rot
96 h 120 h 144 h
F
F1
D
Days after mating D0 D1 D2 D3 D4
Flush
Blastocysts
Rot 0 200 400 800 1600 nM
D5
D6
D7
D8
D9
D10
E
Survival (%)
Days in culture
Ctrl (n=68)
Rot-200 nM (n=68) P <0.0001
Rot-400 nM (n=83) P <0.0001
Rot-800 nM (n=75) P <0.0001
Rot-1600 nM (n=92) P <0.0001
G
Ctrl
Rot
Ant
Relative expression level
OCT4 NANOG
H
Ctrl Rot Ant
SOX2
OCT4
H3
OCT4/H3 SOX2/H3
Relative density
Ctrl Rot Ant Ctrl Rot Ant
I
Relative cell number
Rot
Pyr
J
H9
Relative cell number
Relative SoNar level
Rot
Ant
Lac
K
H9 4CL naive hPSCs
ETCi 4 days
Ctrl Rot Ant
Released, 3 days
Ctrl
Rot Rot-released
Ant Ant-released
Cell number (x 10⁶)
Day
L
H9 4CL naive hPSCs
Ctrl Rot Ant
Relative expression level
NANOG PRDM14
M
H9 4CL naive hPSCs
Relative SoNar level
Ctrl Rot Ant
N
HCT116
Relative cell number.
Relative SoNar level
Rot
Ant
DS
SHIN1
O
H9
Relative cell number
Relative SoNar level
Rot
Ant
DS
SHIN1

Figure EV5. ETC inhibition induces pausing in early embryonic development and human PSCs, related to Fig. 4.

(**A**) Images of developing embryos from control and condition 1 treated with different doses of Rot starting at 24 h post-mating, scale bar, 200 µm. (**B**, **C**) Images (**B**) and percentage (**C**) of developing embryos from control and condition #3 at the indicated time points (mean ± SD, $n = 3$ each with biological replicates), scale bar, 200 µm. (**D**) Collection and culture of fertilized eggs for blastocyst generation ex vivo. Blastocysts were treated with indicated doses of Rot and cultured until the blastocysts collapsed, scale bar, 200 µm. (**E**) Kaplan–Meier survival curves of blastocysts treated with rotenone (Rot). *n* represents the total number of blastocysts per condition, pooled from three independent experiments showing consistent survival trends. Statistical significance was determined by the log-rank (Mantel–Cox) test, comparing each Rot dose to control ($p < 0.0001$). (**F**) Images of live pups (F0, upper image) born from blastocysts treated with Rot 400 nM for 3 days and F1 live mice generated from F0 (lower image). (**G**) Relative expression levels of *OCT4* and *NANOG* in H9 primed hESCs treated with Rot or Ant for 4 days (mean ± SD, $n = 3$ each with biological replicates). (**H**) Western blotting for OCT4 and SOX2 and relative density of H9 primed hESCs treated with Rot or Ant for 4 days. H3 was used as the loading control (mean ± SD, $n = 3$ each with biological replicates). (**I**) Relative cell number of H9 primed hESCs treated with or without Rot and pyruvate (2 mM) in mTeSR (mean ± SD, $n = 3$ each with biological replicates). (**J**) Relative cell number and relative SoNar levels from H9 primed hESCs treated with Rot or Ant and with and without lactate (10 mM) for 4 days (mean ± SD, $n = 3$ each with biological replicates). (**K**) Cell images and cell number of H9 4CL naive hESCs treated with Rot (150 nM) or Ant (250 nM) for 4 days and released for 3 days in 4CL (mean ± SD, $n = 3$ each with biological replicates), scale bar, 200 µm. (**L**) Relative expression levels of pluripotent markers of H9 4CL naive hESCs treated with Rot or Ant for 4 days in 4CL (mean ± SD, $n = 3$ each with biological replicates). (**M**) Relative SoNar levels of H9 4CL naive hESCs treated with Rot or Ant for 4 days in 4CL medium (mean ± SD, $n = 3$ each with biological replicates), scale bar, 200 µm. (**N**, **O**) Relative cell number and relative SoNar levels (HCT116) (**N**), relative cell number and relative SoNar levels (H9) (**O**), with the indicated treatments for 4 days (mean ± SD, $n = 3$). Data information, in Fig. EV5C, G, H–J, L–O, columns represent mean, points are individual biological replicates; error bars are standard deviation, lines indicate comparisons between different groups; stars indicate statistical significance. *$p < 0.05$, **$p < 0.01$, ***$p < 0.001$, ****$p < 0.0001$; ns not significant by unpaired two-tailed Student's *t*-test. Individual *p* values are provided in Table EV1.

 © The Author(s)
