# Supplementary material for: Mitochondrial NADH-redox inflexibility constrains genomic and epigenetic stability in pluripotent stem cells
Source: EMBO J. 2026 May 27;45(13):4417–46. doi: 10.1038/s44318-026-00784-2 (PMC13324003; doi:10.1038/s44318-026-00784-2)
Supplement: Supplementary file 2 — Peer Review File [file 44318_2026_784_MOESM2_ESM.pdf]

THE EMBO JOURNAL

Mitochondrial NADH-redox inflexibility constrains genomic and epigenetic stability in pluripotent stem cells

Tanveer Ahmed, Hui Zhang, Ghulam Mushraf, Yongzhang Pan, Zeyu Zhang, Zhenzhu Sun, Mengran Yin, Xueting Xu, Xinyu Wu, Yin Gao, Yan Li, Peizhi Li, Liang Ding, Qiang Zhang, Runxia Lin, Khair Ullah, Yinghua Huang, Bushra Mirza, Yifeng Huang, Abdul Sammad, Xiangqian Kong, Dajiang Qin, Miguel Esteban, Lulu Wang, and Baoming Qin

Corresponding authors: Baoming Qin (qin_baoming@gibh.ac.cn) , Tanveer Ahmed (tanveer@gibh.ac.cn), Lulu Wang (wang_lulu@gzhmu.edu.cn)

Review Timeline:

Submission Date:	22nd Oct 24
Editorial Decision:	27th Jan 25
Appeal:	14th Jan 26
Editorial Decision:	2nd Feb 26
Editorial Correspondence:	4th Feb 26
Revision Received:	8th Mar 26
Accepted:	10th Apr 26

Editor: Daniel Klimmeck

Transaction Report:

(Note: With the exception of the correction of typographical or spelling errors that could be a source of ambiguity, letters and reports are not edited. Depending on transfer agreements, referee reports obtained elsewhere may or may not be included in this compilation. Referee reports are anonymous unless the Referee chooses to sign their reports.)

Dear Dr Qin,

Thank you for the submission of your manuscript (EMBOJ-2024-119405) to The EMBO Journal. Please accept my sincere apologies for the protraction in processing your manuscript, which as mentioned was due to delayed referee input as well as detailed discussion here in the editorial team. Your manuscript has been sent to two referees for evaluation with expertise in embryonic stem cell biology, development and metabolism, and we have received reports both of them, which I enclose below. I regret to say that in light of their input we decided that we cannot offer publication in The EMBO Journal.

As you will see, the referees appreciate the potential interest of your results. However, they also raise major concerns with the analysis that I am afraid preclude publication here. In more detail, referee #1 states substantial concerns regarding the experimental design chosen and the dosing applied and physiological relevance of your experiments (ref#1, pt.3). This referee also points to unclear specificity of experimental setup applied and important gaps remaining to convincingly support the claims made (ref#1, pts.1,2,4; see also ref#2, pt.4). Reviewer #2 expresses major reservations regarding unclear mechanistic detail provided on how TDH inhibition translates into stress response (Ref#2, pt.1); this expert also requests more evidence for conservation of the findings in human (Ref#2, pt.2).

Given these critical opinions from good experts on the field on multiple levels and i.p. considering that we operate with a single major round of revisions at our journal policy, I am afraid we concluded that we cannot offer to formally invite revisions.

Please note that given the interest stated. we would be able to editorially assess a substantially amended version of the study. Please note however that that given the extsive revision work requested the outcome of such reassessment is entirely unclear now.

Thank you in any case for the opportunity to consider this manuscript. I regret we cannot be more positive on this occasion but hope nevertheless that you will find our referees' comments helpful.

Kind regards,

Daniel Klimmeck

Daniel Klimmeck, PhD
Senior Editor
The EMBO Journal

****

Referee #1:

In many proliferating cells, electron transport chain (ETC) disruption decreases proliferative capacity through reductive stress, which is ameliorated by exogenous electron acceptor supplementation. In this manuscript submitted to EMBO, Ahmed et al. questioned why the self-renewal of pluripotent stem cells is blocked during ETC dysfunction even in the presence of electron acceptors like pyruvate and AKB, which ought to ease reductive stress on the cell. Interestingly, they find that in this context, pyruvate is not sufficient to robustly fix reductive stress because the mitochondrial TDH pathway (absent in humans) continues to generate NADH. They find that inhibiting TDH in complex I impaired mESCs rescued redox imbalance and self-renewal capacity. They show that the TDH pathway is therefore beneficial as it can act, via selection of the fittest, act as a quality control mechanism to filter out stem cells with impaired mitochondrial function. Investigation of this mechanism reveals that PDH is activated during TDH impairment, which leads to genetic abnormalities, indicating that TDH can potentially help preserve stem cell function. This manuscript identifies an interesting and unique metabolic phenomenon specific to mouse pluripotent stem cells: TDH can sustain reductive stress caused by ETC impairment, which could be a bottleneck in early mouse development. Overall the manuscript highlights some novel interactions and explores some interesting themes that builds surrounding NADH reductive stress. The observation that human PSCs are also paused during mitochondrial dysfunction, despite the absence of the TDH pathway in human cells, leaves room for further study. However, some missing important controls and metabolic links remain.

Major Issues:

1) It is not strictly true that pyruvate or AKB does not provide any rescue to E14 cells treated with rotenone or antimycin (Figure 1SD). While this figure is missing error bars and so is difficult to evaluate, there appears to be a ~3-4x increase in cell number at most doses of pyruvate or AKB compared to those without. This appears to be a similar effect size to the additional benefit received by treatment with QC1 (Figure 1Q). If the effect of these treatments is additive, that is not disqualifying for their model but needs to be discussed. It is also unclear how much of an effect QC1 has compared to pyruvate (alone) on redox status, since the mitochondrial inhibitor + Pyr (without QC1) condition in missing from Figure 1U. More clarity is needed on the effect size of these two treatment conditions individually and in combination on cell number and redox status.

2) The metabolic differences between E14 cells and other cell types remain unclear. The authors find that mouse E14 mESCs are paused by ETC dysfunction and not rescued by electron acceptors, aspartate, or an MTHFD2 inhibitor while 3T3 embryonic fibroblasts are rescued by aspartate or an MTHFD2 inhibitor. The authors hypothesize that E14 cells differ from 3T3 cells due to them having an additional NADH burden from increased expression (and presumably, increased activity) of threonine dehydrogenase (TDH) (Figure 1I). The data to support this argument is that a TDH inhibitor (QC1) increases cell number and decreases NADH in E14 cells. However, several experiments remain missing to proving this larger point. 1) What is the effect of QC1 on 3T3 cells (with or without pyruvate)? It would also be useful to determine how on target QC1 is by observing its effects on human cells, which do not have TDH activity. 2) Can the authors provide any direct evidence that this dose of QC1 is, in fact, inhibiting TDH in this setting? (e.g. threonine consumption, changes in 13C threonine labeling into the TCA cycle or lipid pool, etc). If so, are the metabolic effects of QC1 similar in 3T3 cells? Does TDH inhibition restore aspartate levels and other TCA metabolites in CI impaired mESCs? 3) Can limiting threonine in the media or outcompeting with 3-HNV cause similar effects as Tdh inhibition in both systems?

3) One line of reasoning is that E14 cells have different redox interactions compared to 3T3 cells because 3T3 cells are rescued by the MTHFD2 inhibitor DS, while E14 are not rescued. However, the dosing between these conditions is substantially different, with the rescue dose in 3T3 cells being 1-2 uM and the E14 cells being 25-50 nM. Using the lack of a rescue as an interpretable finding in E14 cells in the context where they also decrease the dosage by ~20 fold is highly concerning without orthogonal data supporting the appropriateness of these dosing choices. The authors need to address this discrepancy by showing the effects of a dose titration across the full range of this drug on cell number and SoNaR level in both contexts.

4) While the authors highlight that NDI1 expression reverses the effects of rotenone treatment on NADH/NAD+ homeostasis, they ignore the fact that it also restores all the other functions of CI activity (aside from proton pumping at CI), including proton pumping at complexes III and IV and downstream ATP synthesis, oxygen consumption, ROS homeostasis, etc. Perhaps the authors can instead employ a LbNOX (mito or cyto) to more directly separate the effects of complex I inhibition on NADH versus the other effects on mitochondrial metabolism (The Titov et al. lbnox paper is in the reference section but I did not see it cited in the text)?

5) The evidence that TDH inhibition causes a meaningful decrease in acetyl-coa production and "TDH to PDH rewiring" is somewhat weak. My understanding is that this is concluded simply by the change in phosphorylation on PDH (which is well known to track with NADH/NAD+) upon QC1 treatment and an increased sensitivity to UK5099 and 3FP in QC1 treated cells. However, these authors also show that QC1 rescues the toxicity of rotenone, which is also well known to impair PDH activity by increasing mitochondrial NADH, implying that neither route of acetyl-coa production is needed for self-renewal and the UK5099/3FP may be toxic for another reason. If the toxicity of QC1 and UK5099 or 3-FP is due to acetyl-coa deficiency, can it be rescued by acetate? Does the combination of QC1 with UK5099, 3-FP, or rotenone impact histone acetylation? More evidence is needed to claim that a TDH-to-PDH rewiring is occurring and necessary for acetyl-coa production, particularly if they want to claim this impacts self-renewal/cell proliferation.

6) Similarly, a subsequent claim is that the histone hypomethylation is a result of THD inhibition impacting one carbon metabolism, by decreasing the availability of SAM. This hypothesis derives from cited work (Shyh-Chang et al., 2013) describing this effect, who showed that glycine/pyruvate treatment could ameliorate epigenetic dysregulation from impairments in threonine catabolism in mESCs. Can the authors determine if glycine/pyruvate is similarly able to restore epigenetic status and/or reverse DNA damage phenotypes?

Minor Issues:

Some schematics of ETC dysfunction in Figure 1 would be helpful.

The statement that "The propensity to sustain strong reductive stress during ETC inhibition functions as a mitochondrial quality control mechanism in mESCs" is misleading, as it seems to imply that it activates a response pathway to repair intracellular mitochondria. Even knowing that it is instead referring the proliferative disadvantage of these cells, it still implies a level of functional knowledge that has not been tested (i.e. if TDH were missing, would mice propagate dysfunctional mitochondria into progeny?). I would recommend carefully rewording this statement (and similar ones elsewhere in the text) to more accurately reflect that this is simple Darwinian selection against slow growing cells.

Referee #2:

General Summary and Opinion

This study investigates the role of NADH reductive stress in mediating developmental pausing induced by electron transport chain (ETC) inhibition in both mouse embryonic stem cells (mESCs) and human pluripotent stem cells (hPSCs). The authors demonstrate that ETC inhibition leads to a reversible paused pluripotent state, with reductive stress playing a central role and TDH identified as a key regulator of mitochondrial and nuclear stability. These findings extend to early mouse embryonic development, where ETC inhibition induces developmental pausing at the 2-cell (2C) stage and in blastocysts, offering insights into how cells with mitochondrial DNA (mtDNA) mutations may be selectively excluded.

The study is significant as it sheds light on the intersection of mitochondrial metabolism, redox balance, and cellular decision-making, particularly in early development and pluripotency. However, while the data presented is compelling, several major and minor issues need to be addressed to solidify the study's conclusions and enhance its translational relevance.

Major Concerns

1. Mechanistic Role of TDH in Reductive Stress
o The study convincingly demonstrates that TDH inhibition alleviates reductive stress, but its precise biochemical role in regulating NADH levels needs to be clarified.
o Experimental data examining the relationship between TDH, PDH, and other NADH-consuming enzymes is necessary to strengthen the central argument.
2. Relevance to Human Biology
o Since TDH is a pseudogene in humans, the relevance of these findings to human biology needs to be addressed. The authors speculate that a similar mechanism may exist in human PSCs but provide no direct evidence.
o Investigating whether LDH could serve as a functional analog of TDH in humans is essential to establish translational relevance.
o Additional experiments assessing LDH activity and its role in human PSCs under ETC inhibition need to be included.
o Instead of just adding pyruvate to the media, lactate should be added. Lactate is not found in commercially available media, but is abundant in the circulation. Pushing the LDH reaction in the opposite direction will produce more NADH. (see blow)
3. Role of the Mitochondrial NAD Transporter (SLC25A51)
o Given the centrality of NADH in the proposed mechanism, the role of the mitochondrial NAD transporter SLC25A51 needs to be assessed. The authors should evaluate its contribution by inhibiting or knocking it out to clarify its involvement.
4. Validation of Experimental Tools
o The use of inhibitors (e.g., QC1, Rot, Ant, UK5099) requires validation to ensure specificity. Non-specific effects of these inhibitors could confound the results.
o All inhibitor experiments need to be complemented with knockout (KO) data to confirm that the observed effects are due to the intended targets.
5. Metabolic Pathway Directionality
o Steady-state metabolomics does not confirm pathway directionality. The authors need to incorporate 13C-tracing experiments, such as using 13C-glucose, to definitively determine reaction fluxes.
6. Lactate as a Potential Modulator
o The authors need to explore the addition of lactate to the media to test its impact on NADH generation via the LDH reaction. This is critical given the central role of NADH in the study and lactate's abundance in vivo.
7. Mitochondrial Health and Function
o Additional measures of mitochondrial health, such as blue native gels to analyze ETC complexes and supercomplex formation, are necessary to support mitochondrial function conclusions.
o Assessing mitochondrial pyruvate carrier (MPC1 and MPC2) levels is necessary to better interpret the observed effects.

Minor Concerns

1. Use of Acronyms
o The authors use acronyms throughout the paper without first introducing them, making the manuscript difficult to follow and potentially confusing for readers unfamiliar with the field.
2. Normalization and Cell Viability
o The authors need to ensure that all metabolomics data, including NADH and NAD+, is normalized to cell number or protein content. If this was done, it should be clearly stated in the text.
o Cell viability assays need to accompany all experiments to exclude cell death as a confounding factor.
3. Consistency in Experimental Design
o PDK inhibitors, PDH activation, and MPC modulation are used inconsistently across experiments. A more uniform approach is needed to strengthen the study's conclusions.
4. Controls for High-Dose Treatments
o The controls used for high-dose treatments (e.g., QC1, Rot) need to be clarified. Were alternative ETC inhibitors tested to confirm specificity?
5. Replicates
o The number of biological replicates, particularly in animal experiments, needs to be increased to improve statistical confidence in the findings.

Non-Essential Suggestions

1. Epigenetic and Transcriptomic Analysis
o Including bisulfite sequencing to assess methylation changes and RNA-seq to confirm transcriptional alterations would provide a more comprehensive understanding of downstream effects.
2. Mitochondrial Quality Control Mechanisms
o The discussion briefly mentions mitochondrial DNA mutation accumulation. Expanding this to include pathways like mitochondrial autophagy would enhance the study's broader implications.
3. Generalizability
o The authors could discuss whether the mechanisms observed in PSCs are likely to apply to differentiated cells or other stem cell types.

Conclusion
This is a well-executed and insightful study that advances our understanding of NADH reductive stress in developmental pausing and pluripotency. Addressing the major concerns, particularly the role of TDH, its human analogs, and the mitochondrial NAD transporter, is necessary to support the study's conclusions and enhance its translational impact. Incorporating additional experimental validations, introducing acronyms clearly, and discussing the broader implications would further strengthen the manuscript.
The study has the potential to make a significant contribution to mitochondrial biology and its role in cellular decision-making and early development.

** As a service to authors, EMBO Press provides authors with the possibility to transfer a manuscript that one journal cannot offer to publish to another EMBO publication or the open access journal Life Science Alliance launched in partnership between EMBO Press, Rockefeller University Press and Cold Spring Harbor Laboratory Press. The full manuscript and if applicable, reviewers' reports, are automatically sent to the receiving journal to allow for fast handling and a prompt decision on your manuscript. For more details of this service, and to transfer your manuscript please click on Link Not Available. **

Please do not share this URL as it will give anyone who clicks it access to your account.

Referee#1:

In many proliferating cells, electron transport chain (ETC) disruption decreases proliferative capacity through reductive stress, which is ameliorated by exogenous electron acceptor supplementation. In this manuscript submitted to EMBO, Ahmed et al. questioned why the self-renewal of pluripotent stem cells is blocked during ETC dysfunction even in the presence of electron acceptors like pyruvate and AKB, which ought to ease reductive stress on the cell. Interestingly, they find that in this context, pyruvate is not sufficient to robustly fix reductive stress because the mitochondrial TDH pathway (absent in humans) continues to generate NADH. They find that inhibiting TDH in complex I impaired mESCs rescued redox imbalance and self-renewal capacity. They show that the TDH pathway is therefore beneficial as it can act, via selection of the fittest, act as a quality control mechanism to filter out stem cells with impaired mitochondrial function. Investigation of this mechanism reveals that PDH is activated during TDH impairment, which leads to genetic abnormalities, indicating that TDH can potentially help preserve stem cell function. This manuscript identifies an interesting and unique metabolic phenomenon specific to mouse pluripotent stem cells: TDH can sustain reductive stress caused by ETC impairment, which could be a bottleneck in early mouse development. Overall the manuscript highlights some novel interactions and explores some interesting themes that builds surrounding NADH reductive stress. The observation that human PSCs are also paused during mitochondrial dysfunction, despite the absence of the TDH pathway in human cells, leaves room for further study. However, some missing important controls and metabolic links remain.

Response: We thank the reviewer for the positive assessment of our manuscript.

Major Issues:

1) It is not strictly true that pyruvate or AKB does not provide any rescue to E14 cells treated with rotenone or antimycin (Figure 1SD). While this figure is missing error bars and so is difficult to evaluate, there appears to be a ~3-4x increase in cell number at most doses of pyruvate or AKB compared to those without. This appears to be a similar effect size to the additional benefit received by treatment with QC1 (Figure 1Q). If the effect of these treatments is additive, that is not disqualifying for their model but needs to be discussed. It is also unclear how much

of an effect QC1 has compared to pyruvate (alone) on redox status, since the mitochondrial inhibitor + Pyr (without QC1) condition in missing from Figure 1U. More clarity is needed on the effect size of these two treatment conditions individually and in combination on cell number and redox status.

Response:

We thank the reviewer for these thoughtful comments. We have now assessed this issue with direct experiments. As shown in the Rebuttal Figure 1A–C, pyruvate or AKB provides a moderate but significant rescue effect in E14 cells treated with rotenone (Rot) or antimycin A (Ant), and these are accompanied with a reduction of cytosolic redox, based on SoNar measurement. These new data support the reviewer's idea that in mESCs, electron acceptors still play a role in reducing reductive stress, although weaker than in other cell types, such as MEF or 3T3.

We have removed the original EV1D and replaced it with these new results (see the new figure EV1E and corresponding text in the revised manuscript, page 5, lines 110–115).

Rebuttal Figure 1

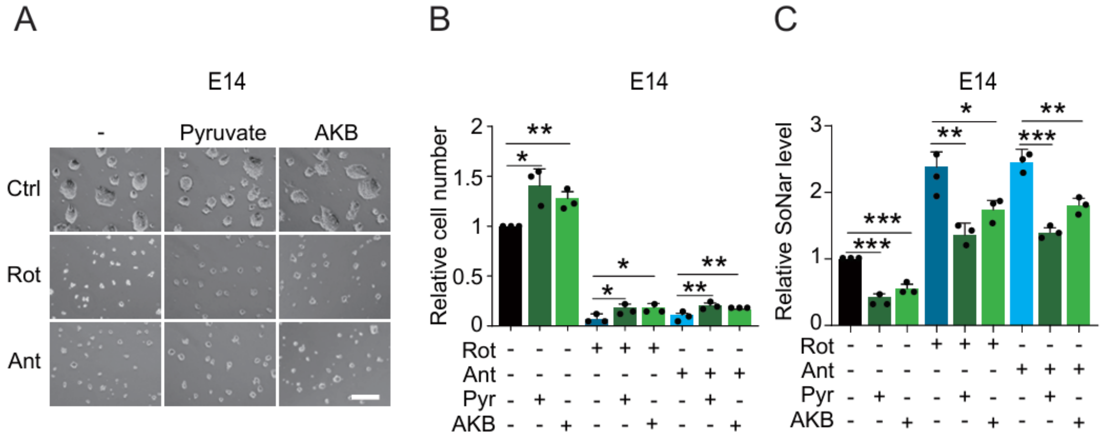

For the original Figure 1U, we had in fact included the pyruvate-alone condition in our initial experiments, as now shown in Rebuttal Figures 1D–F. We did not present this condition in the original figure because pyruvate showed no rescue effect on proliferation, although it markedly reduced SoNar signals. The discrepancy in pyruvate's effects between the two experiments (Rebuttal Fig. 1A–C vs. 1D–F) likely arises from the use of feeder cells. As noted in our original text, when both ETC and TDH are inhibited, pyruvate becomes essential for the survival of mESCs on gelatin. Feeder cells can partially compensate for pyruvate deficiency, but at the same time mask its moderate rescue effect on proliferation. In any case, this discrepancy further highlights that the ability of pyruvate to

alleviate the paused phenotype of mESCs is context-dependent.

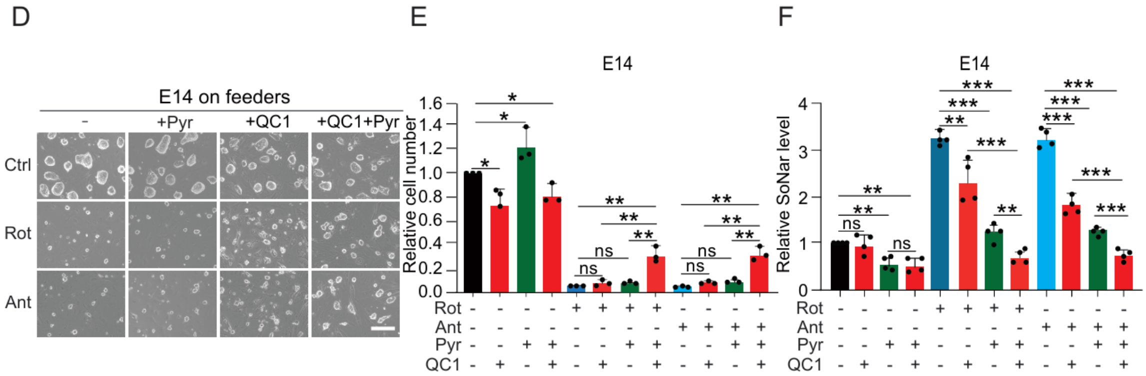

We have now included these conditions in the revised manuscript (new figure 1S–U, and corresponding text on page 7, lines 171–175).

2) The metabolic differences between E14 cells and other cell types remain unclear. The authors find that mouse E14 mESCs are paused by ETC dysfunction and not rescued by electron acceptors, aspartate, or an MTHFD2 inhibitor while 3T3 embryonic fibroblasts are rescued by aspartate or an MTHFD2 inhibitor. The authors hypothesize that E14 cells differ from 3T3 cells due to them having an additional NADH burden from increased expression (and presumably, increased activity) of threonine dehydrogenase (TDH) (Figure 1I). The data to support this argument is that a TDH inhibitor (QC1) increases cell number and decreases NADH in E14 cells. However, several experiments remain missing to proving this larger point. 1) What is the effect of QC1 on 3T3 cells (with or without pyruvate)? It would also be useful to determine how on target QC1 is by observing its effects on human cells, which do not have TDH activity. 2) Can the authors provide any direct evidence that this dose of QC1 is, in fact, inhibiting TDH in this setting? (e.g. threonine consumption, changes in 13C threonine labeling into the TCA cycle or lipid pool, etc). If so, are the metabolic effects of QC1 similar in 3T3 cells? Does TDH inhibition restore aspartate levels and other TCA metabolites in CI impaired mESCs? 3) Can limiting threonine in the media or outcompeting with 3-HNV cause similar effects as Tdh inhibition in both systems?

Response:

We thank the reviewer for these insightful questions. To test whether the ETCi-induced proliferation arrest depends on TDH expression and activity, we first compared *Tdh* mRNA levels across different cell types, including somatic cells (MEFs, which lack *Tdh* expression), immortalized fibroblasts (3T3), and mESCs

(E14). As expected, E14 cells expressed high levels of *Tdh*, while 3T3 cells expressed lower, yet detectable levels (Rebuttal Fig. 2A). Consistent with this, QC1 rescued ~2 fold of the ETC inhibitor-induced blockade of proliferation and moderate SoNar signals in 3T3 cells, whereas pyruvate produced a stronger rescue (~4–5 fold) and fully normalized the SoNar signal similar like pyruvate alone. The combined treatment (pyruvate + QC1) further enhanced proliferation rescue in 3T3 cells. In E14 cells, neither QC1 nor pyruvate alone restored proliferation, although both partially rescued the SoNar signal; however, their combination rescued proliferation by ~3–4 fold and fully restored the SoNar signal. In MEFs, only pyruvate rescued proliferation and SoNar, and QC1—alone or combined with pyruvate—provided no additional benefit (Rebuttal Fig 2B–D). Together, these results indicate that pyruvate plays the predominant role in rescuing ETC inhibitor-induced defects in 3T3 cells and MEFs, whereas in E14 cells its effectiveness requires TDH inhibition by QC1. These findings further support that QC1's rescue activity is dependent on *Tdh* expression levels and reflects TDH-specific inhibition.

Rebuttal Figure 2

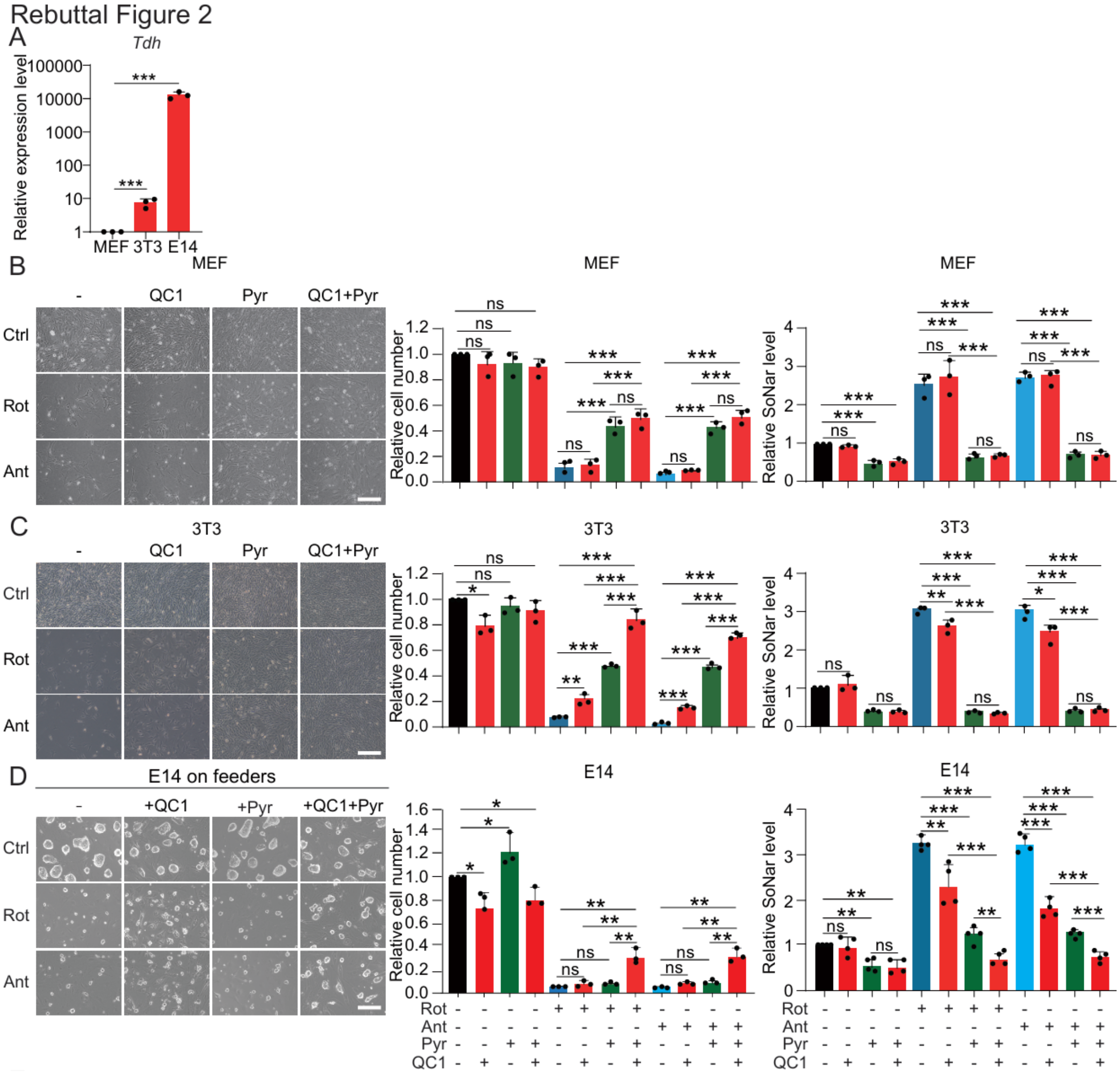

Furthermore, QC1 treatment had no detectable effect in human ESCs (H9) or in HCT116 cells, both of which lack *Tdh* expression (Rebuttal Fig. 2E, F).

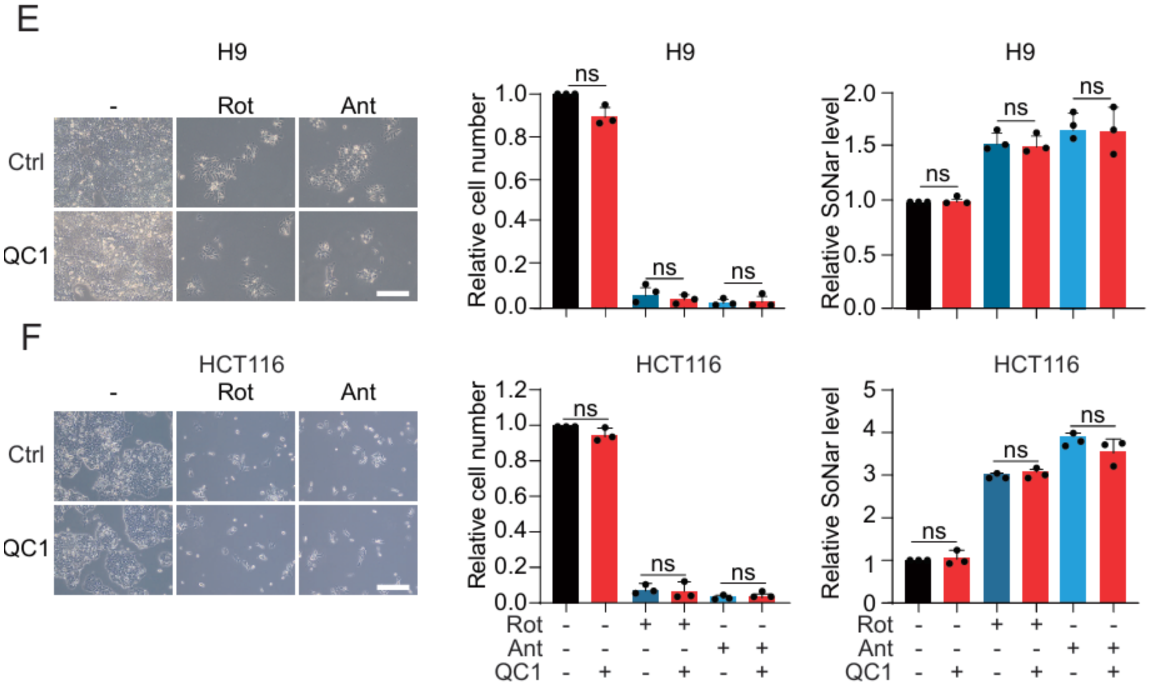

These negative results, which further support the TDH-specific action of QC1, are provided in the rebuttal for your review. We have kept them out of the revised manuscript to maintain focus and clarity, but we are happy to include them in the main text or supplementary materials if the reviewer think it is necessary.

(2) To further confirm that QC1 reliably inhibits TDH activity, we conducted metabolomic analyses in E14 cells treated with rotenone (Rot) with or without QC1. QC1 treatment caused threonine accumulation under both basal and Rot conditions, consistent with TDH inhibition. Moreover, QC1 restored the levels of numerous metabolites altered by Rot treatment, including TCA intermediates (citrate, fumarate), nucleotide and nucleotide precursors (IMP, CTP, adenosine, deoxyadenosine), and lipids such as LPC (30:0) and sphinganine (d18:0) (Rebuttal Fig. 2G–J). Although aspartate was not detected in this dataset, these results collectively support QC1's role as a functional TDH inhibitor that restores at least partially metabolic homeostasis.

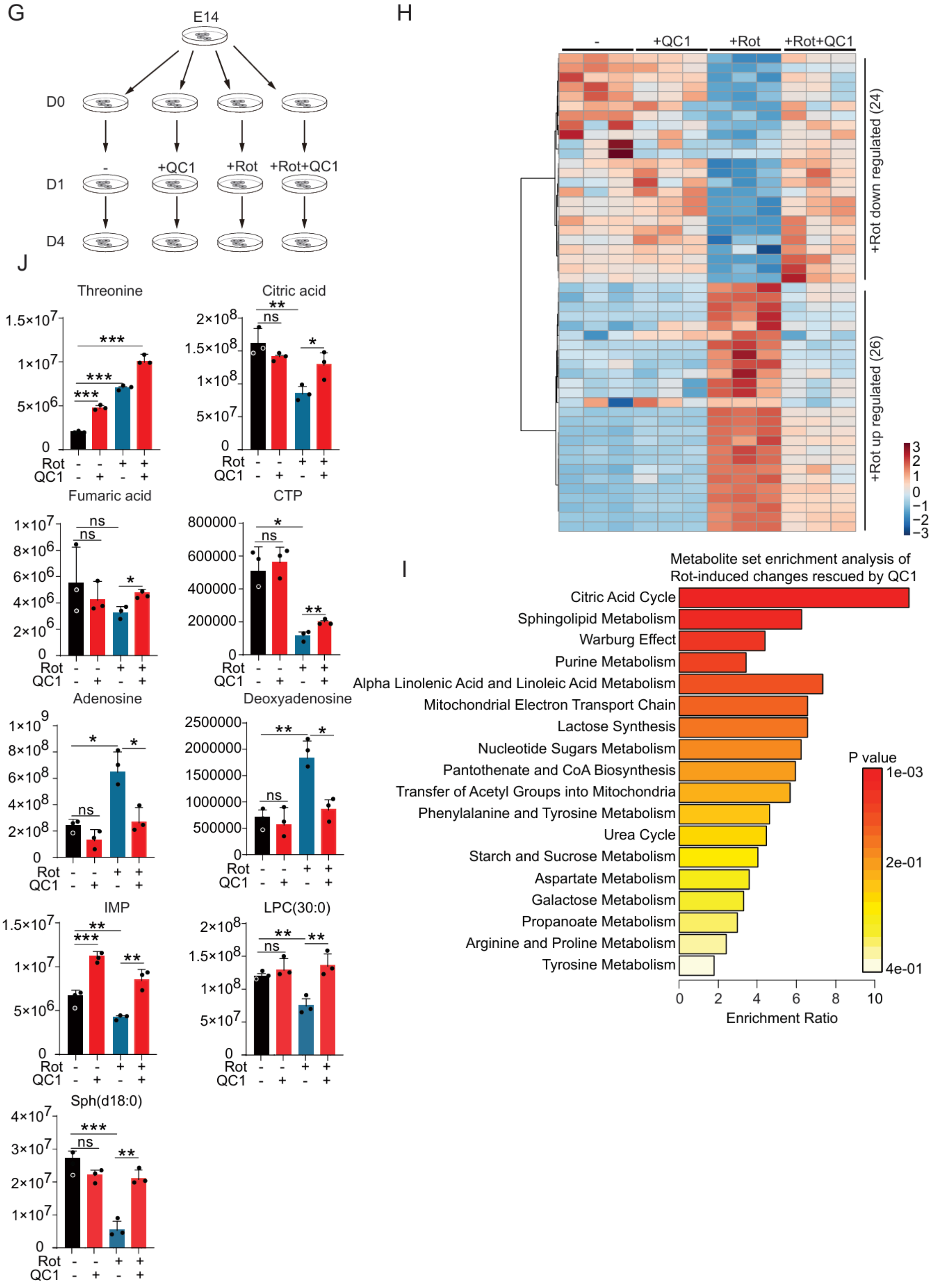

(3) To further test the role of threonine metabolism, we reduced threonine in the medium, which moderately rescued the paused phenotype of ETCi-treated E14 cells (Rebuttal Fig. 2K). This modest effect likely reflects incomplete suppression of threonine flux and limited impact on cytosolic redox. In contrast, the threonine analogue 3-HNV failed to rescue proliferation in either E14 or 3T3 cells (Rebuttal Fig. 2L, M). These negative results of 3-HNV further support QC1's specificity for

TDH, though they are not included in the revised manuscript to keep manuscript focused. However, we are ready to include in revised manuscript if reviewer think it is necessary.

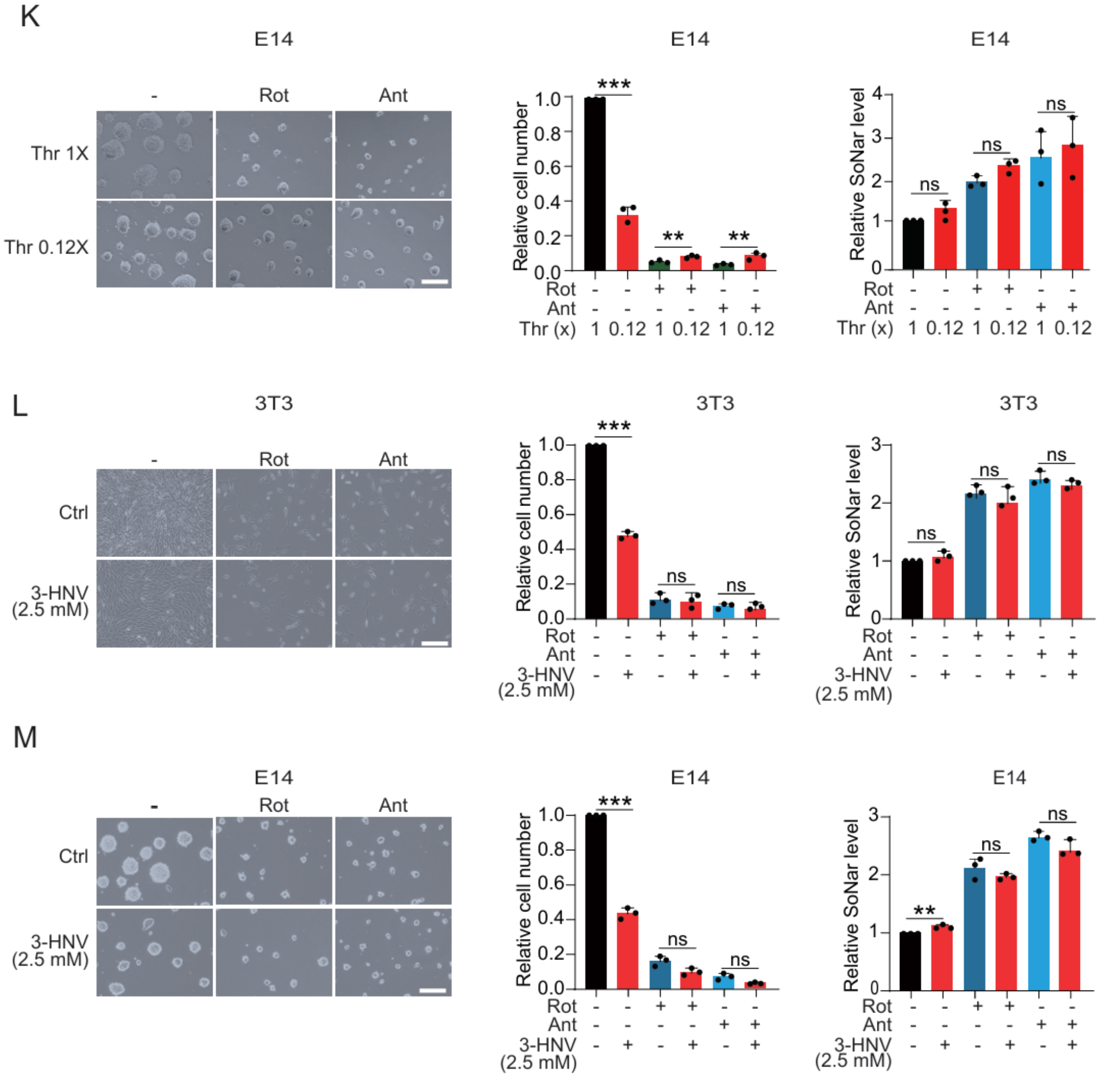

Together, these new data provide multiple lines of evidence that QC1 specifically targets TDH and that threonine catabolism through TDH contributes to the NADH burden and proliferative arrest observed during ETC inhibition in *Tdh*-expressing cells.

We have now included these new results in the revised manuscript (new figures 1S–U, EV2K–O, EV2J, EV2A, B and EV2D, and corresponding text on page 6,7, lines 171–177, 169–171, 155–159 and 161–163).

3) One line of reasoning is that E14 cells have different redox interactions compared to 3T3 cells because 3T3 cells are rescued by the MTHFD2 inhibitor DS, while E14 are not rescued. However, the dosing between these conditions is substantially different, with the rescue dose in 3T3 cells being 1-2 uM and the E14

cells being 25-50 nM. Using the lack of a rescue as an interpretable finding in E14 cells in the context where they also decrease the dosage by ~20 fold is highly concerning without orthogonal data supporting the appropriateness of these dosing choices. The authors need to address this discrepancy by showing the effects of a dose titration across the full range of this drug on cell number and SoNaR level in both contexts.

Response:

We thank the reviewer for highlighting this important point. To directly address the concern, we performed a full dose titration of DS in both E14 and 3T3 cells over a range of 12.5–200 nM. We found that in 3T3 cells, 50–200 nM DS significantly rescued ETCi-induced defects in both proliferation and SoNar signals (Rebuttal Fig. 3A–C). By contrast, E14 cells did not show any rescue effect at any dose tested (Rebuttal Fig. 3D–F). These new data support our original conclusion and further emphasize the distinct features of one-carbon metabolism between the two cell types.

We have incorporated these new results (covering the 50–100 nM range) into the revised manuscript (new figure EV1M–P and corresponding text on page 6, lines 141–148).

Rebuttal Figure 3

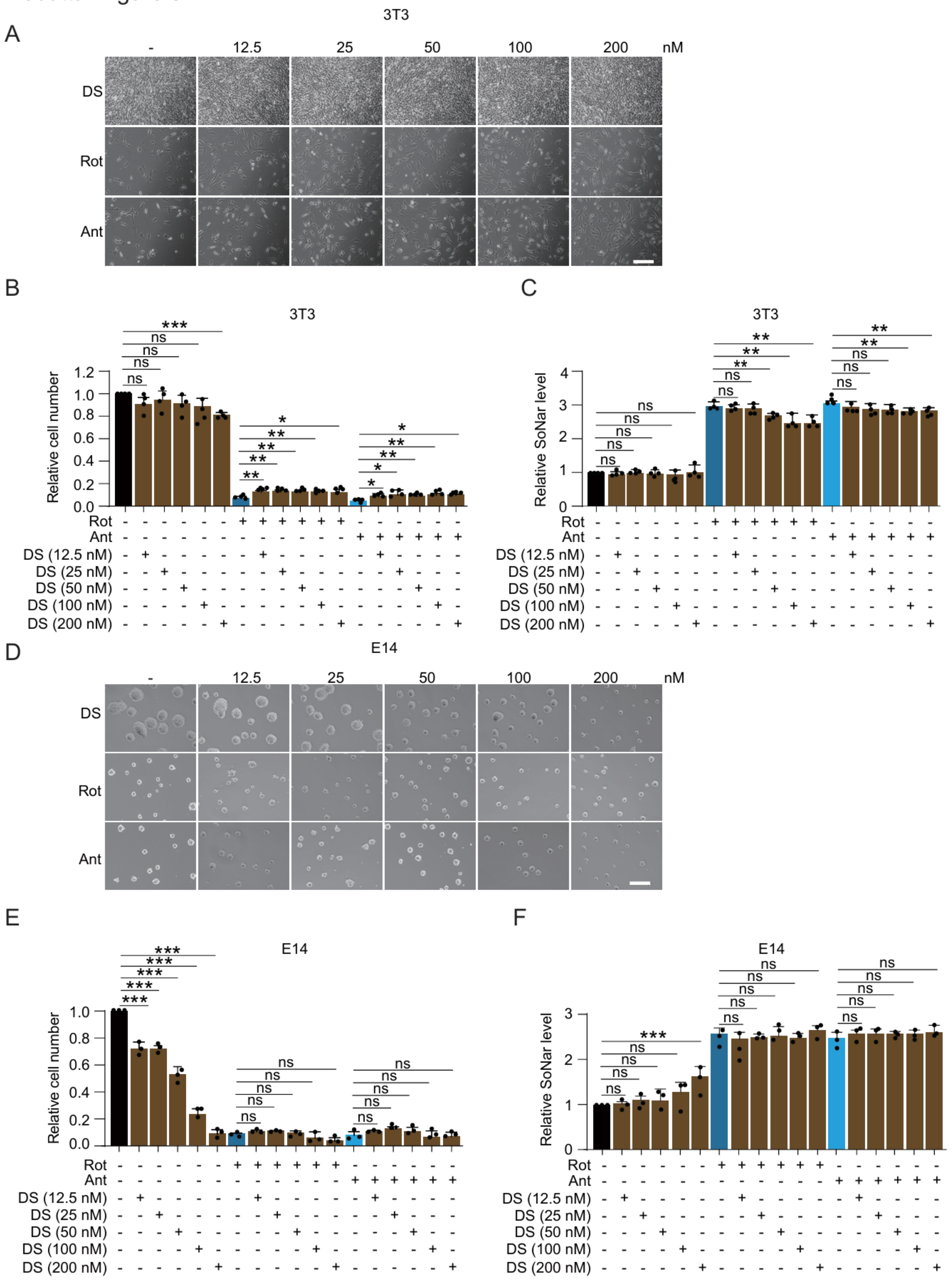

4) While the authors highlight that NDI1 expression reverses the effects of rotenone treatment on NADH/NAD+ homeostasis, they ignore the fact that it also restores all the other functions of CI activity (aside from proton pumping at CI), including proton pumping at complexes III and IV and downstream ATP synthesis, oxygen consumption, ROS homeostasis, etc. Perhaps the authors can instead employ a LbNOX (mito or cyto) to more directly separate the effects of complex I

inhibition on NADH versus the other effects on mitochondrial metabolism (The Titov et al. lbnox paper is in the reference section but I did not see it cited in the text)?

Response:

We thank the reviewer for this insightful comment. We agree that *Ndi1* not only restores rotenone-induced NADH/NAD$^+$ imbalance but also compensates for other complex I activity–dependent functions (except CI proton pumping). To directly separate the effects on NADH/NAD$^+$ from other mitochondrial functions, we expressed both mitochondrial and cytosolic forms of *Lb*NOX (mito-*Lb*NOX and cyto-*Lb*NOX) in 3T3 and E14 cells, and confirmed their expression (Rebuttal Fig. 4A, B). In 3T3 cells, both mito- and cyto-*Lb*NOX partially rescued ETC inhibitor–induced proliferation defects (~2–4-fold) and fully restored SoNar signals (Rebuttal Fig. 4C–E). In contrast, neither form rescued proliferation nor SoNar signals in E14 cells (Rebuttal Fig. 4F–H), suggesting that the cytosolic/mitochondrial NADH/NAD$^+$ imbalance remains unresolved and is therefore a principal driver of the paused phenotype. We acknowledge that *Lb*NOX activity in our hands may be suboptimal; nevertheless, the lack of SoNar rescue in E14, together with the partial rescue observed in 3T3 and the full rescue by *Ndi1*, supports the conclusion that NADH/NAD$^+$ disequilibrium is a major causal factor, while complete restoration of electron flow (as achieved by *Ndi1*) is required for full phenotypic recovery.

We have incorporated these new data into the revised manuscript (new figure EV1H–L and corresponding text on page 5, 6, line 130–136) and corrected the citation of Titov et al (page 6,9, line 132,133 and 259).

Rebuttal Figure 4

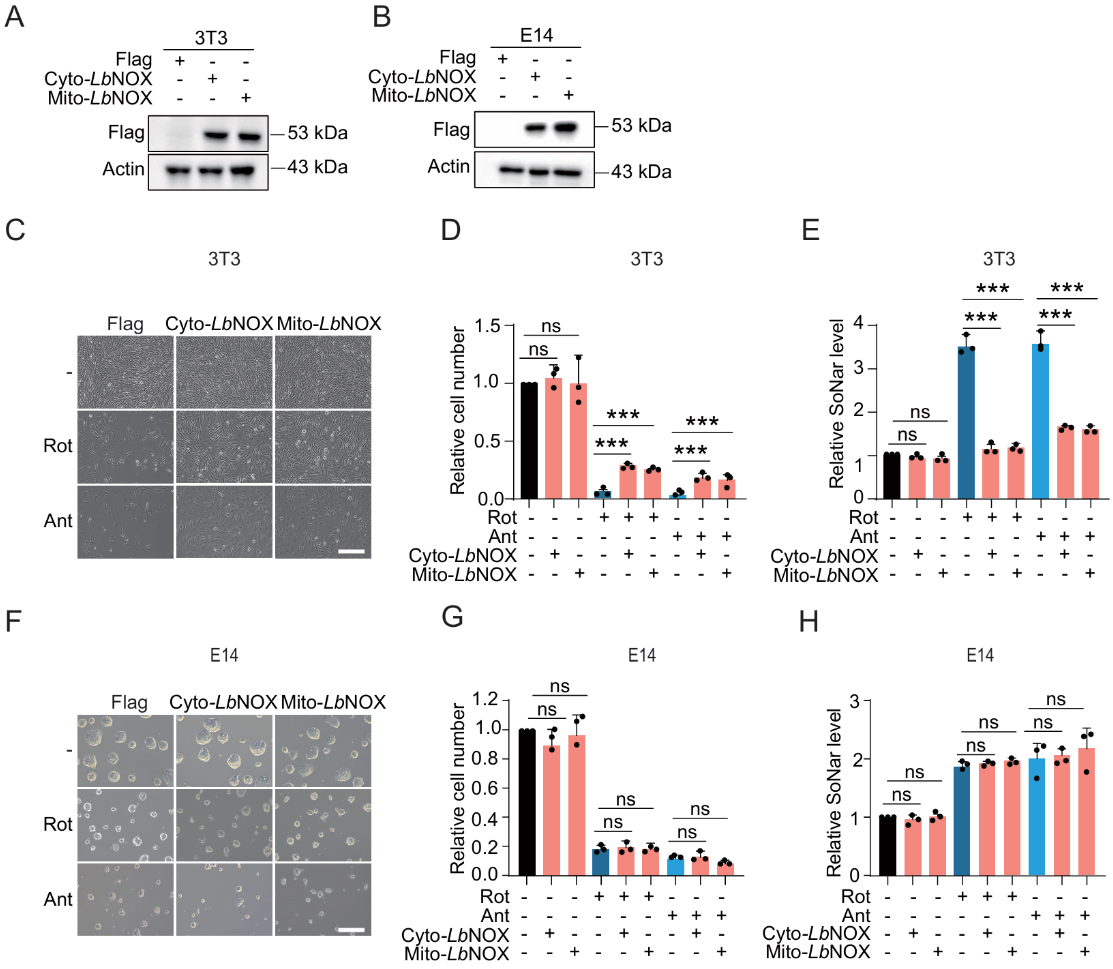

5) The evidence that TDH inhibition causes a meaningful decrease in acetyl-coa production and "TDH to PDH rewiring" is somewhat weak. My understanding is that this is concluded simply by the change in phosphorylation on PDH (which is well known to track with NADH/NAD+) upon QC1 treatment and an increased sensitivity to UK5099 and 3FP in QC1 treated cells. However, these authors also show that QC1 rescues the toxicity of rotenone, which is also well known to impair PDH activity by increasing mitochondrial NADH, implying that neither route of acetyl-coa production is needed for self-renewal and the UK5099/3FP may be toxic for another reason. If the toxicity of QC1 and UK5099 or 3-FP is due to acetyl-coa deficiency, can it be rescued by acetate? Does the combination of QC1 with UK5099, 3-FP, or rotenone impact histone acetylation? More evidence is needed to claim that a TDH-to-PDH rewiring is occurring and necessary for acetyl-coa production, particularly if they want to claim this impacts self-renewal/cell proliferation.

Response:

We thank the reviewer for this important comment. To further substantiate our conclusion on TDH-to-PDH rewiring, we performed additional experiments. First, we confirmed the essential role of pyruvate in QC1-treated E14 cells, showing that removal of pyruvate severely impaired cell survival (Rebuttal Fig. 5A, B).

To verify that this dependence reflects pyruvate metabolism through PDH and acetyl-CoA production, we tested whether exogenous acetate could compensate for PDH blockade. As shown in Rebuttal Fig. 5C and D, inhibition of pyruvate transport into mitochondria (UK5099) or PDH activity (3-FP) markedly reduced proliferation and induced cell death in QC1-treated E14 cells, whereas supplementation with 10 mM acetate significantly rescued both effects. These findings indicate that acetyl-CoA deficiency underlies the observed phenotypes.

Finally, we examined histone acetylation by western blot. The combination of QC1 with UK5099 or 3-FP markedly decreased H3K27ac and H3K9ac levels, while the combination of QC1 with rotenone caused only a modest reduction (Rebuttal Fig. 5E).

Together, these new experiments directly address your concerns and provide compelling evidence that the metabolic rewiring from TDH to PDH is critical for maintaining acetyl-CoA levels and histone acetylation to support self-renewal in E14 cells.

We have incorporated these findings into the revised manuscript (new figures EV4D, and 3D and corresponding text on page 9, 10, lines 252–256 and 270–276).

Rebuttal Figure 5

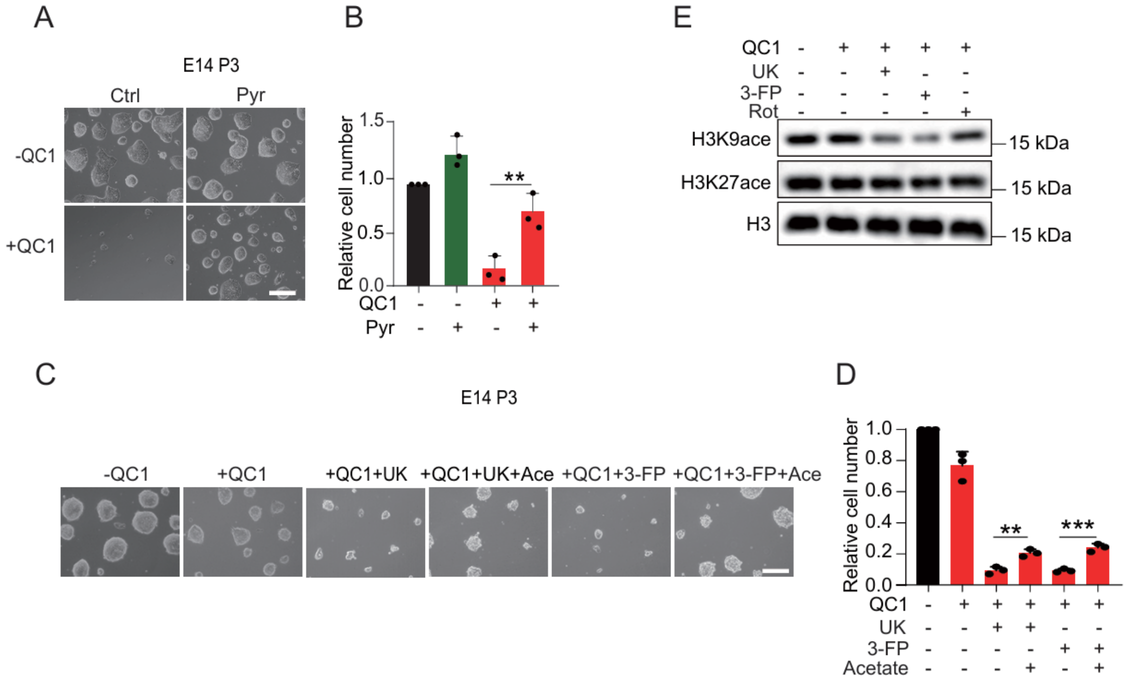

6) Similarly, a subsequent claim is that the histone hypomethylation is a result of THD inhibition impacting one carbon metabolism, by decreasing the availability of SAM. This hypothesis derives from cited work (Shyh-Chang et al., 2013) describing this effect, who showed that glycine/pyruvate treatment could ameliorate epigenetic dysregulation from impairments in threonine catabolism in mESCs. Can the authors determine if glycine/pyruvate is similarly able to restore epigenetic status and/or reverse DNA damage phenotypes?

Response:

We thank the reviewer for raising this relevant point. As noted, our culture media already contains normal levels of pyruvate (1.8 mM) and glycine (1.65 mM). To test whether they are insufficient to maintain normal epigenetic and genome stability, we supplemented QC1-treated E14 cells with higher concentrations of pyruvate (10 mM) and glycine (6 mM). Our results clearly showed that the extra of these two inhibits self-renewal of QC1-treated E14 cells, with completely no influence on DNA methylation and double-strand break levels (Rebuttal Fig 6A–F). These results indicate that, unlike the effects described in Shyh-Chang et al (2013), the epigenetic and genomic instability observed in our system are from a different reason.

We have incorporated these findings into the revised manuscript (new figure EV4H–J, and corresponding text on page10, 11 and lines 293–297).

Rebuttal Figure 6

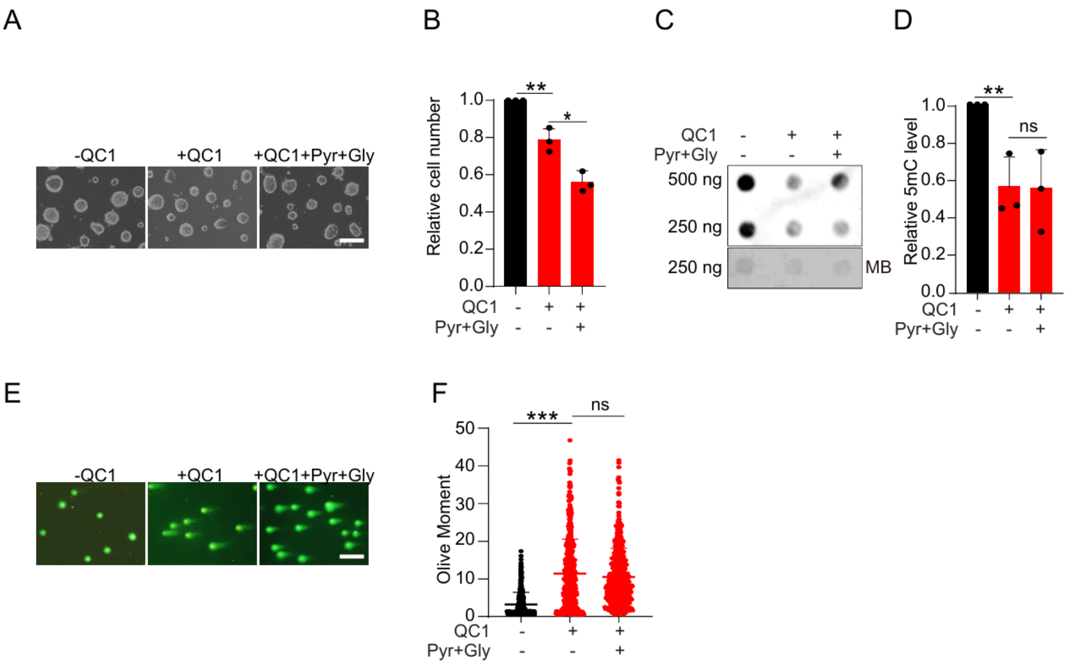

Minor Issues:

Some schematics of ETC dysfunction in Figure 1 would be helpful.

Response:

We thank the reviewer for this helpful suggestion. We have prepared a new schematic illustrating ETC dysfunction and incorporated it into the revised manuscript, new figure EV1A.

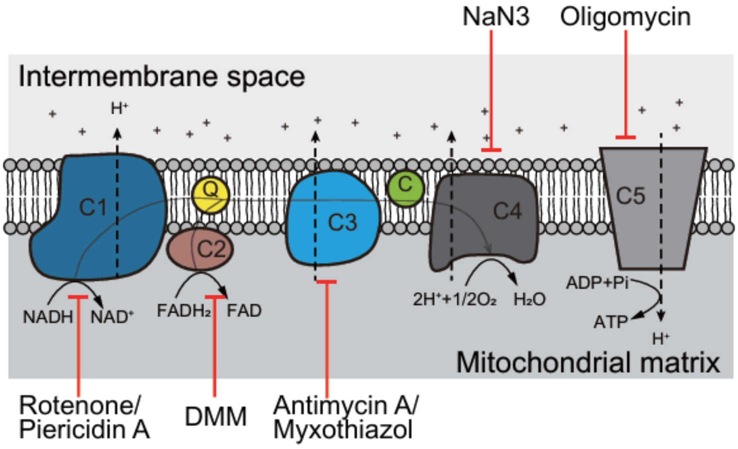

The statement that "The propensity to sustain strong reductive stress during ETC inhibition functions as a mitochondrial quality control mechanism in mESCs" is misleading, as it seems to imply that it activates a response pathway to repair intracellular mitochondria. Even knowing that it is instead referring the proliferative

disadvantage of these cells, it still implies a level of functional knowledge that has not been tested (i.e. if TDH were missing, would mice propagate dysfunctional mitochondria into progeny?). I would recommend carefully rewording this statement (and similar ones elsewhere in the text) to more accurately reflect that this is simple Darwinian selection against slow growing cells.

Response:

We agree with the reviewer and have revised the text accordingly to avoid overinterpretation. The original Results section subtitle and corresponding figure titles have been reworded to "**TDH-driven reductive stress during ETC inhibition favors cells with intact mitochondrial function in mESCs**" (Revised text, page 7, 38, 39 lines 189, 190 and 951, 952).

Referee #2:

General Summary and Opinion

This study investigates the role of NADH reductive stress in mediating developmental pausing induced by electron transport chain (ETC) inhibition in both mouse embryonic stem cells (mESCs) and human pluripotent stem cells (hPSCs). The authors demonstrate that ETC inhibition leads to a reversible paused pluripotent state, with reductive stress playing a central role and TDH identified as a key regulator of mitochondrial and nuclear stability. These findings extend to early mouse embryonic development, where ETC inhibition induces developmental pausing at the 2-cell (2C) stage and in blastocysts, offering insights into how cells with mitochondrial DNA (mtDNA) mutations may be selectively excluded.

The study is significant as it sheds light on the intersection of mitochondrial metabolism, redox balance, and cellular decision-making, particularly in early development and pluripotency. However, while the data presented is compelling, several major and minor issues need to be addressed to solidify the study's conclusions and enhance its translational relevance.

Response:

We sincerely thank the reviewer for the positive and thoughtful evaluation of our manuscript and for recognizing the significance of our findings in linking mitochondrial metabolism, redox balance, and pluripotency.

Major Concerns

1. Mechanistic Role of TDH in Reductive Stress

o The study convincingly demonstrates that TDH inhibition alleviates reductive stress, but its precise biochemical role in regulating NADH levels needs to be clarified.

o Experimental data examining the relationship between TDH, PDH, and other NADH-consuming enzymes is necessary to strengthen the central argument.

Response:

We thank the reviewer for raising this important point. To further clarify the biochemical role of TDH in redox regulation, we examined mitochondrial and glycolytic metabolism in QC1-treated E14 cells. As shown in the initial Figure 1N,

O and Rebuttal Figure 7A, TDH inhibition reduced OCR and markedly increased glycolysis via LDH induction, reflecting a compensatory response to maintain ATP production when mitochondrial NADH oxidation is compromised. This glycolytic activation was also observed under QC1 + ETC inhibition (Rebuttal Fig. 7B). Metabolomic profiling further revealed a trend toward increased lactate levels, although the difference between -QC1 and +QC1 conditions did not reach statistical significance because of biological variability (Rebuttal Fig. 7C), in line with enhanced LDH-dependent pyruvate reduction.

To evaluate whether LDH functionally contributes to the redox network and self-renewal in mESCs, we inhibited LDH using the specific inhibitor NHI-2 (Granchi et al, 2011). LDH inhibition alone impaired self-renewal and induced cytosolic reductive stress, and under ETC inhibition LDH was also required to maintain the partial redox balance and residual proliferation that are supported by pyruvate in the medium (Rebuttal Fig. 7D–F).

Rebuttal Figure 7

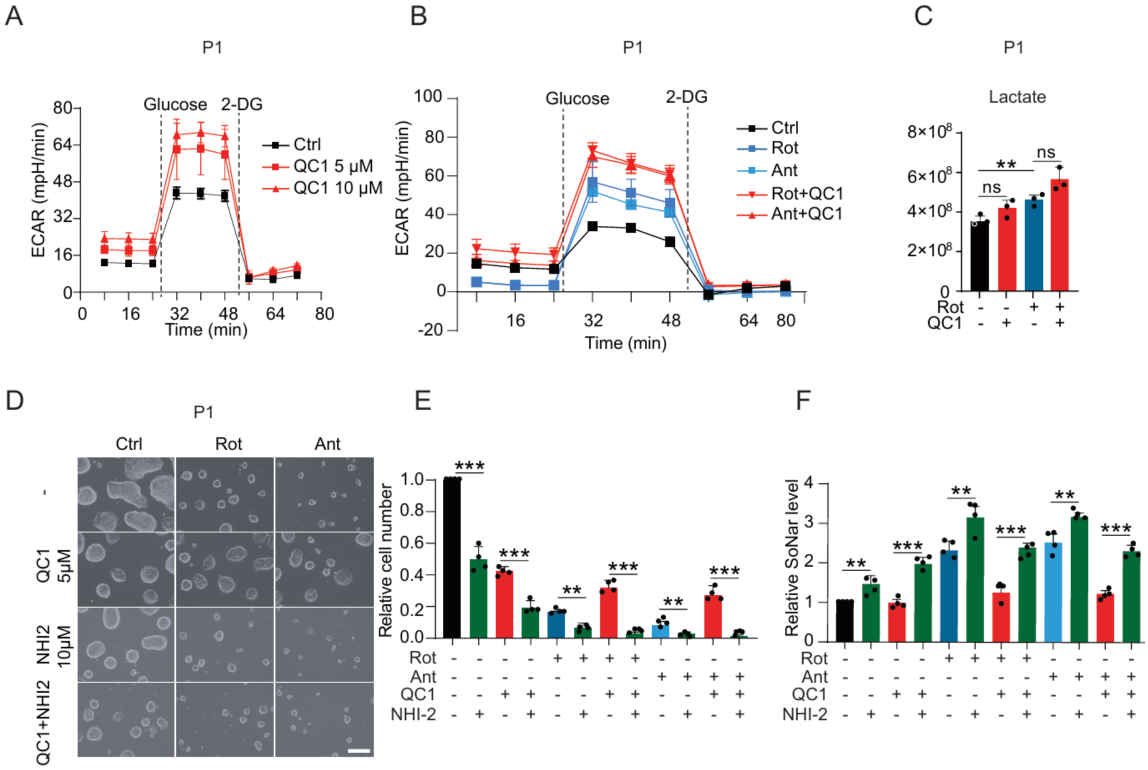

These measurements were obtained after one passage (P1). In earlier experiments, prolonged QC1 treatment (P3) eliminated intrinsic mitochondrial reductive stress and induced TDH-to-PDH metabolic rewiring (initial Fig. 3A–H). To test whether LDH is involved in this long-term adaptation, we inhibited LDH in QC1-treated cells for three passages. The QC1-induced OCR reduction, glycolytic increase, altered redox state, and reduced self-renewal all persisted (Rebuttal Fig.

7G–K). Because redox balance between compartments is coordinated via shuttles such as the malate-aspartate shuttle, we reasoned that LDH-dependent cytosolic reductive stress would propagate to mitochondria. Indeed, PDH phosphorylation increased under LDH inhibition in QC1-treated cells, demonstrating that LDH activity plays an integral—and at least partial—role in mediating TDH-to-PDH rewiring during TDH inhibition (Rebuttal Fig. 7L).

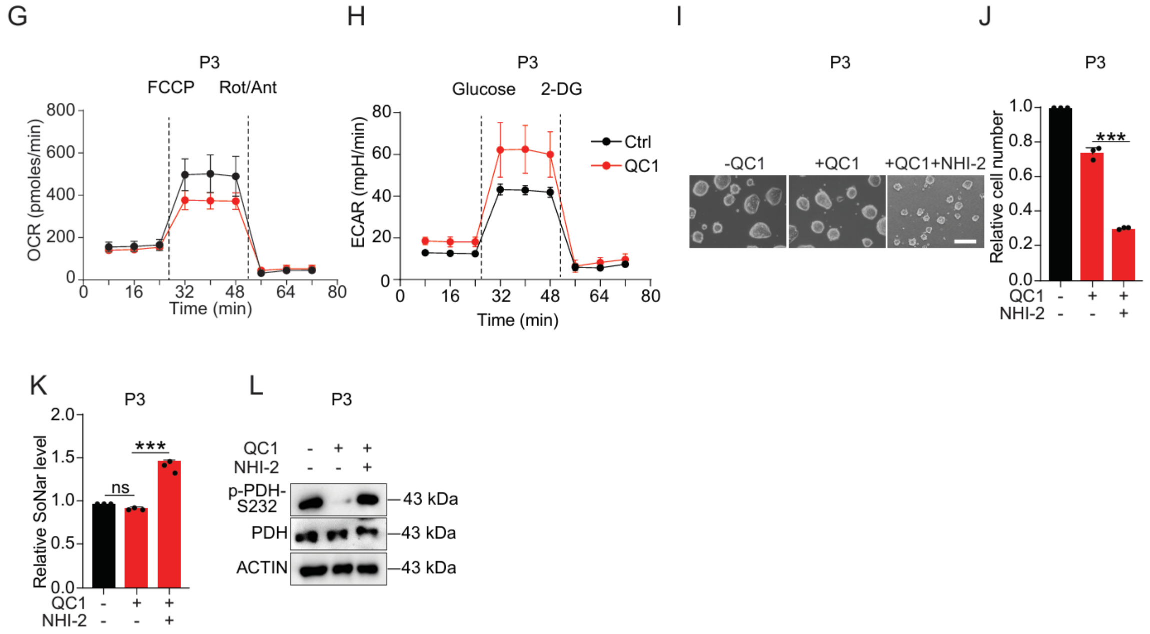

In conclusion, these data show that TDH inhibition leads to coordinated activation of LDH, glycolysis, and the PDH axis, linking these pathways into a unified redox network that shapes the proliferative response of mESCs.

We have incorporated these findings into the revised manuscript (new Figs 1O, EV2P–R, 3C, EV4A,B,E,F and corresponding text on page 7, 9, and 10 lines 166–168, 248–250, and 259–269).

2. Relevance to Human Biology

o Since TDH is a pseudogene in humans, the relevance of these findings to human biology needs to be addressed. The authors speculate that a similar mechanism may exist in human PSCs but provide no direct evidence.

o Investigating whether LDH could serve as a functional analog of TDH in humans is essential to establish translational relevance.

o Additional experiments assessing LDH activity and its role in human PSCs under ETC inhibition need to be included.

o Instead of just adding pyruvate to the media, lactate should be added. Lactate is not found in commercially available media, but is abundant in the circulation.

Pushing the LDH reaction in the opposite direction will produce more NADH. (see blow)

Response:

We thank the reviewer for raising this important point. The reviewer asked whether LDH could serve as a functional analogue of TDH in human ESCs. To address this, we first examined ECAR, a common indicator of LDH activity, in ETC inhibitor–treated H9 cells. As shown in Rebuttal Figure 8A, ETC inhibition induced a modest but significant increase in ECAR, although the effect was weaker than in mESCs. This suggests that, similar to mESCs, LDH in hESCs mainly operates in the forward direction, converting pyruvate to lactate rather than the reverse.

Next, we tested whether lactate affects redox balance and self-renewal during ETC inhibition. As shown in Rebuttal Figure 8B–D, exogenous lactate markedly increased cytosolic reductive stress (SoNar) and reduced self-renewal, indicating that while lactate oxidation can occur when sufficient substrate is provided, its activity is normally minimal or suppressed under both basal and ETC-inhibited conditions. We also examined pyruvate's role. Since pyruvate cannot be removed from mTeSR medium, we instead supplemented additional pyruvate in H9 cells under control or ETC-inhibited conditions. No changes in self-renewal were observed (Suppl. Fig. 4G of the initial manuscript), suggesting that pyruvate in the culture medium is already sufficient and that LDH operates near maximal capacity to convert pyruvate into lactate, thereby regenerating NAD^+ rather than producing NADH. Together, these results indicate that LDH does not function as a TDH analogue in hESCs.

Rebuttal Figure 8

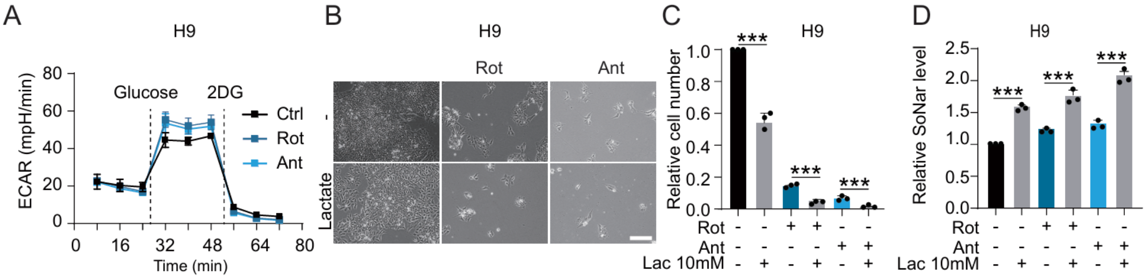

To further investigate this issue, we followed the rationale established in mESCs that certain dehydrogenases enriched in hESCs might function similarly to TDH by continuously generating NADH under reductive stress. Previous studies have shown that the one-carbon (1C) pathway enzyme MTHFD2 supplies NADH when the ETC is inhibited and that depletion of MTHFD2 or inhibition of its upstream

enzymes SHMT1/2 can partially rescue proliferation defects (Yang et al, Cell Metab, 2020). We therefore compared the mRNA expression of 1C pathway enzymes between hESCs and human embryonic fibroblasts (HEFs) using publicly available RNA-seq data (Guan et al, Nature, 2022). Most 1C enzymes were upregulated in hESCs, with particularly strong enrichment of glycine cleavage system (GCS) components (Rebuttal Fig. 8E). As illustrated in Rebuttal Figure 8F, in addition to SHMT1/2, the GCS provides an alternative source of 1C units that feed into this pathway.

Because no specific GCS inhibitor is available, we used inhibitors targeting SHMT1/2 (SHIN1) and MTHFD2 (DS18561882). Each inhibitor was tested individually in both H9 and HCT116 cells. As shown in Rebuttal Figure 8G–J, during ETC inhibition, both SHIN1 and DS partially rescued proliferation and SoNar signals in HCT116 cells, but neither was effective in H9 cells. Given the enrichment of GCS in hESCs, we reasoned that combining the two inhibitors might produce a synergistic effect. Indeed, as shown in Rebuttal Figure 8K–N, combined treatment with DS and SHIN1 (1 μM each) significantly rescued the self-renewal of rotenone-treated H9 cells, accompanied by a pronounced reduction in total NADH/NAD^+ ratio.

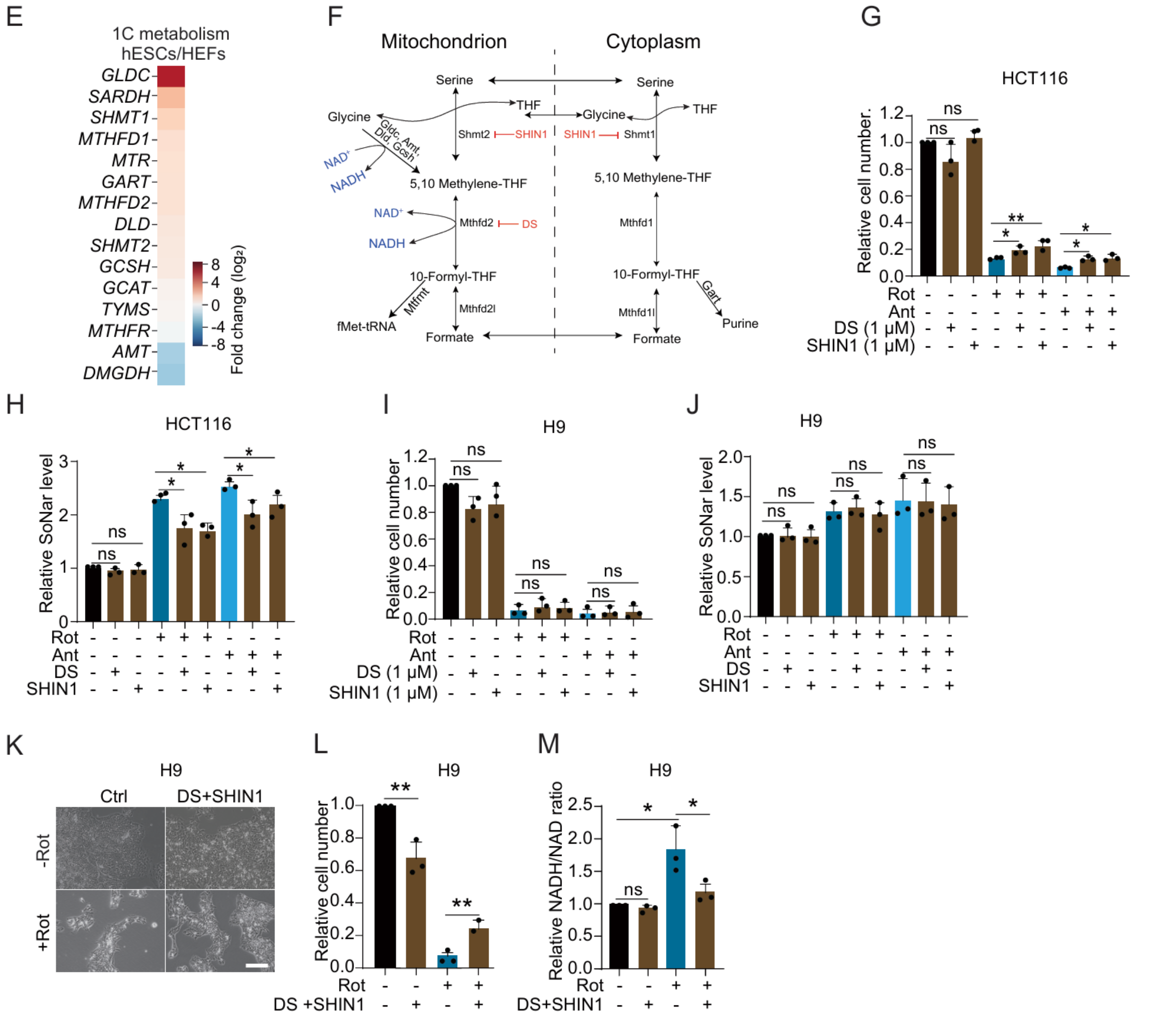

In summary, our new results identify highly active one-carbon metabolism—particularly its mitochondrial branch—as the pathway responsible for driving the paused phenotype in hESCs under ETC inhibition, thereby providing a mechanistic link between the mouse and human systems.

We have incorporated these findings into the revised manuscript (new Figs 4J–M, EV5M,N and corresponding text on page, 2, 13, and lines 38–42, 371–387).

3. Role of the Mitochondrial NAD Transporter (SLC25A51)

o Given the centrality of NADH in the proposed mechanism, the role of the mitochondrial NAD transporter SLC25A51 needs to be assessed. The authors should evaluate its contribution by inhibiting or knocking it out to clarify its involvement.

Response:

We thank the reviewer for raising this relevant point. To examine the role of SLC25A51 in redox balance and self-renewal, we inhibited its function using the

reported chemical inhibitor fludarabine phosphate (Flu) (Li et al, 2023, Cell Death & Differentiation) and by shRNA-mediated knockdown in E14 cells. Flu treatment markedly reduced self-renewal and caused a moderate but significant decrease in SoNar signals, indicating that blocking NAD^+ transport into mitochondria impairs self-renewal and leads to NAD^+ accumulation in the cytosol (Rebuttal Fig. 9A–C). Consistently, SLC25A51 knockdown also significantly reduced self-renewal and strongly suppressed mitochondrial metabolism, as measured by OCR (Rebuttal Fig. 9D–G). Under ETC inhibition, the effects of Flu and ETCi on self-renewal were additive, with cytosolic NADH/NAD^+ further reduced, consistent with a more severe block of mitochondrial metabolism (Rebuttal Fig. 9H–J). Together, these data support a key role for SLC25A51 in maintaining redox balance between cytosol and mitochondria, thereby sustaining mitochondrial metabolism and self-renewal.

In line with this, our published RNA-seq datasets (Huang et al, Nat Commun 2020) show that *Slc25a51* mRNA expression is lower in mESCs than in MEFs (Rebuttal Fig. 9K). This suggests that NAD^+ transport into mitochondria could be rate-limiting and may contribute to the reductive stress of mESCs. We further envisage that overexpressing this transporter might partially alleviate mitochondrial reductive stress and rescue the paused phenotype in ETCi-treated mESCs. Due to logistical constraints, however, we were unable to obtain *Slc25a51* cDNA for overexpression experiments.

In view of the relatively weak connection between SLC25A51 and our main findings, we decided not to include these results in the revised manuscript.

Rebuttal Figure 9

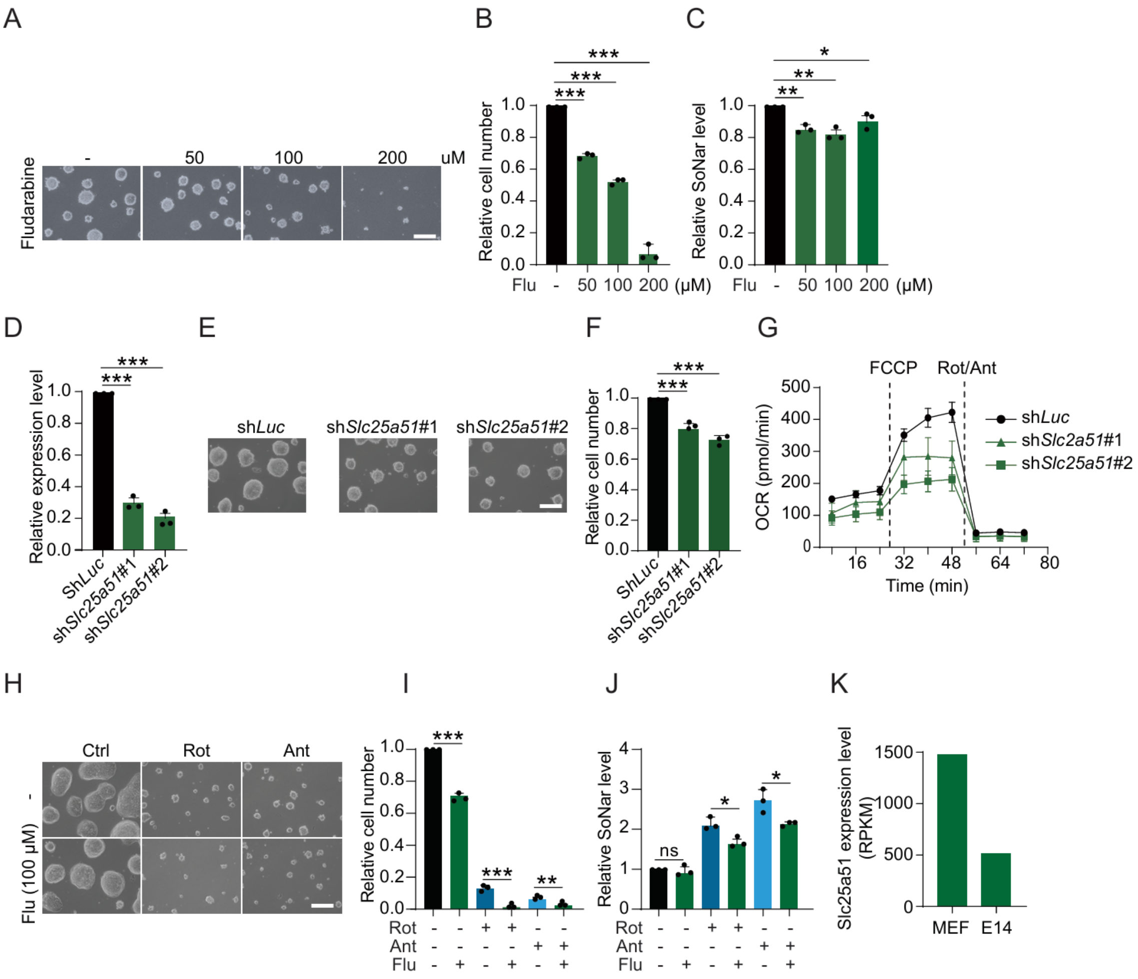

4. Validation of Experimental Tools

o The use of inhibitors (e.g., QC1, Rot, Ant, UK5099) requires validation to ensure specificity. Non-specific effects of these inhibitors could confound the results.

o All inhibitor experiments need to be complemented with knockout (KO) data to confirm that the observed effects are due to the intended targets.

Response:

We thank the reviewer for this important comment regarding the validation of inhibitor specificity.

For **QC1**, in addition to demonstrating inhibition of TDH activity (initial Suppl. Fig. 1K), we performed several complementary validation experiments. As shown in Rebuttal Figure 2G-I, metabolomic profiling revealed that QC1 treatment led to threonine accumulation under both basal and ETC-inhibited conditions—a direct metabolic consequence of TDH inhibition. Moreover, as shown in Rebuttal Figure 2J, threonine withdrawal in mESCs partially rescued the ETCi-induced pause, indicating that reducing threonine flux through TDH produced a similar, though

weaker, rescue effect. Finally, as shown in Rebuttal Figure 7A and B, QC1 treatment significantly enhanced glycolysis, consistent with a compensatory response to reduced mitochondrial NADH input and ATP production—an expected outcome of TDH inhibition rather than a non-specific mitochondrial effect. Together, these findings strongly support that QC1 acts specifically through inhibition of TDH.

For **rotenone** (**Rot**) and **antimycin A** (**Ant**), we confirmed their canonical activities by OCR and ECAR measurements: both inhibitors completely blocked oxygen consumption and increased glycolysis, as expected (Rebuttal Fig. 10A, B). Moreover, expression of the rotenone-insensitive yeast NADH dehydrogenase NDI1 rescued Rot-induced defects in cell proliferation, cytosolic redox state (SoNar), and mitochondrial respiration (OCR) in E14 cells (Rebuttal Fig. 10C–F). In addition, we tested other widely used ETC inhibitors, Piericidin A (Pier) for C1 and Myxothiazol (Myxo) for C3, Sodium azide (NaN_3) For C4 and Oligomycin (Oligo) for C5 and examined rescue by QC1. These results fully corroborated our earlier conclusions (Rebuttal Fig. 10G–I).

For **UK5099** and **3-FP**, as shown in the Rebuttal Figure 5C and D, the ability of acetate supplementation to rescue the observed defects further confirmed that their effects were due to blockade of pyruvate entry into the TCA cycle.

Rebuttal Figure 10

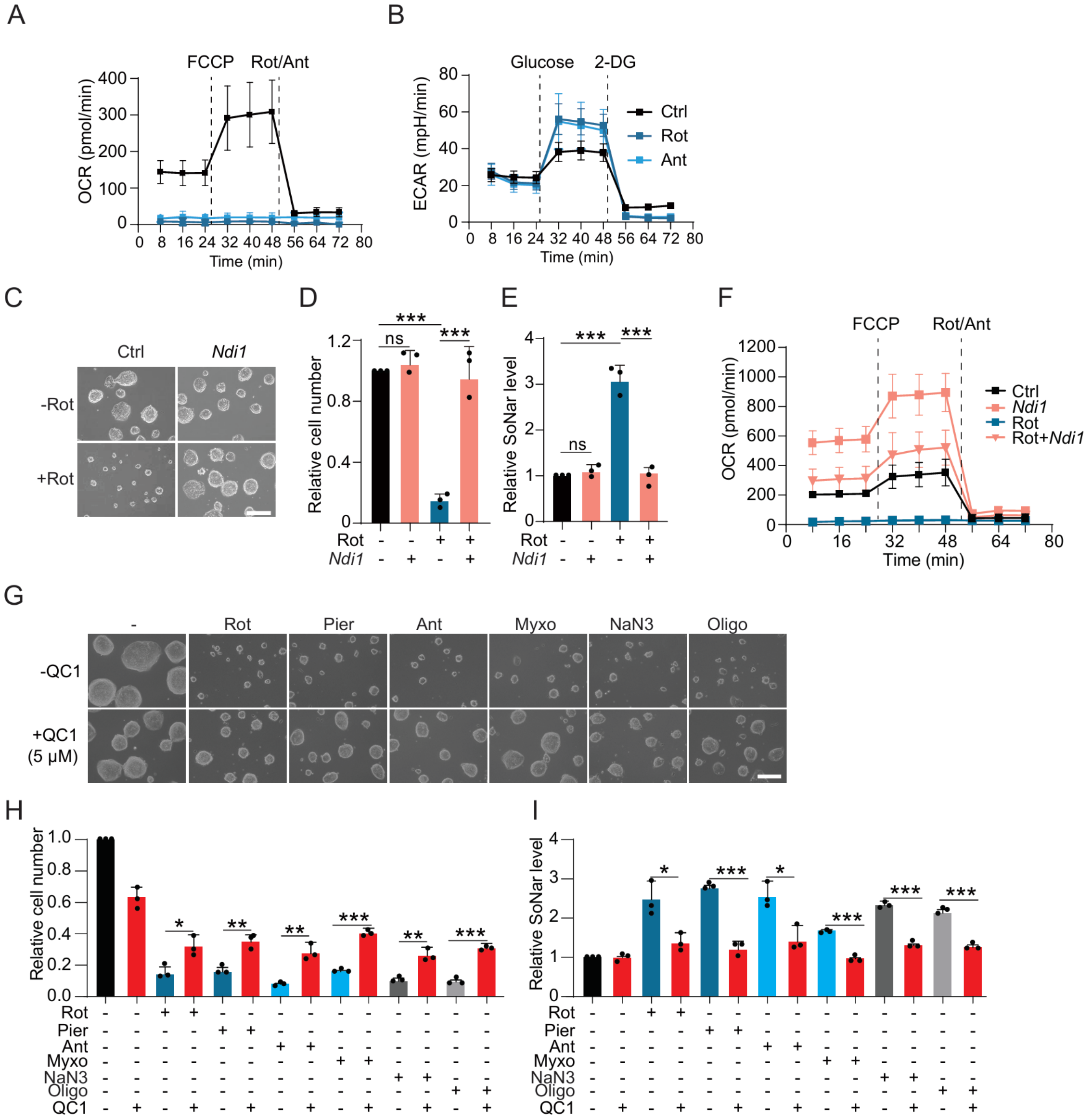

Overall, these multiple lines of evidence demonstrate that the inhibitors used in our study act on their intended targets and that our conclusions are not confounded by non-specific effects. We therefore believe that generating additional KO cell lines is not essential for confirming the specificity of these findings.

We have incorporated these findings into the revised manuscript (new Fig. EV2G–I and corresponding text on page 7, line 169–171).

5. Metabolic Pathway Directionality

o Steady-state metabolomics does not confirm pathway directionality. The authors need to incorporate 13C-tracing experiments, such as using 13C-glucose, to definitively determine reaction fluxes.

Response:

We thank the reviewer for this important point. We understand that this comment refers to the metabolomic data presented in our initial Figure S1F and seeks additional evidence regarding metabolic flux and the interplay among LDH, TDH, and PDH in the ETCi-induced pause phenotype of mESCs. We fully agree that ^{13}C-tracing is the most direct way to define pathway fluxes. However, due to technical and logistical limitations, we were unable to perform isotope-labeling experiments in the current study. Instead, we sought to address the reviewer's concern by carrying out a series of complementary assays. These included metabolomics profiling (Rebuttal Fig. 2G–I), threonine withdrawal experiments (Rebuttal Fig. 2J), acetate rescue (Rebuttal Fig. 5C, D), glycolytic activity measurements by ECAR (Rebuttal Fig. 7A, B), and LDH inhibition studies (Rebuttal Fig. 7D–K). Together, these results consistently support our conclusion that TDH functions as a central redox node in mESCs and that its inhibition induces global rewiring of the redox network underlying the observed phenotypic changes.

6. Lactate as a Potential Modulator

o The authors need to explore the addition of lactate to the media to test its impact on NADH generation via the LDH reaction. This is critical given the central role of NADH in the study and lactate's abundance in vivo.

Response:

We thank the reviewer for this valuable suggestion. We examined the effect of lactate supplementation in E14 cells under basal conditions and during ETC inhibition. Lactate alone elevated SoNar signals and suppressed cell proliferation (Rebuttal Fig. 11A–C). When combined with ETC inhibitors, lactate further exacerbated reductive stress and reduced self-renewal, consistent with its role in driving NADH generation through the LDH reaction, similar to our findings in H9 cells. We also anticipate that lactate addition could re-establish reductive stress and re-inhibit PDH activity in QC1-treated E14 cells, in line with the effects observed using LDH inhibition (Rebuttal Fig. 7H–K).

In summary, exogenous lactate intensifies reductive stress via LDH, highlighting pyruvate–lactate balance as a simple lever to regulate cellular metabolism and state. We have emphasized this point in the revised Discussion.

Rebuttal Figure 11

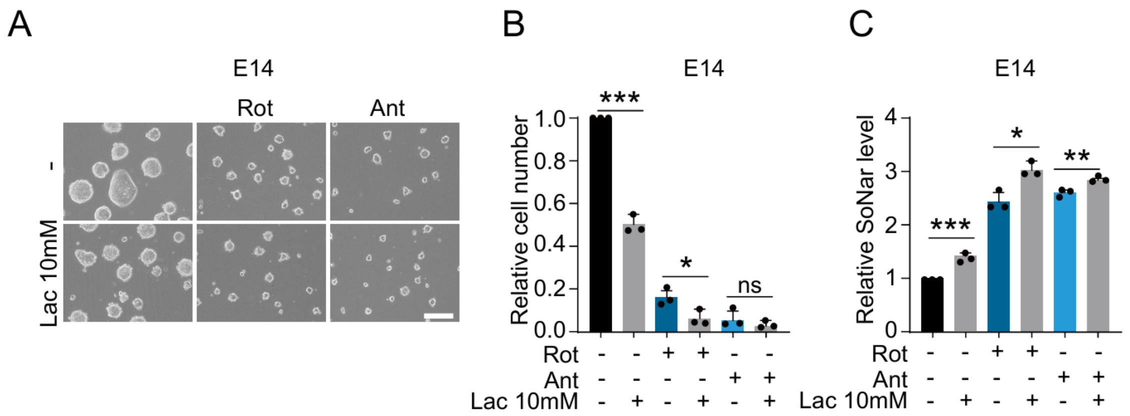

7. Mitochondrial Health and Function

o Additional measures of mitochondrial health, such as blue native gels to analyze ETC complexes and supercomplex formation, are necessary to support mitochondrial function conclusions.

o Assessing mitochondrial pyruvate carrier (MPC1 and MPC2) levels is necessary to better interpret the observed effects.

Response:

We thank the reviewer for raising this important point. We understand the concern as primarily relating to the ETCi-induced pause and the *Polg*-mutant induced mitochondrial quality models of mESCs. Although we were unable to perform blue native gel analysis of ETC complexes and supercomplexes due to logistical constraints, we assessed mitochondrial function in the *Polg*-mutant model by OCR measurements (Rebuttal Fig. 12A, B). These results are consistent with the observed mitochondrial mutation patterns and support our conclusions on impaired mitochondrial activity. In line with this, proliferation assays showed that cells with a higher burden of mitochondrial mutations proliferate more slowly than those with fewer mutations, indicating that eliminating the TDH-driven intrinsic reductive stress leads to increased mtDNA mutations and, consequently, reduced mitochondrial function and proliferation capacity (Rebuttal Fig. 12B).

For the ETCi-induced pause model, in addition to OCR and ECAR measurements (Rebuttal Fig. 10A, B), we analyzed transcriptomic changes by RNA-seq. As shown in Rebuttal Figure 12C, mitochondrial genes, including ETC complex subunits, TCA enzymes, and the pyruvate carrier genes (*Mpc1* and *Mpc2*), remain largely unchanged in Rot- and Ant-induced paused E14 cells.

Finally, we directly assessed *Mpc1* and *Mpc2* expression by RT-qPCR in QC1-treated E14 cells, which undergo TDH-to-PDH metabolic rewiring. We observed no significant changes, indicating that this rewiring is not attributable to altered *Mpc1* and *Mpc2* expression (Rebuttal Fig. 12D).

Rebuttal Figure 12

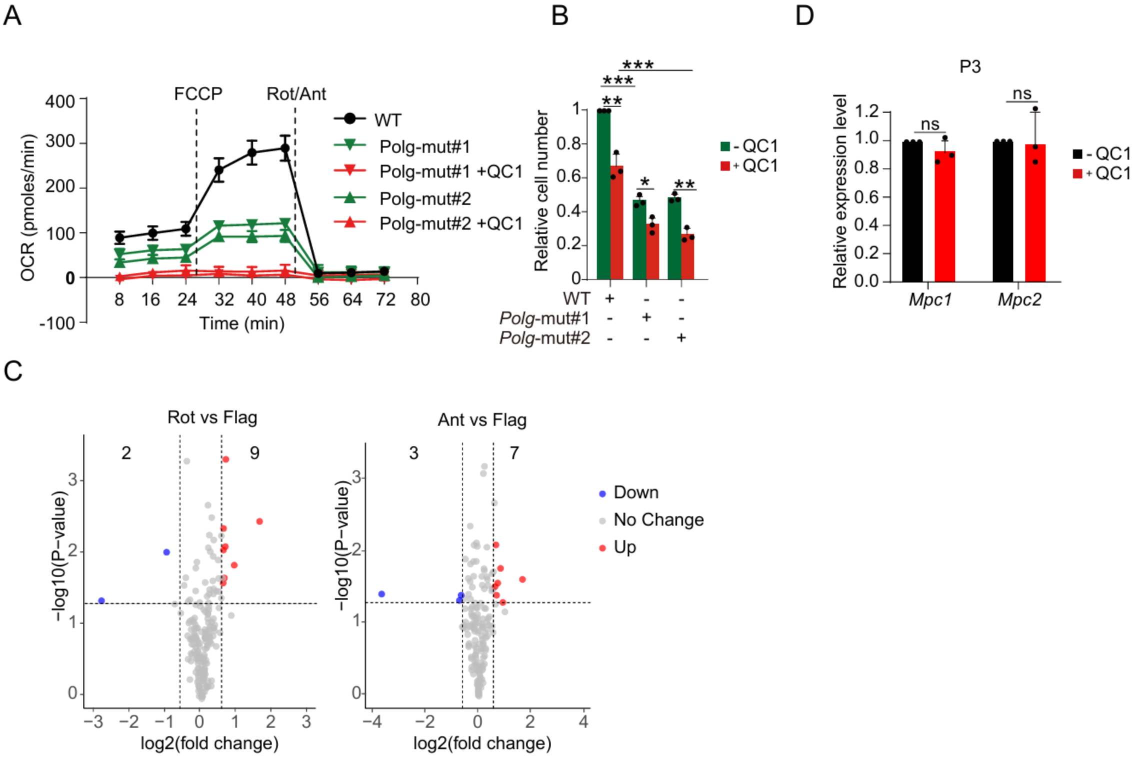

Minor Concerns

1. Use of Acronyms

o The authors use acronyms throughout the paper without first introducing them, making the manuscript difficult to follow and potentially confusing for readers unfamiliar with the field.

Response:

We thank the reviewer for pointing this out. We have revised the manuscript to ensure that all acronyms are defined at first use.

2. Normalization and Cell Viability

o The authors need to ensure that all metabolomics data, including NADH and NAD+, is normalized to cell number or protein content. If this was done, it should be clearly stated in the text.

o Cell viability assays need to accompany all experiments to exclude cell death as

a confounding factor.

Response:

We appreciate the reviewer's comment. All metabolomics data, NADH/NAD^+ measurements as well as OCR/ECAR assays were normalized to cell number. This has now been clarified in the Methods, page 26–30, and line 588,589,606,664. To assess cell viability, we measured apoptosis in ETCi-treated mouse and human ESCs, using streptozotocin (STZ) as a positive control. As shown in Rebuttal Figure 13A and B, Rot and Ant did not induce apoptosis in mESCs. In H9 cells, Rot and Ant increased apoptosis by approximately twofold compared with untreated controls, which already display higher basal apoptosis than mESCs; however, the levels remained markedly lower than those induced by STZ (Rebuttal Fig. 13C). These results confirm that cell death was not a confounding factor in our study.

Rebuttal Figure 13

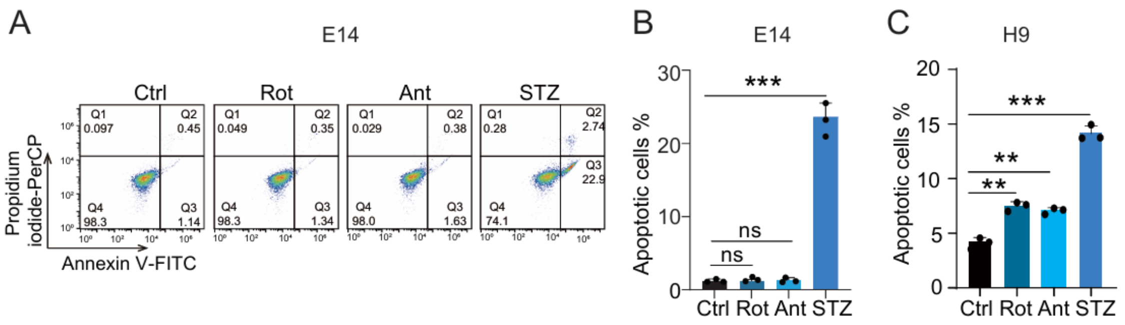

3. Consistency in Experimental Design

o PDK inhibitors, PDH activation, and MPC modulation are used inconsistently across experiments. A more uniform approach is needed to strengthen the study's conclusions.

Response:

We thank the reviewer for highlighting this important point. We acknowledge that different perturbations were used in various parts of the study to address specific mechanistic questions. With the new data obtained in the QC1-induced TDH-to-PDH model—showing that acetate rescues cell proliferation and survival in the UK5099/3FP context (Rebuttal Fig. 5), and that LDH inhibition re-establishes reductive stress and suppresses PDH activity (Rebuttal Fig. 7)—we believe our

original conclusion is well supported and the proof-of-principle concept validated. We also appreciate the reviewer's suggestion for a more unified experimental framework and will aim to further harmonize these approaches in future work to enhance consistency across models.

4. Controls for High-Dose Treatments

o The controls used for high-dose treatments (e.g., QC1, Rot) need to be clarified. Were alternative ETC inhibitors tested to confirm specificity?

Response:

We thank the reviewer for raising this point. Appropriate vehicle controls (e.g., DMSO) were included in all experiments. Stock solutions of inhibitors were prepared at high concentrations, and equivalent volumes of solvent were added to control samples to match the highest doses used. In addition, to confirm the specificity of the observed effects, we tested several other widely used ETC inhibitors—Piericidin A (Complex I), Myxothiazol (Complex III), sodium azide (Complex IV), and oligomycin (Complex V). The results were fully consistent with those obtained using rotenone and antimycin A, further supporting our conclusions (Rebuttal Fig. 10G–I).

We have incorporated these findings into the revised manuscript (new Fig. EV2 G–I and corresponding text on page 7, line 169–171).

5. Replicates

o The number of biological replicates, particularly in animal experiments, needs to be increased to improve statistical confidence in the findings.

Response:

We thank the reviewer for this important suggestion. We understand the concern as primarily relating to Figures 4B and C. In Figure 4B, only representative embryo images were shown, which may have given the impression that the sample size was small. In fact, these images were cropped from larger fields containing many more embryos. We now provide the full images (Rebuttal Fig. 14A) together with detailed embryo counts (Rebuttal Fig. 14B). Although variability among embryos is high, the statistical significance remains clear.

In addition, we have repeated the embryo experiments corresponding to the original Figures S4D and S4E with increased embryo numbers, and the revised data now provide stronger statistical confidence.

Rebuttal Figure 14

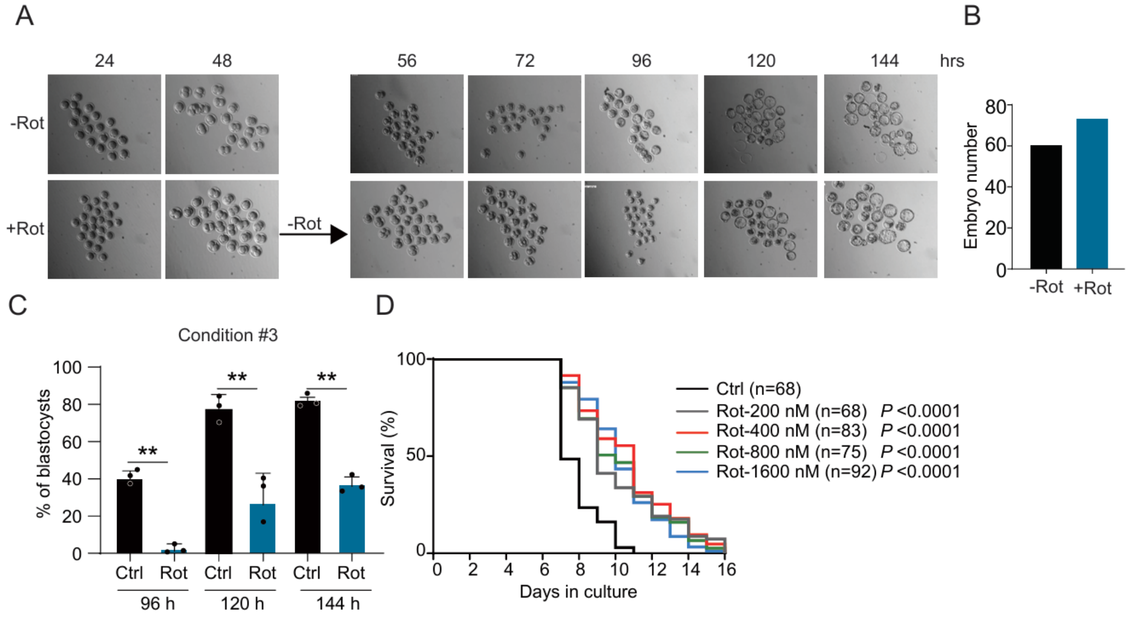

We have incorporated these new data into the revised manuscript (new Fig. EV5C,E).

Non-Essential Suggestions

1. Epigenetic and Transcriptomic Analysis

o Including bisulfite sequencing to assess methylation changes and RNA-seq to confirm transcriptional alterations would provide a more comprehensive understanding of downstream effects.

Response:

We thank the reviewer for this valuable suggestion. While we were unable to perform bisulfite sequencing and RNA-seq analyses in the QC1 model at this stage, we fully agree that such approaches would provide deeper insights into the downstream epigenetic and transcriptional consequences of TDH inhibition.

2. Mitochondrial Quality Control Mechanisms

o The discussion briefly mentions mitochondrial DNA mutation accumulation.

Expanding this to include pathways like mitochondrial autophagy would enhance the study's broader implications.

Response:

We thank the reviewer for raising this relevant point. To address it, we assessed mitochondrial autophagy using the mitoQC reporter, a tool we employed in our earlier study (Wang et al, FEBS J, 2019), in the *Polg*-mutant models. As shown in Rebuttal Figure 15A and B, mitophagy was markedly reduced in QC1-treated cells, both in wild-type and *Polg*-mutant backgrounds. We have incorporated these results as new Figure EV3G, H to highlight their physiological significance in revised text (page 8, 9 lines 226–233).

Rebuttal Figure 15

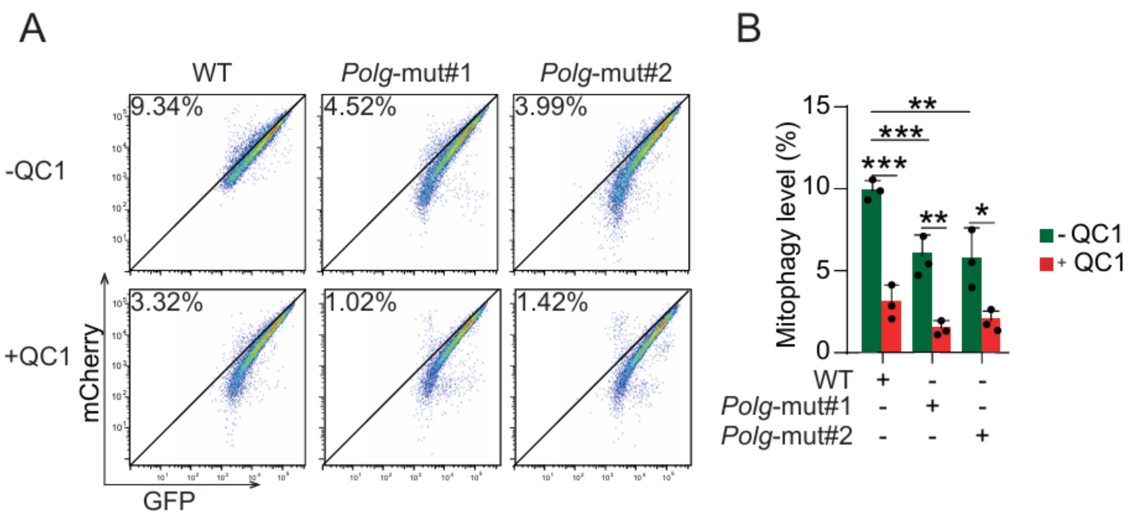

3. Generalizability

o The authors could discuss whether the mechanisms observed in PSCs are likely to apply to differentiated cells or other stem cell types.

Response:

We thank the reviewer for this suggestion. We have expanded the discussion to address the broader implications of our findings, including the potential relevance of these mechanisms to differentiated cells and other stem cell types (refer to revised texts, page 14, lines 398–408).

Dear Dr Qin,

Thank you for submitting your amended appeal manuscript (EMBOJ-2024-119405R-Q) to The EMBO Journal, together with a rebuttal point-by-point response to the issues raised during peer-review. As mentioned, your revised study was sent back to the previous referees for their scientific reassessment, and we have received detailed re-reports from both of them, which I enclose below. As you will see, the referees state that the work has been enhanced by the revision and they are now in favour of publication, pending reasonable additional revision.

Thus, we are pleased to inform you that we concluded that we can invite you to amend your manuscript according to expert #2's input, to the progress towards publication in The EMBO Journal.

Please carefully consider the remaining points raised by referee #2 by adding revisiting data presentation and statistics, and by introducing caveats and relativise claims in the manuscript text where appropriate.

Also, we now need you to take care of a number of minor issues related to formatting and data annotation, which I will share shortly in a separate message, together with additional changes and requests by our production team for Source Data provision.

Please submit a revised version of the manuscript using the link enclosed below, addressing the advisor's comments.

Thank you again for giving us the chance to consider your manuscript for The EMBO Journal, I look forward to hearing from you and receiving your final revised version of the manuscript.

Kind regards,

Daniel Klimmeck

Daniel Klimmeck PhD
Senior Editor
The EMBO Journal

Instructions for preparing your revised manuscript:

Read our guidance for manuscript revisions and related editorial policies:
https://link.springer.com/journal/44318/submission-guidelines#cms-Revised-submissions

Please also check that the title and abstract of the manuscript are brief, yet explicit, even to non-

specialists.

When assembling figures, please refer to our figure preparation guideline in order to ensure proper formatting and readability in print as well as on screen:
https://media.springernature.com/original/springer-cms/rest/v1/content/27825798/data/v1

IMPORTANT: When you send the revision we will require
- a point-by-point response to the referees' comments, with a detailed description of the changes made (as a word file).
- a word file of the manuscript text.
- individual production quality figure files (one file per figure)
- a complete author checklist
- Expanded View files (replacing Supplementary Information)
- a Reagents and Tools Table as part of the Methods section

Please remember: Digital image enhancement is acceptable practice, as long as it accurately represents the original data and conforms to community standards. If a figure has been subjected to significant electronic manipulation, this must be noted in the figure legend or in the 'Methods' section. The editors reserve the right to request original versions of figures and the original images that were used to assemble the figure.

We realize that it is difficult to revise to a specific deadline. In the interest of protecting the conceptual advance provided by the work, we recommend a revision within 3 months (3rd May 2026). Please discuss the revision progress ahead of this time with the editor if you require more time to complete the revisions.

Referee #1:

In this revised manuscript, the authors provide further evidence for their mechanism that TDH activity can impact the redox status of mESCs, such that the defects of mitochondrial dysfunction can no longer be surmounted by treatment with electron acceptors.My biggest issues with the prior draft were some overstated claims, missing controls, and some seemingly unfair experimental comparisons. The authors have done a commendable job of resolving those issues in the revised manuscript and response to reviewers document. As such, my major concerns have been alleviated and I believe that the revised manuscript is acceptable for publication.

Referee #2:

General summary and opinion

This revised manuscript investigates how mitochondrial electron transport chain inhibition induces a

reversible paused state in pluripotent stem cells and proposes that sustained NADH reductive stress underlies this response. The authors identify threonine dehydrogenase as a major contributor to reductive stress in mouse embryonic stem cells and propose that distinct metabolic pathways generate a similar redox burden in human pluripotent stem cells. The study further links intrinsic reductive stress to suppression of pyruvate dehydrogenase activity, altered acetyl CoA availability, and increased genomic and epigenetic instability when pausing is relieved.

The revision includes substantial new experimental work that addresses many of the concerns raised in the initial review, particularly regarding redox regulation, PDH dependence, and relevance to human pluripotent stem cells. Overall, the study tackles an important question at the intersection of mitochondrial metabolism, redox biology, and cell state regulation, and the experimental effort in the revision is significant. However, several key conclusions remain overstated relative to the data presented, primarily due to language that implies deliberate, programmed, or conserved quality control mechanisms without direct evidence. These issues largely concern interpretation and framing rather than missing experiments, but they are essential to address in order to fully support the manuscript's conclusions.

Major concerns

Overinterpretation of deliberate or programmed quality control mechanisms

Throughout the Abstract, Results, and Discussion, the manuscript describes sustained reductive stress as a deliberate or systemic quality control mechanism that preserves genomic and epigenetic stability. While the data convincingly show that reductive stress correlates with proliferative pausing and that relieving this stress permits proliferation at the cost of increased genomic instability, the study does not demonstrate that cells actively induce reductive stress as a regulated quality control pathway. The data are more consistent with intrinsic metabolic constraints and population level selection against slower growing cells.

The authors should revise the language throughout the manuscript to avoid implying intent or programming. Phrasing that emphasizes intrinsic metabolic constraints or selection like effects is more appropriate and consistent with the evidence.

Strength of causal claims regarding threonine dehydrogenase in mouse embryonic stem cells

The revised data support an important role for threonine dehydrogenase in contributing to NADH burden and limiting the effectiveness of electron acceptors in mouse embryonic stem cells. However, the manuscript often presents TDH as the primary or dominant driver of the paused state. This conclusion is stronger than warranted given that pyruvate and TDH inhibition each partially reduce reductive stress, and that rescue effects are context dependent.

In addition, several datasets used to support TDH specificity and dominance are presented as representative examples with n equal to one. While these data are informative, they should not be used to justify strong quantitative or exclusive claims. The authors should recalibrate the language to describe TDH as a major contributor rather than the sole or primary determinant of pausing.

Human pluripotent stem cell mechanism is not pathway conserved

The authors propose that reductive stress is a conserved driver of pausing in both mouse and human pluripotent stem cells, but that the metabolic source of NADH differs between species. The new data showing that combined inhibition of SHMT1 or SHMT2 and MTHFD2 rescues self renewal and reduces NADH to NAD plus ratio in human pluripotent stem cells represent a significant advance.

However, the manuscript currently states that mitochondrial one carbon metabolism is identified as the driver of reductive stress in human pluripotent stem cells. Given that this conclusion is based on pharmacologic interactions rather than direct flux measurements or genetic perturbations, this language is too strong. The authors should rephrase these claims to state that the data support a model in which mitochondrial one carbon metabolism contributes to reductive stress in human pluripotent stem cells.

Interpretation of LbNOX versus Ndi1 experiments

The comparison between Ndi1 expression and lack of rescue by LbNOX is used to argue that unresolved NADH to NAD plus imbalance is the principal driver of the paused state in mouse embryonic stem cells. While these experiments are informative, the failure of LbNOX to rescue may reflect limited enzyme activity, compartmental access, or inability to access the relevant NADH pool, rather than persistence of reductive stress per se.

The authors should acknowledge these alternative interpretations and avoid presenting the LbNOX results as definitive evidence of redox causality.

Use of metabolic rewiring language without flux based evidence

The manuscript frequently describes TDH to PDH or LDH to PDH metabolic rewiring. Although functional dependence on PDH and acetyl CoA is well supported by acetate rescue and PDH inhibition experiments, no isotope tracing data are presented to establish metabolic flux or pathway directionality.

The authors should replace rewiring terminology with language describing inferred metabolic adaptation or functional dependence unless flux based evidence is added.

Minor concerns

Lactate supplementation experiments are discussed conceptually and were referenced in the rebuttal, but corresponding data are not clearly presented in the revised manuscript. If these experiments were performed, the data should be included, even as supplementary figures, as they directly support the proposed redox model.

Sample sizes for key experiments such as karyotyping and some metabolic measurements are low and should be clearly indicated in figure legends and discussed cautiously in the text.

The manuscript relies largely on unpaired two tailed Student's t tests despite multiple comparisons and dose response analyses. Clearer justification or more consistent statistical handling would strengthen confidence in the conclusions.

The embryo pausing experiments are compelling at the phenotypic level, but mechanistic links to threonine dehydrogenase or one carbon metabolism are not directly tested. The manuscript should avoid implying mechanistic equivalence and instead frame these findings as consistent with, but not definitive proof of, the proposed model.

Non essential suggestions

Genetic perturbation of threonine dehydrogenase or flux based isotope tracing in future work would further strengthen conclusions regarding pathway specificity and directionality.

A schematic explicitly distinguishing mouse specific and human specific sources of NADH while emphasizing shared redox logic would improve clarity for readers.

Expanded discussion of whether similar redox driven pausing mechanisms may operate in differentiated cells or other stem cell types would broaden the impact of the study.

Dear Dr Qin,

Further to below, I attach the mentioned list of formatting changes required for your final resubmission.

Please let us know any time should you have additional questions related.

Best regards,

Daniel Klimmeck

Daniel Klimmeck PhD
Senior Editor
The EMBO Journal

******

Formatting changes required for the revised version of the manuscript:

>> As you might have seen on our webpage, every paper at the EMBO Journal includes a 'Synopsis', displayed on the html and freely accessible to all readers. The synopsis includes a 'model' figure as well as 2-5 one-short-sentence bullet points that summarize the article. I would appreciate if you could provide this figure and the bullet points.

>> Please add up to five keywords to your study.

>> Please provide the main manuscript text as .docx file.

>> Limit the abstract to maximally 175 words.

>> Authors: please correct the order of the author names in our system so that they match the order in which they are listed in the manuscript text. Y. H. is in the manuscript text but not in our online system.

>> Author Contributions: Remove the author contributions information from the manuscript text. Note that CRediT has replaced the traditional author contributions section as of now because it offers a systematic machine-readable author contributions format that allows for more effective research assessment. and use the free text boxes beneath each contributing author's name to add specific details on the author's contribution.

More information is available in our guide to authors.
https://www.embopress.org/page/journal/14602075/authorguide

>> Adjust the title of the 'Conflict of Interest' section to 'Disclosure and Competing Interests Statement'.
Please revisit adding a statement for H.Z. as to his affiliation: Bioland Laboratory, Guangzhou, listed as a Manufacturer/Factory (https://www.made-in-china.com/showroom/6c126545b456c0b4/).

>> Provide a completed Author Checklist.

>> Section order should be corrected as follows: title page with complete author information, abstract, keywords, introduction, results, discussion, methods, data availability section, acknowledgements, disclosure and competing interests statement, references, main figure legends, tables, expanded figure legends.´.

>> Figures in separate files: Figures should be removed from the manuscript text and uploaded as individual, high resolution figure files. Legends should be placed after the References.

>> Please add a Reagents and Tools table to the Methods section, as a separate file using the existing template in the Guide For Authors, listing key reagents, experimental models, software and relevant equipment.

Please compare also our Guide-to-Authors instructions:
https://www.embopress.org/page/journal/14602075/authorguide#structuredmethods

>> Funding: please enter all funding information in the list of funders in our online system, as they will be linked in PubMed.

>> Dataset EV legends: Please correct the nomenclature for Expanded View Table 1 to "Table EV".

>> Please provide source data for the study as to the separate request e-mail. Source data should be uploaded as one (zipped) file per figure.

>> Data availability section: please add a statement: 'No large-scale data amenable to data repository deposition were generated in this study.'.

>> Consider additional changes and comments from our production team as indicated below:

- Figure legends:
1. Please note that the exact p values are not provided in the legends of figures 1B, C, E, F, H, L, M, Q, R, T, U; 2D, F; 3A, D, F, G, M, N, Q; 4C, G, H, I, L, M; EV1 E, F, I, J, K, L, M, N, O; EV2 A, D, F, H, I, J, K, L, M, N, O, Q, R; EV3 A, B, C, E, H; EV4 D, E, F, H, I, J, M, O, P; EV5 C, E, I, L, M.
2. Please note that information related to n is missing in the legends of figures 1N, O; 4L, M; EV4 G
3. Please note that n=2 in figures EV4 A, B, R
4. Although 'n' is provided, please describe the nature of entity for 'n' in the legends of figures 1B, C, E, F, H, L, M, Q, R, T, U; 2D, F; 3A, D, F, G, M, N, Q; 4C, F, G, H, I; EV1 C, F, E, I-L, M-P; EV2 A, D, F, H, I, J, K, L, M, N, O, Q, R; EV3 A, B, C, E, H; EV4 C, D, E, F, H, I, J, M, O, P, S; EV5 C, G, H, I, J, K, L, M, N;
5. Please note that the error bars are not defined in the legends of figures 1N, O; 4L, M; EV1 E, EV4 A, B, G

Further information is available in our Guide For Authors: https://link.springer.com/journal/44318/submission-guidelines

Please use the link below to submit your revision:

https://emboj.msubmit.net/cgi-bin/main.plex

****

Dear Dr Qin,

Thank you for submitting your amended appeal manuscript (EMBOJ-2024-119405R-Q) to The EMBO Journal, together with a rebuttal point-by-point response to the issues raised during peer-review. As mentioned, your revised study was sent back to the previous referees for their scientific reassessment, and we have received detailed re-reports from both of them, which I enclose below. As you will see, the referees state that the work has been enhanced by the revision and they are now in favour of publication, pending reasonable additional revision.

Thus, we are pleased to inform you that we concluded that we can invite you to amend your manuscript according to expert #2's input, to the progress towards publication in The EMBO Journal.

Please carefully consider the remaining points raised by referee #2 by adding revisiting data presentation and statistics, and by introducing caveats and relativise claims in the manuscript text where appropriate.

Also, we now need you to take care of a number of minor issues related to formatting and data annotation, which I will share shortly in a separate message, together with additional changes and requests by our production team for Source Data provision.

Please submit a revised version of the manuscript using the link enclosed below, addressing the advisor's comments.

Thank you again for giving us the chance to consider your manuscript for The EMBO Journal, I look forward to hearing from you and receiving your final revised version of the manuscript.

Kind regards,

Daniel Klimmeck

Daniel Klimmeck PhD
Senior Editor
The EMBO Journal

Instructions for preparing your revised manuscript:

Read our guidance for manuscript revisions and related editorial policies: https://link.springer.com/journal/44318/submission-guidelines#cms-Revised-submissions

Please also check that the title and abstract of the manuscript are brief, yet explicit, even to non-specialists.

When assembling figures, please refer to our figure preparation guideline in order to ensure proper formatting and readability in print as well as on screen:
https://media.springernature.com/original/springer-cms/rest/v1/content/27825798/data/v1

IMPORTANT: When you send the revision we will require
- a point-by-point response to the referees' comments, with a detailed description of the changes made (as a word file).
- a word file of the manuscript text.
- individual production quality figure files (one file per figure)
- a complete author checklist
- Expanded View files (replacing Supplementary Information)
- a Reagents and Tools Table as part of the Methods section

Please remember: Digital image enhancement is acceptable practice, as long as it accurately represents the original data and conforms to community standards. If a figure has been subjected to significant electronic manipulation, this must be noted in the figure legend or in the 'Methods' section. The editors reserve the right to request original versions of figures and the original images that were used to assemble the figure.

We realize that it is difficult to revise to a specific deadline. In the interest of protecting the conceptual advance provided by the work, we recommend a revision within 3 months (3rd May 2026). Please discuss the revision progress ahead of this time with the editor if you require more time to complete the revisions. Use the link below to submit your revision:

https://emboj.msubmit.net/cgi-bin/main.plex

Referee #1:

In this revised manuscript, the authors provide further evidence for their mechanism that TDH activity can impact the redox status of mESCs, such that the defects of mitochondrial dysfunction can no longer be surmounted by treatment with electron acceptors.My biggest issues with the prior draft were some overstated claims, missing controls, and some seemingly unfair

experimental comparisons. The authors have done a commendable job of resolving those issues in the revised manuscript and response to reviewers document. As such, my major concerns have been alleviated and I believe that the revised manuscript is acceptable for publication.

Referee #2:

General summary and opinion

This revised manuscript investigates how mitochondrial electron transport chain inhibition induces a reversible paused state in pluripotent stem cells and proposes that sustained NADH reductive stress underlies this response. The authors identify threonine dehydrogenase as a major contributor to reductive stress in mouse embryonic stem cells and propose that distinct metabolic pathways generate a similar redox burden in human pluripotent stem cells. The study further links intrinsic reductive stress to suppression of pyruvate dehydrogenase activity, altered acetyl CoA availability, and increased genomic and epigenetic instability when pausing is relieved.

The revision includes substantial new experimental work that addresses many of the concerns raised in the initial review, particularly regarding redox regulation, PDH dependence, and relevance to human pluripotent stem cells. Overall, the study tackles an important question at the intersection of mitochondrial metabolism, redox biology, and cell state regulation, and the experimental effort in the revision is significant. However, several key conclusions remain overstated relative to the data presented, primarily due to language that implies deliberate, programmed, or conserved quality control mechanisms without direct evidence. These issues largely concern interpretation and framing rather than missing experiments, but they are essential to address in order to fully support the manuscript's conclusions.

Major concerns

Overinterpretation of deliberate or programmed quality control mechanisms

Throughout the Abstract, Results, and Discussion, the manuscript describes sustained reductive stress as a deliberate or systemic quality control mechanism that preserves genomic and epigenetic stability. While the data convincingly show that reductive stress correlates with proliferative pausing and that relieving this stress permits proliferation at the cost of increased genomic instability, the study does not demonstrate that cells actively induce reductive stress as a regulated quality control pathway. The data are more consistent with intrinsic metabolic constraints and population level selection against slower growing cells.

The authors should revise the language throughout the manuscript to avoid implying intent or programming. Phrasing that emphasizes intrinsic metabolic constraints or selection like effects is more appropriate and consistent with the evidence.

Strength of causal claims regarding threonine dehydrogenase in mouse embryonic stem cells

The revised data support an important role for threonine dehydrogenase in contributing to NADH burden and limiting the effectiveness of electron acceptors in mouse embryonic stem cells. However, the manuscript often presents TDH as the primary or dominant driver of the paused state. This conclusion is stronger than warranted given that pyruvate and TDH inhibition each partially reduce reductive stress, and that rescue effects are context dependent.

In addition, several datasets used to support TDH specificity and dominance are presented as representative examples with n equal to one. While these data are informative, they should not be used to justify strong quantitative or exclusive claims. The authors should recalibrate the language to describe TDH as a major contributor rather than the sole or primary determinant of pausing.

Human pluripotent stem cell mechanism is not pathway conserved

The authors propose that reductive stress is a conserved driver of pausing in both mouse and human pluripotent stem cells, but that the metabolic source of NADH differs between species. The new data showing that combined inhibition of SHMT1 or SHMT2 and MTHFD2 rescues self renewal and reduces NADH to NAD plus ratio in human pluripotent stem cells represent a significant advance.

However, the manuscript currently states that mitochondrial one carbon metabolism is identified as the driver of reductive stress in human pluripotent stem cells. Given that this conclusion is based on pharmacologic interactions rather than direct flux measurements or genetic perturbations, this language is too strong. The authors should rephrase these claims to state that the data support a model in which mitochondrial one carbon metabolism contributes to reductive stress in human pluripotent stem cells.

Interpretation of LbNOX versus Ndi1 experiments

The comparison between Ndi1 expression and lack of rescue by LbNOX is used to argue that unresolved NADH to NAD plus imbalance is the principal driver of the paused state in mouse embryonic stem cells. While these experiments are informative, the failure of LbNOX to rescue may reflect limited enzyme activity, compartmental access, or inability to access the relevant NADH pool, rather than persistence of reductive stress per se.

The authors should acknowledge these alternative interpretations and avoid presenting the LbNOX results as definitive evidence of redox causality.

Use of metabolic rewiring language without flux based evidence

The manuscript frequently describes TDH to PDH or LDH to PDH metabolic rewiring. Although functional dependence on PDH and acetyl CoA is well supported by acetate rescue and PDH inhibition experiments, no isotope tracing data are presented to establish metabolic flux or pathway directionality.

The authors should replace rewiring terminology with language describing inferred metabolic adaptation or functional dependence unless flux based evidence is added.

Minor concerns

Lactate supplementation experiments are discussed conceptually and were referenced in the rebuttal, but corresponding data are not clearly presented in the revised manuscript. If these experiments were performed, the data should be included, even as supplementary figures, as they directly support the proposed redox model.

Sample sizes for key experiments such as karyotyping and some metabolic measurements are low and should be clearly indicated in figure legends and discussed cautiously in the text.

The manuscript relies largely on unpaired two tailed Student's t tests despite multiple comparisons and dose response analyses. Clearer justification or more consistent statistical handling would strengthen confidence in the conclusions.

The embryo pausing experiments are compelling at the phenotypic level, but mechanistic links to threonine dehydrogenase or one carbon metabolism are not directly tested. The manuscript should avoid implying mechanistic equivalence and instead frame these findings as consistent with, but not definitive proof of, the proposed model.

Non essential suggestions

Genetic perturbation of threonine dehydrogenase or flux based isotope tracing in future work would further strengthen conclusions regarding pathway specificity and directionality.

A schematic explicitly distinguishing mouse specific and human specific sources of NADH while emphasizing shared redox logic would improve clarity for readers.

Expanded discussion of whether similar redox driven pausing mechanisms may operate in differentiated cells or other stem cell types would broaden the impact of the study.

Referee #1:

In this revised manuscript, the authors provide further evidence for their mechanism that TDH activity can impact the redox status of mESCs, such that the defects of mitochondrial dysfunction can no longer be surmounted by treatment with electron acceptors. My biggest issues with the prior draft were some overstated claims, missing controls, and some seemingly unfair experimental comparisons. The authors have done a commendable job of resolving those issues in the revised manuscript and response to reviewers document. As such, my major concerns have been alleviated and I believe that the revised manuscript is acceptable for publication.

Response

We thank the reviewer for the positive evaluation of our revised manuscript and for acknowledging the improvements made in response to prior concerns. We also appreciate your thoughtful feedback throughout the review process.

Referee #2:

General summary and opinion

This revised manuscript investigates how mitochondrial electron transport chain inhibition induces a reversible paused state in pluripotent stem cells and proposes that sustained NADH reductive stress underlies this response. The authors identify threonine dehydrogenase as a major contributor to reductive stress in mouse embryonic stem cells and propose that distinct metabolic pathways generate a similar redox burden in human pluripotent stem cells. The study further links intrinsic reductive stress to suppression of pyruvate dehydrogenase activity, altered acetyl CoA availability, and increased genomic and epigenetic instability when pausing is relieved.

The revision includes substantial new experimental work that addresses many of the concerns raised in the initial review, particularly regarding redox regulation, PDH dependence, and relevance to human pluripotent stem cells. Overall, the study tackles an important question at the intersection of mitochondrial metabolism,

redox biology, and cell state regulation, and the experimental effort in the revision is significant. However, several key conclusions remain overstated relative to the data presented, primarily due to language that implies deliberate, programmed, or conserved quality control mechanisms without direct evidence. These issues largely concern interpretation and framing rather than missing experiments, but they are essential to address in order to fully support the manuscript's conclusions.

Response

We thank the reviewer for their positive assessment of our study and for recognizing the efforts we have made to address the prior concerns.

Major concerns

Overinterpretation of deliberate or programmed quality control mechanisms

Throughout the Abstract, Results, and Discussion, the manuscript describes sustained reductive stress as a deliberate or systemic quality control mechanism that preserves genomic and epigenetic stability. While the data convincingly show that reductive stress correlates with proliferative pausing and that relieving this stress permits proliferation at the cost of increased genomic instability, the study does not demonstrate that cells actively induce reductive stress as a regulated quality control pathway. The data are more consistent with intrinsic metabolic constraints and population level selection against slower growing cells. The authors should revise the language throughout the manuscript to avoid implying intent or programming. Phrasing that emphasizes intrinsic metabolic constraints or selection like effects is more appropriate and consistent with the evidence.

Response

We thank the reviewer for this important conceptual point. We agree that our original wording could be interpreted as implying that reductive stress acts as a deliberate or evolutionarily programmed quality-control mechanism, which was not our intention. We have therefore revised it throughout the manuscript, changing the description of sustained reductive stress as a deliberate or programmed quality control mechanism to intrinsic metabolic constraints or selection-like effects.

(See revised text highlighted in yellow in the manuscript, pages 2, 4, 8, 9, 11, and 14; lines 33-36, 89, 90, 198-200, 238, 239, 243-245, 247, 248, 323-325, and 402-408).

Strength of causal claims regarding threonine dehydrogenase in mouse embryonic stem cells

The revised data support an important role for threonine dehydrogenase in contributing to NADH burden and limiting the effectiveness of electron acceptors in mouse embryonic stem cells. However, the manuscript often presents TDH as the primary or dominant driver of the paused state. This conclusion is stronger than warranted given that pyruvate and TDH inhibition each partially reduce reductive stress, and that rescue effects are context dependent.

In addition, several datasets used to support TDH specificity and dominance are presented as representative examples with n equal to one. While these data are informative, they should not be used to justify strong quantitative or exclusive claims. The authors should recalibrate the language to describe TDH as a major contributor rather than the sole or primary determinant of pausing.

Response

We thank the reviewer for this important and constructive comment. We agree that some of our original wording may have unintentionally suggested that TDH is the primary or dominant driver of the paused state upon ETC inhibition. We have therefore revised the manuscript throughout to more accurately reflect that TDH represents a major contributor to NADH reductive stress and to ETC inhibition–induced pausing in mESCs, rather than the sole determinant of this phenotype.

We also appreciate the reviewer’s concern regarding datasets presented as representative examples with n = 1. We agree that such data should not be used to support strong quantitative or exclusive claims. Accordingly, we have revised the text and figure legends to avoid language implying quantitative dominance and to ensure that these datasets are presented only as illustrative observations rather than as quantitative evidence.

All relevant statements throughout the manuscript have been adjusted accordingly. (See revised text highlighted in yellow in the manuscript, pages 2, 4, 5, 7, 36, 38, 42 and 44 ; lines 33, 83-86, 100, 184, 185, 910, 932-937, 988-990, 1117-1119, 1133-135, and 1176-1179).

Human pluripotent stem cell mechanism is not pathway conserved

The authors propose that reductive stress is a conserved driver of pausing in both mouse and human pluripotent stem cells, but that the metabolic source of NADH differs between species. The new data showing that combined inhibition of SHMT1 or SHMT2 and MTHFD2 rescues self-renewal and reduces NADH to NAD plus ratio in human pluripotent stem cells represent a significant advance. However, the manuscript currently states that mitochondrial one carbon metabolism is identified as the driver of reductive stress in human pluripotent stem cells. Given that this conclusion is based on pharmacologic interactions rather than direct flux measurements or genetic perturbations, this language is too strong. The authors should rephrase these claims to state that the data support a model in which mitochondrial one carbon metabolism contributes to reductive stress in human pluripotent stem cells.

Response

We thank the reviewer for this constructive comment and appreciate your recognition of the significance of our new data in human pluripotent stem cells. We agree that reductive stress is a conserved driver of the paused state across both species, however, the excess NADH pool stems from distinct metabolic pathways in mice and humans, and these NADH metabolic sources are not evolutionarily conserved. We also acknowledge that pharmacological perturbation alone is insufficient to make a definitive claim without complementary genetic perturbations or flux measurements. Therefore, we have revised the relevant wording from mitochondrial one-carbon metabolism driving reductive stress to that of mitochondrial one-carbon metabolism contributing to reductive stress in human pluripotent stem cells upon ETC inhibition.

(See revised text highlighted in yellow in the manuscript, page 14, lines 397-399, 404, and 405).

Interpretation of LbNOX versus Ndi1 experiments

The comparison between Ndi1 expression and lack of rescue by LbNOX is used to argue that unresolved NADH to NAD plus imbalance is the principal driver of the paused state in mouse embryonic stem cells. While these experiments are informative, the failure of LbNOX to rescue may reflect limited enzyme activity, compartmental access, or inability to access the relevant NADH pool, rather than persistence of reductive stress per se. The authors should acknowledge these alternative interpretations and avoid presenting the LbNOX results as definitive evidence of redox causality.

Response

We thank the reviewer for raising this important point regarding the interpretation of *Lb*NOX and *Ndi1* experiments. We agree that the failure of *Lb*NOX to rescue the ETCi-induced paused proliferation in mESCs may not reflect persistent reductive stress *per se*, but instead could be attributed to insufficient *Lb*NOX enzyme activity, limited compartmental excess, or an inability to access the relevant NADH pool in our experimental system. We have therefore revised the manuscript to acknowledge these alternative interpretations and have softened our claims, no longer presenting the *Lb*NOX results as definitive evidence for redox causality in driving the paused state.
(See revised text highlighted in yellow in the manuscript, page 6, lines 137-141).

Use of metabolic rewiring language without flux based evidence

The manuscript frequently describes TDH to PDH or LDH to PDH metabolic rewiring. Although functional dependence on PDH and acetyl CoA is well supported by acetate rescue and PDH inhibition experiments, no isotope tracing data are presented to establish metabolic flux or pathway directionality. The authors should replace rewiring terminology with language describing inferred

metabolic adaptation or functional dependence unless flux based evidence is added.

Response

We thank the reviewer for this important clarification. We agree that the term "metabolic rewiring" requires direct quantitative flux measurements via isotope tracing. Our data demonstrate an inferred metabolic adaptation or functional dependence on PDH activation for acetyl-CoA when TDH is inhibited. Therefore, we acknowledge that "rewiring" may overstate the mechanistic resolution provided by our current data. Accordingly, we have revised the manuscript throughout to replace the term "metabolic rewiring" with phrasing such as inferred metabolic adaptation or functional dependence, which more accurately reflects our experimental findings.

(See revised text highlighted in yellow in the manuscript, pages 10 and 11; lines 275, 281, 284, 286, 295, 301, 313, and 323-325).

Minor concerns

Lactate supplementation experiments are discussed conceptually and were referenced in the rebuttal, but corresponding data are not clearly presented in the revised manuscript. If these experiments were performed, the data should be included, even as supplementary figures, as they directly support the proposed redox model.

Response

We thank the reviewer for noting that the lactate supplementation data referenced in our rebuttal were not clearly presented in the revised manuscript. We have now included these data as EV Figure 1M,1N and 5J. These data show that lactate supplementation increases the NADH/NAD$^+$ ratio, which is consistent with the modulation of cytosolic redox balance via LDH-dependent NAD$^+$–NADH interconversion, and thus support our redox model that excess reductive stress inhibits cell proliferation.

(See the revised text highlighted in yellow in the manuscript, page 6 and 13; lines 143-145 and 370-372).

Sample sizes for key experiments such as karyotyping and some metabolic measurements are low and should be clearly indicated in figure legends and discussed cautiously in the text.

Response

We thank the reviewer for highlighting the low sample sizes of key experiments presented in the manuscript. We have now clearly indicated the sample sizes of all key experiments (including karyotyping and metabolic measurements) in the figure legends and discussed these low sample sizes cautiously in the text.

(See revised text highlighted in yellow in the manuscript, pages 36, 38, 39, 42, 44, and 45; lines 932-937, 1001-1006, 1019-1022, 1027-1029, 1133-1135, 1176-1179, and 1212-1217).

The manuscript relies largely on unpaired two tailed Student's t tests despite multiple comparisons and dose response analyses. Clearer justification or more consistent statistical handling would strengthen confidence in the conclusions.

Response

We thank the reviewer for raising this important concern about our statistical analyses. We have now revised the statistical handling where they are needed, with appropriate adjustments for multiple comparisons and dose-response analyses, and the revisions are particularly thorough for the blastocyst experiment. (See revised text highlighted in yellow in the manuscript, page 45, lines 1234-1239).

The embryo pausing experiments are compelling at the phenotypic level, but mechanistic links to threonine dehydrogenase or one carbon metabolism are not directly tested. The manuscript should avoid implying mechanistic equivalence and instead frame these findings as consistent with, but not definitive proof of, the proposed model.

Response

We thank the reviewer for this insightful concern. We agree that our embryo pausing experiments are phenotypically compelling, but we have not directly tested

the mechanistic links between this phenotype and TDH or one-carbon metabolism. We therefore acknowledge that the manuscript previously implied an overstated mechanistic equivalence, and we have revised the relevant wording throughout to frame these findings as consistent with our proposed model, rather than definitive proof of it—this includes softening language related to TDH/one-carbon metabolism in 2C embryo and blastocyst pausing.
(See revised text highlighted in yellow in the manuscript, page 14, lines 410 and 411).

Non essential suggestions
Genetic perturbation of threonine dehydrogenase or flux based isotope tracing in future work would further strengthen conclusions regarding pathway specificity and directionality.
Response
We thank to the reviewer for this valuable suggestion. We agree with the reviewer's comments and have now incorporated this important suggestion in our discussion. (See revised text highlighted in yellow in the manuscript, page 14, lines 420-422).

A schematic explicitly distinguishing mouse specific and human specific sources of NADH while emphasizing shared redox logic would improve clarity for readers.
Response
We thank the reviewer for highlighting this important point. We have now included a revised schematic illustration that explicitly distinguishes the mouse-specific and human-specific sources of NADH, while also emphasizing the shared redox logic between the two species for improved reader clarity.
(See revised text highlighted in yellow in the manuscript, page 14, lines 402-408).

Expanded discussion of whether similar redox driven pausing mechanisms may operate in differentiated cells or other stem cell types would broaden the impact of the study.
Response

We thank the reviewer for this important suggestion. We have now expanded the discussion section to address whether similar redox-driven pausing mechanisms operate in differentiated cells and other stem cell types.

(See revised text highlighted in yellow in the manuscript, page 14, lines 424-426).

2nd Revision - Editorial Decision 10th Apr 2026

Dear Dr Qin,

Thank you for submitting the revised version of your manuscript. I have now evaluated your amended manuscript and concluded that the remaining minor concerns have been sufficiently addressed.

I am thus pleased to inform you that your manuscript has been accepted for publication in the EMBO Journal.

Your manuscript will be processed for publication by EMBO Press. It will be copy edited and you will receive page proofs prior to publication. Please note that you will be contacted by Springer Nature Author Services to complete licensing and payment information.

Please note that it is The EMBO Journal policy for the transcript of the editorial process (containing referee reports and your response letter) to be published as an online supplement to each paper. If you do NOT want this, you will need to inform the Editorial Office via email immediately. More information is available here: https://www.embopress.org/transparent-process#Review_Process

You may qualify for financial assistance for your publication charges - either via a Springer Nature fully open access agreement or an EMBO initiative. Check your eligibility: https://www.embopress.org/page/journal/14602075/authorguide#chargesguide

Should you be planning a Press Release on your article, please get in contact with embo_production@springernature.com as early as possible in order to coordinate publication and release dates.

If you have any questions, please do not hesitate to contact the Editorial Office. Thank you for your contribution to The EMBO Journal.

Thank you again for this contribution to The EMBO Journal and congratulations on a successful publication! Please consider us again in the future for your most exciting work.

Best regards,

Daniel Klimmeck

Daniel Klimmeck, PhD
Senior Editor
The EMBO Journal
EMBO
Postfach 1022-40
Meyerhofstrasse 1
D-69117 Heidelberg
contact@embojournal.org

*****
Please note that it is The EMBO Journal policy for the transcript of the editorial process (containing referee reports and your response letters) to be published as an online supplement to each paper. If you should prefer removal of any referee-only figures included in the point-by-point response(s), e.g. because they may still be used for future publication or because they have been reproduced from published work by others, please do let us know immediately via response email.

More information is available here: https://link.springer.com/partners/embo-press/editorial-policies#Peer%20review

** Click here to be directed to your login page: https://emboj.msubmit.net

EMBO Press Author Checklist

Corresponding Author Name: Qin Baoming	
Journal Submitted to: The EMBO Journal	
Manuscript Number: EMBOJ-2024-119405R1	

USEFUL LINKS FOR COMPLETING THIS FORM
The EMBO Journal - Author Guidelines
EMBO Reports - Author Guidelines
Molecular Systems Biology - Author Guidelines
EMBO Molecular Medicine - Author Guidelines

Reporting Checklist for Life Science Articles (updated January
This checklist is adapted from Materials Design Analysis Reporting (MDAR) Checklist for Authors. MDAR establishes a minimum set of requirements in transparent reporting in the life sciences (see Statement of Task: 10.31222/osf.io/9sm4x). Please follow the journal's guidelines in preparing your
Please note that a copy of this checklist will be published alongside your article.

Abridged guidelines for figures
1. Data
The data shown in figures should satisfy the following conditions:
- the data were obtained and processed according to the field's best practice and are presented to reflect the results of the experiments in an accurate and unbiased manner.
- ideally, figure panels should include only measurements that are directly comparable to each other and obtained with the same assay.
- plots include clearly labeled error bars for independent experiments and sample sizes. Unless justified, error bars should not be shown for technical
- if n<5, the individual data points from each experiment should be plotted. Any statistical test employed should be justified.
- Source Data should be included to report the data underlying figures according to the guidelines set out in the authorship guidelines on Data

2. Captions
Each figure caption should contain the following information, for each panel where they are relevant:
- a specification of the experimental system investigated (eg cell line, species name).
- the assay(s) and method(s) used to carry out the reported observations and measurements.
- an explicit mention of the biological and chemical entity(ies) that are being measured.
- an explicit mention of the biological and chemical entity(ies) that are altered/varied/perturbed in a controlled manner.
- the exact sample size (n) for each experimental group/condition, given as a number, not a range;
- a description of the sample collection allowing the reader to understand whether the samples represent technical or biological replicates (including how many animals, litters, cultures, etc.).
- a statement of how many times the experiment shown was independently replicated in the laboratory.
- definitions of statistical methods and measures:
  - common tests, such as t-test (please specify whether paired vs. unpaired), simple χ2 tests, Wilcoxon and Mann-Whitney tests, can be unambiguously identified by name only, but more complex techniques should be described in the methods section;
  - are tests one-sided or two-sided?
  - are there adjustments for multiple comparisons?
  - exact statistical test results, e.g., P values = x but not P values < x;
  - definition of 'center values' as median or average;
  - definition of error bars as s.d. or s.e.m.

Please complete ALL of the questions below.
Select "Not Applicable" only when the requested information is not relevant for your study.

Materials

Newly Created Materials	Information included in the manuscript?	In which section is the information available? (Reagents and Tools Table, Materials and Methods, Figures, Data Availability Section)
New materials and reagents need to be available; do any restrictions apply?	Not Applicable	Not Applicable

Antibodies	Information included in the manuscript?	In which section is the information available? (Reagents and Tools Table, Materials and Methods, Figures, Data Availability Section)
For **antibodies** provide the following information:<br>- Commercial antibodies: RRID (if possible) or supplier name, catalogue number and or/clone number<br>- Non-commercial: RRID or citation	Yes	The names of antibodies, sources and catalogue number are given in reagents and tools table and methods

DNA and RNA sequences	Information included in the manuscript?	In which section is the information available? (Reagents and Tools Table, Materials and Methods, Figures, Data Availability Section)
**Short novel DNA or RNA including primers, probes**: provide the sequences.	Yes	the sequences of the primers, shRNA, sgRNA are given in reagents and tools table

Cell materials	Information included in the manuscript?	In which section is the information available? (Reagents and Tools Table, Materials and Methods, Figures, Data Availability Section)
**Cell lines:** Provide species information, strain. Provide accession number in repository **OR** supplier name, catalog number, clone number, and/**OR** RRID.	Yes	The details regarding the cell lines are given in reagents and tools table
**Primary cultures:** Provide species, strain, sex of origin, genetic modification status.	Yes	Not Applicable
Report if the cell lines were recently **authenticated** (e.g., by STR profiling) and tested for mycoplasma contamination.	Yes	Not Applicable

Experimental animals	Information included in the manuscript?	In which section is the information available? (Reagents and Tools Table, Materials and Methods, Figures, Data Availability Section)
**Laboratory animals or Model organisms:** Provide species, strain, sex, age, genetic modification status. Provide accession number in repository **OR** supplier name, catalog number, clone number, **OR** RRID.	Yes	The details regarding the laboratory animals are given in reagents and tools table and methods
**Animal observed in or captured from the field:** Provide species, sex, and age where possible.	Not Applicable	Not Applicable
Please detail **housing and husbandry conditions**.	Yes	The details of laboratory animal housing condition are given in methods

Plants and microbes	Information included in the manuscript?	In which section is the information available? (Reagents and Tools Table, Materials and Methods, Figures, Data Availability Section)
**Plants:** provide species and strain, ecotype and cultivar where relevant, unique accession number if available, and source (including location for collected wild specimens).	Not Applicable	Not Applicable
**Microbes:** provide species and strain, unique accession number if available, and source.	Not Applicable	Not Applicable

Human research participants	Information included in the manuscript?	In which section is the information available? (Reagents and Tools Table, Materials and Methods, Figures, Data Availability Section)
If collected and within the bounds of privacy constraints report on age, sex and gender or ethnicity for all study participants.	Not Applicable	Not Applicable

Core facilities	Information included in the manuscript?	In which section is the information available? (Reagents and Tools Table, Materials and Methods, Figures, Data Availability Section)
If your work benefited from core facilities, was their service mentioned in the acknowledgments section?	Not Applicable	Not Applicable

Design

Study protocol	Information included in the manuscript?	In which section is the information available? (Reagents and Tools Table, Materials and Methods, Figures, Data Availability Section)
If study protocol has been **pre-registered, provide DOI in the manuscript**. For clinical trials, provide the trial registration number **OR** cite DOI.	Not Applicable	Not Applicable
Report the **clinical trial registration number** (at ClinicalTrials.gov or equivalent), where applicable.	Not Applicable	Not Applicable

Laboratory protocol	Information included in the manuscript?	In which section is the information available? (Reagents and Tools Table, Materials and Methods, Figures, Data Availability Section)
Provide DOI OR other citation details if **external detailed step-by-step protocols** are available.	Yes	The citations of the protocols are described in the methods and listed in the references section

Experimental study design and statistics	Information included in the manuscript?	In which section is the information available? (Reagents and Tools Table, Materials and Methods, Figures, Data Availability Section)
Include a statement about **sample size** estimate even if no statistical methods were used.	Yes	The details of samples size and statistical methods are given in figure legends and methods.
Were any steps taken to minimize the effects of subjective bias when allocating animals/samples to treatment (e.g. **randomization procedure**)? If yes, have they been described?	Not Applicable	Not Applicable
Include a statement about **blinding** even if no blinding was done.	Not Applicable	Not Applicable
Describe **inclusion/exclusion criteria** if samples or animals were excluded from the analysis. Were the criteria pre-established?<br><br>If sample or data points were omitted from analysis, report if this was due to attrition or intentional exclusion and provide justification.	Not Applicable	Not Applicable
For every figure, are **statistical tests** justified as appropriate? Do the data meet the assumptions of the tests (e.g., normal distribution)? Describe any methods used to assess it. Is there an estimate of variation within each group of data? Is the variance similar between the groups that are being statistically compared?	Yes	The details of statistical methods are given in figure legends and methods.

Sample definition and in-laboratory replication	Information included in the manuscript?	In which section is the information available? (Reagents and Tools Table, Materials and Methods, Figures, Data Availability Section)
In the figure legends: state number of times the experiment was **replicated** in laboratory.	Yes	It is described in the figure legends
In the figure legends: define whether data describe **technical or biological replicates**.	Yes	It is described in the figure legends

Ethics

Ethics	Information included in the manuscript?	In which section is the information available? (Reagents and Tools Table, Materials and Methods, Figures, Data Availability Section)
Studies involving **human participants**: State details of **authority granting ethics approval** (IRB or equivalent committee(s), provide reference number for approval.	Not Applicable	Not Applicable
Studies involving **human participants**: Include a statement confirming that **informed consent** was obtained from all subjects and that the experiments conformed to the principles set out in the WMA Declaration of Helsinki and the Department of Health and Human Services Belmont Report.	Not Applicable	Not Applicable
Studies involving **human participants: For publication of patient photos**, include a statement confirming that consent to publish was obtained.	Not Applicable	Not Applicable
Studies involving experimental **animals**: State details of **authority granting ethics approval** (IRB or equivalent committee(s), provide reference number for approval. Include a statement of compliance with ethical regulations.	Yes	It is described in the methods
Studies involving **specimen and field samples:** State if relevant **permits** obtained, provide details of authority approving study; if none were required, explain why.	Not Applicable	Not Applicable

Dual Use Research of Concern (DURC)	Information included in the manuscript?	In which section is the information available? (Reagents and Tools Table, Materials and Methods, Figures, Data Availability Section)
Could your study fall under dual use research restrictions? Please check biosecurity documents and list of **select agents and toxins** (CDC): https://www.selectagents.gov/sat/list.htm	Not Applicable	Not Applicable
If you used a select agent, is the security level of the lab appropriate and reported in the manuscript?	Not Applicable	Not Applicable
If a study is subject to dual use research of concern regulations, is the name of the **authority granting approval and reference number** for the regulatory approval provided in the manuscript?	Not Applicable	Not Applicable

Reporting

The MDAR framework recommends adoption of discipline-specific guidelines, established and endorsed through community initiatives. Journals have their own policy about requiring specific guidelines and recommendations to complement MDAR.

Adherence to community standards	Information included in the manuscript?	In which section is the information available? (Reagents and Tools Table, Materials and Methods, Figures, Data Availability Section)
State if relevant guidelines or checklists (e.g., **ICMJE, MIBBI, ARRIVE, PRISMA**) have been followed or provided.	Not Applicable	Not Applicable
For **tumor marker prognostic studies,** we recommend that you follow the **REMARK** reporting guidelines (see link list at top right). See author guidelines, under 'Reporting Guidelines'. Please confirm you have followed these guidelines.	Not Applicable	Not Applicable
For **phase II and III randomized controlled trials**, please refer to the **CONSORT** flow diagram (see link list at top right) and submit the CONSORT checklist (see link list at top right) with your submission. See author guidelines, under 'Reporting Guidelines'. Please confirm you have submitted this list.	Not Applicable	Not Applicable

Data Availability

Data availability	Information included in the manuscript?	In which section is the information available? (Reagents and Tools Table, Materials and Methods, Figures, Data Availability Section)
Have **primary datasets** been deposited according to the journal's guidelines (see 'Data Deposition' section) and the respective accession numbers provided in the Data Availability Section?	Not Applicable	Not Applicable
Were **human clinical and genomic datasets** deposited in a public access-controlled repository in accordance to ethical obligations to the patients and to the applicable consent agreement?	Not Applicable	Not Applicable
Are **computational models** that are central and integral to a study available without restrictions in a machine-readable form? Were the relevant accession numbers or links provided?	Not Applicable	Not Applicable
If publicly available data were reused, provide the respective **data citations in the reference list**.	Yes	Yes, all publicaly available data reused in this studs has been cited and listed in the references section.
